# Nanohoops Favour Light‐Induced Energy Transfer over Charge Separation in Porphyrin/[10]CPP/Fullerene Rotaxanes

**DOI:** 10.1002/anie.202413404

**Published:** 2024-11-11

**Authors:** Fabian Schwer, Simon Zank, Markus Freiberger, Fabian M. Steudel, Niklas Geue, Lei Ye, Perdita E. Barran, Thomas Drewello, Dirk M. Guldi, Max von Delius

**Affiliations:** ^1^ Institute of Organic Chemistry Ulm University Albert-Einstein-Allee 11 89081 Ulm Germany; ^2^ Department of Chemistry and Pharmacy, FAU Profile Center Solar, Interdisciplinary Center for Molecular Materials (ICMM) Friedrich-Alexander Universität Erlangen-Nürnberg Egerlandstrasse 3 91058 Erlangen Germany; ^3^ Michael Barber Centre for Collaborative Mass Spectrometry, Manchester Institute of Biotechnology, Department of Chemistry The University of Manchester 131 Princess Street Manchester M1 7DN UK

**Keywords:** Supramolecular chemistry, rotaxanes, charge separation, energy transfer, nanohoops

## Abstract

[2]Rotaxanes offer unique opportunities for studying and modulating charge separation and energy transfer, because the mechanical bond allows the robust, yet spatially dynamic tethering of photoactive groups. In this work, we synthesized [2]rotaxane triads comprising a central (aza)[10]CPP⊃C_60_ bis‐adduct complex and two zinc porphyrin stoppers to address how the movable nanohoop affects light‐induced charge separation and energy transfer between the rotaxane subcomponents. We found that neither the parent nanohoop [10]CPP nor its electron‐deficient analogue aza[10]CPP actively participate in charge separation. In contrast, the nanohoops completely prevented through‐space charge separation. This result is likely due to supramolecular “shielding”, because charge separation was observed in the thread that acted as reference dyad. On the other hand, the suppression of electron transfer allowed the observation of energy transfer from the porphyrin triplet to the fullerene triplet state with a lifetime of ca. 25 μs. The presence of the interlocked nanohoops therefore leads to a dramatic switch between charge separation and energy transfer. We suggest that our results explain observations made by others in photovoltaic devices comprising nanohoops and may pave the way toward strategic uses of mechanically interlocked architectures in devices that feature (triplet) energy transfer.

## Graphical abstract

The two different nanohoops in elaborate porphyrin/(aza)[10]CPP/fullerene [2]rotaxanes (CPP: cycloparaphenylene) have been shown to act as “supramolecular shields” and completely prevent through‐space electron transfer between the electron‐donating porphyrin and electron‐accepting fullerene. These findings shed light on some previously unexplained results and open the door for future strategic uses of strained carbon nanohoops.

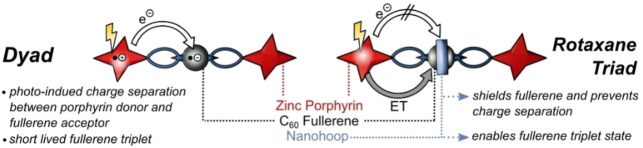

## Introduction

Soon after the first syntheses of cycloparaphenylenes (CPPs) by Jasti and Bertozzi,^[1]^ chemists started to explore the supramolecular potential of this new class of shape‐persistent macrocycles.[^2^, ^3^, ^4^] CPPs and structurally related carbon nanohoops were shown to encapsulate a variety of guests including fullerenes,[^5^, ^6^, ^7^, ^8^, ^9^, ^10^, ^11^, ^12^, ^13^, ^14^, ^15^, ^16^, ^17^, ^18^] smaller nanohoops,[^19^, ^20^, ^21^, ^22^, ^23^] aromatics,[^24^, ^25^, ^26^, ^27^] and even aliphatic molecules.[^25^, ^28^, ^29^] Thanks to the growing body of knowledge on CPP synthesis and supramolecular chemistry,[^14^, ^30^, ^31^, ^32^, ^33^] the first mechanically interlocked molecules (MIMs) constructed from carbon nanohoops were synthesized by passive^[34]^ and active metal template strategies.[^35^, ^36^, ^37^, ^38^, ^39^, ^40^, ^41^] Whereas those strategies necessitate the use of heteroatom containing macrocycles such as aza[*n*]CPPs, Itami and Cong developed covalent‐template strategies which allow the construction of all‐benzene catenanes and rotaxanes.[^42^, ^43^, ^44^, ^45^]

**Figure 1:**
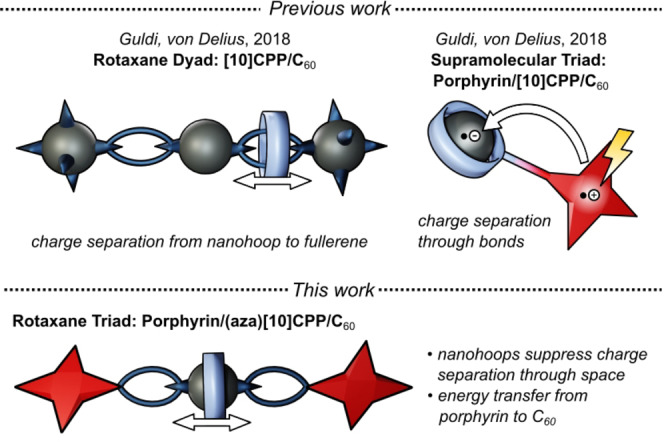
This work in context of previous studies from our laboratories.[^46^, ^71^]

A third approach does not require metal templates, heteroatoms or cleavable covalent bonds. In 2018, we showed that concave‐convex π–π templation allows the synthesis of [2]rotaxanes, in which a [10]CPP ring is mechanically interlocked with a fullerene bis‐adduct thread (Figure 1, top left).^[46]^ Transient‐absorption spectroscopy (TAS) revealed insights into the positional dynamics of the [10]CPP ring in this [2]rotaxane.

Owing to their unique photophysical‐ and optoelectronic properties,[^47^, ^48^, ^49^, ^50^, ^51^] carbon nanohoops are promising components in organic electronic devices.[^52^, ^53^, ^54^, ^55^, ^56^, ^57^, ^58^, ^59^, ^60^, ^61^, ^62^, ^63^, ^64^, ^65^, ^66^, ^67^] For instance, Tao, Du, and co‐workers showed that the addition of [9]CPP to fullerene electron acceptors led to an increase of up to 20 % of the power conversion efficiency (PCE) in bulk heterojunction solar cells.^[68]^ From a supramolecular perspective, this result is unexpected, because the [9]CPP macrocycle is size‐mismatched with both PCBM and PC_71_BM and does only allow partial encapsulation. Such unexplained observations highlight the need to answer the question how the binding of CPPs modulates the capability of fullerenes to undergo light‐induced processes such as charge separation and energy transfer. We propose that supramolecular and mechanically interlocked architectures are ideally suited for this purpose, as they can be used to bring photoactive components together in a modular, dynamic, and predictable way and therefore allow solution‐phase pump‐probe studies on the fs timescale.[^46^, ^69^, ^70^, ^71^] Experimental studies of mechanically interlocked molecules in solution can also be cross‐checked with molecular‐dynamics (MD) simulations, which are capable of resolving molecular motions at atomistic resolution.[^72^, ^73^]

We previously synthesized supramolecular porphyrin‐[10]CPP⊃fullerene triads and studied photo‐induced charge separation between a porphyrin electron‐donor and fullerene electron‐acceptors. We found that charge separation occurred readily in such architectures, presumably through the covalent bridge between porphyrin and nanohoop (Figure 1, top right).^[71]^ While this was a valuable demonstration of a functionalized carbon nanohoop acting as an “innocent” supramolecular mediator of *through‐bond* electron transfer, it left one important question unanswered that is highly relevant for photovoltaic devices: How does encapsulation of C_60_ in [10]CPP affect *through‐space* electron transfer? Herein we report the synthesis of two [2]rotaxanes that we specifically designed to address this aspect. We observed that the nanohoops [10]CPP and aza[10]CPP completely suppress charge separation in these mechanically interlocked architectures, which stands in stark contrast to our previous study.^[71]^ We suggest that the observed suppression is due to the fact that only *through‐space* electron transfer is possible in the rotaxane architecture.

## Results and Discussion

### Molecular Design and Synthesis of Precursors

The envisaged [2]rotaxanes feature a porphyrin stopper as electron donor, a central fullerene bis‐adduct as electron acceptor and a mechanically interlocked [10]CPP or the previously reported,^[74]^ “nitrogen‐doped” aza[10]CPP macrocycle (Figure 1, bottom). A “capping” strategy is used to covalently capture the ring on the thread between the two porphyrin stoppers that carry three aryl substituents in the *meso* positions and are thus large enough to prevent the large (aza)[10]CPP macrocycles from dissociating from the thread. Two consecutive Bingel addition reactions generate the fullerene bis‐adduct, which due to particularly strong concave‐convex π–π interactions leads to the mechanical interlocking of the nanohoops as well as some degree of regioselectivity (preferred formation of the *trans*‐2 and *trans*‐3 regioisomers).^[46]^ Because the nanohoop is not covalently attached to either the electron donor or the electron acceptor, this architecture is ideally suited to focus on how the binding of the nanohoop to the central fullerene modulates its capability to accept photo‐induced electrons (Figure 1). Even though the nanohoop is, in principle, free to translate along the thread, it is important to note that the fullerene binding site represents a deep thermodynamic minimum on the free energy landscape, causing, the nanohoop to reside there most of the time. By nitrogen‐doping of the [10]CPP scaffold, we simultaneously modulate two relevant characteristics of the nanohoop: (i) the binding affinity for C_60_ (to be ca. 4× higher in aza[10]CPP) and (ii) the LUMO level (to be ca. 0.1 eV lower for aza[10]CPP).^[74]^

**Scheme 1:**
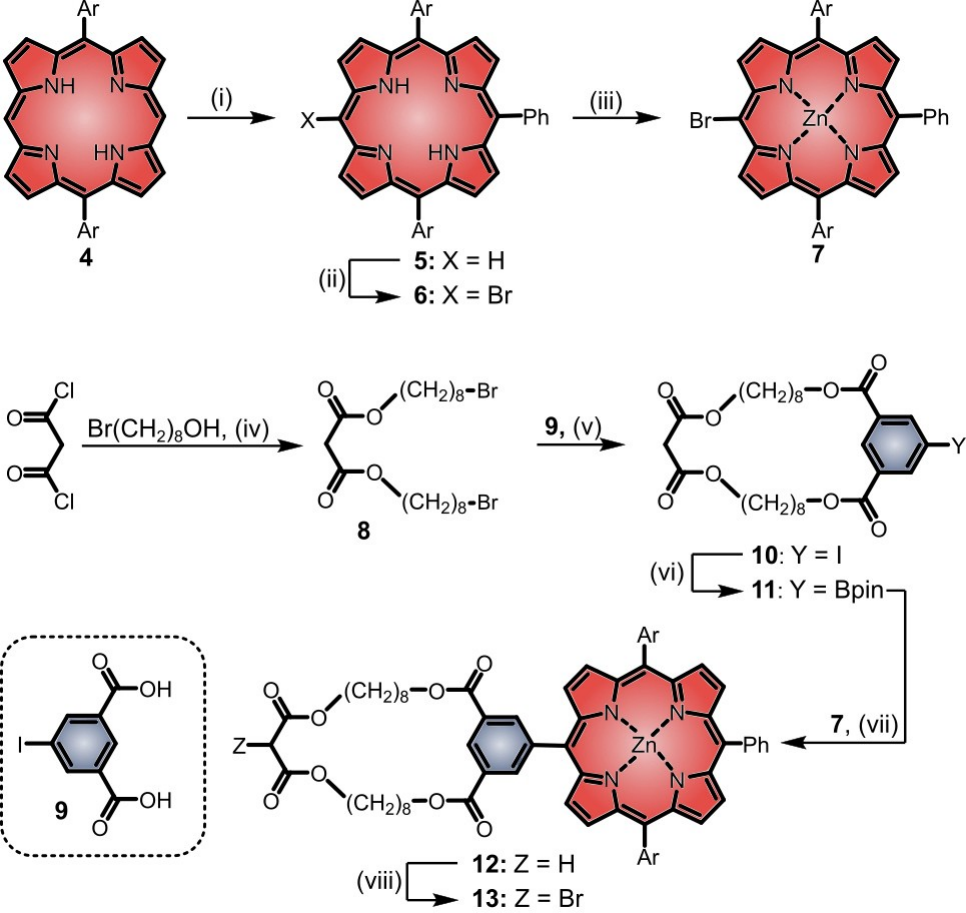
Synthesis of precursors. Reaction conditions and yields: (i) 1. PhLi, 2. H_2_O, 3. DDQ, THF, 0 °C, 77 %. (ii) NBS, pyridine, CHCl_3_, 97 %. (iii) Zn(OAc)_2_, DCM/MeOH, quant. (iv) DMAP, NEt_3_, DCM, 0 °C, 64 %. (v) KHCO_3_, DMF, 90 °C, 70 %. (vi) Bpin_2_, KOAc, Pd(OAc)_2_, DMF, 90 °C, 91 %. (vii) K_2_CO_3_, Pd(PPh_3_)_4_, PhMe/DMF, 90 °C, 88 %. (viii) CBr_4_, DBU, THF, −78 °C, 74 %. Ar=3,5‐di‐*tert*‐butyl‐phenyl.

We decided to use Senge's S_N_ method to obtain desymmetrized porphyrins as stoppers (Scheme 1).[^75^, ^76^, ^77^] Using phenyl lithium (PhLi) as nucleophile, followed by hydrolysis and subsequent oxidation with 2,3‐dichloro‐5,6‐dicyano‐1,4‐benzoquinone (DDQ) we were able to transform diarylporphyrin **4** into the desymmetrized triarylporphyrin **5** in a yield of 80 %. The following bromination and zinc insertion proceeded quantitatively and furnished porphyrin **7**. To synthesize a symmetric, macrocyclic linker that is a suitable starting material for the Bingel^[78]^ addition to C_60_, we combined malonyl chloride with 8‐bromo‐octanol to obtain U‐shaped **8**. Under high dilution, we treated **8** with the salt of 5‐iodoisophthalic acid **9** and were able to isolate the macrocycle **10** in a yield of 70 %. After transformation into the boronic ester **11**, Suzuki–Miyaura cross‐coupling (product: **12**)^[79]^ and selective mono‐bromination of the malonate with CBr_4_ assisted in obtaining key compound **13**.

### Rotaxane Synthesis and Regioisomers

**Figure 2:**
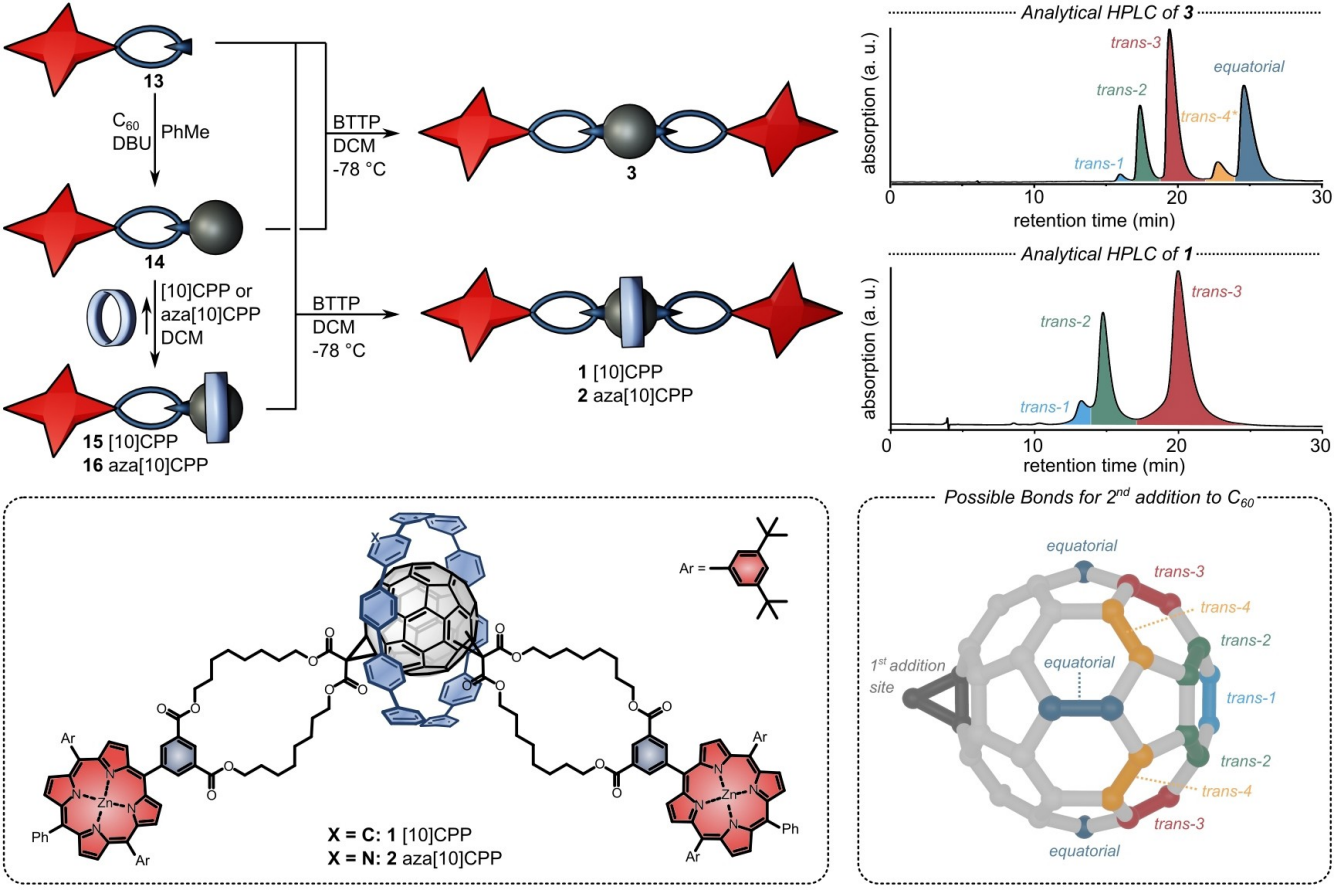
Synthesis of rotaxanes **1** and **2** as well as thread **3**. Details for the synthesis of the respective compounds: **14**: 2 equiv. C_60_, 3 equiv. DBU, toluene, rt, 72 %. **15/16**: 1 equiv. (aza)[10]CPP, DCM, rt, quant. **3**: 1 equiv. **13**, 2 equiv. BTTP, −78  °C, 79 %. **1**: 2 equiv. **13**, 3 equiv. BTTP, −78  °C, 24 %. **2**: 2 equiv. **13**, 3 equiv. BTTP, −78  °C, 20 %. BTTP=*tert*‐butylimino‐tri(pyrrolidino)phosphorane). Analytical HPLC traces of **3** (Buckyprep, ethyl acetate, 430 nm, 0.5 mL/min) and **1** (silica gel, PhMe/n‐Hexane 9 : 1, 430 nm, 0.5 mL/min) after initial purification showing different fullerene regioisomers (*tentative assignment). Molecular structure of the rotaxanes **1** and **2**. Possible bonds for second addition to C_60_ and respective nomenclature of selected regioisomers.

With the “half‐stopper” in hands, we proceeded with the rotaxane synthesis by treating **13** with an excess of C_60_ in a first Bingel reaction to form the fullerene mono‐adduct **14** (Figure 2). By simple addition of stoichiometric amounts of [10]CPP or aza[10]CPP in dichloromethane we obtained pseudorotaxanes **15** and **16**. To ensure that the nanohoops are bound throughout the second Bingel addition and to minimize unwanted side reactions, the following capping step was performed at −78  °C. We only obtained reasonable yields of the rotaxanes when using the phosphazene base BTTP ((*tert*‐butylimino)tris(pyrrolidino)phosphorane) rather than DBU (1,8‐diazabicyclo(5.4.0)undec‐7‐ene) or sodium hydride. To prepare “thread” **3** as a reference for the photophysical studies, we performed the same reaction in the absence of any nanohoop. Comparison of the HPLC traces after both reactions (Figure 2, right) corroborates the template effect of the nanohoop on regioselectivity, which we had described in our previous rotaxane synthesis.^[46]^ While we observed six out of eight possible isomers in the absence of the nanohoop,^[80]^ only three different isomers were observed in the nanohoop‐templated reaction. The “nitrogen‐doped” nanohoop, which had not yet been studied previously as a template, led to the same regioisomeric outcome as the parent nanohoop [10]CPP. Initial purification by column chromatography afforded the rotaxanes in yields of 24 % (**1**), 20 % (**2**) as a mixture of *trans*‐1, *trans*‐2, and *trans*‐3 regioisomers. To separate the isomers by preparative HPLC, we investigated various stationary and mobile phases and achieved the isolation of the *trans*‐2 and *trans*‐3 regioisomers of [10]CPP rotaxane **1** by using a 9 : 1 mixture of toluene and *n*‐hexane on a normal phase HPLC column. Even though aza[10]CPP rotaxane **2** only differs in one out of 308 non‐hydrogen atoms from [10]CPP rotaxane **1**, we were unable to separate the three isomers of **2** using the same or any modified conditions. So we studied the properties of this compound as a mixture of isomers. Using a Buckyprep HPLC column with ethyl acetate as mobile phase, we were able to isolate three isomers of thread **3** on a semi‐preparative scale. The presence of the nanohoop(s) renders these regioisomer separations significantly more difficult. The rings essentially shield those parts of the molecules from the stationary phase, where the structural differences between isomers are most pronounced.

**Figure 3:**
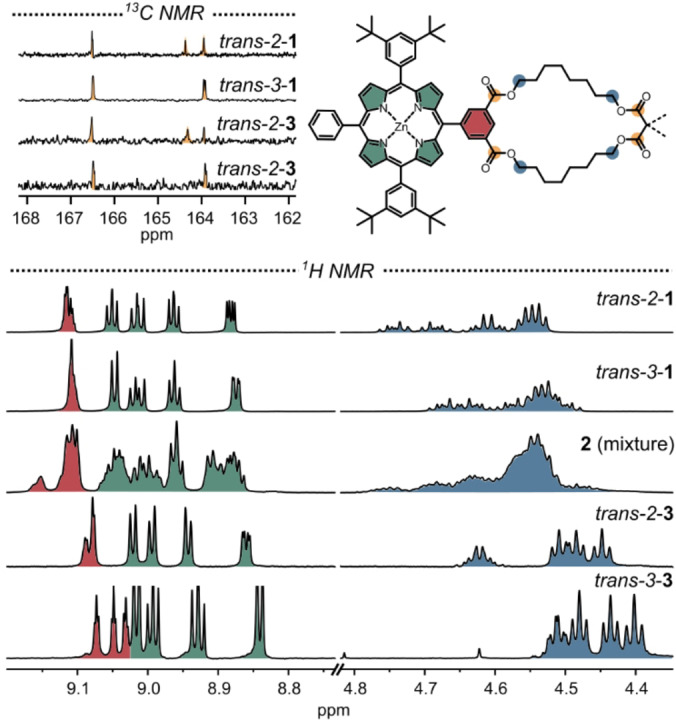
Selected regions of the ^1^H and ^13^C NMR spectra (CD_2_Cl_2_, 600 MHz) of rotaxanes **1** and **2** and thread **3**. For full spectra and assignments see the Supporting Information.

Our assignment of the isolated products to specific regioisomers was informed by earlier work from our lab^[46]^ and further corroborated by the C=O region in the ^13^C NMR spectra (Figure 3). This allowed comparisons with published data on simpler dimethyl malonate fullerene bis‐adducts.^[73]^ Because the “gold standard” technique to assign fullerene regioisomers—analysis of the fingerprint region in the UV/Vis spectra—was hampered by the overlapping porphyrin Soret‐band absorption, we decided to perform transesterification experiments with MeOH as introduced by Diederich.^[81]^ By converting all malonates into simple methyl esters, we could clearly identify the characteristic ^1^H NMR shifts of the *trans*‐2 and *trans*‐3‐dimethyl malonate fullerene bis‐adducts (Figures S2 and S3).

Comparison of the ^1^H NMR spectra of the *trans‐2* and *trans‐3* isomers of [10]CPP rotaxane **1** with thread **3** (Figure 3) reveals major differences. On one hand, peak splitting happens. On the other hand, chemical shifts of the protons closest to the fullerene, which are the C*H*_2_ protons next to the malonate (blue in Figure 3), and the protons of the isophthalate (red in Figure 3). Moreover, when compared to thread **3**, most protons in rotaxanes **1** and **2** are shifted downfield. They therefore experience a deshielding effect due to the presence of the CPP rings (e.g. due to the presence of the CPP protons).

### Evidence for [2]Rotaxane Structure

**Figure 4:**
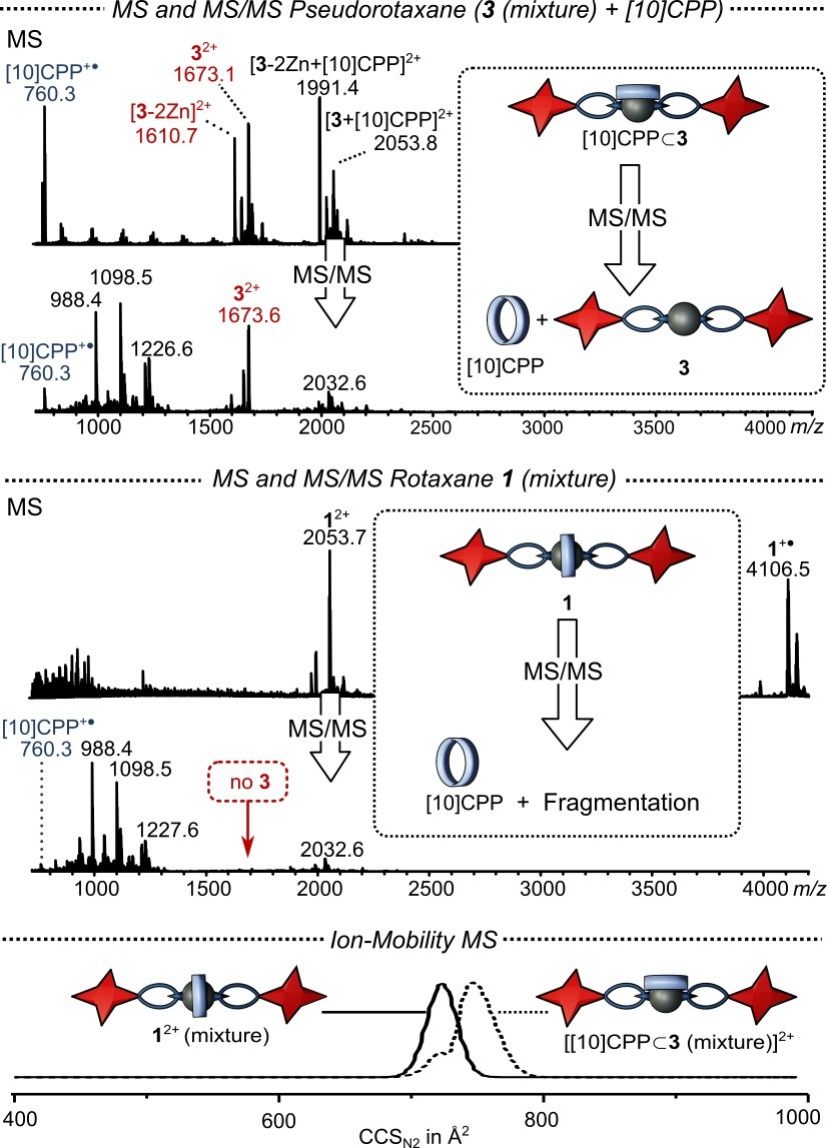
Top: ESI‐MS spectra of the pseudorotaxane (prepared by mixing **3** and [10]CPP) and corresponding MS/MS spectrum after mass‐selection of [**3**+[10]CPP]^2+^ and collision with an acceleration voltage of 165 V. Middle: ESI‐MS spectra of rotaxane **1** and corresponding MS/MS spectrum after mass‐selection of **1** ^2+^ and collision with an acceleration voltage of 170 V. Bottom: CCS distribution in N_2_ of the [**3**+[10]CPP]^2+^ and the rotaxane **1^2+^**.

To provide evidence for the mechanically interlocked structures of rotaxanes **1** and **2**, we performed mass spectrometry (MS) studies, including both tandem (MS/MS) and ion mobility mass spectrometry (IM–MS).[^82^, ^83^] As a reference, we employed a non‐interlocked pseudorotaxane, by simply mixing thread **3** (as isomeric mixture) with the respective nanohoops ([10]CPP or aza[10]CPP). These reference complexes are stable enough to be observed as their dications in the ESI^+^ mass spectrum (Figure 4, top). MS/MS via collision‐induced dissociation revealed that the weak supramolecular bond in the pseudorotaxane is easily broken and both the nanohoop ([10]CPP^+^⋅) and the thread (**3^2+^**) are found in the respective spectrum. In contrast, the MS/MS spectrum of rotaxane **1** does not reveal the thread (**3^2+^**), but only fragments derived from it (Figure 4, middle). This result indicates that the nanohoop is mechanically interlocked, as the thread needs to be broken to release the nanohoop.

We also used IM–MS to distinguish between the pseudorotaxane and rotaxane (Figure 4, bottom).[^84^, ^85^] By measuring the time that the ions need to traverse a gas‐filled cell under the influence of an electric field, structural information was obtained from converting this time to a collisional cross section (CCS). **1** ^2+^ presents as a clean distribution centered around CCS_N2_=724 Å^2^ (CCS measured in nitrogen gas), whereas [**3**+[10]CPP]^2+^ has its maximum at CCS_N2_=745 Å^2^, reflecting the larger size of pseudorotaxanes compared to rotaxanes (Figure 4, bottom). The distribution of [**3**+[10]CPP]^2+^ also includes a shoulder which may be a result of the isomeric mixture used. This assumption is supported by the fact, that we were able to distinguish between several rotaxane isomers after passing **2** ^2+^ repeatedly through a cyclic ion‐mobility cell (Figure S10).^[86]^

### Photophysical Characterization

**Figure 5:**
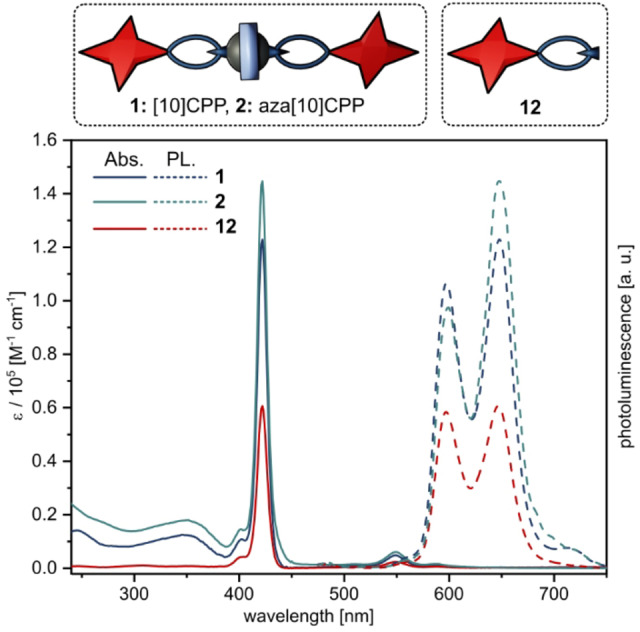
UV/Vis absorption (solid‐line) and fluorescence spectra of **1** (*trans‐3*‐isomer), **2** (mixture of isomers) and **12** recorded in DCM.

The UV/Vis absorption and fluorescence spectra of the rotaxanes **1** and **2** (Figure 5, blue) follow in general the characteristics of porphyrins (Figure 5, red) with the intensive Soret‐band absorption at around 420 nm, the Q‐band absorption at around 550 nm and two emission bands at around 600 and 650 nm. Additionally, the absorption of [10]CPP or aza[10]CPP appear at around 350 nm, whereas the emission of the nanohoops, expected between 470 and 480 nm, is fully quenched. To study the deactivation pathways after photoexcitation, we turned to time‐resolved transient absorption spectroscopy (TAS) on femto‐ and nanosecond timescales. Of particular interest is the role of the [10]CPP and aza[10]CPP rings and their influence on the electron‐donating porphyrins and electron‐accepting fullerene C_60_. Therefore, we used a wavelength of 430 nm to photoexcite into the Soret‐band absorption of the porphyrins.

We focussed on the *trans*‐3 regioisomer of [10]CPP‐rotaxane **1**, while for aza[10]CPP‐rotaxane **2** and the thread **3**, a mixture of isomers was examined. Tetrahydrofuran (THF) and benzonitrile (PhCN) were used, as we had already investigated the electron transfer between [10]CPP as well as aza[10]CPP with C_60_ in these solvents in a previous study.^[74]^

**Figure 6:**
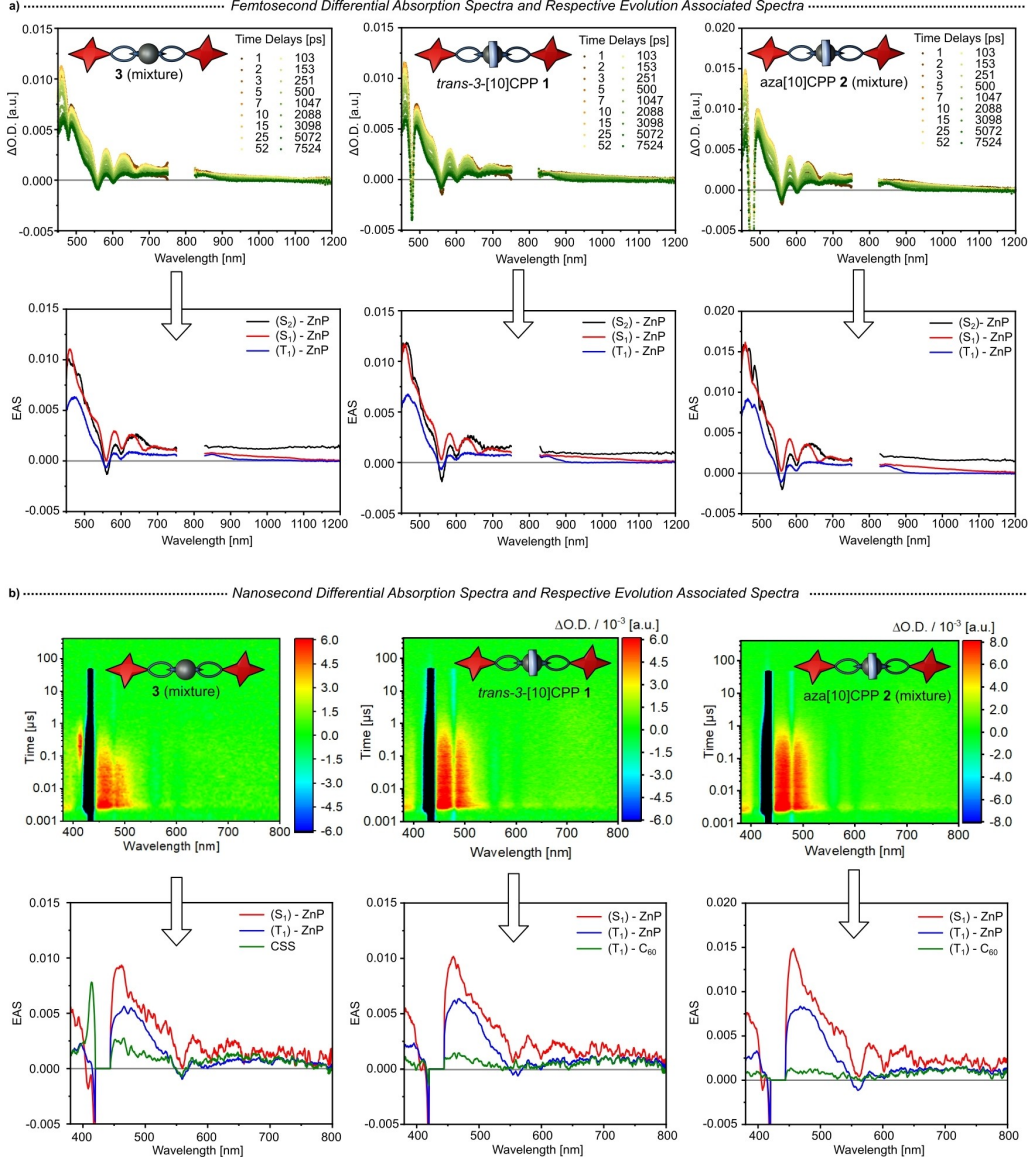
**a)** Femtosecond differential absorption spectra in argon purged PhCN at time delays between 1 and 7550 ps after 430 nm laser excitation of thread **3** (isomeric mixture), [10]CPP‐rotaxane **1** (*trans‐3*‐isomer), and aza[10]CPP‐rotaxane **2** (isomeric mixture). Below are the corresponding deconvoluted EAS obtained via global analysis following a sequential deactivation model (black‐red‐blue). Scattering at 480 nm was corrected by subtracting the background**. b)** Nanosecond differential absorption spectra in argon purged PhCN at time delays between 0.001 and 440 μs after 430 nm laser excitation of thread **3**, [10]CPP‐rotaxane **1**, and aza[10]CPP‐rotaxane **2**. Below are the corresponding deconvoluted EAS obtained via global analysis following a sequential deactivation model (red‐blue‐green).

To determine the influence of the nanohoops on the deactivation of the rotaxanes and differentiate between [10]CPP and aza[10]CPP, we first studied porphyrin‐C_60_ thread **3** lacking any macrocycle as reference. Deconvolution via global analysis necessitates four total species to fit the data over the entire fs‐ and ns‐timescales and a sequential model was used to generate the evolution‐associated spectra (EAS). The first species, seen in Figure 6a (left), shows a lifetime of 1.1 ps and is assigned to the second singlet excited state (S_2_) of the porphyrin. It has its main feature in the form of excited state absorption (ESA) at 465 nm and shows typical porphyrin ground state bleaching (GSB) at around 560 and 600 nm. Following its decay, the second species evolves in the fs‐timeframe with a lifetime of 0.7 ns. Noticeable is an additional minimum at 660 nm, which stems from stimulated emission (SE) of the porphyrin. The main ESA maximum shifts to 460 nm and part from this, it reveals only subtle differences compared to the first one and is therefore assigned to a porphyrin‐centered first singlet excited state (S_1_). No rise of any additional feature in the 1000 to 1100 nm region is observed in this timeframe, which has been established as the most reliable way to monitor the reduction of C_60_ in many similar studies.[^87^, ^88^, ^89^, ^90^, ^91^] The lifetime of the following third species exceeds the detection range of our fs‐TAS setup and is evaluated via ns‐TAS (Figure 6b, left). It has a lifetime of 0.1 μs and its main ESA is broadened and shifts to 470 nm. GSBs remain at 560 and 600 nm but the fluorescence related SE at 660 nm disappeared. Additionally, it shows an ESA at 850 nm, which is a spectroscopic fingerprint of the triplet excited state (T_1_) of zinc‐tetraphenylporphyrins.[^92^, ^93^] In turn, we assign it to the porphyrin (T_1_). Overall, up to this point the deactivation pathway is mainly centered on the porphyrin.^[94]^ Its decay gives rise to the fourth species.^[95]^ In THF and PhCN, the 470 nm ESA is replaced by a weak ESA that spans from 600 to 760 nm. Only in PhCN, the 415 nm feature is discernible, which correlates with the signature of the one‐electron oxidized form of the porphyrin. From this finding we conclude that a charge‐separated state (CSS), which includes porphyrin oxidation and CPP⊃C_60_ reduction, is formed.[^96^, ^97^] Its lifetime is 0.7 μs in PhCN.

In the next step, we probed [10]CPP‐rotaxane **1** (*trans*‐3 isomer) and aza[10]CPP‐rotaxane **2** (mixture of isomers). At first glance, their deactivation follows the same pathway summarized above for thread (**3**). A closer look reveals, however, subtle differences. Starting directly after the photo‐excitation, the formation of porphyrin (S_2_) is seen with a 465 nm ESA and GSB minima at 560 and 600 nm (Figure 6a). Lifetimes for both [10]CPP‐rotaxane and aza[10]CPP‐rotaxane are similar in THF as well as PhCN and are in the range of 0.9 to 1.8 ps. Following its decay, the rise of the second species is accompanied by a blue‐shift of the ESA to 460 nm and SE at 660 nm. This species is again assigned to (S_1_) porphyrin and the lifetimes are in the range of 0.9 to 1.0 ns. Next in line is the third species, for which a red‐shift of the ESA to 470 nm goes together with a new 850 nm ESA. This and the fact that no 660 nm SE is detected underlines its (T_1_) character.

We could not gather any evidence for any electron transfer for either of the two [2]rotaxanes, which stands in contrast to the results observed for thread **3**. Non‐discernible is the one‐electron reduced form of C_60_ for **1** or **2**. Only the porphyrin triplet state (T_1_) was seen in ns‐TAS measurements and its lifetime, which ranges from 0.7 to 1.0 μs, is independent on solvent and nanohoop (Figure 6b). Compared to the 0.1 μs lifetime determined for thread **3**, this result reflects an extended lifetime due to the introduction of CPPs. When considering THF, the porphyrin triplet (T_1_) directly returns to the ground state. Notably, measurements in PhCN give a different picture. Adding a fourth species is necessary to fit the data taken for [10]CPP‐rotaxane (**1**) and aza[10]CPP‐rotaxane (**2**). Characteristics of the fourth species are 470 and 720 nm ESAs. However, the 415 nm ESA, which we noted for the thread **3**, is absent. Upon spectral comparison, we assign the fourth species to (T_1_) of C_60_.[^46^, ^74^, ^98^, ^99^] (T_1_) lifetimes are 25 and 24 μs for [10]CPP‐rotaxane and aza[10]CPP‐rotaxane, respectively. This and the missing clear evidence for any charge separation led us to conclude that energy transfer from the porphyrin (T_1_) is responsible to form the long‐lived (T_1_) of C_60_.

In summary, all rotaxanes give rise to a quasi‐identical deactivation behavior for the first steps after photo‐excitation of the porphyrin at 430 nm. First, (S_2_) of the porphyrin is formed with a lifetime of 0.9 to 1.8 ps. Subsequently, it transitions into (S_1_) with a lifetime of 0.7 to 1.0 ns. Following its decay, (T_1_) is formed, which is still centered at the porphyrin. Here, the thread (**3**) displays a shorter lifetime of 0.1 μs compared to rotaxanes **1** and **2** with lifetimes of around 0.7 to 1.0 μs. At this point, thread and mechanically interlocked compounds start to differ significantly. On one hand, the thread exhibits the formation of a charge separated state, which decays with a lifetime of 0.7 μs in PhCN. On the other hand, for rotaxanes **1** and **2**, the presence of the CPP rings masks the electron transfer. Instead, energy transfer occurs from the porphyrin (T_1_) to C_60_, resulting in its (T_1_) with a lifetime around 24 to 25 μs in PhCN. Overall, no particular influence specifically linked to the different CPP rings, that is [10]CPP vs. aza[10]CPP, was noted. A summary of the formed species in PhCN and the corresponding lifetimes after photoexcitation is provided in Table 1.

**Table 1:** Comparison of the different lifetimes and the assigned species from time‐dependent photophysical studies in PhCN and illustration of the most important findings.

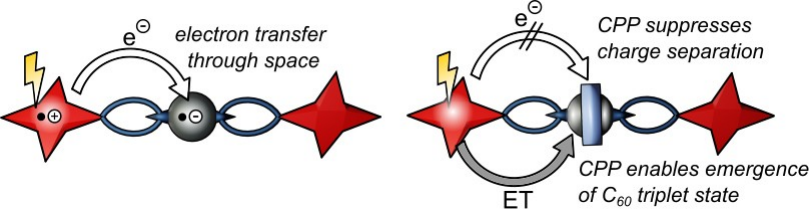		
lifetime of *assigned species*		
thread **3**	[10]CPP rotaxane **1**	aza[10]CPP rotaxane **2**
1.1 ps (*S_2_)‐ZnP*	1.0 ps (*S_2_)‐ZnP*	0.9 ps (*S_2_)‐ZnP*
0.7 ns (*S_1_)‐ZnP*	0.9 ns *(S_1_)‐ZnP*	1.0 ns (*S_1_)‐ZnP*
0.1 μs (*T_1_)‐ZnP*	0.7 μs *(T_1_)‐ZnP*	0.7 μs *(T_1_)‐ZnP*
0.7 μs *(ZnP)* ^*+*^⋅	*no charge separation observed*	
(*T_1_)‐C_60_* not obs.	25 μs (*T_1_)‐C_60_*	24 μs *(T_1_)‐C_60_*

Possible CPP shuttling was examined via temperature‐dependent TAS measurements (Table S2 and Figures S38–41). Despite heating up to 80 °C, no significant changes are noted in the deactivation pathway after photo‐excitation. Triplet lifetimes of **1** and **2** are slightly shortened without giving rise to any electron transfer. **3** still exhibits a CSS with a lifetime of 0.7 μs. We conclude that a possible shuttling plays a minor role, if any at all. A NOESY NMR experiment is in agreement with this finding (Figure S70).

To compare the rotaxanes with a simple mixture of a porphyrin, a comparable fullerene derivative, and [10]CPP, we performed TAS in PhCN at different molar ratios of the three components (Table S1 and Figures S23–S36). First, porphyrin **12** was measured and it displays a slightly longer (S_1_) lifetime of 2.2 ns. Furthermore, its (T_1_) lifetime with >400 μs exceeds the time window of our measurement setup and is orders of magnitude longer compared to what we see for the rotaxanes. When adding a *trans*‐3 diethyl malonate C_60_ bis‐adduct in a 1 : 1 molar ratio, the (T_1_) lifetime is reduced to 92 μs and a CSS evolves. Spectroscopic evidence for the CSS includes the 415 nm fingerprint of the one‐electron oxidized form of the porphyrin. Adding [10]CPP or aza[10]CPP at an overall 1 : 1 : 1 molar ratio of the components leads to further reduction of the (T_1_) lifetime to 56 μs. A CSS is still formed. Changing the molar ratios only effects the quenching of (T_1_) and forming of CSS, but the lifetime of (S_1_) remains constant at 2.2 ns regardless of the respective ratio.

In contrast to the rotaxanes, the CSS is observed in every mixture. We propose that this result is due to the predominant (ca. 95 % according to BindSim, Figure S42) presence of the non‐complexed fullerene under the (necessarily) dilute conditions of TAS measurements. The three component experiment therefore highlights the great utility of rotaxane architectures for transient absorption studies: the mechanical interlocking of CPP ring and fullerene bis‐adduct makes the complete dissociation of the two components impossible and therefore is a way to bypass the mass action law in solution.

## Conclusion

We synthesized structurally complex [2]rotaxanes featuring a central fullerene⊂nanohoop complex and porphyrin stoppers at the periphery. In the rotaxane forming step, the nanohoop acts as a supramolecular template^[46]^ that limits the number of regioisomeric fullerene bis‐adducts. HPLC analysis of the regioisomers and synthesis of the non‐interlocked thread as reference allowed us to evaluate the effect of [10]CPP and aza[10]CPP on the rotaxane forming reaction. Interestingly, despite the significantly higher fullerene binding affinity of aza[10]CPP (vs. [10]CPP),^[74]^ we observed nearly identical yields and isomer distributions.

In ultrafast transient absorption spectroscopy studies, we sought to shed light on the photo‐induced electron transfer processes of the three different electron donor‐acceptor architectures **1**, **2** and **3**. In thread **3**, we observed a charge‐separated state as result of through‐space electron transfer. In contrast, the different nanohoops in rotaxanes **1** and **2** seem to act as “supramolecular shields” and completely prevent through‐space electron transfer between the electron‐donating porphyrin and electron‐accepting fullerene acceptor. This finding stands in contrast to our previously studied porphyrin‐[10]CPP⊃fullerene supramolecular architecture (Figure 1).^[71]^ Evidently, nanohoops are capable of facilitating though‐bond electron transfer between porphyrins and encapsulated C_60_, but they will prevent through‐space electron transfer to encapsulated C_60_ in a mechanically interlocked architecture and enable the emergence of a fullerene‐centered triplet state instead.

These results corroborate the capability of shape‐persistent [10]cycloparaphenylene nanohoops to significantly modulate the photophysical features of fullerenes encapsulated in either supramolecular or mechanically interlocked architectures. The suppression of charge separation to fullerenes encapsulated in [10]CPP may explain a counter‐intuitive result by Tao and Du, namely that the size‐mismatched combination of [9]CPP with the fullerene acceptor PCBM was particularly effective in an organic solar cell.^[68]^

## Conflict of Interests

The authors declare no conflict of interest.

1

## Supporting information

As a service to our authors and readers, this journal provides supporting information supplied by the authors. Such materials are peer reviewed and may be re‐organized for online delivery, but are not copy‐edited or typeset. Technical support issues arising from supporting information (other than missing files) should be addressed to the authors.

Supporting Information

## Data Availability

The data that support the findings of this study are available in the supplementary material of this article.
