# Supplementary material for: Nanohoops Favour Light‐Induced Energy Transfer over Charge Separation in Porphyrin/[10]CPP/Fullerene Rotaxanes
Source: Angew Chem Int Ed Engl. 2024 Nov 11;64(1):e202413404. doi: 10.1002/anie.202413404 (PMC11701370; doi:10.1002/anie.202413404)
Supplement: Supplementary file 1 — Supporting Information [file ANIE-64-e202413404-s001.pdf]

Angewandte Chemie
Eine Zeitschrift der Gesellschaft Deutscher Chemiker

Supporting Information

Nanohoops Favour Light-Induced Energy Transfer over Charge Separation in Porphyrin/[10]CPP/Fullerene Rotaxanes

F. Schwer, S. Zank, M. Freiberger, F. M. Steudel, N. Geue, L. Ye, P. E. Barran, T. Drewello, D. M. Guldi, M. von Delius**

Nanohoops Favour Light-Induced Energy Transfer over Charge Separation in Porphyrin/[10]CPP/Fullerene Rotaxanes

Fabian Schwer,[a] Simon Zank,[b] Markus Freiberger,[b] Fabian M. Steudel,[a] Niklas Geue,[c] Lei Ye,[b] Perdita E. Barran,[c] Thomas Drewello,[b] Dirk M. Guldi*[b] and Max von Delius*[a]

Supporting Information

Table of Contents

1. General Experimental Section ... 3
2. Synthesis ... 6
4. Transesterification Experiments ... 26
5. Additional Data on Rotaxane Synthesis and Purification ... 29
6. MS Experiments ... 30
7. Transient Absorption Spectroscopy ... 33
8. Spectra ... 67
9. References ... 105

1. General Experimental Section

Reagents

All commercially available chemicals were purchased from Sigma Aldrich, TCI Germany, VWR International, Fischer Scientific, Carl Roth GmbH & Co., Acros Organics, ABCR and Alfa Aesar. All of them were used without further purification. Anhydrous solvents were dried prior to use on an MBraun SPS-800 system.

Aza[10]CPP was synthesized as previously reported.[1]

NMR

NMR spectra were recorded on BrukerAvance 400 or 600 NEO spectrometers (^{1}H: 400 or 600 Hz, ^{13}C: 101 Hz) at room temperature (298 K) if not noted otherwise. Chemical shifts (δ) are reported in ppm using residual solvent protons (^{1}H NMR: δ_H = 7.26 ppm for $CDCl_3$; ^{13}C NMR: δ_C = 77.16 ppm for $CDCl_3$) as internal standard. The splitting patterns are designated as follows: s (singlet), d (doublet), t (triplet), and m (multiplet). Coupling constants J relate to proton-proton couplings.

Tandem and Ion Mobility Mass Spectrometry

High resolution MALDI mass spectra were recorded on a Bruker SolariX ((Hybrid 7T FT-ICR) or on a Bruker UltrafleXtreme (MALDI-TOF/TOF) using *trans*-2-[3-(4-*tert*-butylphenyl)-2-methyl-2-propenylidene]malononitrile (DCTB) as matrix. High resolution ESI mass spectra were recorded on Bruker SolariX (Hybrid 7T FT-ICR).

<u>MS-MS</u>

Sample preparation: Stock solutions of the samples were prepared in DCM at concentrations of 0.3 g L^{-1} and 0.2 g L^{-1}, respectively. Electrospray ionization (ESI) experiments were performed using a solvent mixture of ACN/DCM/Tol (3:2:1, v:v:v) and adding 2 µL of TFA. The analyte concentrations were maintained at 1 x 10^{-5} mol L-1.

Mass spectrometry experiments were performed on an electrospray ionization quadrupole time-of-flight (ESI-Qq-ToF) mass spectrometer (microToF-Q II, Bruker Daltonics, Bremen). Analyte solutions were directly injected with a syringe pump at a flow rate of 180 µL h^{-1}. The temperature of the nitrogen counter flow was set to 180 °C and a capillary voltage of -4.5 kV was applied. The instrumental parameters were optimized to obtain optimal intensities for respective experiments. For MS^2 experiments, precursor ions were mass-selected by the quadrupole mass analyzer and accelerated into the collision cell quadrupole. N_2 was employed as the collision gas which was generated by a Parker LCMS nitrogen generator with a purity of 99.999% at a flow rate of 0.4 L min^{-1}.

IM-MS

Samples were ionized and transferred to the gas phase with an nESI source and were sprayed from borosilicate glass capillaries (World Precision Instruments). The latter were pulled on the Flaming/Brown P-2000 laser puller (Sutter Instrument Company). The capillary voltage (typically 1.0–1.5 kV) was applied through a platinum wire (diameter 0.125 mm, Goodfellow) inserted into the nESI capillaries. Source temperatures of 50 °C were applied.

IM–MS experiments were performed on a Synapt G2 and a prototype of the Select Series Cyclic IMS (both Waters Corp). Following ionization (cone voltage, 20 – 100 V; purge gas flow: 0 – 500 L h^{-1}), ions were transferred to the trap and further injected to the travelling wave drift cell (Synapt) or the cyclic ion mobility drift ring (Cyclic IMS). They were separated by using a non-uniform electric field under a constant nitrogen gas flow (Synapt: 75 mL min^{-1}; Cyclic: 30 mL min^{-1}) with travelling waves at different wave heights (Synapt: 40 V; Cyclic: 25 V) and velocity (Synapt: 671 m s^{-1}, Cyclic: 255 m s^{-1}), pushing the ions through the drift cell or ring, respectively. Lastly, they were transferred (transfer voltage: 2–4 V) to a time-of-flight mass analyser. For the experiments on the Cyclic IMS, ions were subjected to separation of up to 8 passes (separation time: 2–142 ms) and

Experimentally obtained arrival times and their distributions were converted to collisional cross-sections and their distributions *via* published calibration procedures.[2] The Agilent tune mix was used as a calibrant.[3]

Column and Thin Layer Chromatography

Normal-phase flash column chromatography was performed using silica 60 with a particle size of 0.04 – 0.063 mm from Macherey-Nagel. Thin layer chromatography was performed using TLC sheets with a particle size of 60 μm.

UV-vis Spectroscopy

UV-vis spectra were recorded using a Fisher NanoDrop OneC or on a Perkin Elmer UV/Vis Lambda 365 spectrophotometer with a quartz cuvette (10×10 mm).

Fluorescence Spectroscopy

Fluorescence spectra were recorded using a Perkin Elmer FL 6500 with a quartz cuvette (10×10 mm) .

HPLC

Analytical and Semipreparative HPLC was performed on a Shimadzu system with a Cosmosil buckyprep or a Ascentis silica column. Preparative HPLC was performed on a Büchi Flash/Prep HPLC System C-850 using a Ascentis silica column.

Transient Absorption Spectroscopy

Pump-probe transient absorption spectroscopy was performed using an Astrella-F-1K Ti:Sapphire amplifier from Coherent as a laser source with a central wavelength at 800 nm, repetition rate of 1 kHz, 5.0 W power output, 80 fs pulse duration, and 5 mJ pulse energy. Further, a fraction of 1.2 mJ was used for pump beam generation via the Topas Prime from Light Conversion with the standard NirUVis extension module. Femtosecond and nanosecond transient absorption studies were conducted with the HELIOS (0 to 7525 ps) and EOS (1 ns to 440 μs) transient absorption spectrometers from Ultrafast Systems. The white light probe pulse for the femtosecond measurements was generated by focusing a part of the 800 nm fundamental onto two sapphire disks after guiding it through a delay line. In the case of nanosecond experiments, a supercontinuum laser source with a fundamental at 1064 nm, 2 kHz repetition rate and pulse duration of 1 ns provided the white light probe pulse. For the measurements the energy per pump pulse was tuned to 400 nJ. All samples were prepared at room temperature in 2 x 10 mm quartz cuvettes, purged with argon for 20 min and stirred during the measurements.

Data evaluation was carried out using the Glotaran software with the R package TIMP.[4]

2. Synthesis

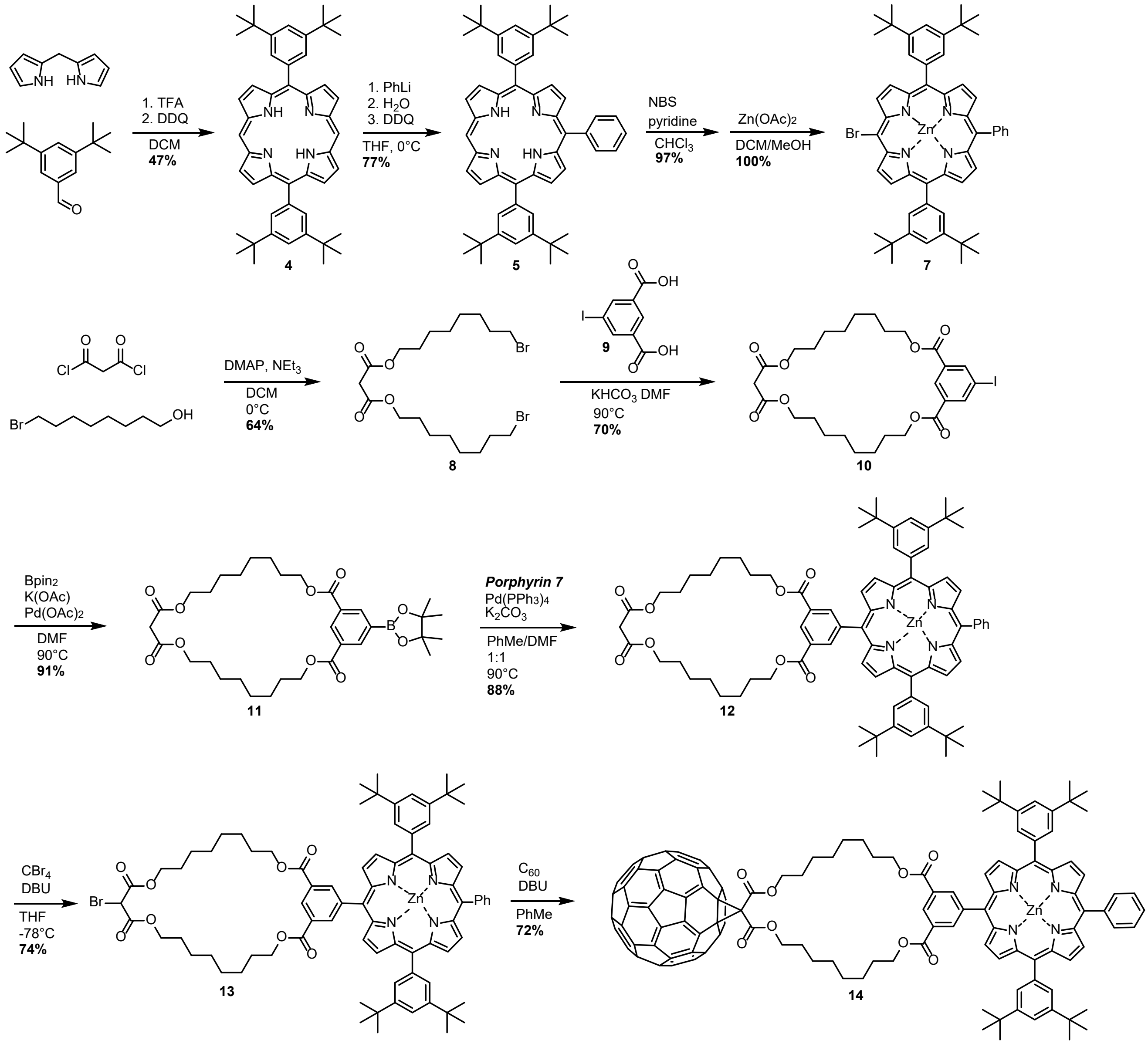

Scheme S1: Overview on the precursor synthesis

Synthesis of di(1*H*-pyrrol-2-yl)methane

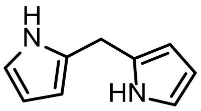

Synthesized according to a literature procedure.[5] Pyrrole (17.0 mL, 245 mmol, 4.1 equiv.) was dissolved in water (60 mL) and the solution was degassed via ultrasonication for 30 min and then cooled down to 0 °C. Formaldehyde (4.5 mL, 37% in water, 60 mmol, 1.0 equiv.) and HCl (0.2 mL, 35%, 2 mmol, 0.03 equiv.) were added dropwise over a period of 15 min and the reaction was stirred for further 2h at 0 °C. The reaction solution was extracted three times with DCM and the combined organic layers were concentrated under reduced pressure. The crude product was purified by distillation (110-120 °C at 0.03 mbar) to yield di(1*H*-pyrrol-2-yl)methane di(1*H*-pyrrol-2-yl)methane as colourless solid (3.22 g, 22 mmol, 37%). Since it is prone to slight decomposition at room temperature,[6] di(1*H*-pyrrol-2-yl)methane was stored in the freezer.

^{1}H NMR (400 MHz, $CDCl_3$): δ = 7.93 (bs, 2H, N*H*), 6.71 – 6.62 (m, 2H, α-Py-*H*), 6.15 (m, 2H, ß-Py-*H*), 6.04 (s, 2H, ß-Py-*H*), 4.00 (s, 2H, CH_2) ppm.

See appendix for spectrum. The measured data is in agreement with the literature.[5]

Synthesis of 4

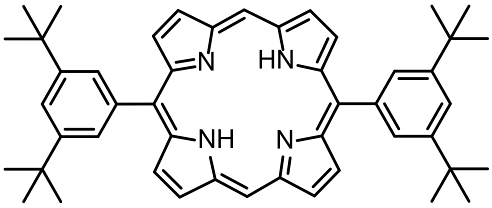

Synthesized according to a literature procedure.[7] 3,5-di-*tert*-butylbenzaldehyde (2.24 g, 10.3 mmol, 1.0 equiv.) and di(1*H*-pyrrol-2-yl)methane (1.50 g, 10.3 mmol, 1.0 equiv.) were dissolved in DCM (1.50 L) and the solution was degassed via ultrasonication for 20 min. TFA (0.30 mL, 3.9 mmol, 0.38 equiv.) was added dropwise and the solution was stirred for 2 h in the dark. DDQ (2.95 g, 13.0 mmol, 1.25 equiv.) was then added and the reaction was stirred for a further hour. After TLC indicated only one product, the reaction was quenched with triethylamine and the solvent was removed under reduced pressure. The crude product was purified by column chromatography (DCM/petroleum ether 1:2) to yield porphyrin **4** as red powder (1.67 g, 2.4 mmol, 47%).

^{1}H NMR (400 MHz, $CDCl_3$): δ = 10.31 (s, 2H, *meso-H*), 9.40 (d, *J* = 4.6 Hz, 4H, Py-*H*), 9.14 (d, *J* = 4.6 Hz, 4H, Py-*H*), 8.15 (d, *J* = 1.8 Hz, 4H, *o*-Ph-*H*), 7.85 (t, *J* = 1.9 Hz, 2H, *p*-Ph-*H*), 1.58 (s, 36H, CH_3), -3.01 (s, 2H, NH_2) ppm.

See appendix for spectrum. The measured data is in agreement with the literature.[7]

Synthesis of 5

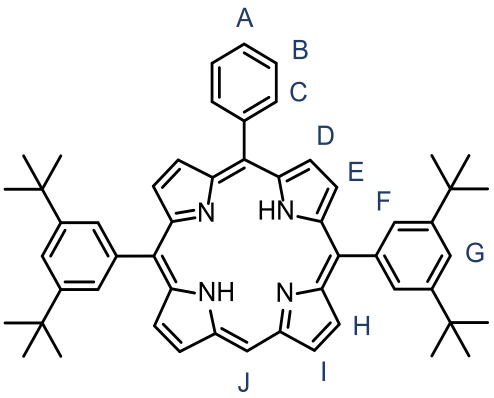

Synthesis adapted from literature procedures.[8,9] Under N_2, porphyrin **4** (100 mg, 0.15 mmol, 1.0 equiv.) was dissolved in anhydrous THF (30 mL). At 0 °C, phenyl lithium (1.6 mL, 1.9 M in Bu_2O, 3.0 mmol, 20 equiv.) was added dropwise and the mixture was stirred for 30 min at 0 °C and for further 30 min at room temperature. Water (~2 mL) was added, and the colour of the mixture changed from red to green. DDQ (136 mg, 0.6 mmol, 4.0 equiv.) was added, causing a colour change back to red and the mixture was stirred for 18 h. The mixture was dried over $MgSO_4$, filtered, and concentrated under reduced pressure. The crude product was purified by filtration through a silica plug (eluent petroleum ether/DCM 3:1) yielding porphyrin **5** as red solid (88 mg, 0.12 mmol, 77%)

^{1}H NMR (400 MHz, $CDCl_3$) δ = 10.23 (s, 1H, H_J), 9.36 (d, *J* = 4.6 Hz, 2H, H_I), 9.09 (d, *J* = 4.6 Hz, 2H, H_H), 8.97 (d, *J* = 4.8 Hz, 2H, H_D), 8.89 (d, *J* = 4.8 Hz, 2H, H_E), 8.27 – 8.19 (m, 2H, H_C), 8.14 (d, *J* = 1.8 Hz, 4H, H_F), 7.84 (t, *J* = 1.8 Hz, 2H, H_G), 7.81 – 7.70 (m, 3H, $H_{A, B}$), 1.57 (s, 36H, CH_3) ppm.

See appendix for spectrum. The measured data is in agreement with the literature.[8]

Synthesis of 6

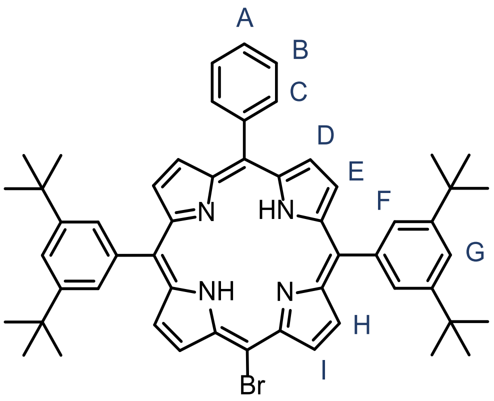

Synthesized according to a literature procedure.[10] Porphyrin **5** (88 mg, 0.12 mmol, 1.0 equiv.) was dissolved in $CHCl_3$ (15 mL) and pyridine (0.1 mL). NBS (23 mg, 0.13 mmol, 1.1 equiv.) was added and the reaction was stirred for 2h. After TLC confirmed the completion of the reaction, acetone was added, and all solvents were removed under reduced pressure. The crude product was filtered through a short silica plug (eluent: petroleum ether/DCM 1:1) to yield porphyrin **6** as red solid (94 mg, 0.11 mmol, 97%).

^{1}H NMR (400 MHz, $CDCl_3$) δ = 9.71 (d, *J* = 4.8 Hz, 2H, H_I), 8.98 (d, *J* = 4.8 Hz, 2H, H_H), 8.87 (d, *J* = 4.9 Hz, 2H, H_D), 8.83 (d, *J* = 4.8 Hz, 2H, H_E), 8.24 – 8.18 (m, 2H, H_C), 8.10 (d, *J* = 1.8 Hz, 4H, H_F), 7.85 (t, *J* = 1.9 Hz, 2H, H_G), 7.76 (d, *J* = 7.2 Hz, 3H, $H_{A,B}$), 1.57 (s, 36H, CH_3), -2.66 (s, 2H, NH) ppm.

See appendix for spectrum. The measured data is in agreement with the literature.[10]

Synthesis of 7

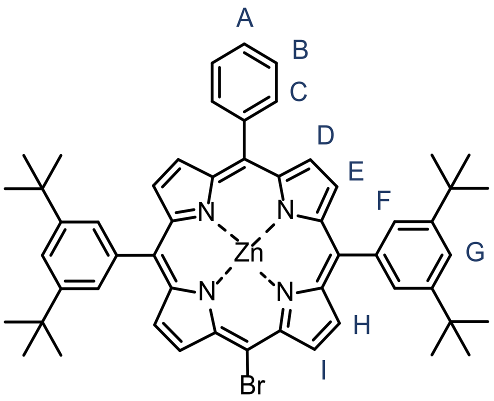

Synthesized according to a literature procedure.[11] Porphyrin **6** (94 mg, 0.11 mmol, 1.0 equiv.) was dissolved in DCM (30 mL). $Zn(OAc)_2$ (103 mg, 0.56 mmol, 5.0 equiv.) was dissolved in methanol (5 mL) and added to the solution. The mixture was refluxed for 2h and after cooling down to room temperature filtered through a short silica plug (eluent: DCM) to yield porphyrin **7** as red solid (110 mg, 0.11 mmol, 100%).

^{1}H NMR (400 MHz, $CDCl_3$) δ = 9.80 (d, *J* = 4.7 Hz, 2H, H_I), 9.06 (d, *J* = 4.7 Hz, 2H, H_H), 8.95 (d, *J* = 4.6 Hz, 2H, H_D), 8.91 (d, *J* = 4.7 Hz, 2H, H_E), 8.21 – 8.15 (m, 2H, H_C), 8.07 (d, *J* = 1.8 Hz, 4H, H_F), 7.82 (t, *J* = 1.8 Hz, 2H, H_G), 7.79 – 7.70 (m, 3H, $H_{A,B}$), 1.54 (s, 36H, CH_3) ppm.

See appendix for spectrum. The measured data is in agreement with the literature.[11]

Synthesis of 8

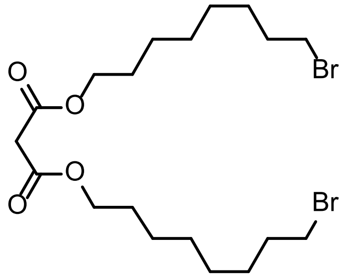

Under N_2, 8-bromooctanol (2.9 mL, 17 mmol, 1.0 equiv.), DMAP (40 mg, 0.30 mmol, 0.02 equiv.) and NEt_3 (2.6 mL, 18 mmol, 1.1 equiv.) was dissolved in anhydrous DCM (100 mL) at 0 °C. A solution of malonyl chloride (0.90 mL, 9.2 mmol, 0.55 equiv.) in anhydrous DCM (15 mL) was added dropwise and the resulting mixture was stirred for 1 h at 0 °C and warmed to room temperature for 12 h. The mixture was washed with 1 M HCl, brine and the organic fraction was dried over $MgSO_4$. The solvent was evaporated, and the crude product was purified by column chromatography (PE/DCM 1:4) to give the product **8** as a colorless liquid (2.6 g, 5.4 mmol, 64%).

^{1}H NMR (400 MHz, $CDCl_3$): δ = 4.12 (t, 4H, *J* = 6.7 Hz, O-C*H*$_2$), 3.39 (t, 4H, *J* = 6.8 Hz, Br-C*H*$_2$), 3.35 (s, 2H, malonyl-*H*), 1.83-1.31 (m, 24H, alkyl-*H*) ppm.

^{13}C NMR (101 MHz, $CDCl_3$): δ = 166.7, 65.6, 41.7, 34.0, 32.8, 29.0, 28.7, 28.5, 28.1, 25.7 ppm.

See appendix for spectra.

Synthesis of 9

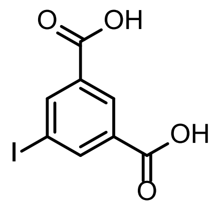

1,3-Dimethyl 5-iodo-1,3-benzenedicarboxylate (5.00 g, 15.6 mmol) was dissolved in methanol (250 mL). An aqueous solution of sodium hydroxide (250 mL, 1M, 46.9 mmol, 3.0 equiv.) was added and the mixture was heated to 85 °C for 2.5 h. After cooling down to room temperature, hydrochloric acid (150 mL, 1 M) was added to basify the mixture (pH 2-4). After cooling to 0 °C the product to precipitated as colourless solid. After filtration, the solid was dried on air to yield pure **9**. The remaining solution was treated again with 1 M hydrochloric acid and methanol was removed under reduced pressure. After filtration and drying on air a second fraction of the product was obtained. Total yield: 3.65 g, 12.5 mmol, 80%.

^{1}H NMR (400 MHz, DMSO-d_6): δ =8.42 (s, 2H, Ph-*H*), 13.51 (bs, 2H, COO-*H*) ppm.

See appendix for spectra. The measured data is in agreement with the literature.[12]

Synthesis of 10

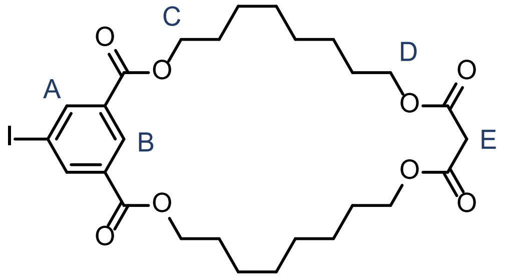

Under N_2, $KHCO_3$ (1.65 mg, 16.4 mmol, 4.0 equiv.) was dissolved in 1.5 L anhydrous DMF. 5-Iodo-1,3-benzenedicarboxylic acid **9** (1.33 g, 4.5 mmol, 1.1 equiv.) was added and the solution was stirred for 10 min at rt. A solution of **8** (2.00 g, 4.11 mmol, 1.0 equiv.) in 40 mL anhydrous DMF was added slowly and the resulting mixture was heated to 90 °C for 4 days. After cooling to room temperature, the solvent was evaporated and the crude product was extracted with DCM and washed with water. The combined organic fractions were dried over $MgSO_4$ and the solvent was removed under reduced pressure. Purification by column chromatography (DCM → 5% EtAc in DCM) yielded macrocycle **10** as a colorless solid (1.78 g, 2.89 mmol, 70%).

^{1}H NMR (400 MHz, $CDCl_3$): δ = 8.57 (d, J = 1.5 Hz, 2H, H_A), 8.53 (t, J = 1.5 Hz, 1H, H_B), 4.35 (t, J = 6.1 Hz, 4H, H_C), 4.15 (t, J = 6.6 Hz, 4H, H_D), 3.36 (s, 2H, H_E), 1.85 – 1.72 (m, 4H, alkyl-H), 1.68 – 1.61 (m, 4H, alkyl-H), 1.53 – 1.44 (m, 4H, alkyl-H), 1.41 – 1.31 (m, 12H, alkyl-H) ppm.

^{13}C NMR (101 MHz, $CDCl_3$): δ = 166.7, 164.5, 142.8, 132.5, 129.3, 93.8, 65.8, 65.7, 42.3, 29.4, 29.3, 28.7, 28.6, 26.3, 26.0 ppm.

MALDI-MS (HR): m/z calc. for $C_{27}H_{38}IO_8$: 617.1611, found: 617.1607 [M+H], 639.1425 $[M+Na]^+$, 655.1163 $[M+K]^+$.

See appendix for spectra.

Synthesis of 11

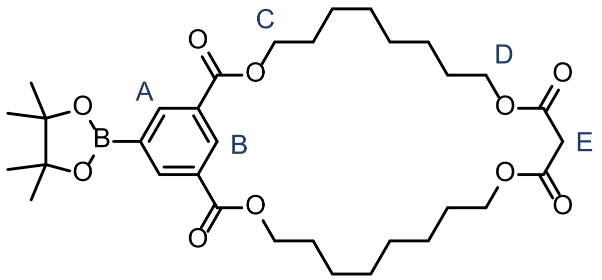

Under N_2, **10** (800 mg, 1.30 mmol, 1.0 equiv), Bis(pinacolato)diboron (3.30 g, 13.0 mmol, 10 equiv.), potassium acetate (382 mg, 3.89 mmol, 3.0 equiv.) and palladium acetate (29 mg, 0.13 mmol, 0.1 equiv.) were suspended in anhydrous degassed DMF (10 mL) and heated to 90 °C for 21 h. DMF was removed under reduced pressure, water was added, and the mixture was extracted with DCM. The combined organic phases were dried over $MgSO_4$, concentrated and the crude product was purified by column chromatography (petroleum ether/ethyl acetate 9:1 → 3:1) to yield **11** as colourless solid (733 mg, 1.18 mmol, 91%).

^{1}H NMR (400 MHz, $CDCl_3$): δ = 8.67 (s, 3H, $H_{A,B}$), 4.35 (t, J = 6.2 Hz, 4H, H_C), 4.14 (t, J = 6.6 Hz, 4H, H_D), 3.36 (s, 2H, H_E), 1.82 – 1.72 (m, 4H, alkyl-H), 1.68 – 1.62 (m, 4H, alkyl-H), 1.55 – 1.47 (m, 4H, alkyl-H), 1.38 – 1.37 (m, 12H, alkyl-H), 1.36 (s, 12H, CH_3) ppm.

^{13}C NMR (101 MHz, $CDCl_3$): δ = 166.7, 165.9, 140.3, 132.7, 130.3, 84.5, 65.7, 65.3, 42.3, 29.4, 29.3, 28.8, 28.6, 26.3, 26.0, 25.0, 25.0 ppm.

MALDI-MS (HR): m/z = calc. for $C_{33}H_{50}BO_{10}$: 617.3499, found: 617.3487 $[M+H]^+$, 639.3304 $[M+Na]^+$, 655.3043 $[M+K]^+$.

See appendix for spectra.

Synthesis of 12

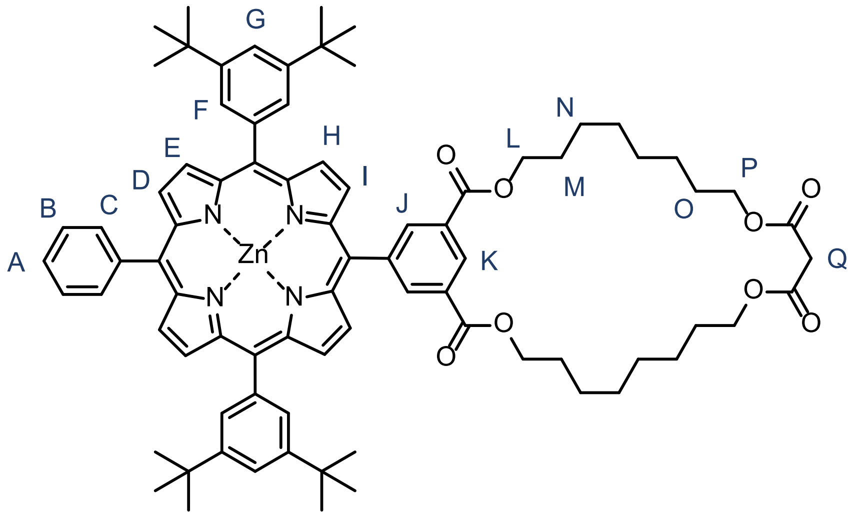

Under N_2, porphyrin **7** (384 mg, 0.42 mmol, 1.0 equiv.), macrocycle **11** (308 mg, 0.5 mmol, 1.2 equiv.), $Pd(PPh_3)_4$ (49 mg, 0.042 mmol, 0.1 equiv.) and K_2CO_3 (580 mg, 4.2 mmol, 10 equiv.) were suspended in a mixture of anhydrous toluene (15 mL) and DMF (7.5 mL) and degassed with a N_2 stream for 10 min. The mixture was heated to 90 °C for 20 h, the solvents were removed under reduced pressure and the crude mixture was purified by column chromatography (DCM) yielding **12** as red-violet solid (481 mg, 0.37 mmol, 88%)

^{1}H NMR (600 MHz, $CDCl_3$) δ = 9.11 (d, *J* = 1.6 Hz, 2H, H_J), 9.08 – 9.02 (m, 5H, $H_{E;H,K}$), 8.97 (d, *J* = 4.6 Hz, 2H, H_D), 8.82 (d, *J* = 4.6 Hz, 2H, H_I), 8.24 (d, *J* = 6.3 Hz, 2H, H_C), 8.13 (d, *J* = 1.8 Hz, 4H, H_F), 7.83 (s, 2H, H_G), 7.80 – 7.68 (m, 3H, $H_{A,B}$), 4.47 (t, *J* = 6.0 Hz, 4H, H_L), 4.19 (t, *J* = 6.6 Hz, 4H, H_P), 3.37 (s, 2H, H_Q), 1.92 – 1.87 (m, 4H, H_O), 1.75 – 1.70 (t, m, 4H, H_M), 1.65 (t, *J* = 7.6 Hz, 4H, H_N), 1.56 (s, 36H, CH_3), 1.52 – 1.42 (m, 12H, CH_2) ppm.

^{13}C NMR (151 MHz, $CDCl_3$) δ = 166.7, 166.2, 150.9, 150.7, 150.4, 149.8, 148.8, 144.2, 143.0, 141.8, 138.7, 134.5, 133.0, 132.6, 132.2, 131.4, 130.0, 129.4, 129.3, 127.6, 126.7, 123.0, 121.6, 121.0, 118.1, 65.7, 65.7, 42.3, 35.2, 31.9, 29.5, 29.4, 28.9, 28.7, 26.5, 26.0 ppm.

MALDI-MS (HR): *m/z* = calc. for $C_{81}H_{92}N_4O_8Zn$: 1312.6207, found: 1312.6190 $[M]^+$

See appendix for spectra. Signals were assigned by means of H,H-COSY, HMBC, HSQC and ROESY NMR.

Synthesis of 13

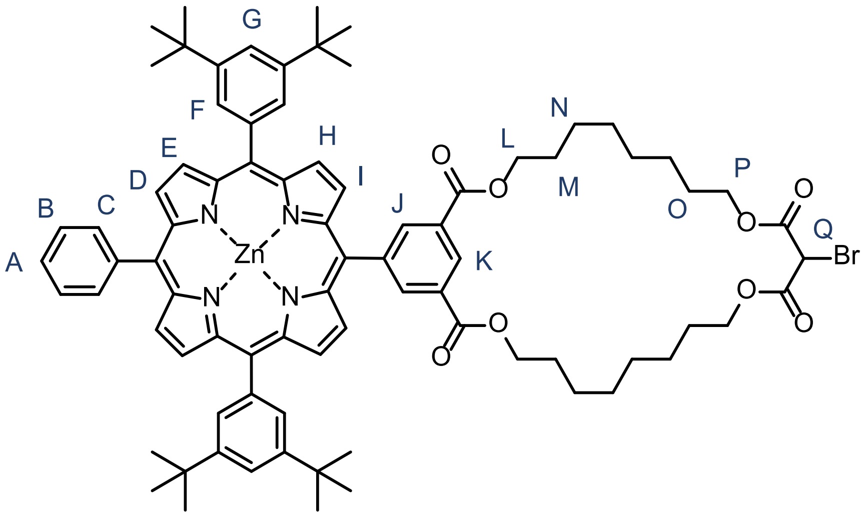

Under N_2, stock solutions of CBr_4 (240 mg) in anhydrous THF (10 mL) and DBU (110 µL) in anhydrous THF (10 mL) were prepared. **12** (80 mg, 61 µmol, 1.0 equiv.) was dissolved in anhydrous THF (3 mL) under nitrogen atmosphere and cooled down to -78 °C. 1 mL of the DBU stock solution (73 µmol, 1.2 equiv.) was added and the reaction was stirred for 10 min before 1 mL CBr_4 stock solution (73 µmol, 1.2 equiv.) was added. After 1 h, TLC still showed remains of starting material **12**, therefore another 0.25 mL (0.3 equiv.) of the stock solutions from DBU and CBr_4 were added. The reaction was stirred for 1 further hour at -78 °C and then quenched by addition of aqueous NH_4Cl solution. THF was removed under reduced pressure and the mixture was extracted with DCM. The combined organic layers were dried over $MgSO_4$, concentrated and the crude mixture was purified by column chromatography (petroleum ether / DCM 2:1 → DCM) yielding **13** as purple solid (63 mg, 45 µmol, 74%).

^{1}H NMR (400 MHz, $CDCl_3$) δ = 9.11 (d, J = 1.6 Hz, 2H, H_J), 9.04 – 9.01 (m, 5H, $H_{K,H,E}$), 8.97 (d, J = 4.6 Hz, 2H, H_D), 8.81 (dd, J = 4.7, J = 1.7 Hz, 2H, H_I), 8.23 (dd, J = 7.6, J = 1.8 Hz, 2H, H_C), 8.12 (d, J = 1.8 Hz, 4H, H_F), 7.82 (t, J = 1.8 Hz, 2H, H_G), 7.78 – 7.71 (m, 3H, $H_{A,B}$), 4.88 (s, 1H, H_Q), 4.47 (t, J = 6.1 Hz, 4H, H_L), 4.38 – 4.21 (m, 4H, H_P), 1.90 – 1.85 (m, 4H, H_M), 1.80 – 1.59 (m, 8H, $H_{O,N}$), 1.55 (s, 36H, CH_3), 1.52 – 1.40 (m, 12H, CH_2). ppm.

^{13}C NMR (101 MHz, $CDCl_3$) δ = 166.2, 164.7, 150.9, 150.7, 150.7, 150.4, 149.7, 148.8, 144.2, 143.0, 141.8, 138.7, 134.5, 133.0, 132.6, 132.2, 131.4, 130.0, 129.4, 129.3, 127.6, 126.7, 123.0, 121.6, 121.0, 118.1, 67.4, 65.6, 43.2, 35.2, 31.9, 29.5, 29.3, 28.9, 28.6, 26.5, 25.9. ppm.

MALDI-MS (HR): m/z = calc. for $C_{81}H_{91}BrN_4O_8Zn$: 1392.5306 found: 1392.5294 $[M]^+$

See appendix for spectra.

Synthesis of 14

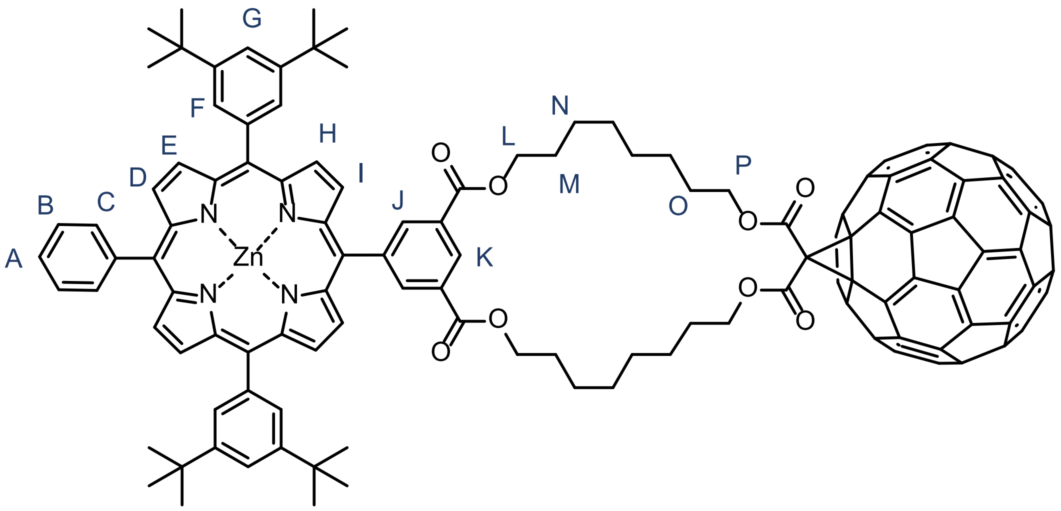

Under N_2, **13** (100 mg, 72 µmol, 1.0 equiv.) and C_{60} (103 mg, 143 µmol, 2.0 equiv.) were dissolved in anhydrous toluene (100 mL) under nitrogen atmosphere. DBU (32 µL, 214 µmol, 3.0 equiv.) was added dropwise and the mixture was stirred for 1 d at room temperature. The reaction was quenched with NH_4Cl solution, and the aqueous phase was extracted with toluene followed by DCM. The combined organic phases were dried over $MgSO_4$, and the solvent was removed under reduced pressure. The product was purified by column chromatography (petroleum ether / ODCB 3:1 → petroleum ether / DCM 4:1 → DCM) yielding **14** as red solid (105 mg, 52 µmol, 72%).

^{1}H NMR (400 MHz, $CDCl_3$) δ = 9.11 (d, J = 1.6 Hz, 2H, H_J), 9.05 (t, J = 1.6 Hz, 1H, H_K), 9.01 (d, J = 4.7 Hz, 4H, $H_{E,H}$), 8.96 (d, J = 4.7 Hz, 2H, H_D), 8.81 (d, J = 4.7 Hz, 2H, H_I), 8.23 (dd, J = 7.5, J =1.8 Hz, 2H, H_C), 8.10 (d, J = 1.8 Hz, 4H, H_F), 7.81 (t, J = 1.8 Hz, 2H, H_G), 7.79 – 7.71 (m, 3H, $H_{A,B}$), 4.43 – 4.45 (m, 8H, $H_{L,P}$), 1.98 – 1.87 (m, 8H, $H_{M,O}$), 1.74 – 1.66 (m, 4H, H_N), 1.56 (s, 12H, CH_2), 1.54 (s, 36H, CH_3) ppm.

^{13}C NMR (151 MHz, $CDCl_3$) δ = 166.2, 163.8, 150.9, 150.7, 150.4, 149.8, 148.8, 145.2, 145.1, 145.0, 144.9, 144.6, 144.5, 144.5, 144.4, 144.3, 143.7, 143.0, 142.9, 142.8, 142.8, 142.0, 141.8, 140.8, 139.1, 138.8, 134.5, 133.0, 132.6, 132.2, 131.4, 130.0, 129.5, 129.3, 127.7, 126.7, 123.1, 121.6, 121.0, 118.1, 71.7, 67.6, 65.7, 52.3, 35.2, 31.9, 29.6, 29.5, 29.0, 28.8, 26.6, 26.2 ppm.

Note: All 16 expected C_{60}-sp2 carbon signals were observed in the ^{13}C NMR.

MALDI-MS (HR): m/z calc. for $C_{141}H_{90}N_4O_8Zn$: 2032.6085, found: 2032.6045 $[M]^+$

See appendix for spectra.

Synthesis of 1

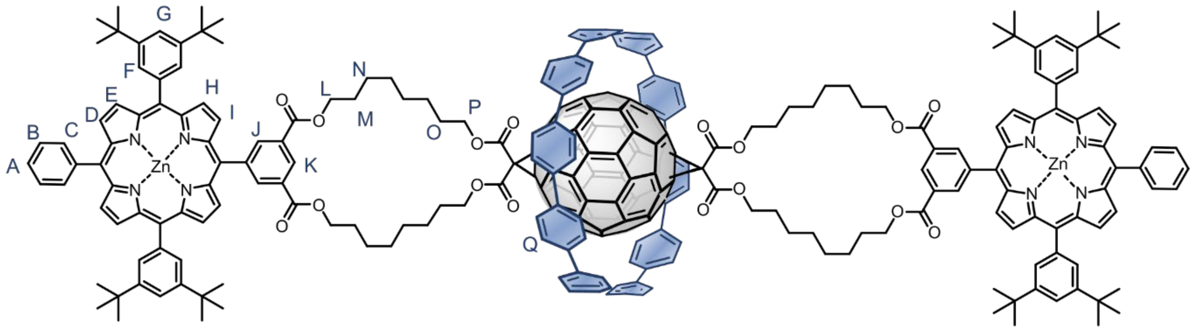

Under N_2, a stock solution of *tert*-butylimino-tri(pyrrolidino)phosphorane (0.90 mL) in anhydrous DCM (10 mL) was prepared.

14 (20.0 mg, 9.83 µmol, 1.0 equiv.) and [10]CPP (7.5 mg, 9.83 µmol, 1.0 equiv.) were dissolved in DCM (50 mL) which was subsequently removed under reduced pressure to afford the self-assembled pseudorotaxane **15**. A ^{1}H NMR was recorded to confirm the 1:1 stoichiometry in the pseudorotaxane which was used without further purification.

Under N_2, the previously prepared pseudorotaxane **15** and **13** (27.4 mg, 19.66 µmol, 2.0 equiv.) were dissolved in anhydrous DCM (5 mL) and cooled down to -78 °C. 0.1 mL of the *tert*-butylimino-tri(pyrrolidino)phosphorane stock solution (29.49 µmol, 3.0 equiv.) was added dropwise and the solution was stirred for 22 h while it was allowed to warm up to room temperature. The reaction was quenched with NH_4Cl solution, and the aqueous phase was extracted with toluene. The combined organic phases were dried over $MgSO_4$, and the solvent was removed under reduced pressure. The crude product was purified by HPLC (Si, toluene / *n*-hexane 87/13) to yield the three regioisomers of **1** (9.7 mg, 2.4 µmol, 24%) as red solids. Samples of pure *trans*-2-**1** (2.8 mg, 0.68 µmol, 7%) and *trans*-3-**1** (5.1 mg, 1.24 µmol, 13%) were obtained from HPLC.

MALDI-MS (HR): *m/z* = calc. for $C_{282}H_{220}N_8O_{16}Zn_2$: 4107.5296 found:4107.5313 $[M]^+$

trans-2

^{1}H NMR (600 MHz, CD_2Cl_2) δ = 9.13 – 9.09 (m, 6H, $H_{J,K}$), 9.05 (dd, ^3J = 4.6, J = 3.9 Hz, 4H, H_H), 9.01 (dd, J = 5.8 Hz, ^3J = 4.6 Hz, 4H, H_E), 8.96 (dd, ^3J = 4.6, J = 3.8 Hz, 4H, H_D), 8.88 (dd, ^3J = 4.6 Hz, J = 2.1 Hz, 4H, H_I), 8.23 (dt, ^3J = 6.6 Hz, ^4J = 1.6 Hz, 4H, H_C), 8.11 (d, ^4J = 1.9 Hz, 8H, H_F), 7.86 (dt, J = 3.5 Hz, ^4J =1.8 Hz, 4H, H_G), 7.81 – 7.74 (m, 6H, $H_{A,B}$), 7.44 (s, 40H, H_Q), 4.74 (dt, J = 10.5 Hz, ^3J = 6.7 Hz, 2H, $H_{L/P}$), 4.68 (dt, J = 10.5 Hz, ^3J = 6.7 Hz, 2H, $H_{L/P}$), 4.65 – 4.57 (m, 4H, $H_{L/P}$), 4.55 (dt, J = 11.8 Hz, ^3J = 5.9 Hz, 8H, $H_{L/P}$), 2.16 – 2.08 (m, 4H, $H_{O/M}$), 2.08 – 1.95 (m, 12H, $H_{O/M}$), 1.84 – 1.62 (m, 36H, CH_2), 1.55 (d, J = 4.9 Hz, 72H, CH_3) ppm.

^{13}C NMR (151 MHz, CD_2Cl_2) δ = 166.51, 164.37, 163.95, 151.33, 151.18, 150.82, 150.31, 150.29, 149.39, 146.76, 145.89, 145.18, 145.00, 144.55, 144.49, 144.20, 144.18, 144.16,

143.68, 143.37, 143.26, 143.03, 142.98, 142.84, 142.82, 142.75, 142.43, 142.22, 141.90, 141.62, 141.28, 141.22, 141.09, 140.84, 140.33, 140.21, 139.46, 139.12, 138.28, 137.09, 136.89, 134.97, 133.31, 132.93, 132.56, 131.79, 131.75, 130.38, 130.15, 129.64, 128.12, 127.90, 127.15, 123.39, 122.02, 121.71, 118.71, 71.17, 70.54, 67.97, 67.83, 66.19, 66.16, 49.91, 35.53, 32.06, 32.05, 30.17, 30.11, 30.09, 29.59, 29.51, 29.46, 27.13, 27.11, 26.85, 26.84 ppm.

Note: All 28 expected C_{60}-sp^2 carbon signals were observed in the ^{13}C NMR spectrum.

MALDI-MS (HR): *m/z* = calc. for $C_{282}H_{220}N_8O_{16}Zn_2$: 4107.5296 found:4107.5266 [M]$^+$

trans-3

^{1}H NMR (600 MHz, CD_2Cl_2) δ = 9.12 - 9.1 (m, 6H, $H_{J,K}$), 9.05 (d, ^3J = 4.6 Hz, 4H, H_H), 9.01 (dd, J = 7.5 Hz, ^3J = 4.6 Hz, 4H, H_E), 8.96 (t, J = 4.4 Hz, 4H, H_D), 8.87 (dd, ^3J = 4.6 Hz, J = 1.4 Hz, 4H, H_I), 8.23 (d, ^3J = 6.4 Hz, 4H, H_C), 8.11 (dd, J = 3.6 Hz, ^4J = 1.9 Hz, 8H, H_F), 7.86 (dt, J = 7.7 Hz, ^4J = 1.8 Hz, 4H, H_G), 7.82 – 7.73 (m, 6H, $H_{A,B}$), 7.44 (s, 40H, H_Q), 4.71 – 4.47 (m, 16H, $H_{L,P}$), 2.10 – 2.04 (m, 4H, $H_{O/M}$), 2.03 – 1.94 (m, 12H, $H_{O/M}$), 1.81 – 1.62 (m, 32H, $H_{N,}$ CH_2), 1.55 (s, 36H, CH_3), 1.54 (d, J = 1.0 Hz, 36H, CH_3) ppm.

^{13}C NMR (151 MHz, CD_2Cl_2) δ = 166.49, 163.95, 163.92, 151.33, 151.18, 150.82, 150.29, 149.39, 146.29, 146.03, 145.76, 145.63, 145.28, 145.19, 145.09, 145.02, 144.48, 144.27, 144.06, 143.56, 143.36, 143.02, 142.88, 142.79, 142.69, 142.27, 142.21, 142.09, 141.85, 141.73, 141.31, 140.51, 140.38, 139.11, 139.04, 138.34, 138.00, 137.97, 134.96, 133.31, 132.93, 132.56, 131.77, 131.74, 130.38, 130.15, 129.63, 128.12, 127.94, 127.14, 123.39, 122.03, 121.70, 118.70, 71.18, 70.76, 67.87, 67.76, 66.16, 51.63, 35.53, 32.06, 32.04, 30.16, 30.11, 30.06, 29.55, 29.47, 29.45, 27.11, 26.84, 26.79 ppm.

Note: 26 out of 28 expected C_{60}-sp^2 carbon signals were observed in the ^{13}C NMR spectrum. The two missing signals are probably overlapped by other signals.

MALDI-MS (HR): *m/z* = calc. for $C_{282}H_{220}N_8O_{16}Zn_2$: 4107.5296 found: 4107.5361 [M]$^+$

See appendix for spectra.

1 was stored in the dark to prevent degradation. Possible sources of acid (like $CHCl_3$) were avoided since they can lead to degradation.

Synthesis of 2

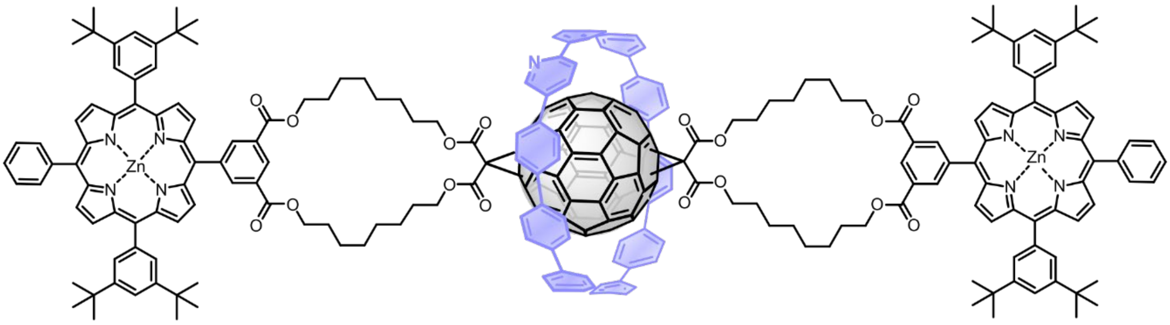

Under N_2, a stock solution of *tert*-butylimino-tri(pyrrolidino)phosphorane (0.52 mL) in anhydrous DCM (10 mL) was prepared.

14 (11.3 mg, 5.55 µmol, 1.0 equiv.) and aza[10]CPP (4.2 mg, 5.55 µmol, 1.0 equiv.) were dissolved in DCM (50 mL) which was subsequently removed under reduced pressure to afford the self-assembled pseudorotaxane **16**. A ^{1}H NMR was recorded to confirm the 1:1 stoichiometry in the pseudorotaxane which was used without further purification.

Under N_2, the previously prepared pseudorotaxane **16** and **13** (15.5 mg, 11.1 µmol, 2.0 equiv.) were dissolved in anhydrous DCM (5 mL) and cooled down to -78 °C. 0.1 mL of the tert-butylimino-tri(pyrrolidino)phosphorane stock solution (16.7 µmol, 3.0 equiv.) were added dropwise and the solution was stirred for 20 h while it was allowed to warm up to room temperature. The reaction was quenched with NH_4Cl solution, and the aqueous phase was extracted with toluene. The combined organic phases were dried over $MgSO_4$, and the solvent was removed under reduced pressure. The crude product was purified by HPLC (PhMe/DCM 35:65 → 20:80) to yield **2** in a mixture of regioisomers as red solid (4.5 mg, 1.1 µmol, 20%).

^{1}H NMR (600 MHz, CD_2Cl_2) δ =9.18 – 9.08 (m, 6H, H_{Porph}), 9.08 – 8.94 (m, 12H, H_{Porph}), 8.93 – 8.86 (m, 4H, H_{Porph}), 8.25 – 8.17 (m, 4H, H_{Porph}), 8.16 – 8.06 (m, 8H, H_{Porph}), 7.88 – 7.72 (m, 4H, H_{Porph}), 7.61 – 7.20 (m, 32H, H_{CPP}), 4.80 – 4.41 (m, 24H, $(CH_2)_n$), 2.18 – 1.89 (m, 24H, $(CH_2)_n$), 1.85 – 1.61 (m, 16H, $(CH_2)_n$), 1.57 – 1.51 (m, 72H, CH_3) ppm.

The additional 7 protons of aza[10]CPP[1] are probably overlapping with the porphyrin signals increasing the integrals of some signals. However, their exact shifts could not be identified undoubtedly. Also in *H,H*-COSY spectra no clear cross peaks were observed for those signals.

^{13}C NMR (151 MHz, CD_2Cl_2) δ = 166.57, 166.51, 164.38, 163.99, 163.89, 151.33, 151.23, 151.17, 150.82, 150.71, 150.27, 150.22, 149.38, 149.33, 146.86, 146.69, 146.26, 145.71, 145.17, 145.08, 144.51, 143.46, 143.38, 142.61, 142.24, 141.79, 141.12, 140.15, 139.36, 139.16, 138.25, 138.19, 138.16, 137.90, 136.89, 134.97, 133.30, 132.92, 132.86, 132.57, 132.51, 132.46, 131.77, 130.52, 130.37, 130.14, 129.62, 129.56, 128.30, 128.11, 128.04,

128.03, 127.95, 127.85, 127.69, 127.57, 127.48, 127.14, 127.09, 123.38, 122.03, 121.91, 121.70, 121.63, 118.74, 118.68, 68.16, 66.23, 66.16, 35.53, 32.06, 30.13, 30.09, 29.52, 29.43, 27.10, 26.79, 26.01 ppm.

Due to the isomeric mixture, the C_{60} signals could not be clearly identified.

MALDI-MS (HR): *m/z* = calc. for $C_{281}H_{219}N_9O_{16}Zn_2$: 4107.5245 found: 4107.5335 $[M]^+$

See appendix for spectra.

2 was stored in the dark to prevent degradation. Possible sources of acid (like $CHCl_3$) were avoided since they can lead to degradation.

Synthesis of 3

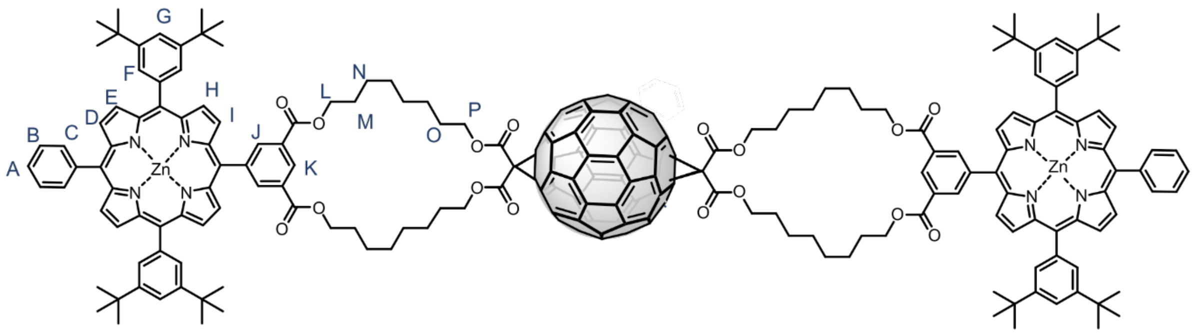

Under N_2, a stock solution of *tert*-butylimino-tri(pyrrolidino)phosphorane (0.44 mL) in anhydrous DCM (10 mL) was prepared.

Under N_2, **14** (15.0 mg, 7.3 µmol, 1.0 equiv.) and **13** (10.2 mg, 7.3 µmol, 1.0 equiv.) were dissolved in anhydrous DCM (4 mL) and cooled down to -78 °C. 0.1 mL of the *tert*-butylimino-tri(pyrrolidino)phosphorane stock solution (14.6 µmol, 2.0 equiv.) were added dropwise and the solution was stirred for 22 h while it was allowed to warm up to room temperature. The reaction was quenched with NH_4Cl solution, and the aqueous phase was extracted with toluene. The combined organic phases were dried over $MgSO_4$, and the solvent was removed under reduced pressure. Column chromatography (petroleum ether / DCM 1:2 → 1:4) yielded **3** as mixture of regioisomers (19.3 mg, 5.77 µmol, 79%).

HPLC (buckyprep, ethyl acetate) was used to isolate 1-2 mg of the *trans-2*, *trans-3* and *e*-isomer.

MALDI-MS (HR): *m/z* = calc. for $C_{222}H_{180}N_8O_{16}Zn_2$: 3346.2143 found: 3346.2162 $[M]^+$

trans-2

^{1}H NMR (600 MHz, CD_2Cl_2) δ = 9.10 – 9.07 (m, 6H, $H_{J,K}$), 9.02 (d, *J* = 4.6 Hz, 4H_H), 8.99 (d, *J* = 4.6 Hz, 4H, H_E), 8.94 (d, *J* = 4.6 Hz, 4H), 8.86 (dd, *J* = 4.6, *J* = 1.8 Hz, 4H, H_I), 8.22 (t, *J* = 7.1 Hz, 4H_C), 8.10 (d, *J* = 1.8 Hz, 8H, H_F), 7.85 (q, *J* = 1.61, 4H, H_G), 7.81 – 7.74 (m, 6H, $H_{A,B}$), 4.66 – 4.58 (m, 4H, $H_{L/P}$), 4.56 – 4.42 (m, 12H, $H_{L/P}$), 2.06 – 1.86 (m, 16H, $H_{O,}$ M), 1.78 – 1.58 (m, 16H, H_N, CH_2), 1.54 (d, J = 1.2 Hz, 72H, CH_3) ppm.

^{13}C NMR (151 MHz, CD_2Cl_2) δ = 166.52, 164.32, 163.94, 151.27, 151.14, 150.77, 150.27, 149.34, 148.56, 147.26, 146.58, 146.39, 145.86, 145.67, 145.41, 145.36, 144.79, 144.64, 144.51, 144.45, 144.23, 143.93, 143.72, 143.65, 143.46, 143.45, 143.30, 142.89, 142.57, 142.52, 142.33, 142.29, 141.93, 141.15, 140.38, 139.23, 138.17, 138.01, 134.97, 133.26, 132.87, 132.50, 131.76, 130.40, 130.13, 129.58, 128.08, 127.11, 123.32, 121.95, 121.67, 118.69, 72.24, 71.65, 68.16, 68.01, 66.13, 60.07, 35.52, 32.05, 30.00, 29.94, 29.89, 29.37, 29.32, 29.22, 26.97, 26.68 ppm.

Note: 26 out of 28 expected C_{60}-sp^2 carbon signals were observed in the ^{13}C NMR spectrum. The two missing signals are probably overlapped by other signals.

MALDI-MS (HR): *m/z* = calc. for $C_{222}H_{1800}N_8O_{16}Zn_2$: 3346.2143 found:3346.2147 [M]$^+$

<u>*trans-3*</u>

^{1}H NMR (600 MHz, CD_2Cl_2) δ = 9.08 – 9.03 (m, 6H, $H_{J,K}$), 9.02 (dd, J = 4.6, 0.9 Hz, 4H, H_H), 8.99 (t, J = 4.6 Hz, 4H, H_E), 8.93 (dd, J = 5.7, 4.6 Hz, 4H, H_D), 8.84 (dd, J = 4.6, 1.0 Hz, 4H, H_I), 8.22 – 8.19 (m, 4H, H_C), 8.10 (t, J = 1.8 Hz, 8H, H_F), 7.85 (dt, J = 3.7, 1.8 Hz, 4H, H_G), 7.79 – 7.71 (m, 6H, $H_{A,B}$), 4.56 – 4.37 (m, 16H, $H_{L,P}$), 1.96 – 1.83 (m, 16H, CH_2), 1.74 – 1.57 (m, 16H, CH_2), 1.53 (dd, J = 3.3, 1.8 Hz, 72H, CH_3) ppm.

^{13}C NMR (151 MHz, CD_2Cl_2) δ = 166.49, 163.92, 163.89, 151.29, 151.17, 150.78, 150.78, 150.29, 149.37, 147.56, 147.22, 147.14, 146.93, 146.81, 146.75, 146.58, 146.17, 146.10, 145.54, 144.86, 144.66, 144.46, 144.18, 144.07, 143.79, 143.60, 143.43, 143.39, 142.76, 142.72, 142.63, 142.24, 142.24, 142.15, 142.02, 140.80, 139.70, 139.15, 139.03, 134.94, 133.29, 132.90, 132.52, 131.78, 130.39, 130.12, 129.58, 128.08, 127.11, 123.36, 121.98, 121.69, 118.71, 72.46, 72.01, 68.05, 67.96, 66.11, 35.52, 32.51, 32.04, 30.26, 30.01, 29.94, 29.89, 29.84, 29.36, 29.25, 29.16, 26.96, 26.66, 26.60, 14.45 ppm.

Note: 27 out of 28 expected C_{60}-sp^2 carbon signals were observed in the ^{13}C NMR spectrum. The missing signal is probably overlapped by other signals.

MALDI-MS (HR): *m/z* = calc. for $C_{222}H_{1800}N_8O_{16}Zn_2$: 3346.2143 found:3346.2159 [M]$^+$

<u>equatorial</u>

^{1}H NMR (600 MHz, CD_2Cl_2) δ = 9.09 – 9.03 (m, 4H, H_J), 9.02 – 8.92 (m, 12H, $H_{K, H, E, D}$), 8.93 8.90 (d, *J* = 4.6 Hz, 1H, H_D), 8.86 (d, *J* = 4.6 Hz, 1H, H_D), 8.83 (dd, *J* = 4.6, *J* = 1.4 Hz, 2H, H_I), 8.80 (d, *J* = 4.6 Hz, 2H, H_I), 8.23 – 8.20 (m, 2H, H_C), 8.16 – 8.13 (m, 2H, H_C), 8.09 (dd, ^4J = 2.9, *J* =1.8 Hz, 8H, H_F), 7.84 (t, J = 1.7 Hz, 4H, H_G), 7.81 – 7.73 (m, 3H, $H_{A,B}$), 7.72 – 7.64 (m, 3H, $H_{A,B}$), 4.51 – 4.41 (m, 10H, $H_{L,P}$), 4.39 (t, *J* = 6.6 Hz, 2H, $H_{L,P}$), 4.32 (t, *J* = 6.0 Hz, 2H, $H_{L,P}$), 1.97 – 1.76 (m, 16H, CH_2), 1.73 -1.56 (m, 8H, CH_2), 1.55 – 1.52 (m, 40H, CH_2, CH_3) ppm.

^{13}C NMR (151 MHz, CD_2Cl_2) δ = 166.49, 166.45, 166.38, 163.96, 163.92, 163.81, 151.26, 151.14, 150.77, 150.24, 149.32, 148.12, 147.36, 146.66, 146.45, 146.21, 145.73, 145.48, 145.12, 145.06, 144.91, 144.88, 144.71, 144.70, 144.58, 144.54, 144.49, 144.20, 144.04, 143.82, 143.70, 143.58, 143.49, 143.13, 142.80, 142.32, 142.12, 142.09, 141.84, 139.19, 139.14, 139.06, 139.00, 134.97, 134.92, 133.22, 132.87, 132.49, 131.72, 130.42, 130.08, 130.03, 129.53, 129.48, 128.05, 127.97, 127.09, 127.02, 123.30, 121.93, 121.64, 118.60,

72.32, 71.05, 67.93, 66.11, 64.21, 35.52, 32.05, 30.27, 30.01, 29.86, 29.37, 29.33, 29.19, 29.13, 26.97, 26.91, 26.61 ppm.

Note: 27 out of 28 expected C_{60}-sp^2 carbon signals were observed in the ^{13}C NMR spectrum. The missing signal is probably overlapped by other signals.

MALDI-MS (HR): *m/z* = calc. for $C_{222}H_{1800}N_8O_{16}Zn_2$: 3346.2143 found:3346.2178 $[M]^+$

4. Transesterification Experiments

General Procedure for Transesterification experiments

Under N_2 ~1 mg of the sample was dissolved in 1 mL anhydrous DCM and 1 ml anhydrous methanol. 10 mg K_2CO_3 were added and the mixture was stirred at room temperature. The reaction was monitored by TLC and after full conversion (typically after 2 -4 h) the mixture was diluted with 5 mL DCM and filtered over a silica plug which was rinsed with more DCM. After concentrating, the mixture was analysed by ^1H NMR. The chemical shifts of the methyl ester protons can be assigned to different C_{60} bisadducts by comparison with the individually prepared C_{60} bisadducts.[13] For the rotaxanes, the comparison was done with the respective [10]CPP complexes.

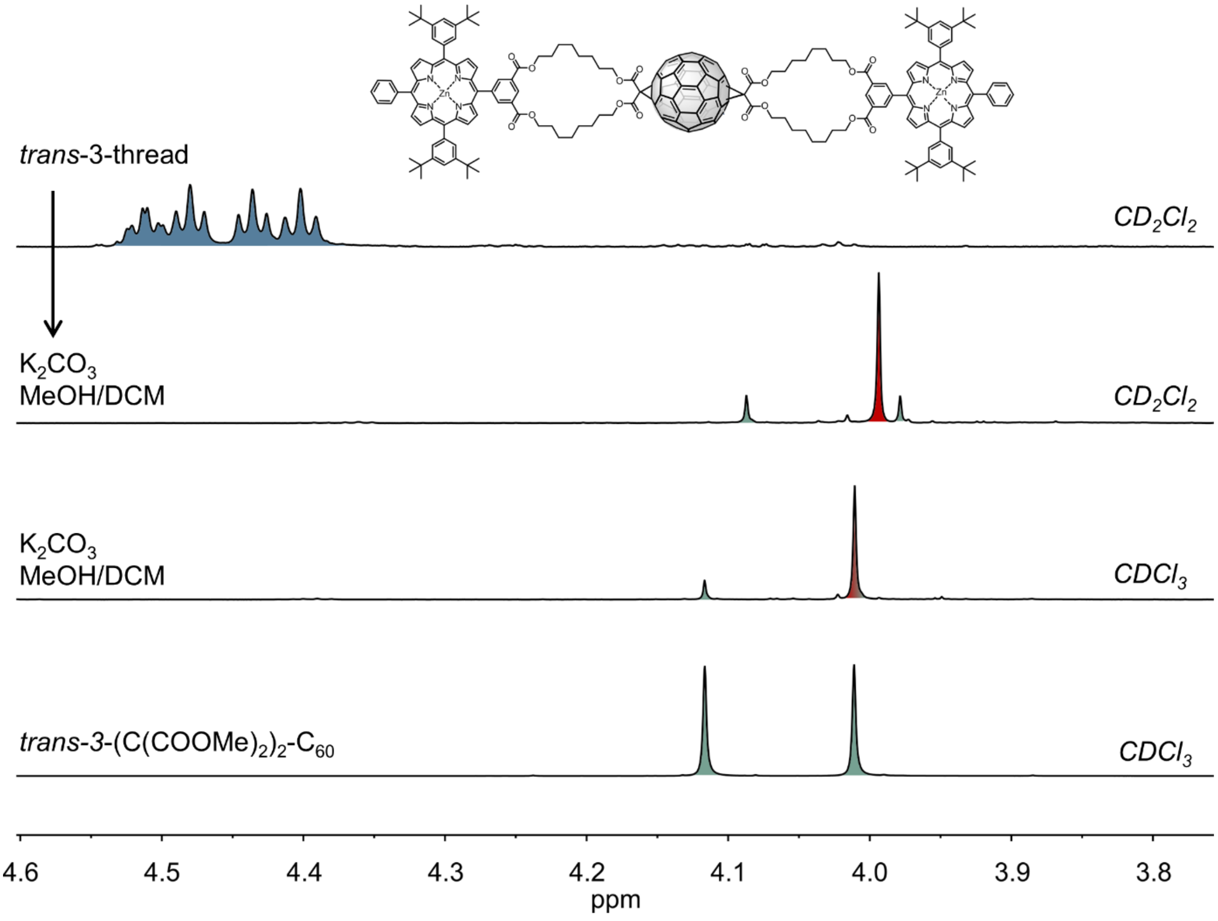

Figure S1: ^1H NMR (600 MHz) before and after transesterification of ***trans-3*-3** compared to the respective C_{60} bisadduct. When measured in $CDCl_3$ one signal of the C_{60} bisadduct (green) overlaps with the methyl ester of the porphyrin (red). When measuring in CD_2Cl_2 those signals separate.

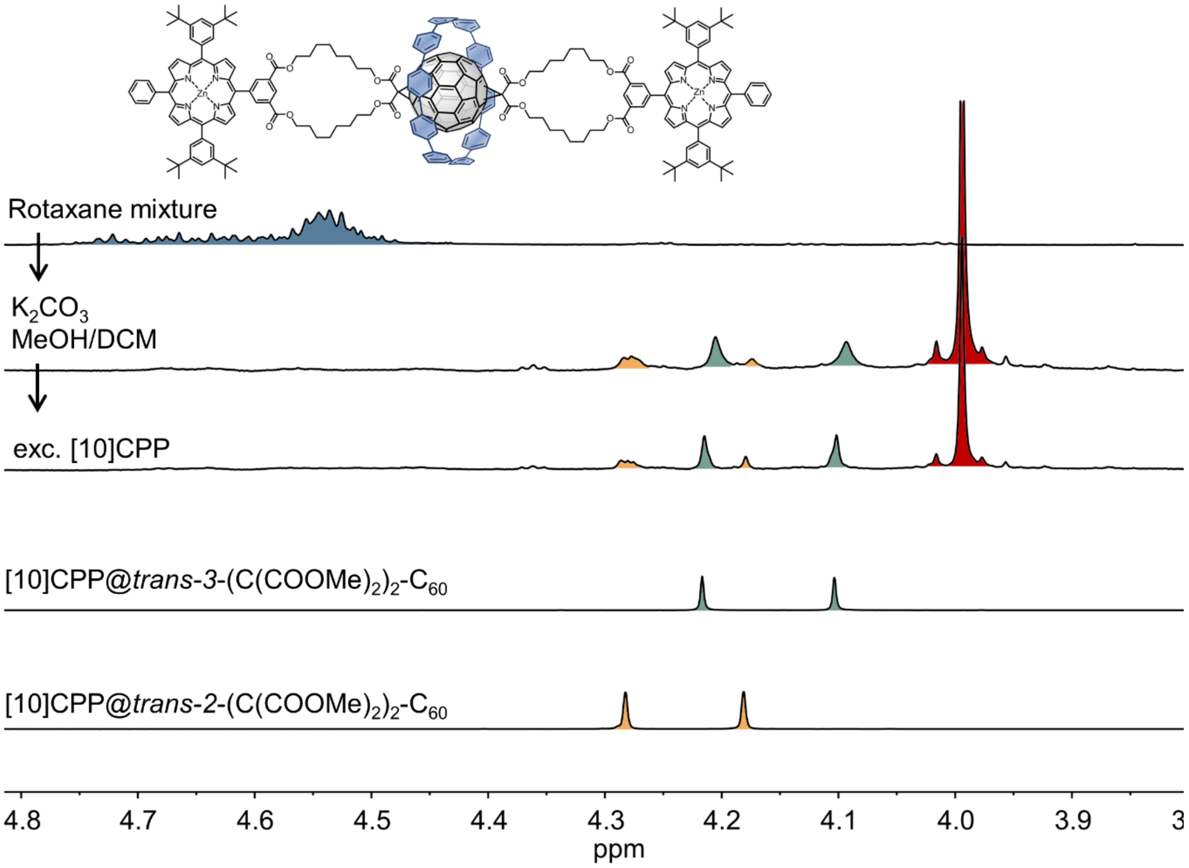

Figure S2: ^{1}H NMR (600 MHz, CD_2Cl_2) before and after transesterification of ***1*** (isomeric mixture) compared to the [10]CPP complexes of the respective C_{60} bisadducts.

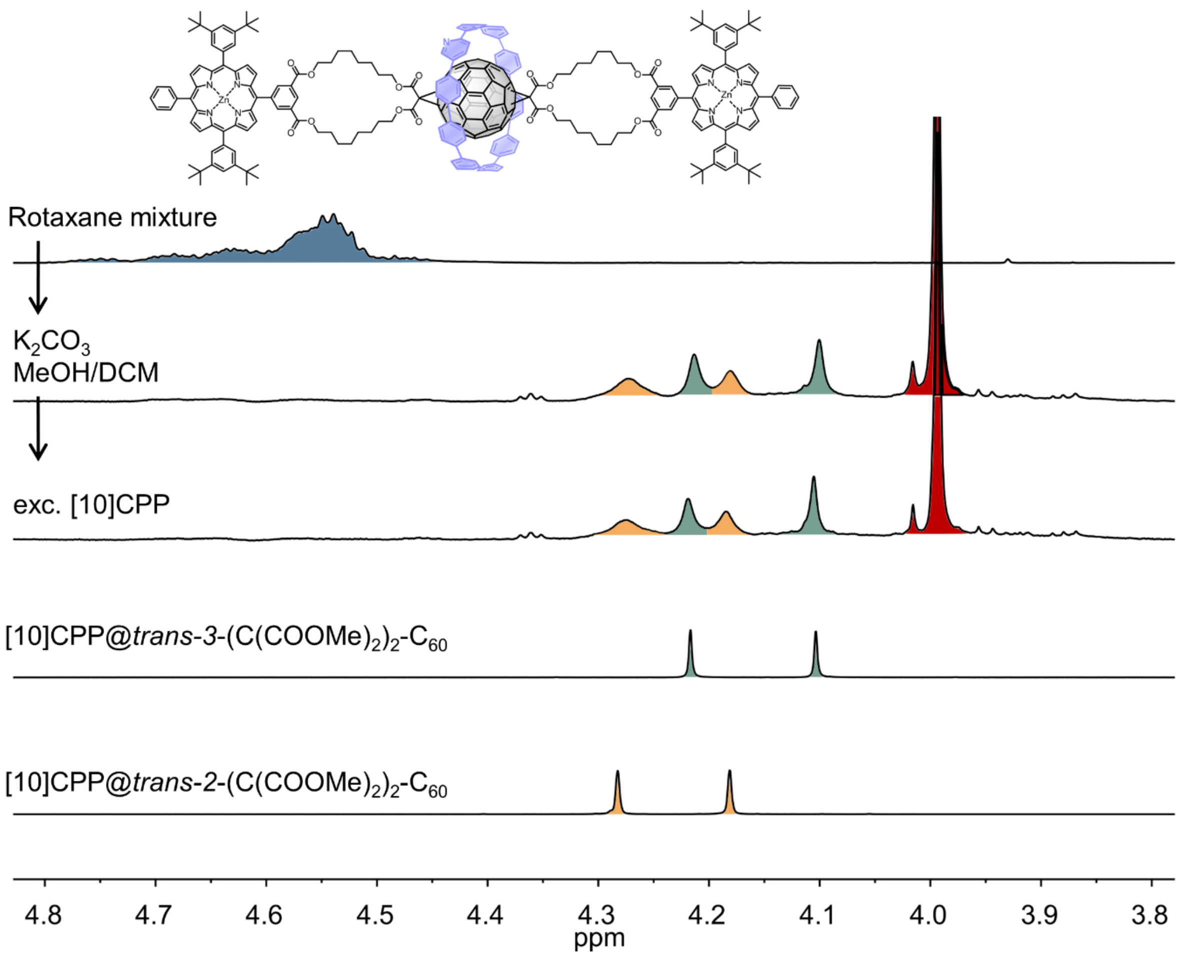

Figure S3: ^{1}H NMR (600 MHz, CD_2Cl_2) before and after transesterification of ***2*** (isomeric mixture) compared to the [10]CPP complexes of the respective C_{60} bisadducts.

5. Additional Data on Rotaxane Synthesis and Purification

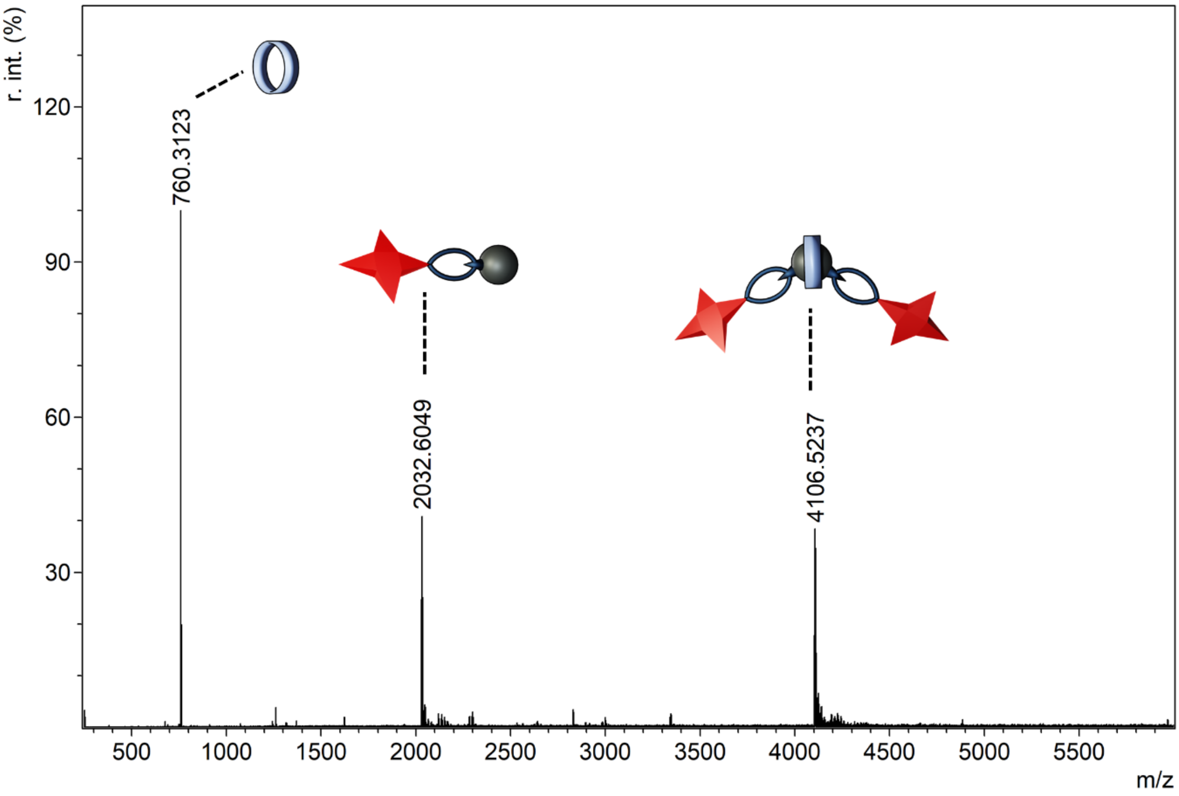

Figure S4: HRMS (MALDI, matrix: DCTB) of the reaction mixture of the synthesis of the [10]CPP Rotaxane

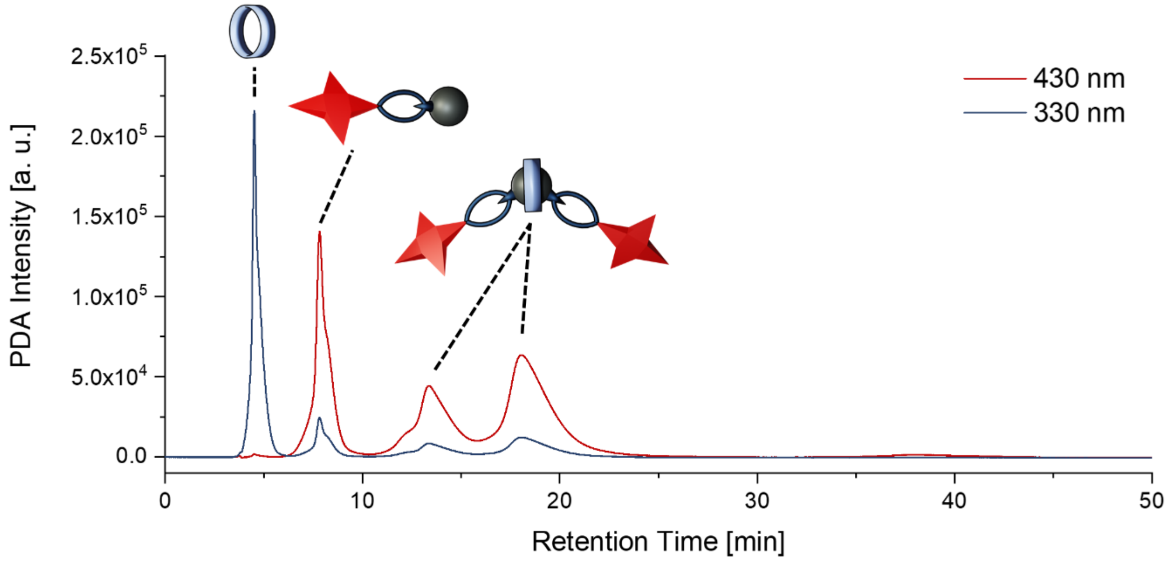

Figure S5: HPLC chromatogram (Si, toluene/n-Hexane 90:10) of the reaction mixture of the synthesis of the [10]CPP Rotaxane

6. MS Experiments

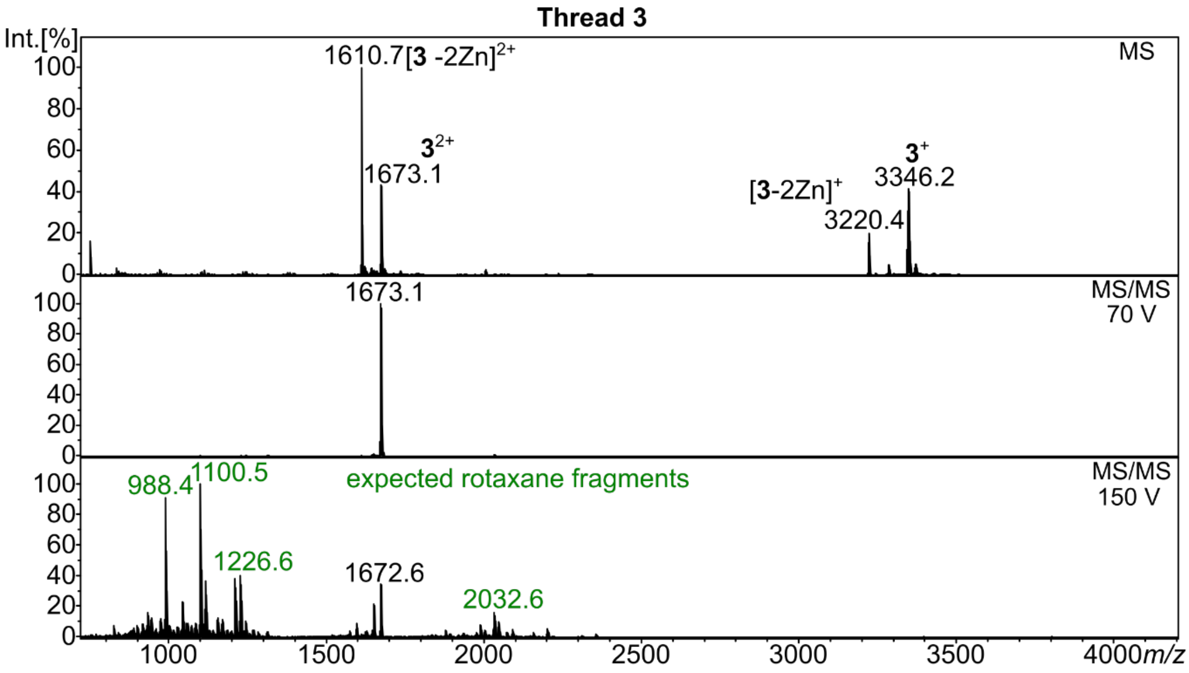

Figure S6: ESI-MS and MS/MS spectra of the thread **3**.

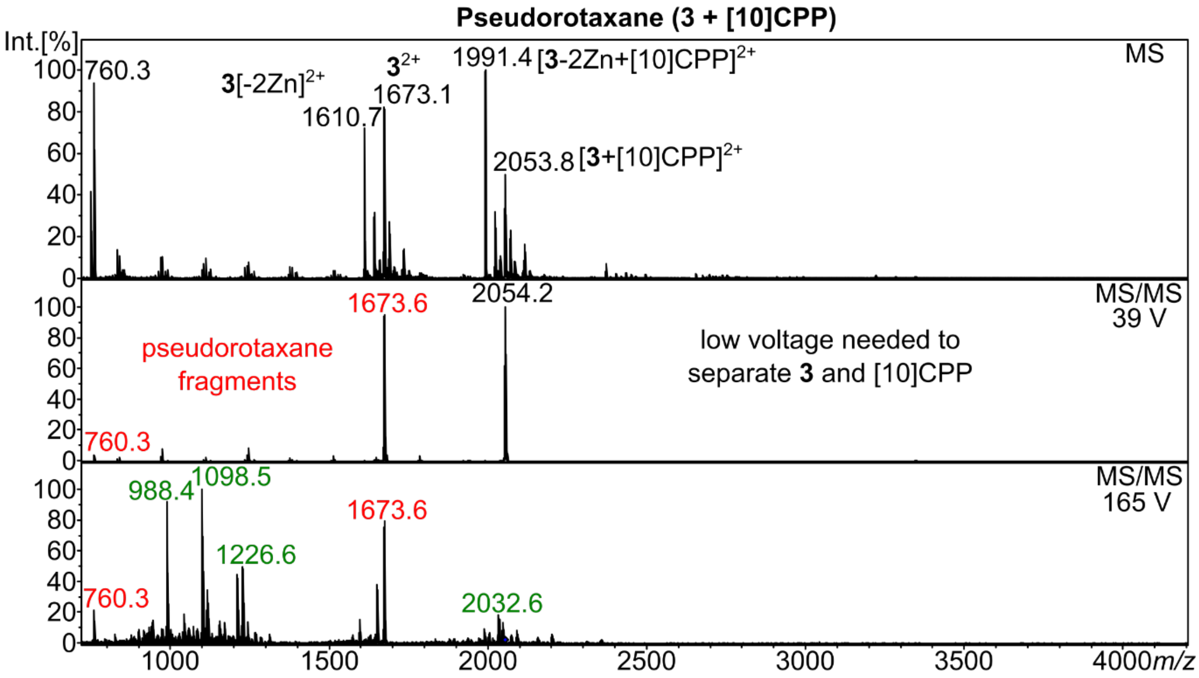

Figure S7: ESI-MS and MS/MS spectra of a pseudorotaxane formed by mixing [10]CPP with the thread **3**. At low acceleration voltage the pseudorotaxane fragments to the thread **3**.

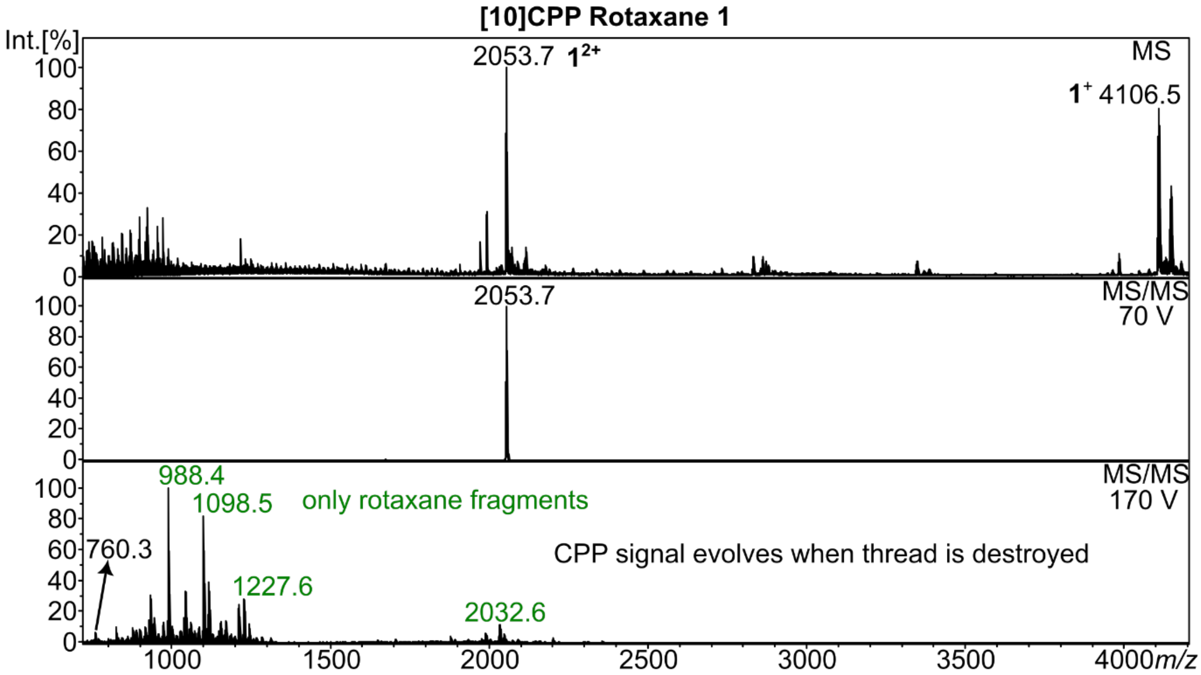

Figure S8: ESI-MS and MS/MS spectra of the rotaxane **1**. At lower acceleration voltage the rotaxane stays intact and the [10]CPP signal only evolves when the thread is destroyed (no formation of the thread **3**) indicating the mechanical interlocking of the CPP.

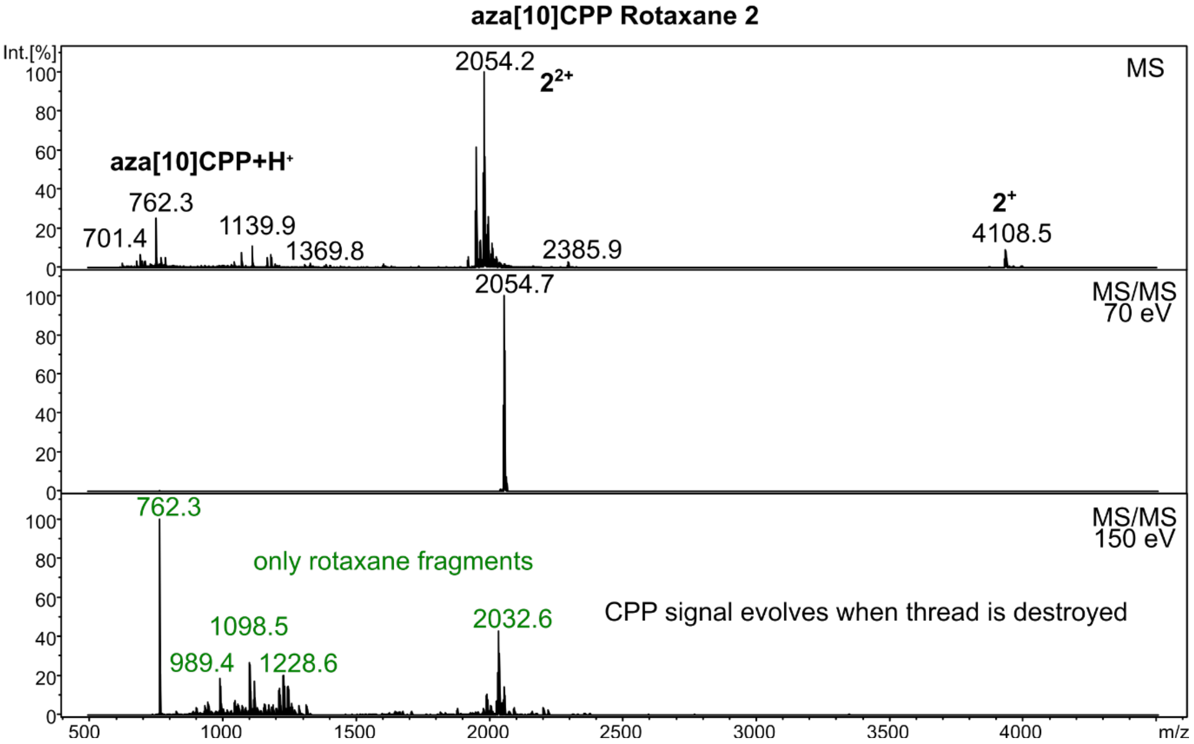

Figure S9: ESI-MS and MS/MS spectra of the rotaxane **2**. At lower acceleration voltage the rotaxane stays intact and the aza[10]CPP signal only evolves when the thread is destroyed (no formation of the thread **3**) indicating the mechanical interlocking of the CPP.

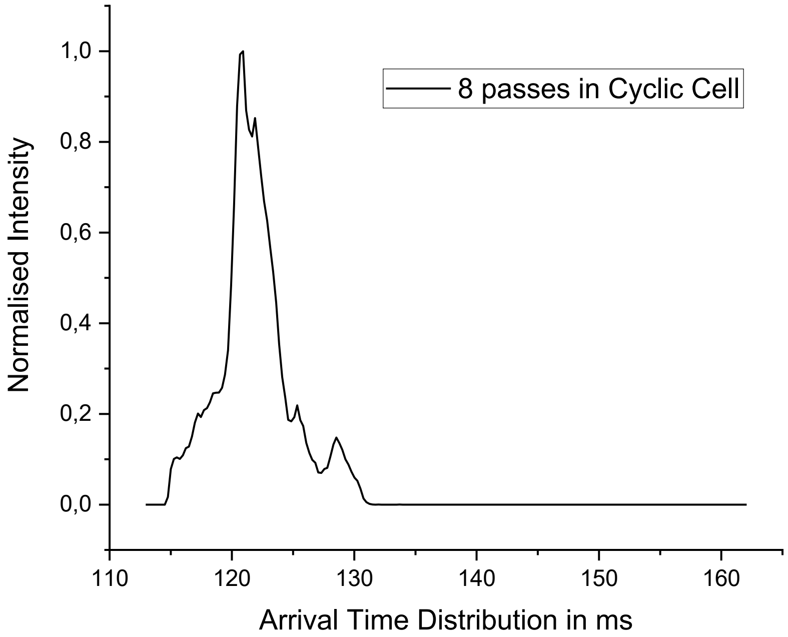

Figure S10: Arrival time distribution of rotaxane ion **1**$^{2+}$ (as isomeric mixture) after eight passes in a cyclic ion-mobility cell indicating the presence of several fullerene regioisomers.

7. Transient Absorption Spectroscopy

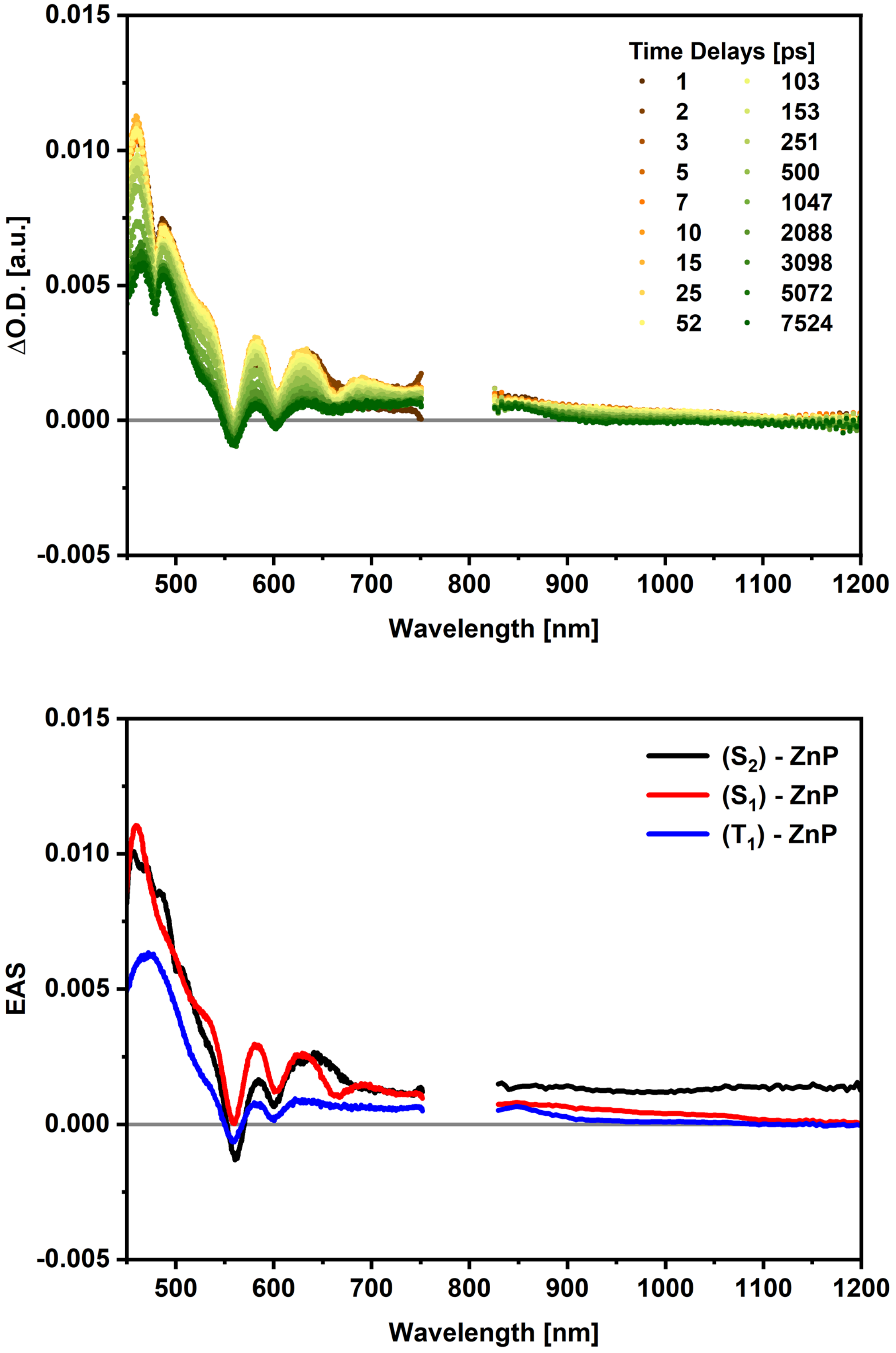

Figure S11: Femtosecond differential absorption spectra of thread **3** (2.5×10^{-6} M) in argon purged PhCN at time delays between 1 and 7525 ps after 430 nm laser excitation. Deconvoluted evolution-associated spectra (EAS) obtained via global analysis are shown beneath, following a sequential deactivation model (black-red-blue). Scattering at 480 nm was corrected by subtracting the background.

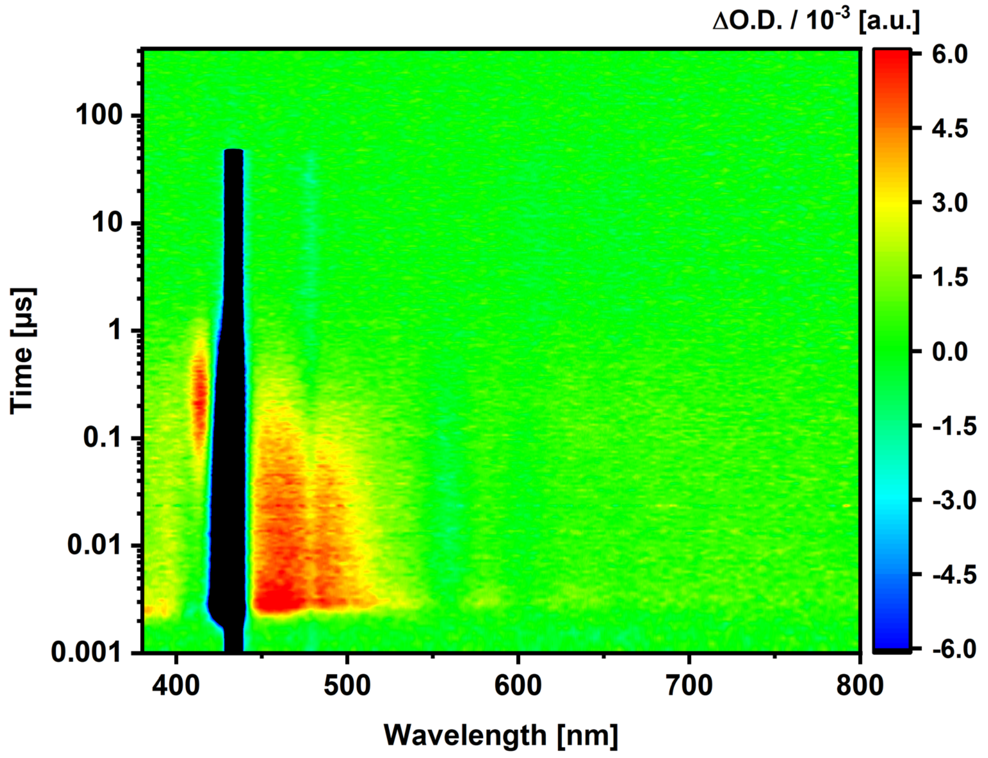

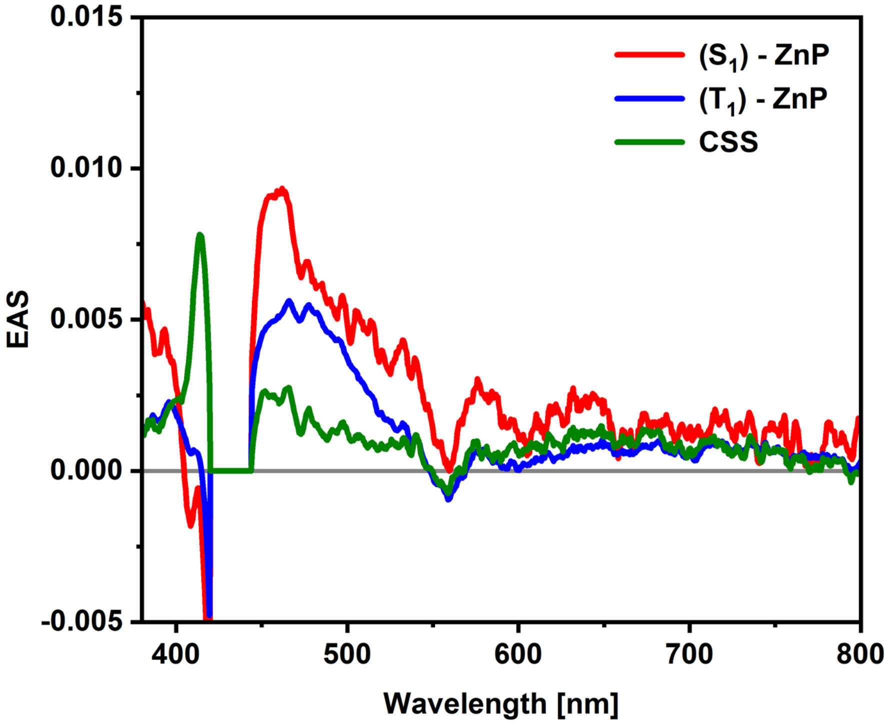

Figure S12: Nanosecond differential absorption spectra of thread **3** (2.5×10^{-6} M) in argon purged PhCN at time delays between 1 ns and 440 µs after 430 nm laser excitation. Deconvoluted evolution-associated spectra (EAS) obtained via global analysis are shown beneath, following a sequential deactivation model (red-blue-green). Scattering at 480 nm was corrected by subtracting the background.

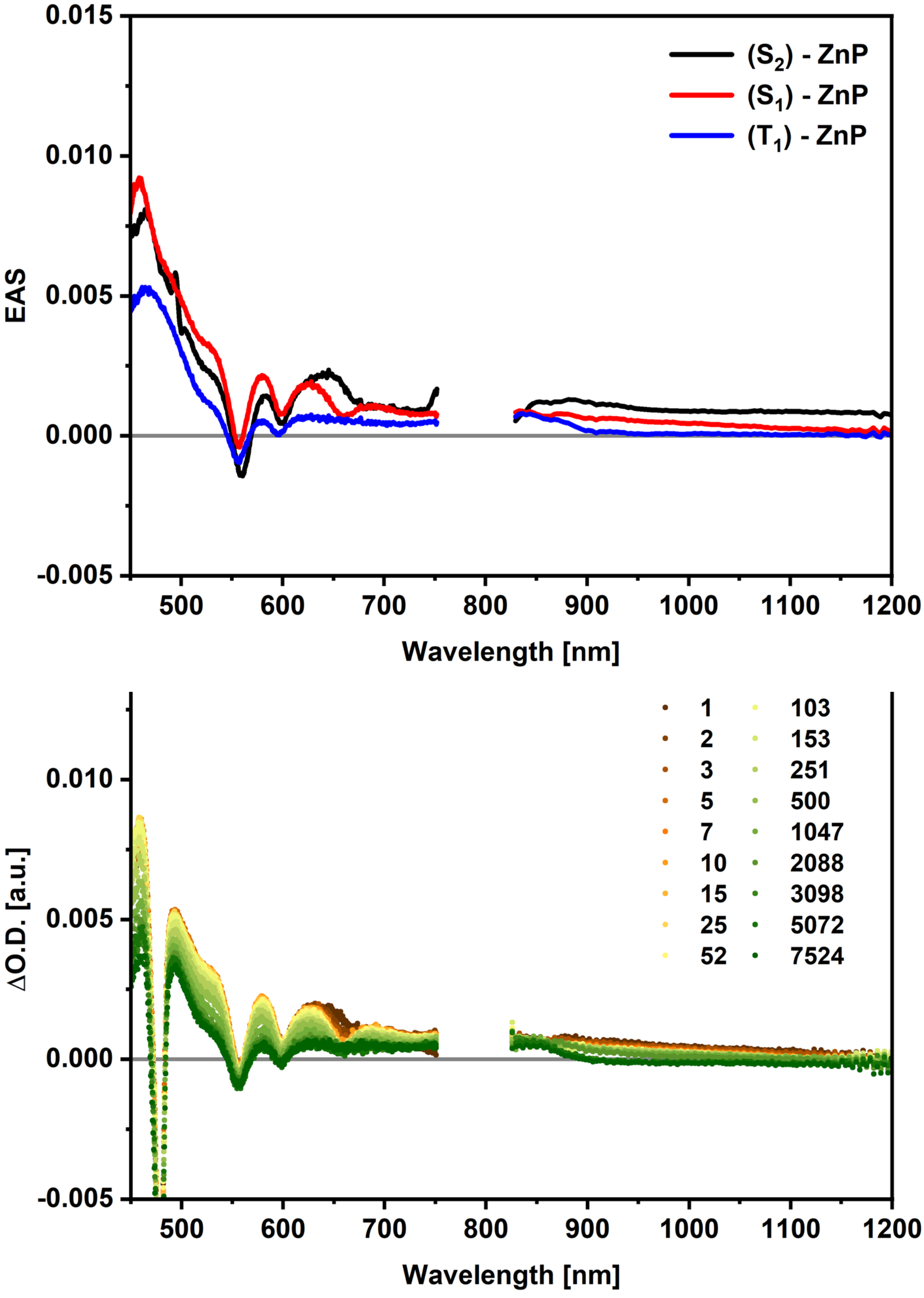

Figure S13: Femtosecond differential absorption spectra of thread **3** (2.5×10^{-6} M) in argon purged THF at time delays between 1 and 7525 ps after 430 nm laser excitation. Deconvoluted evolution-associated spectra (EAS) obtained via global analysis are shown beneath, following a sequential deactivation model (black-red-blue). Scattering at 480 nm was corrected by subtracting the background.

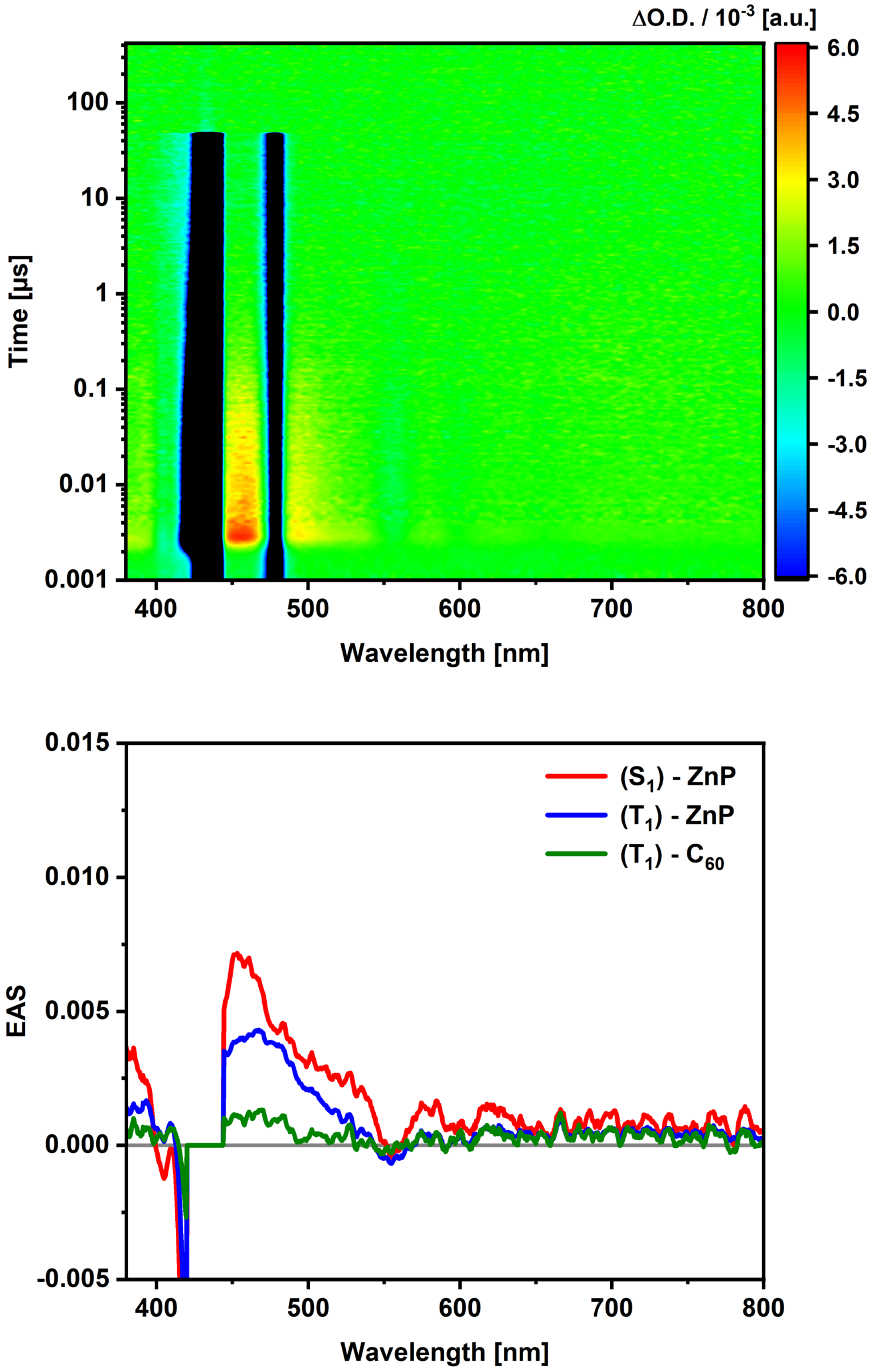

Figure S14: Nanosecond differential absorption spectra of thread **3** (2.5×10^{-6} M) in argon purged THF at time delays between 1 ns and 440 µs after 430 nm laser excitation. Deconvoluted evolution-associated spectra (EAS) obtained via global analysis are shown beneath, following a sequential deactivation model (red-blue-green). Scattering at 480 nm was corrected by subtracting the background.

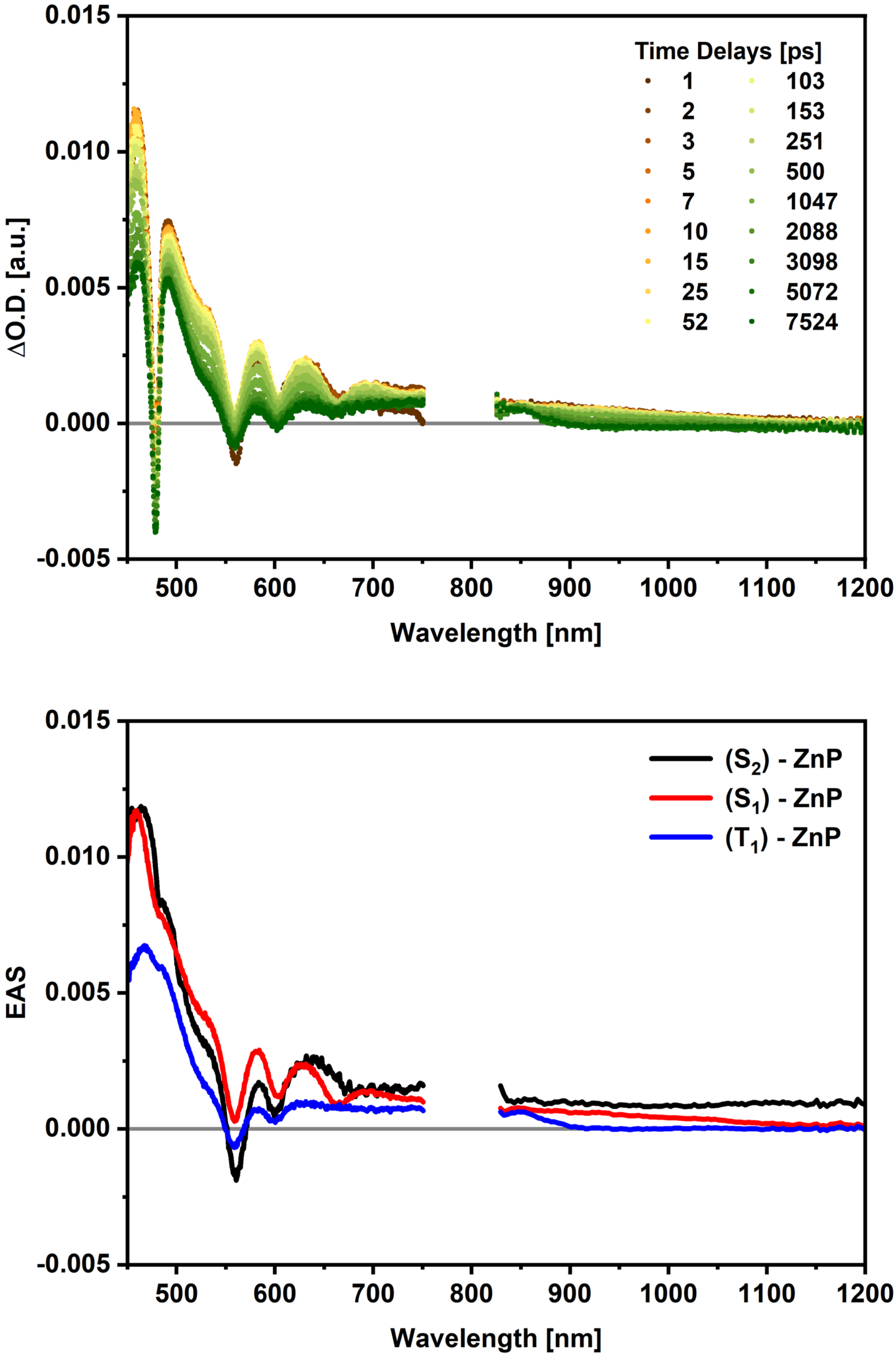

Figure S15: Femtosecond differential absorption spectra of [10]CPP-rotaxane **1** (2.5×10^{-6} M) in argon purged PhCN at time delays between 1 and 7525 ps after 430 nm laser excitation. Deconvoluted evolution-associated spectra (EAS) obtained via global analysis are shown beneath, following a sequential deactivation model (black-red-blue). Scattering at 480 nm was corrected by subtracting the background.

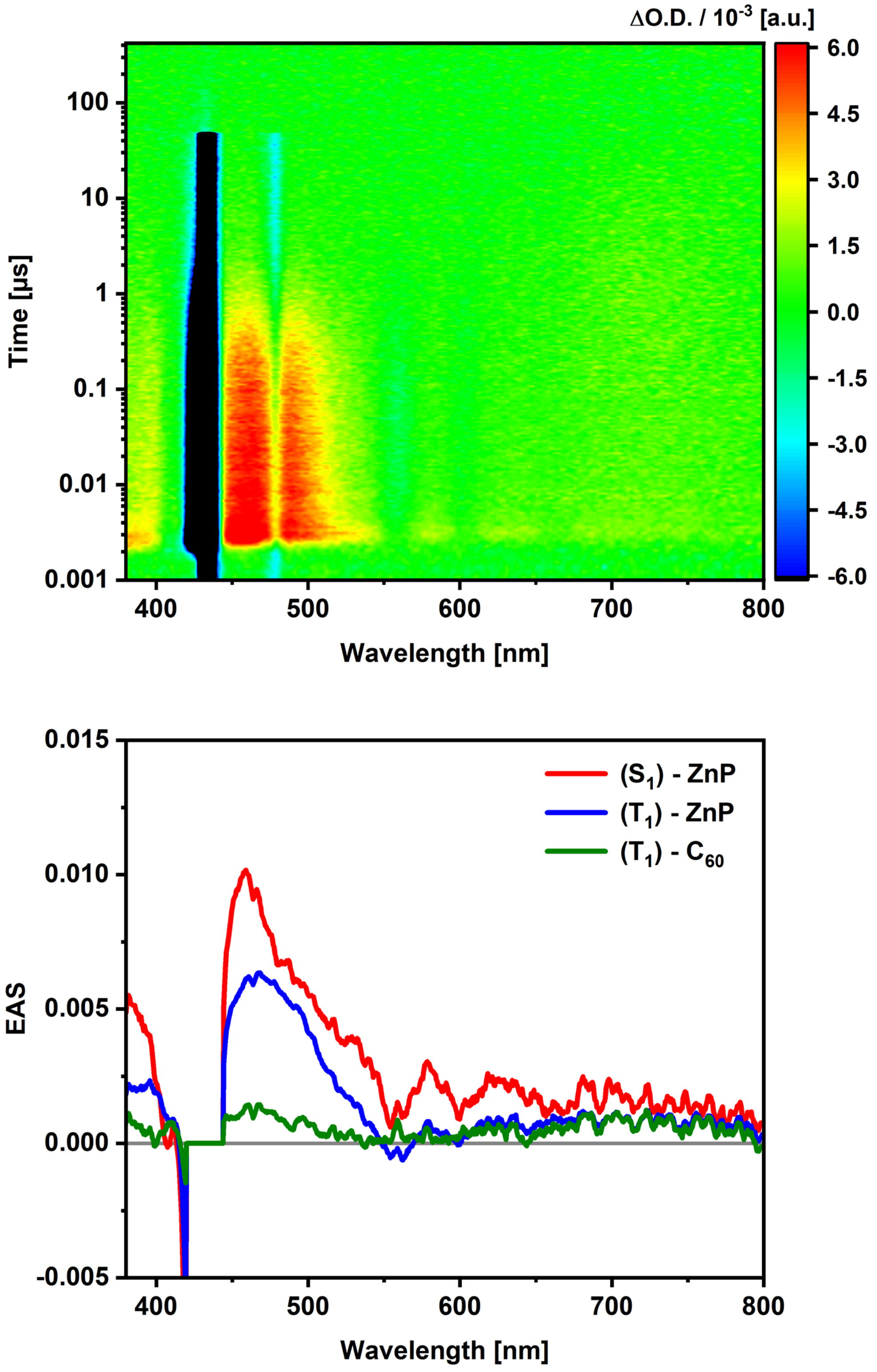

Figure S16: Nanosecond differential absorption spectra of [10]CPP-rotaxane **1** (2.5 x 10^{-6} M) in argon purged PhCN at time delays between 1 ns and 440 µs after 430 nm laser excitation. Deconvoluted evolution-associated spectra (EAS) obtained via global analysis are shown beneath, following a sequential deactivation model (red-blue-green). Scattering at 480 nm was corrected by subtracting the background.

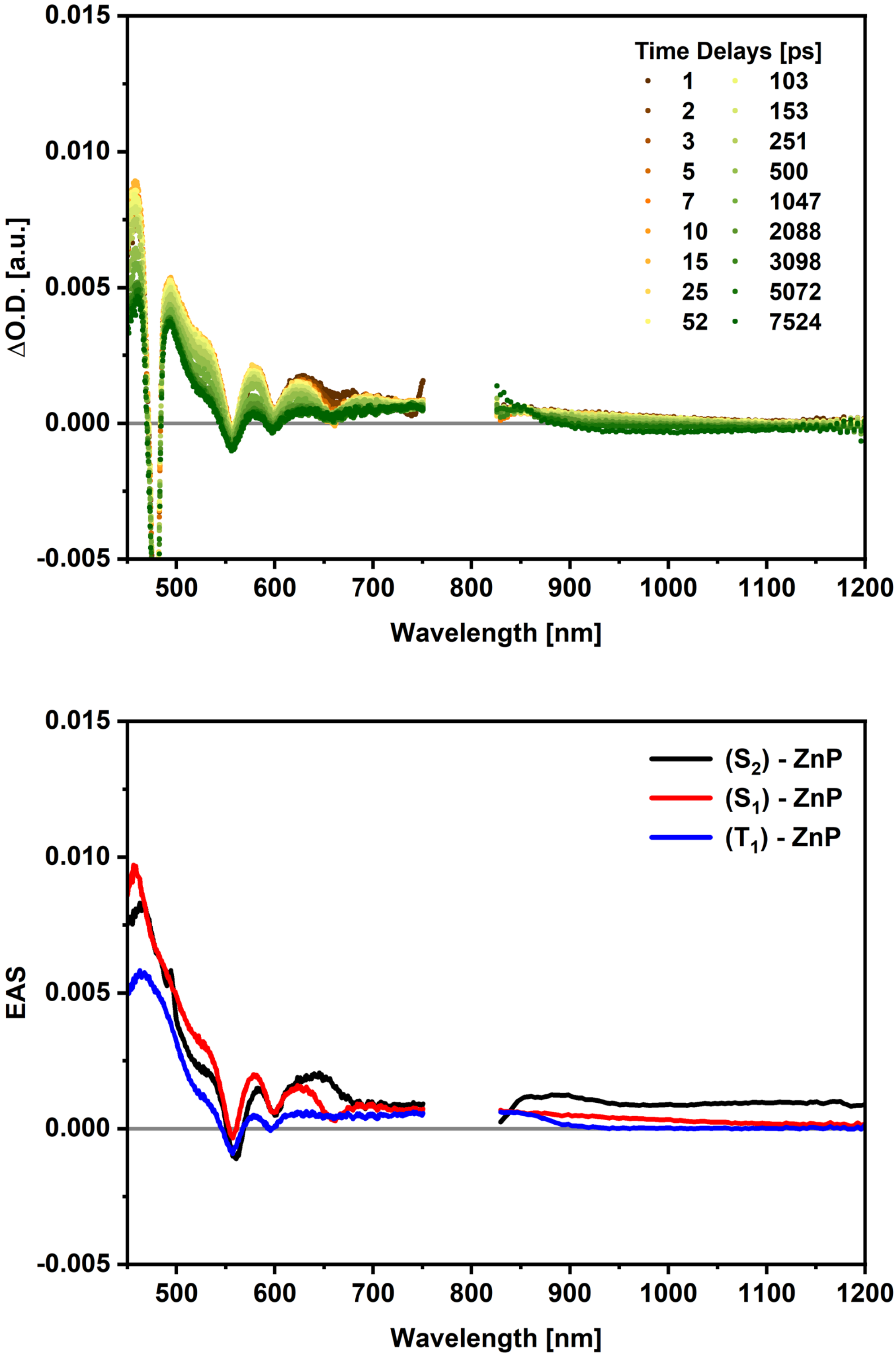

Figure S17: Femtosecond differential absorption spectra of [10]CPP-rotaxane **1** (2.5 x 10^{-6} M) in argon purged THF at time delays between 1 and 7525 ps after 430 nm laser excitation. Deconvoluted evolution-associated spectra (EAS) obtained via global analysis are shown beneath, following a sequential deactivation model (black-red-blue). Scattering at 480 nm was corrected by subtracting the background.

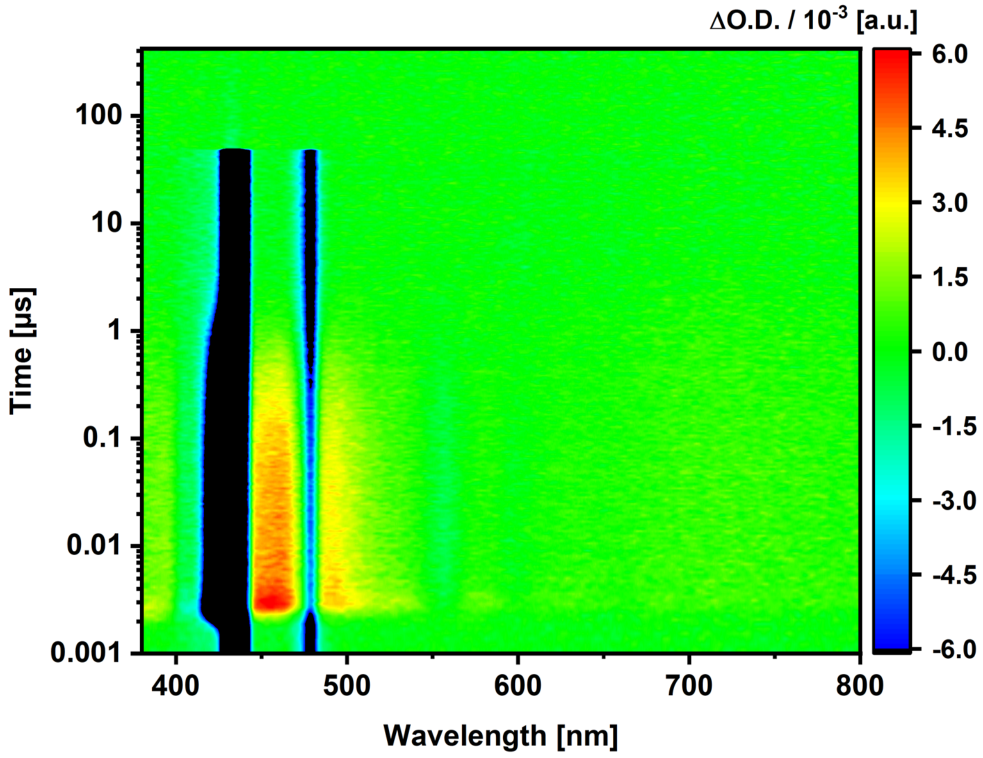

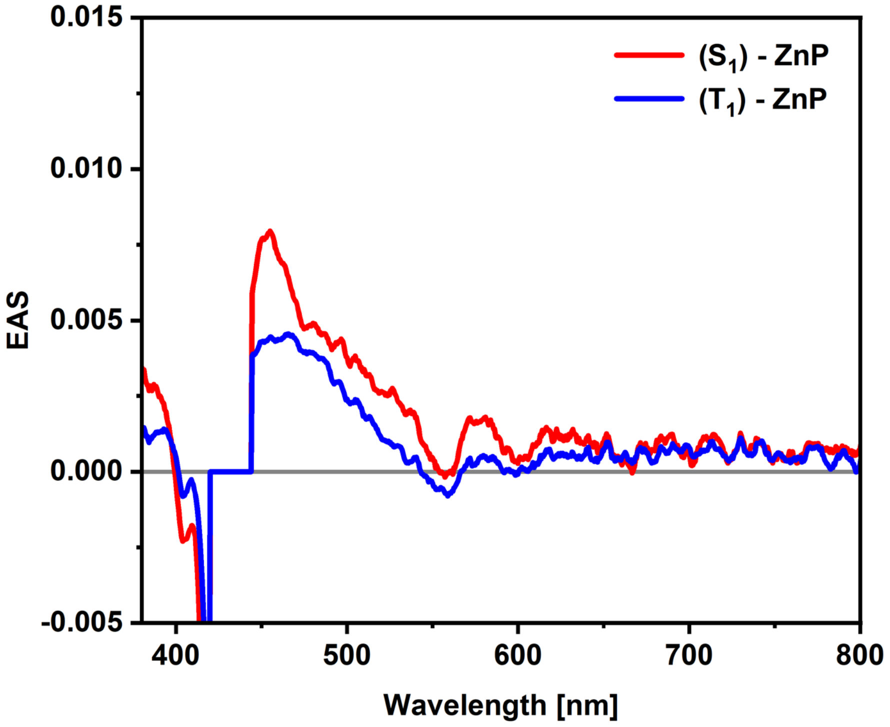

Figure S18: Nanosecond differential absorption spectra of [10]CPP-rotaxane **1** (2.5×10^{-6} M) in argon purged THF at time delays between 1 ns and 440 µs after 430 nm laser excitation. Deconvoluted evolution-associated spectra (EAS) obtained via global analysis are shown beneath, following a sequential deactivation model (red-blue). Scattering at 480 nm was corrected by subtracting the background.

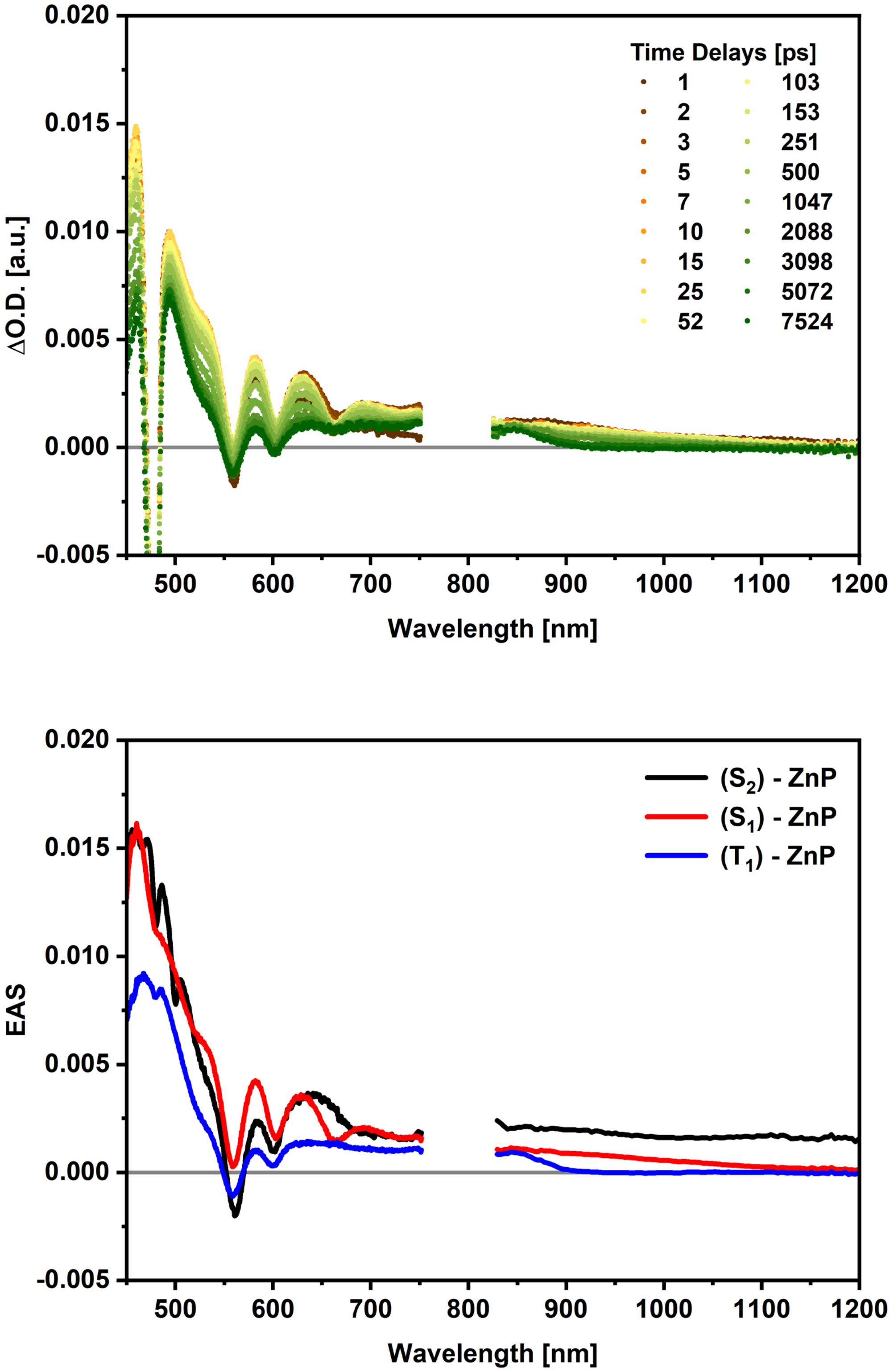

Figure S19: Femtosecond differential absorption spectra of aza[10]CPP-rotaxane **2** (2.5 x 10^{-6} M) in argon purged PhCN at time delays between 1 and 7525 ps after 430 nm laser excitation. Deconvoluted evolution-associated spectra (EAS) obtained via global analysis are shown beneath, following a sequential deactivation model (black-red-blue). Scattering at 480 nm was corrected by subtracting the background.

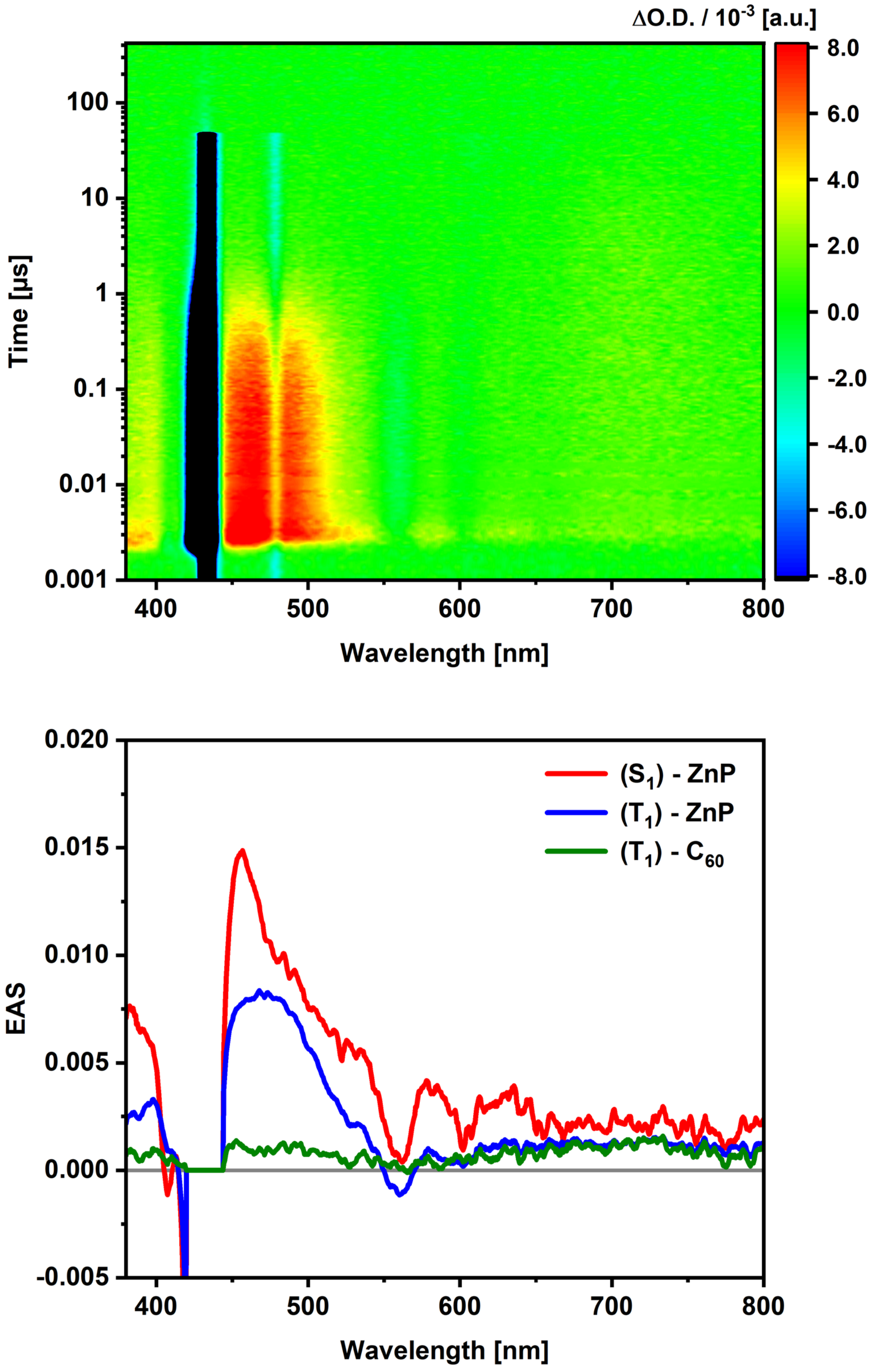

Figure S20: Nanosecond differential absorption spectra of aza[10]CPP-rotaxane **2** (2.5×10^{-6} M) in argon purged PhCN at time delays between 1 ns and 440 µs after 430 nm laser excitation. Deconvoluted evolution-associated spectra (EAS) obtained via global analysis are shown beneath, following a sequential deactivation model (red-blue-green). Scattering at 480 nm was corrected by subtracting the background.

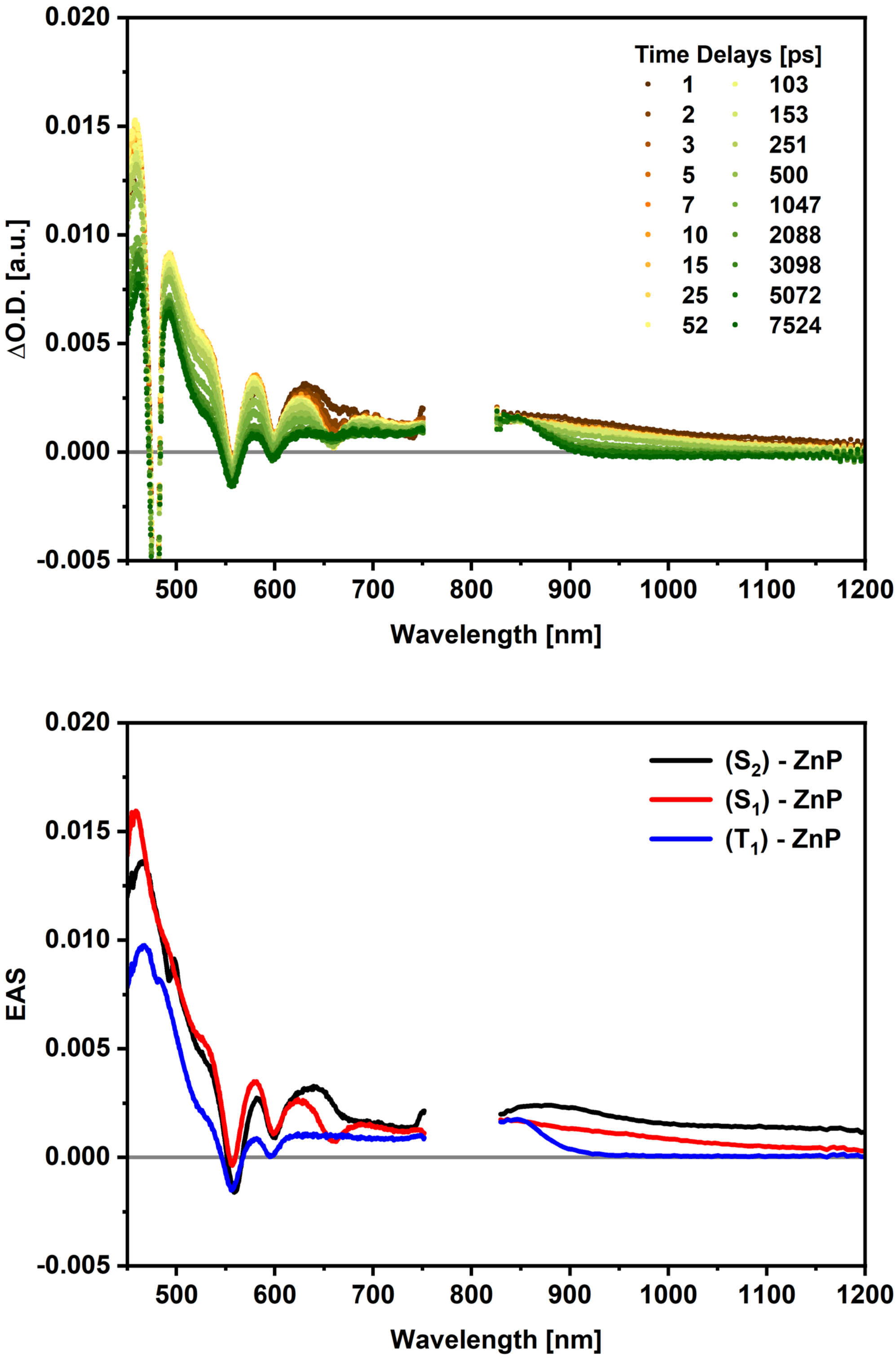

Figure S21: Femtosecond differential absorption spectra of aza[10]CPP-rotaxane 2 (2.5 x 10-6 M) in argon purged THF at time delays between 1 and 7525 ps after 430 nm laser excitation. Deconvoluted evolution-associated spectra (EAS) obtained via global analysis are shown beneath, following a sequential deactivation model (black-red-blue). Scattering at 480 nm was corrected by subtracting the background.

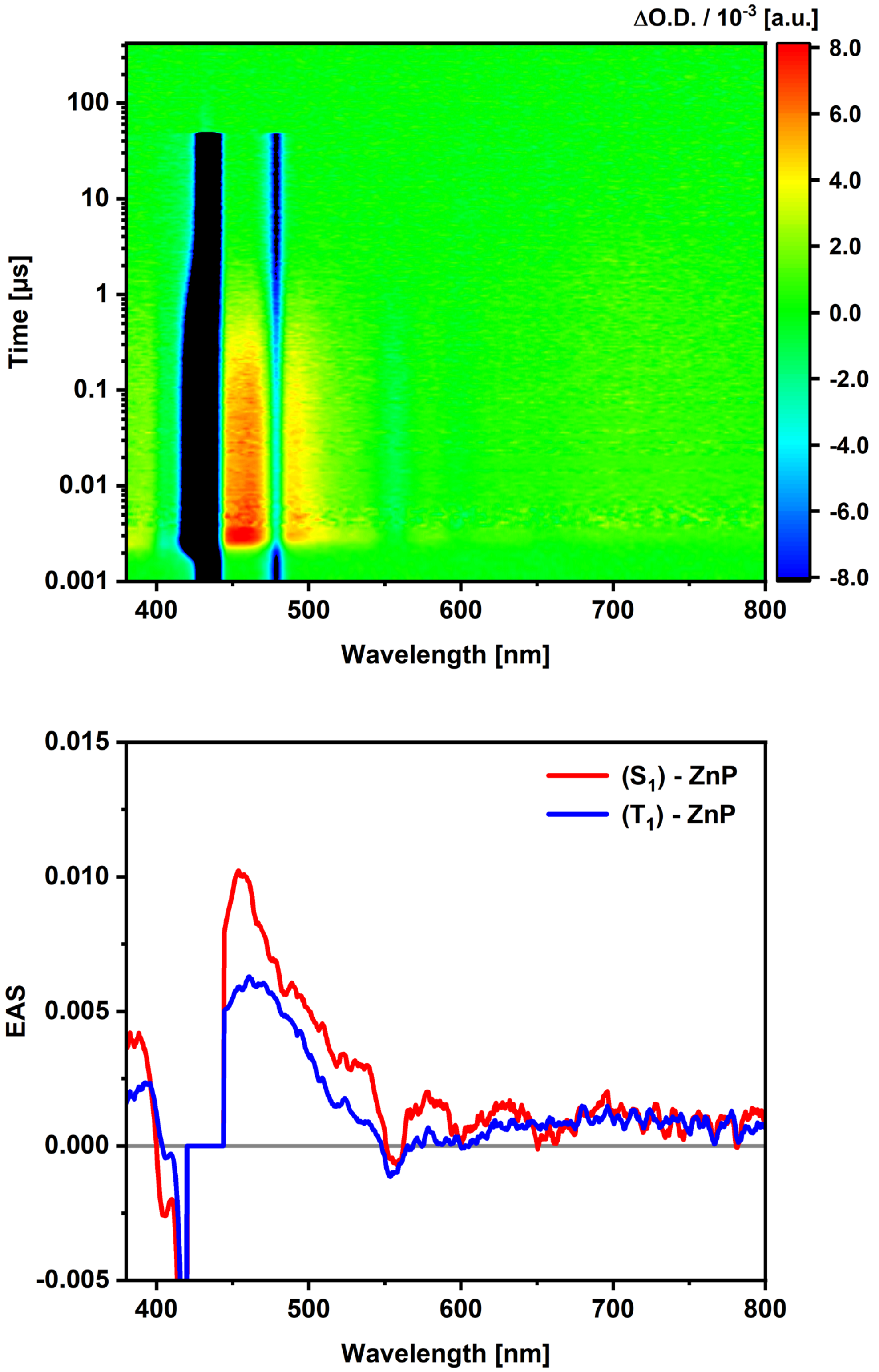

Figure S22: Nanosecond differential absorption spectra of aza[10]CPP-rotaxane **2** (2.5×10^{-6} M) in argon purged THF at time delays between 1 ns and 440 µs after 430 nm laser excitation. Deconvoluted evolution-associated spectra (EAS) obtained via global analysis are shown beneath, following a sequential deactivation model (red-blue). Scattering at 480 nm was corrected by subtracting the background.

Table S1: Summary of the different lifetimes obtained via global analysis from transient absorption spectroscopy measurements of the porphyrin **12** reference (5 x 10^{-6} M) in argon purged PhCN after 430 nm laser excitation. Mixtures of porphyrin **12**, *trans*-3-diethyl malonate C_{60} bis-adduct, and [10]CPP or aza[10]CPP in different molar ratios.

	Porphyrin **12**<br>1:0	+ C_{60} bis-adduct<br>1:1	+ C_{60} bis-adduct<br>1:10	+ C_{60} bis-adduct<br>+ [10]CPP<br>1:1:1	+ C_{60} bis-adduct<br>+ [10]CPP<br>1:10:10	+ C_{60} bis-adduct<br>+ aza[10]CPP<br>1:1:1	+ C_{60} bis-adduct<br>+ aza[10]CPP<br>1:10:10
(S_2)	0.9 ps	1.8 ps	2.6 ps	1.7 ps	3.1 ps	1.6 ps	2.0 ps
(S_1)	2.2 ns	2.2 ns	2.1 ns	2.1 ns	2.6 ns	2.2 ns	2.2 ns
(T_1)	> 400 µs	92 µs	13 µs	56 µs	28 µs	56 µs	16 µs
CSS	not observed	outside of time range	outside of time range	outside of time range	outside of time range	outside of time range	outside of time range

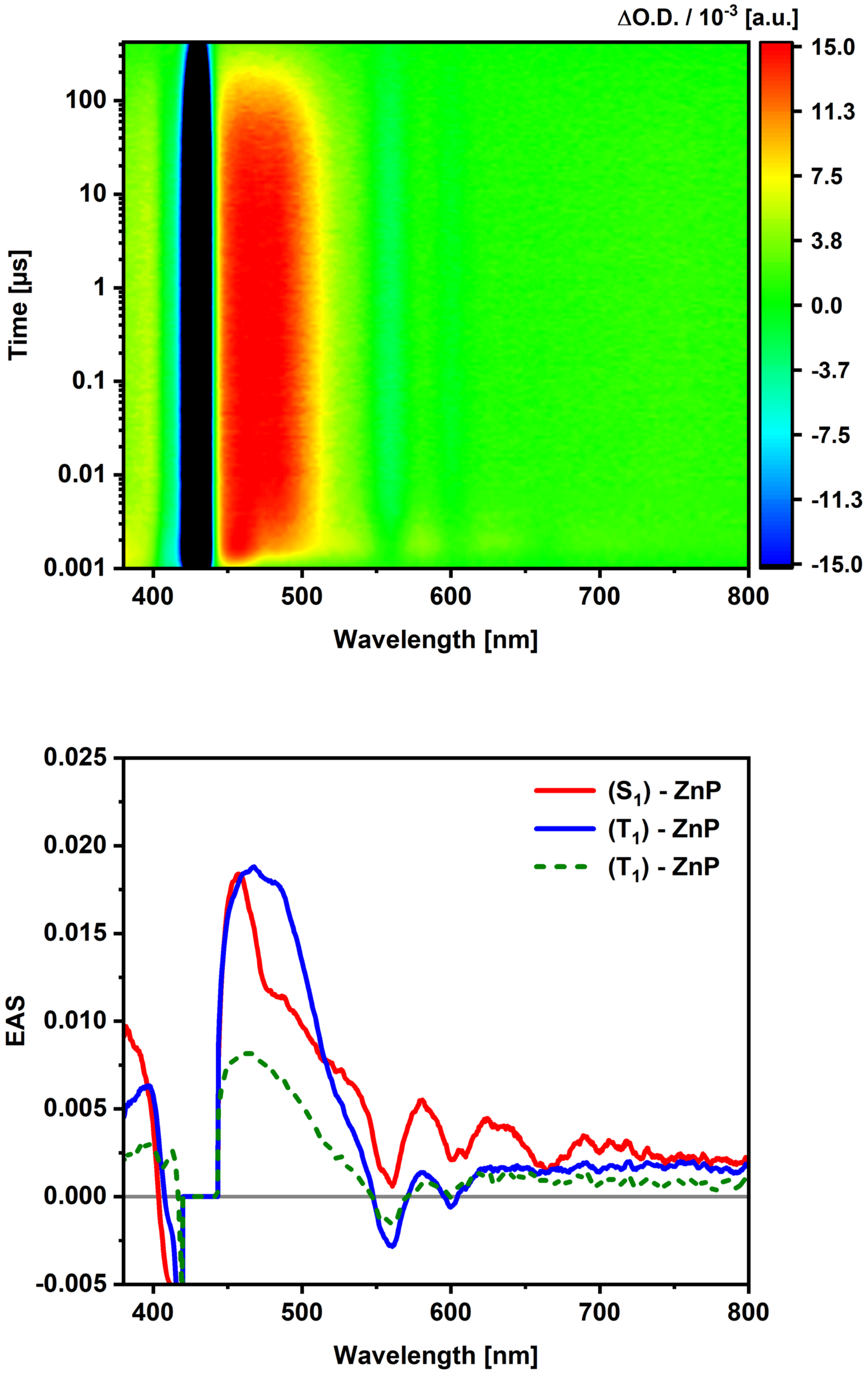

Figure S23: Nanosecond differential absorption spectra of porphyrin **12** (5 x 10^{-6} M) in argon purged PhCN at time delays between 1 ns and 440 µs after 430 nm laser excitation. Deconvoluted evolution-associated spectra (EAS) obtained via global analysis are shown beneath, following a sequential deactivation model (red-blue-green). Lifetime of the (T_1) lies outside of the measurement window (>400 µs), dotted green line shows last time delays.

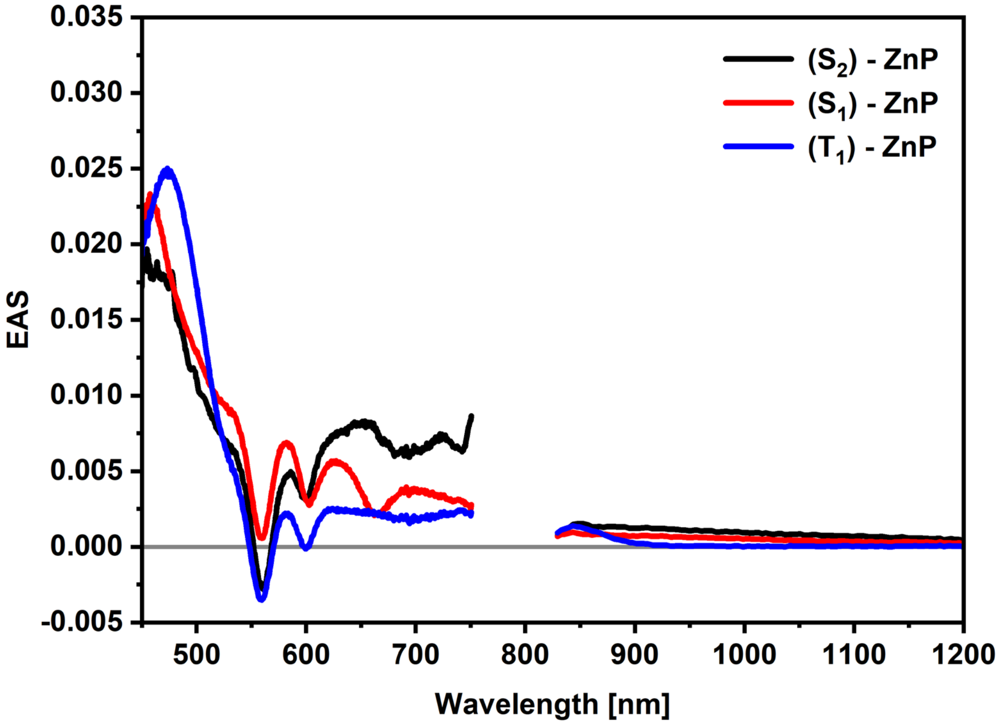

Figure S24: Deconvoluted evolution-associated spectra (EAS) obtained via global analysis of femtosecond differential absorption spectra of porphyrin **12** (5 x 10^{-6} M) in argon purged PhCN at time delays between 1 and 7525 ps after 430 nm laser excitation. Following a sequential deactivation model (black-red-blue).

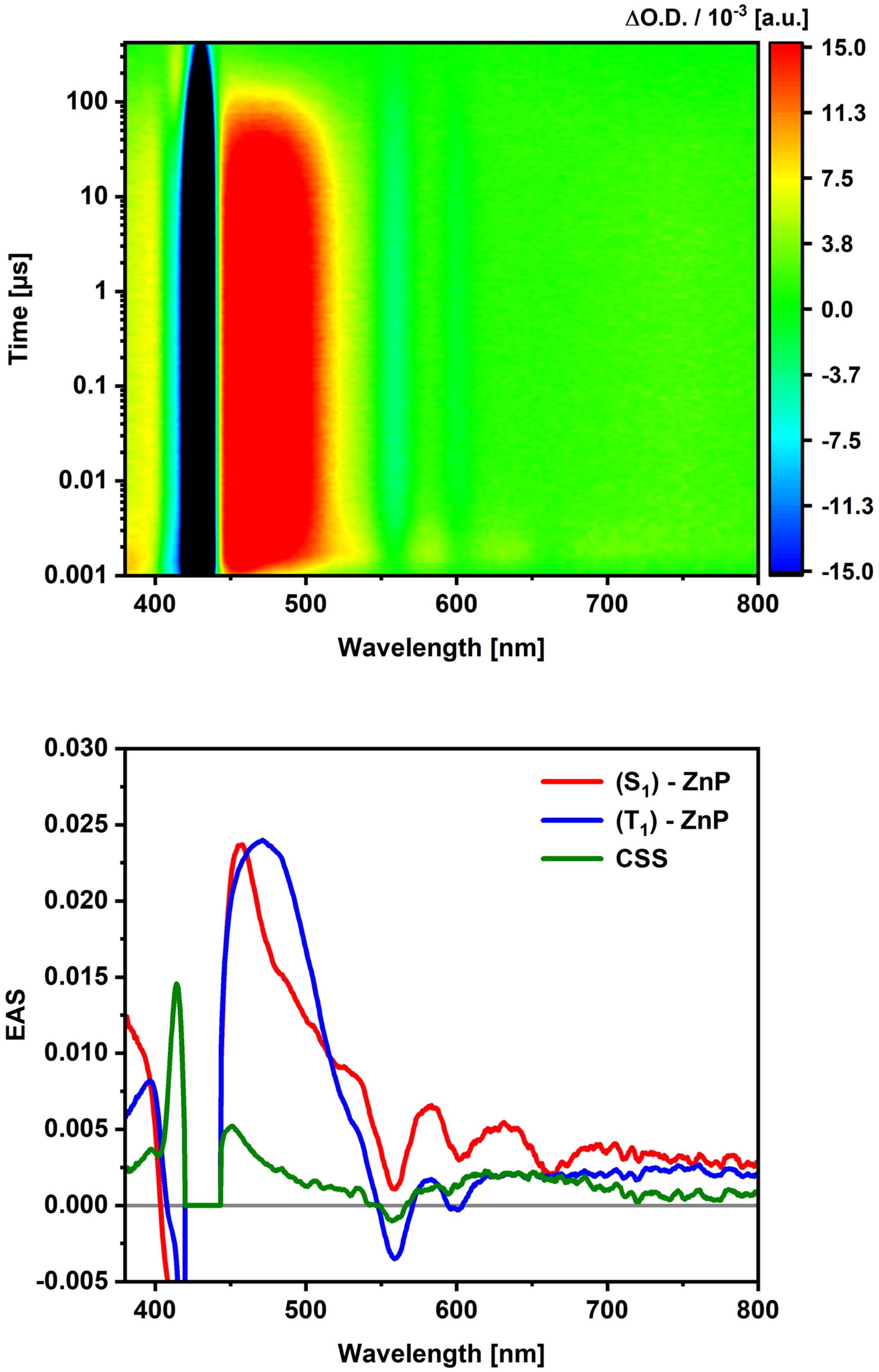

Figure S25: Nanosecond differential absorption spectra of a mixture of porphyrin **12** (5 x 10^{-6} M) and *trans*-3-diethyl malonate C_{60} bis-adduct in a 1:1 molar ratio in argon purged PhCN at time delays between 1 ns and 440 µs after 430 nm laser excitation. Deconvoluted evolution-associated spectra (EAS) obtained via global analysis are shown beneath, following a sequential deactivation model (red-blue-green). Lifetime of the CSS lies outside of the measurement window.

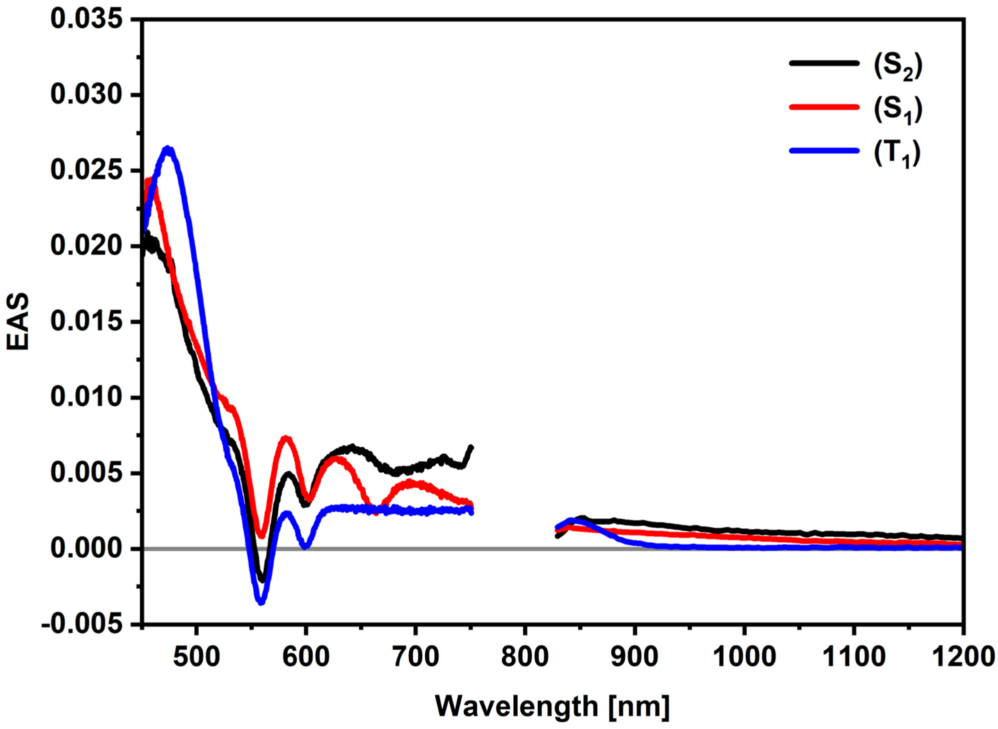

Figure S26: Deconvoluted evolution-associated spectra (EAS) obtained via global analysis of femtosecond differential absorption spectra of a mixture of porphyrin **12** (5×10^{-6} M) and *trans*-3-diethyl malonate C_{60} bis-adduct in a 1:1 molar ratio in argon purged PhCN at time delays between 1 and 7525 ps after 430 nm laser excitation. Following a sequential deactivation model (black-red-blue).

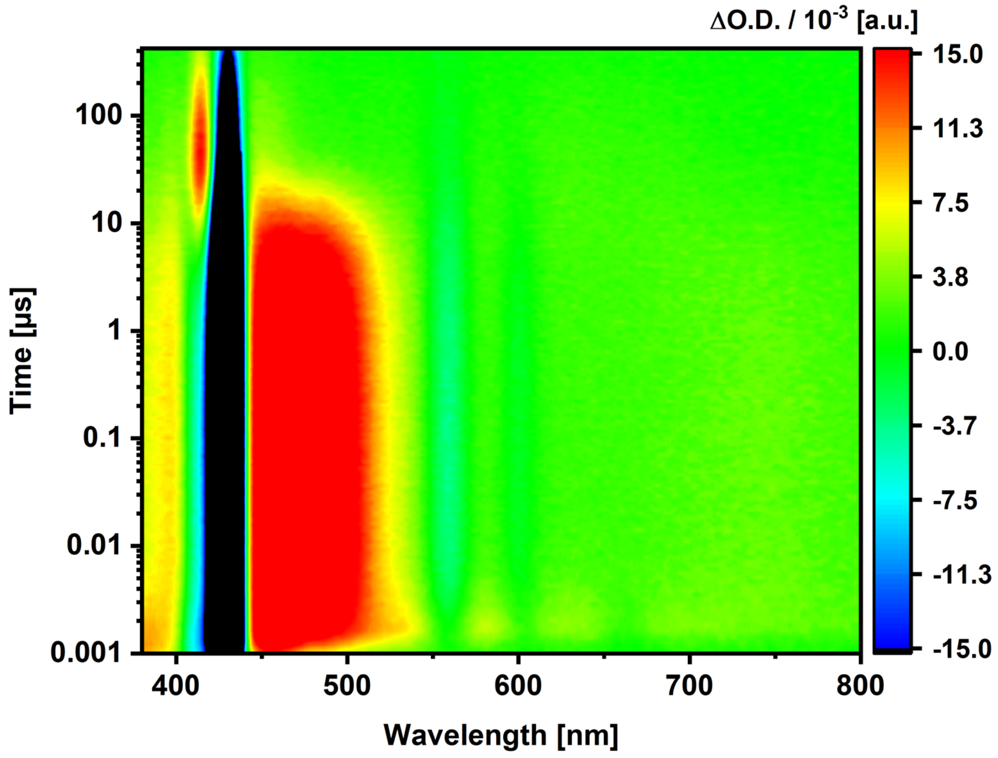

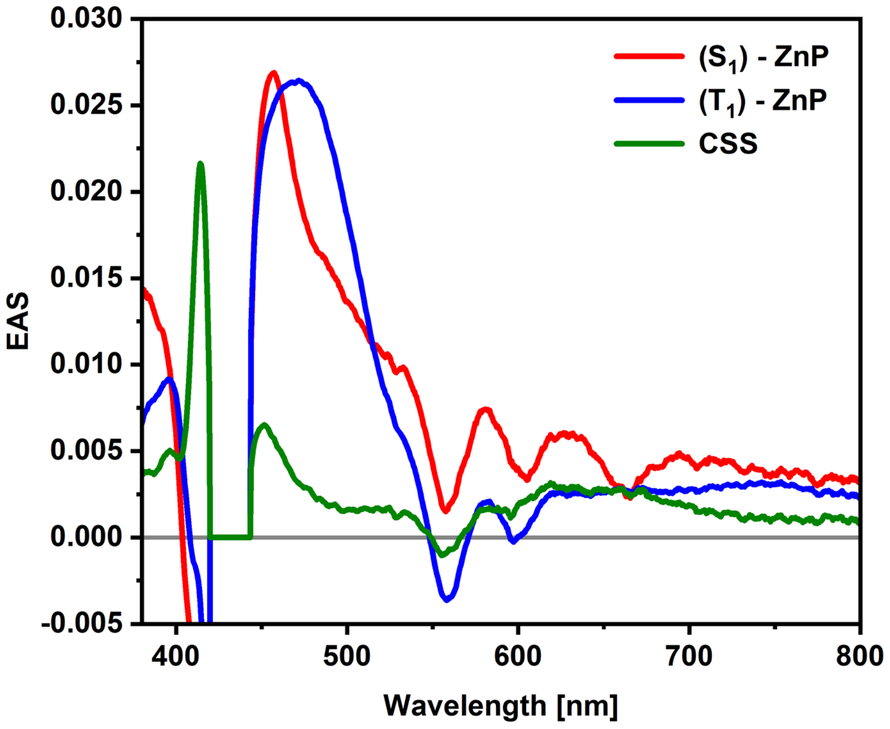

Figure S27: Nanosecond differential absorption spectra of a mixture of porphyrin **12** (5 x 10^{-6} M) and *trans*-3-diethyl malonate C_{60} bis-adduct in a 1:10 molar ratio in argon purged PhCN at time delays between 1 ns and 440 µs after 430 nm laser excitation. Deconvoluted evolution-associated spectra (EAS) obtained via global analysis are shown beneath, following a sequential deactivation model (red-blue-green). Lifetime of the CSS lies outside of the measurement window

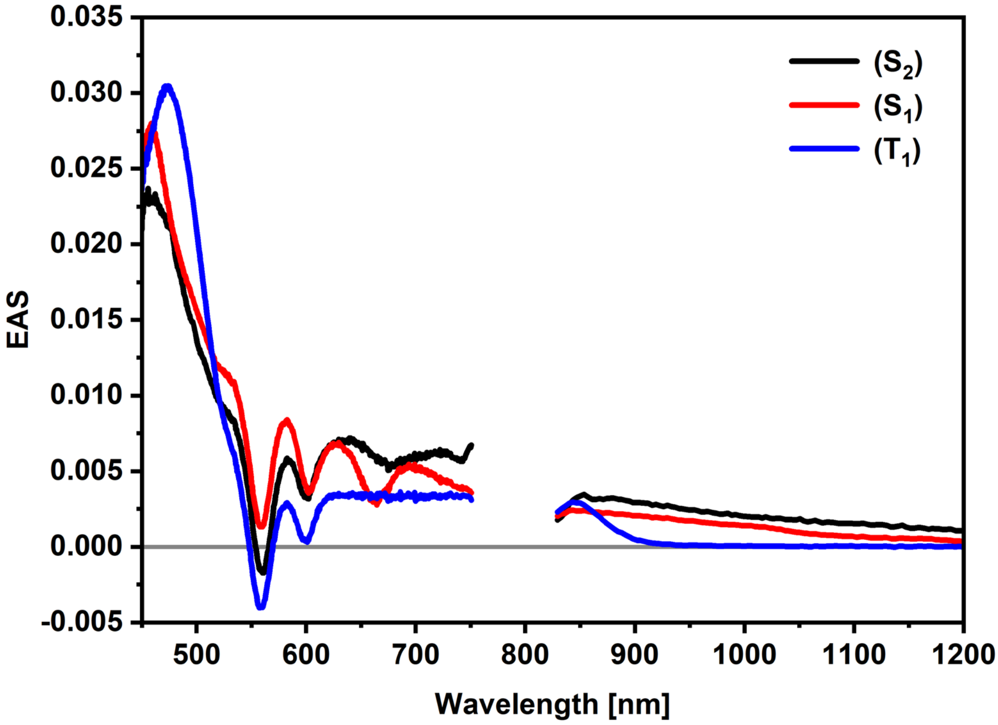

Figure S28: Deconvoluted evolution-associated spectra (EAS) obtained via global analysis of femtosecond differential absorption spectra of a mixture of porphyrin **12** (5×10^{-6} M) and *trans*-3-diethyl malonate C_{60} bis-adduct in a 1:10 molar ratio in argon purged PhCN at time delays between 1 and 7525 ps after 430 nm laser excitation. Following a sequential deactivation model (black-red-blue).

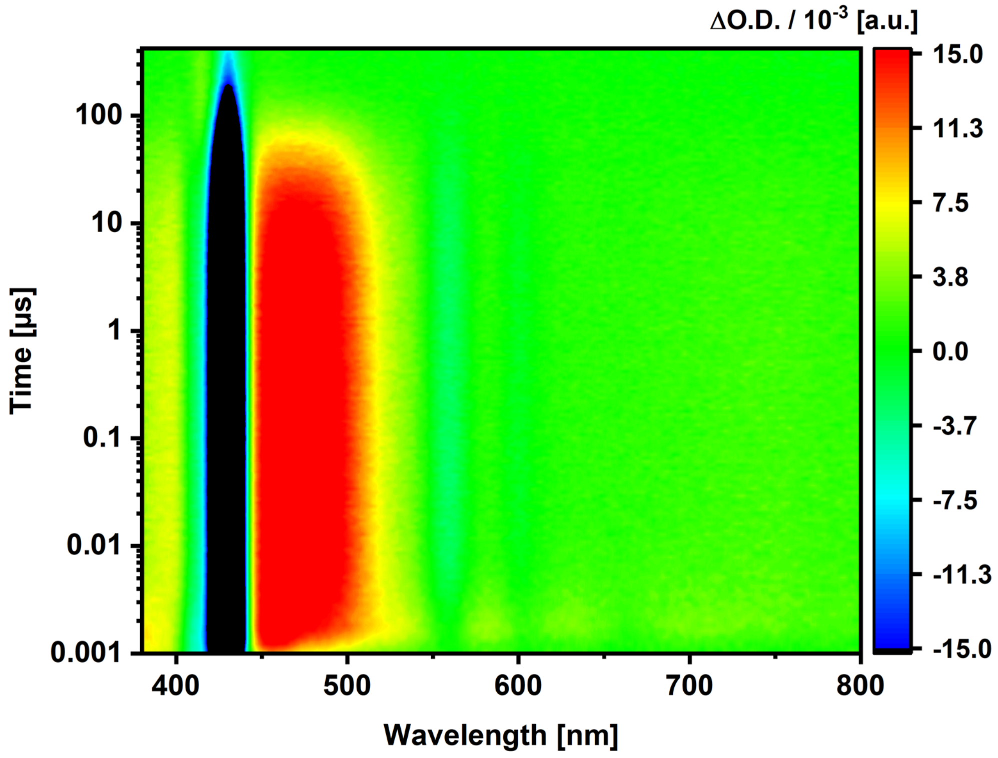

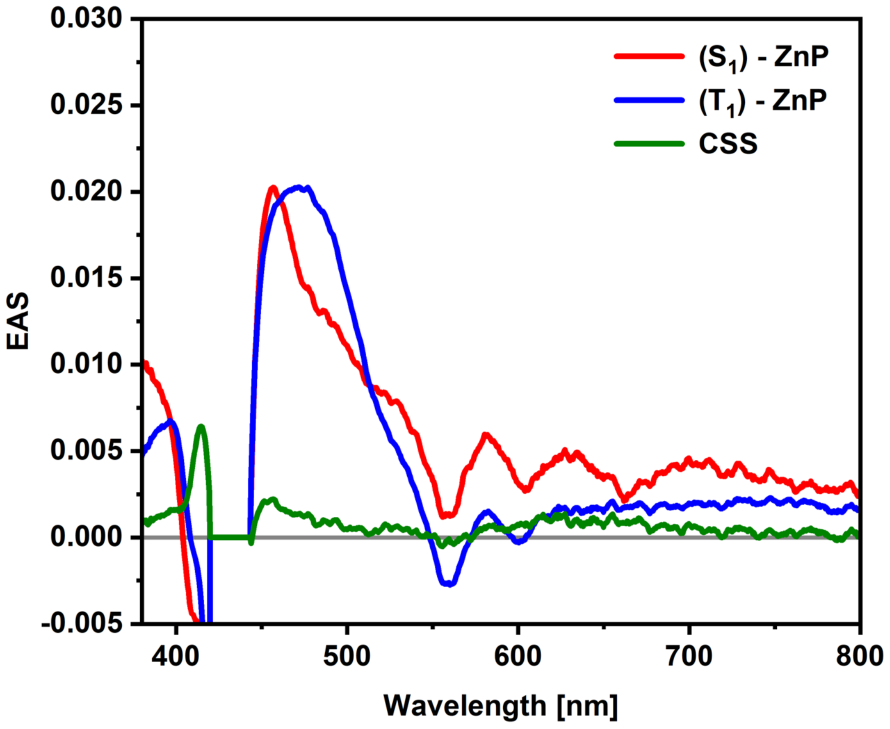

Figure S29: Nanosecond differential absorption spectra of a mixture of porphyrin **12** (5×10^{-6} M), *trans*-3-diethyl malonate C_{60} bis-adduct, and [10]CPP in a 1:1:1 molar ratio in argon purged PhCN at time delays between 1 ns and 440 µs after 430 nm laser excitation. Deconvoluted evolution-associated spectra (EAS) obtained via global analysis are shown beneath, following a sequential deactivation model (red-blue-green). Lifetime of the CSS lies outside of the measurement window.

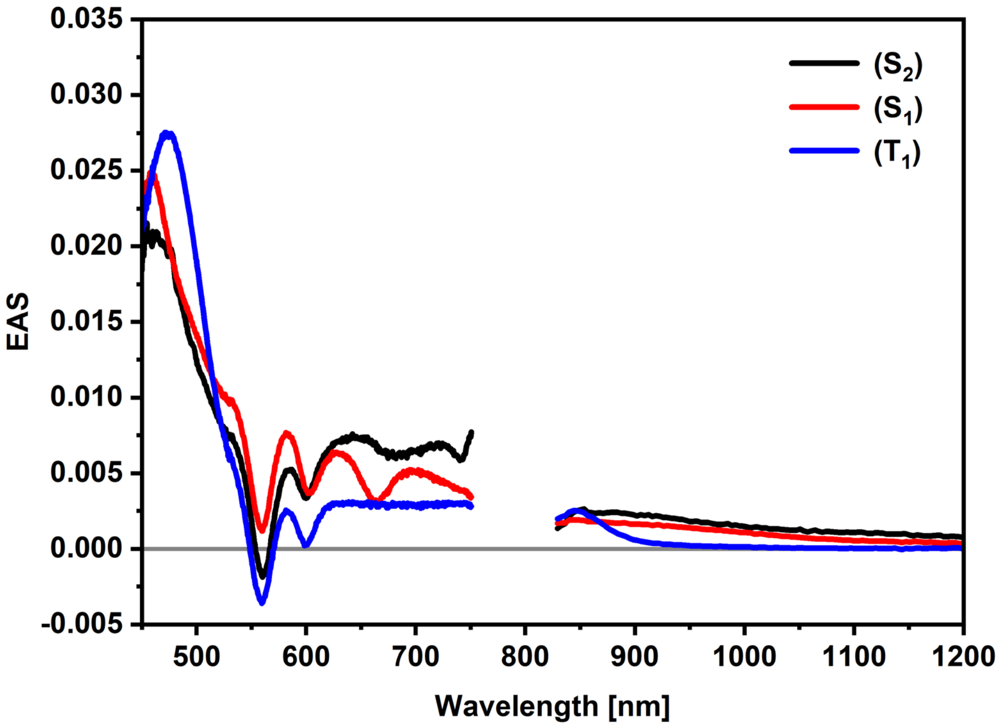

Figure S30: Deconvoluted evolution-associated spectra (EAS) obtained via global analysis of femtosecond differential absorption spectra of a mixture of porphyrin **12** (5 x 10^{-6} M), *trans*-3-diethyl malonate C_{60} bis-adduct, and [10]CPP in a 1:1:1 molar ratio in argon purged PhCN at time delays between 1 and 7525 ps after 430 nm laser excitation. Following a sequential deactivation model (black-red-blue).

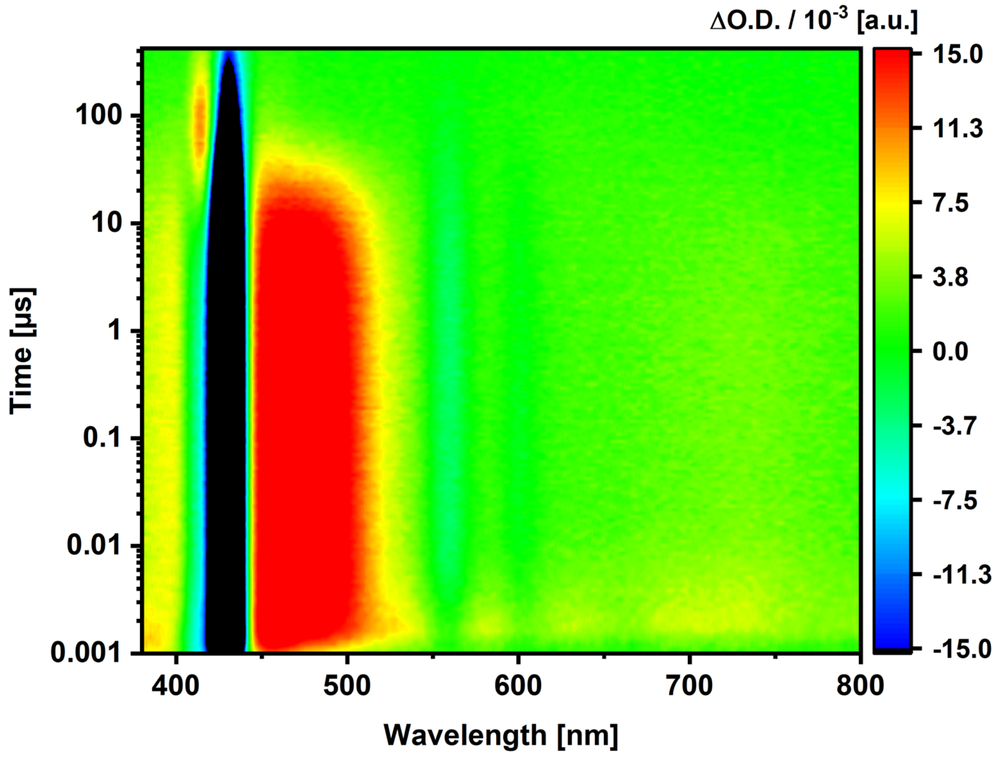

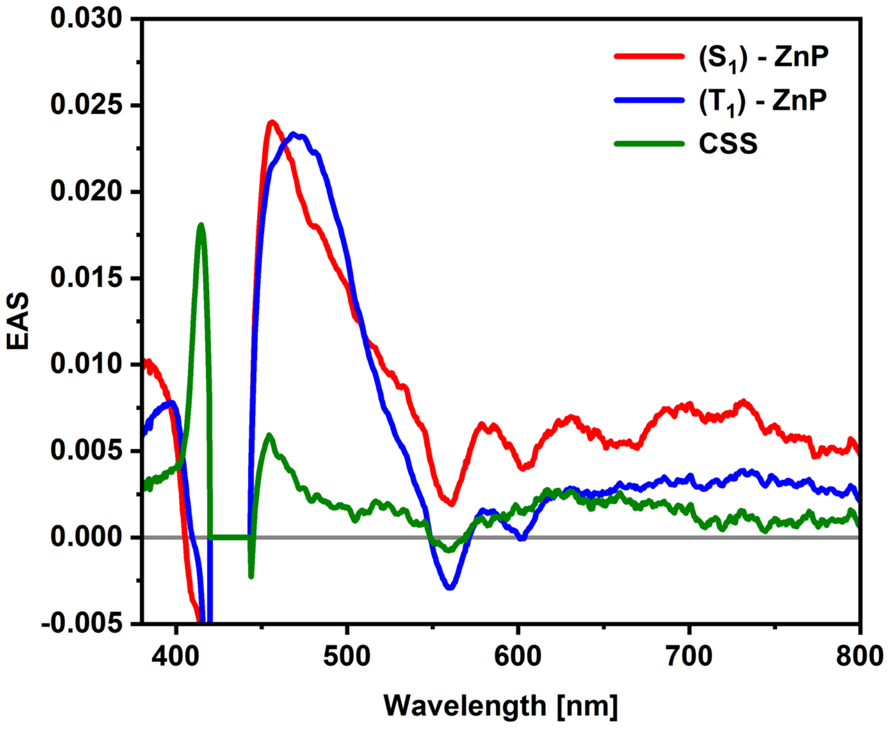

Figure S31: Nanosecond differential absorption spectra of a mixture of porphyrin **12** (5 x 10^{-6} M), *trans*-3-diethyl malonate C_{60} bis-adduct, and [10]CPP in a 1:10:10 molar ratio in argon purged PhCN at time delays between 1 ns and 440 μs after 430 nm laser excitation. Deconvoluted evolution-associated spectra (EAS) obtained via global analysis are shown beneath, following a sequential deactivation model (red-blue-green). Lifetime of the CSS lies outside of the measurement window.

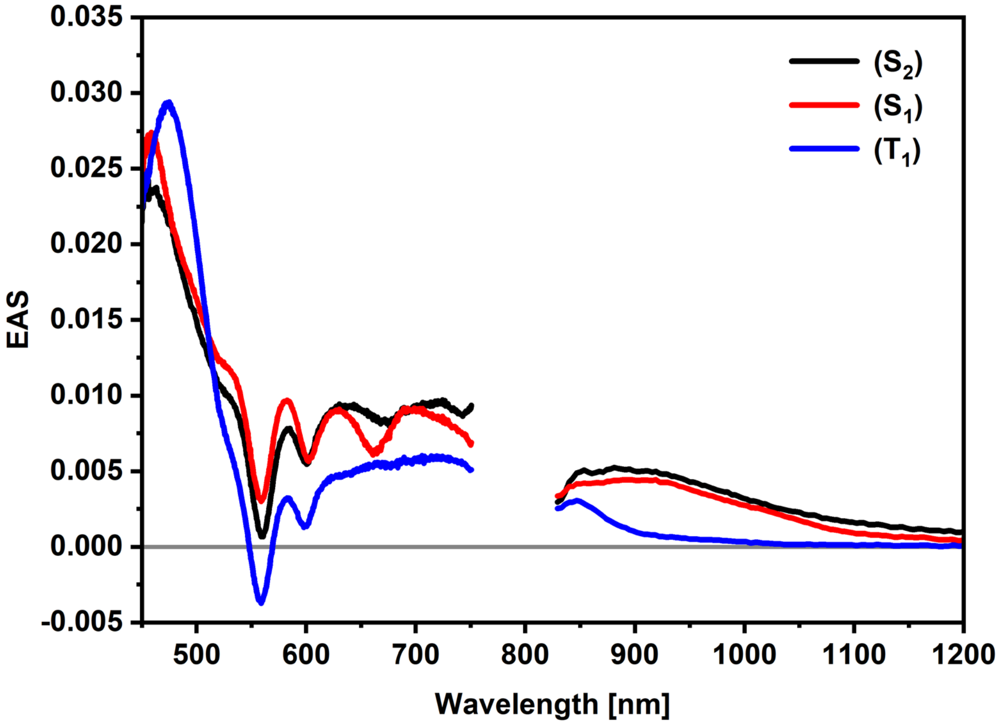

Figure S32: Deconvoluted evolution-associated spectra (EAS) obtained via global analysis of femtosecond differential absorption spectra of a mixture of porphyrin **12** (5 x 10^{-6} M), *trans*-3-diethyl malonate C_{60} bis-adduct, and [10]CPP in a 1:10:10 molar ratio in argon purged PhCN at time delays between 1 and 7525 ps after 430 nm laser excitation. Following a sequential deactivation model (black-red-blue).

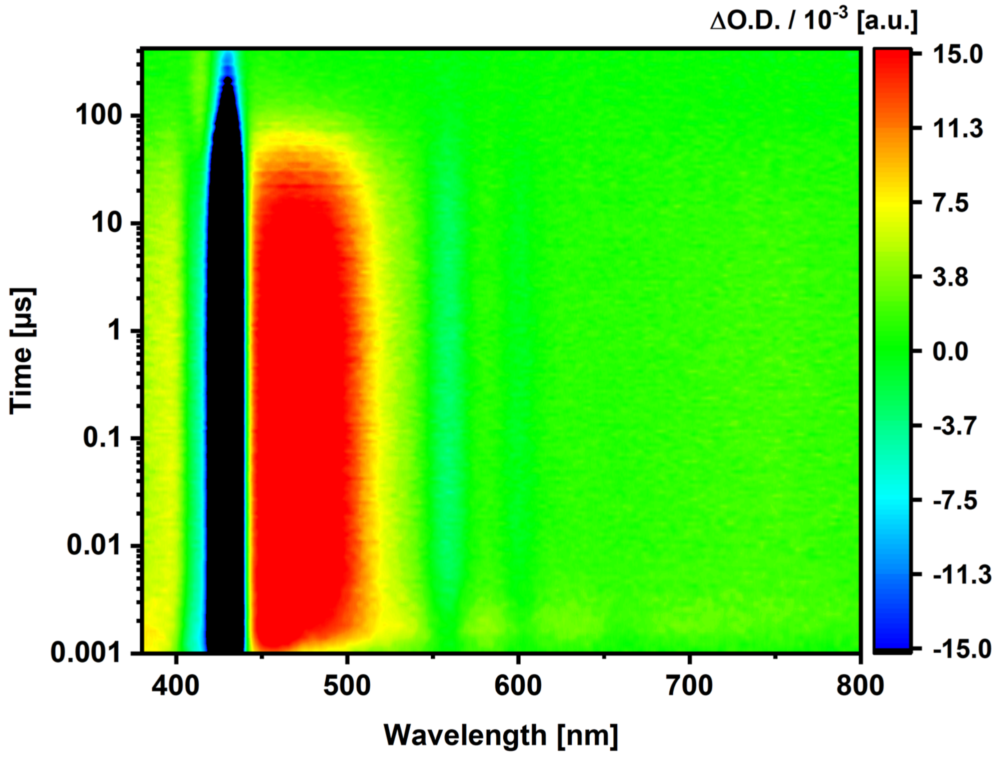

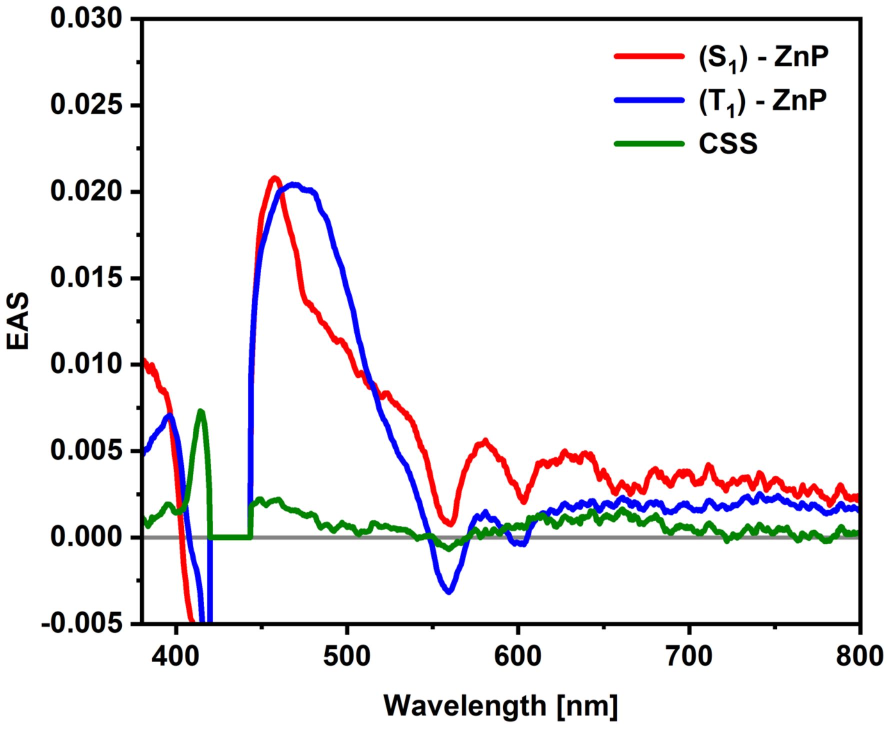

Figure S33: Nanosecond differential absorption spectra of a mixture of porphyrin **12** (5×10^{-6} M), *trans*-3-diethyl malonate C_{60} bis-adduct, and aza[10]CPP in a 1:1:1 molar ratio in argon purged PhCN at time delays between 1 ns and 440 µs after 430 nm laser excitation. Deconvoluted evolution-associated spectra (EAS) obtained via global analysis are shown beneath, following a sequential deactivation model (red-blue-green). Lifetime of the CSS lies outside of the measurement window.

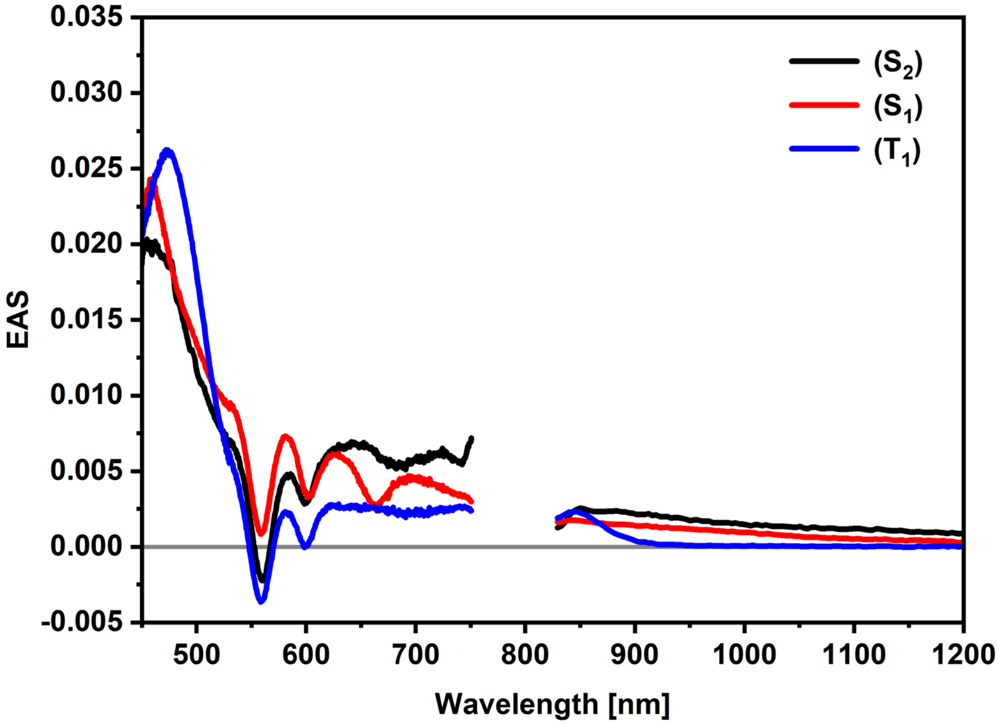

Figure S34: Deconvoluted evolution-associated spectra (EAS) obtained via global analysis of femtosecond differential absorption spectra of a mixture of porphyrin **12** (5×10^{-6} M), *trans*-3-diethyl malonate C_{60} bis-adduct, and aza[10]CPP in a 1:1:1 molar ratio in argon purged PhCN at time delays between 1 and 7525 ps after 430 nm laser excitation. Following a sequential deactivation model (black-red-blue).

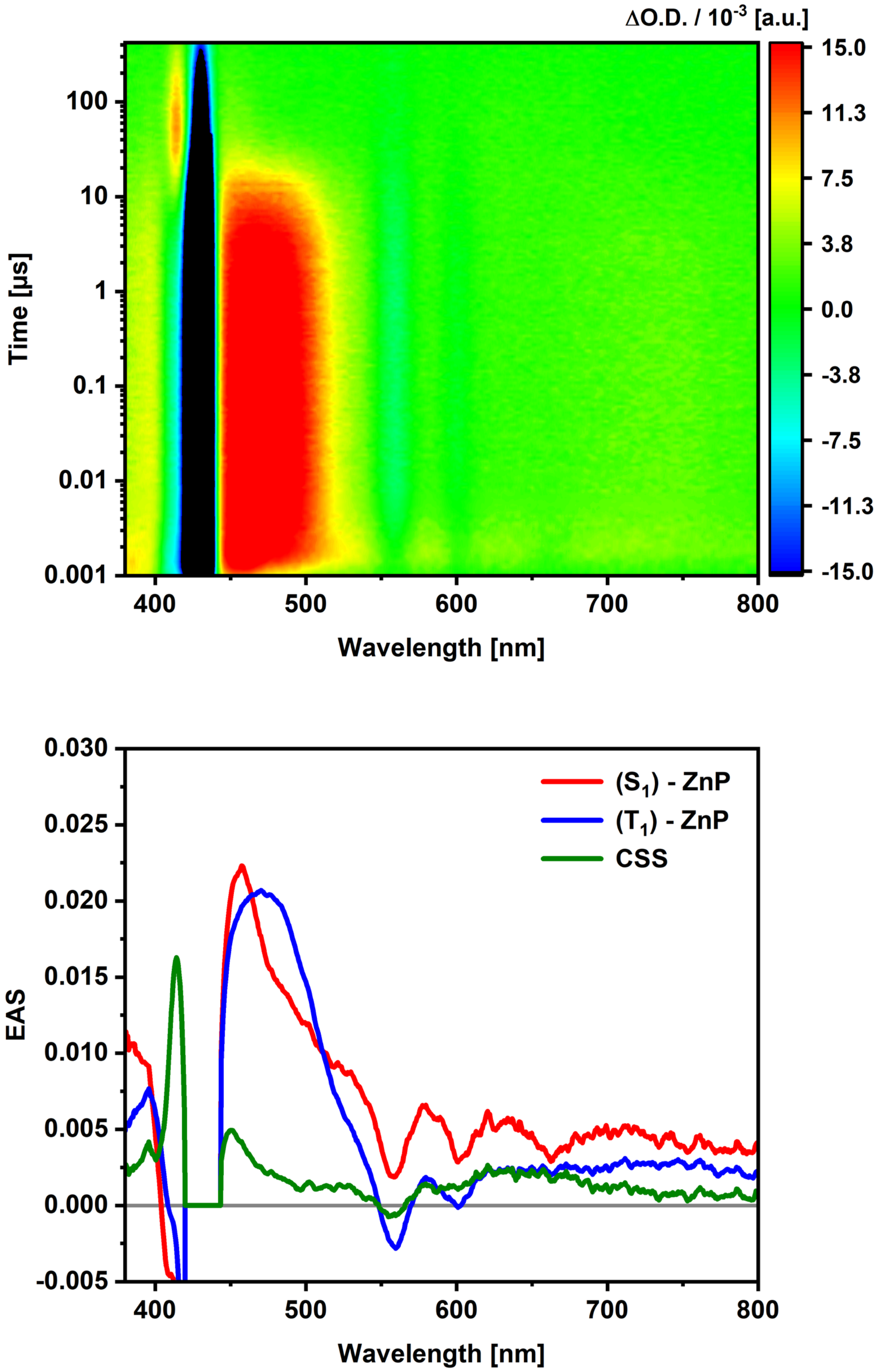

Figure S35: Nanosecond differential absorption spectra of a mixture of porphyrin **12** (5 x 10^{-6} M), *trans*-3-diethyl malonate C_{60} bis-adduct, and aza[10]CPP in a 1:10:10 molar ratio in argon purged PhCN at time delays between 1 ns and 440 µs after 430 nm laser excitation. Deconvoluted evolution-associated spectra (EAS) obtained via global analysis are shown beneath, following a sequential deactivation model (red-blue-green). Lifetime of the CSS lies outside of the measurement window.

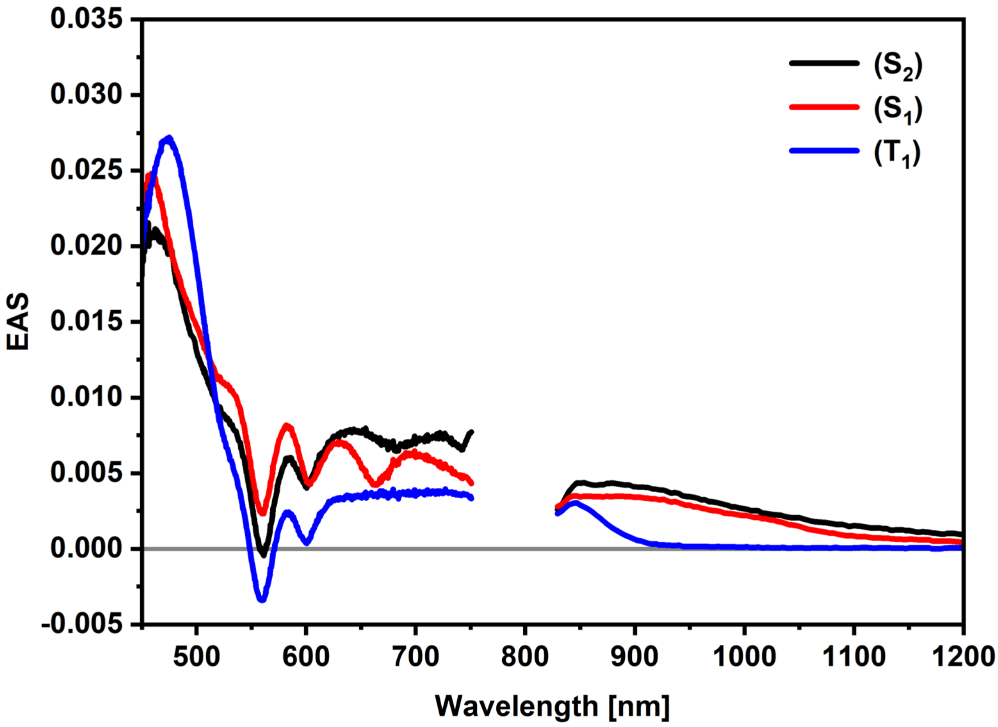

Figure S36: Deconvoluted evolution-associated spectra (EAS) obtained via global analysis of femtosecond differential absorption spectra of a mixture of porphyrin **12** (5×10^{-6} M), *trans*-3-diethyl malonate C_{60} bis-adduct, and aza[10]CPP in a 1:10:10 molar ratio in argon purged PhCN at time delays between 1 and 7525 ps after 430 nm laser excitation. Following a sequential deactivation model (black-red-blue).

Temperature-dependent TAS Measurements

Table S2: Summary of the different lifetimes obtained via global analysis from transient absorption spectroscopy measurements of the thread as well as the rotaxanes (2.5×10^{-6} M) in argon purged PhCN after 430 nm laser excitation. Measured at 80 °C without stirring.

	thread **3**	[10]CPP-rotaxane **1**	aza[10]CPP-rotaxane **2**
(S_2) - ZnP	not measured	2.1 ps	0.9 ps
(S_1) - ZnP	not measured	1.3 ns	1.0 ns
(T_1) - ZnP	< 0.1μs	0.1 μs	0.1 μs
CSS	0.7 μs	not observed	not observed
(T_1) - C_{60}	not observed	13 μs	17 μs

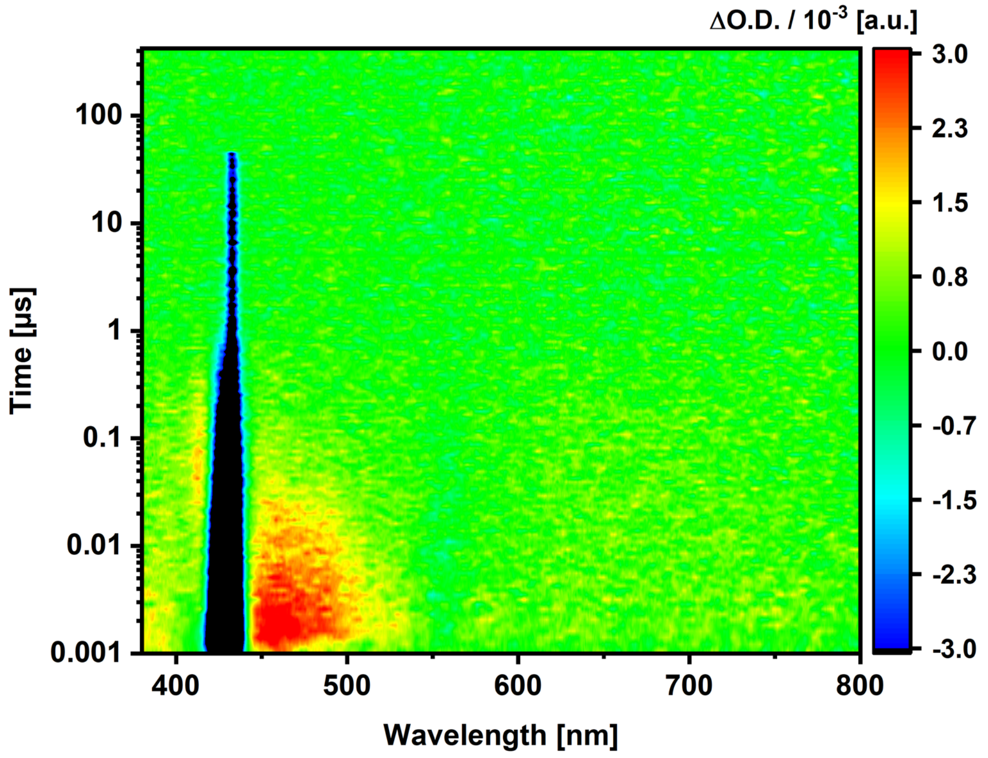

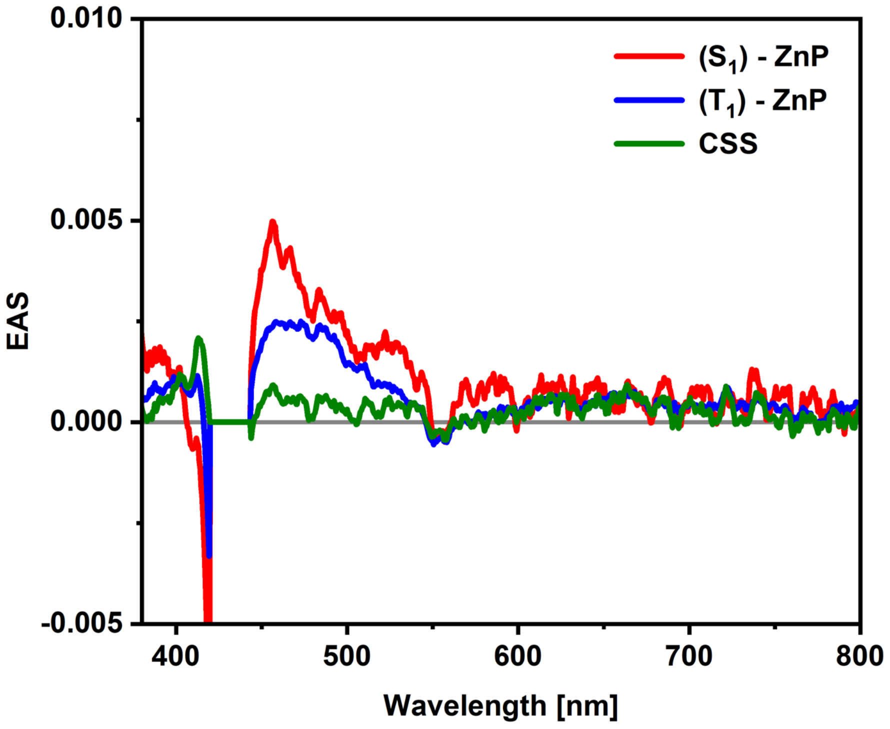

Figure S37: Nanosecond differential absorption spectra of thread **3** (2.5 x 10^{-6} M) in argon purged PhCN at time delays between 1 ns and 440 µs after 430 nm laser excitation. Deconvoluted evolution-associated spectra (EAS) obtained via global analysis are shown beneath, following a sequential deactivation model (red-blue-green). Measured at 80 °C without stirring.

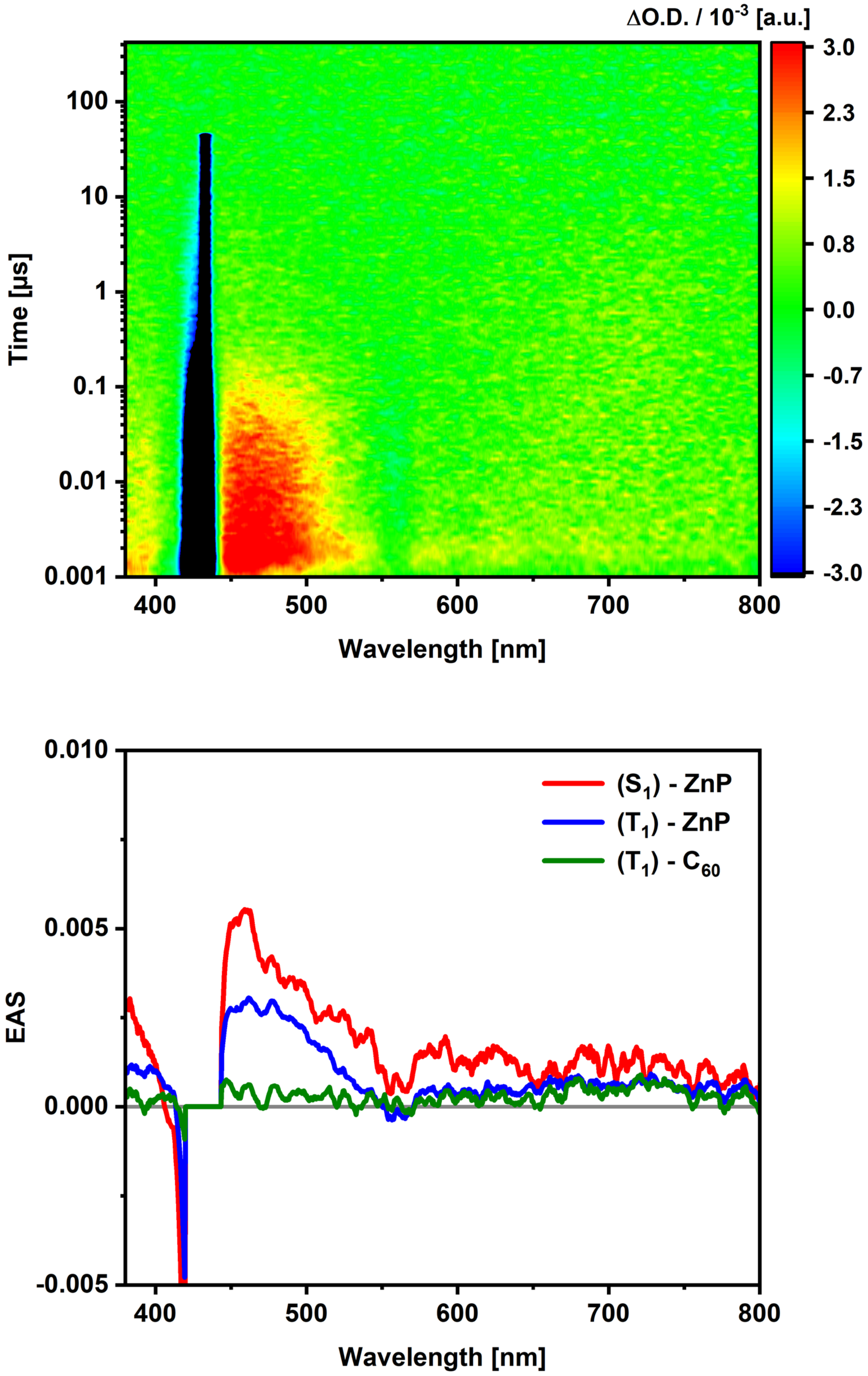

Figure S38: Nanosecond differential absorption spectra of [10]CPP-rotaxane **1** (2.5 x 10^{-6} M) in argon purged PhCN at time delays between 1 ns and 440 µs after 430 nm laser excitation. Deconvoluted evolution-associated spectra (EAS) obtained via global analysis are shown beneath, following a sequential deactivation model (red-blue-green). Measured at 80 °C without stirring.

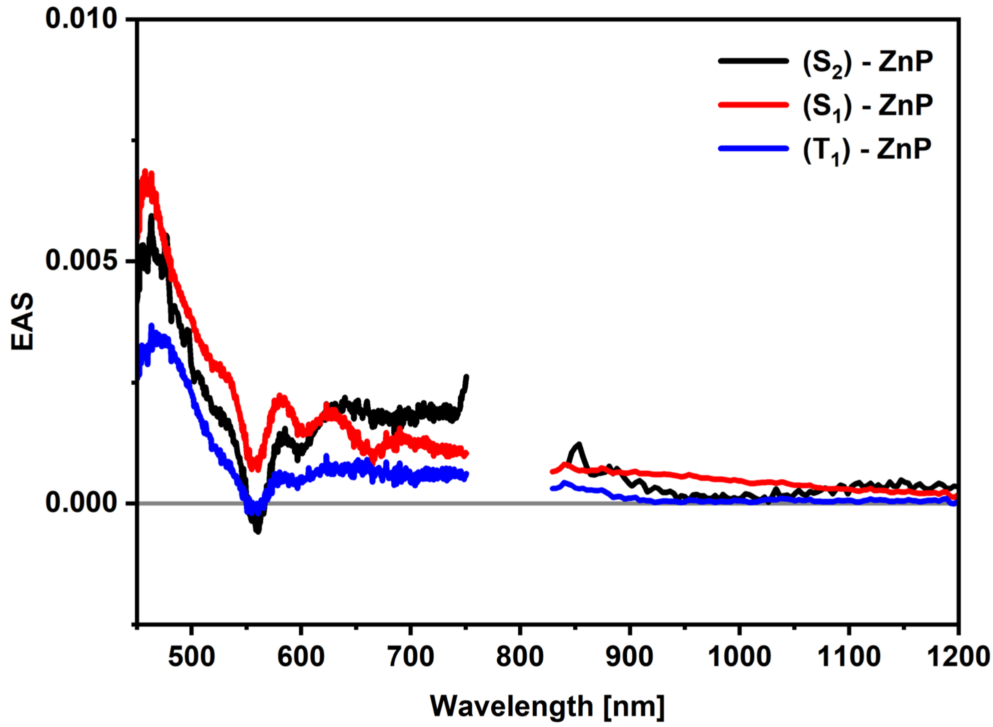

Figure S39: Deconvoluted evolution-associated spectra (EAS) obtained via global analysis of femtosecond differential absorption spectra of [10]CPP-rotaxane **1** (2.5 x 10^{-6} M) in argon purged PhCN at time delays between 1 and 7525 ps after 430 nm laser excitation. Following a sequential deactivation model (black-red-blue). Measured at 80 °C without stirring.

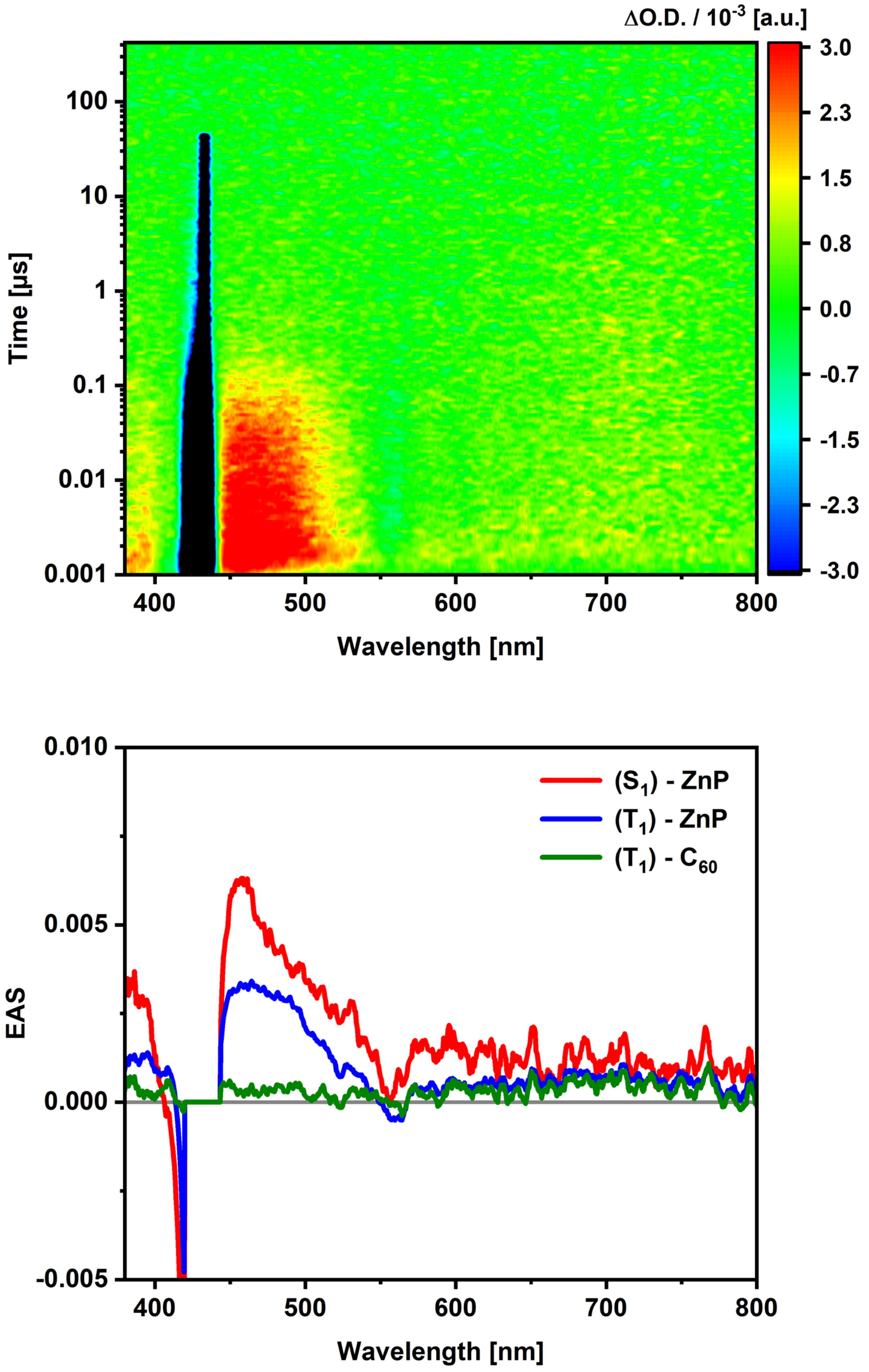

Figure S40: Nanosecond differential absorption spectra of aza[10]CPP-rotaxane **2** (2.5×10^{-6} M) in argon purged PhCN at time delays between 1 ns and 440 µs after 430 nm laser excitation. Deconvoluted evolution-associated spectra (EAS) obtained via global analysis are shown beneath, following a sequential deactivation model (red-blue-green). Measured at 80 °C without stirring.

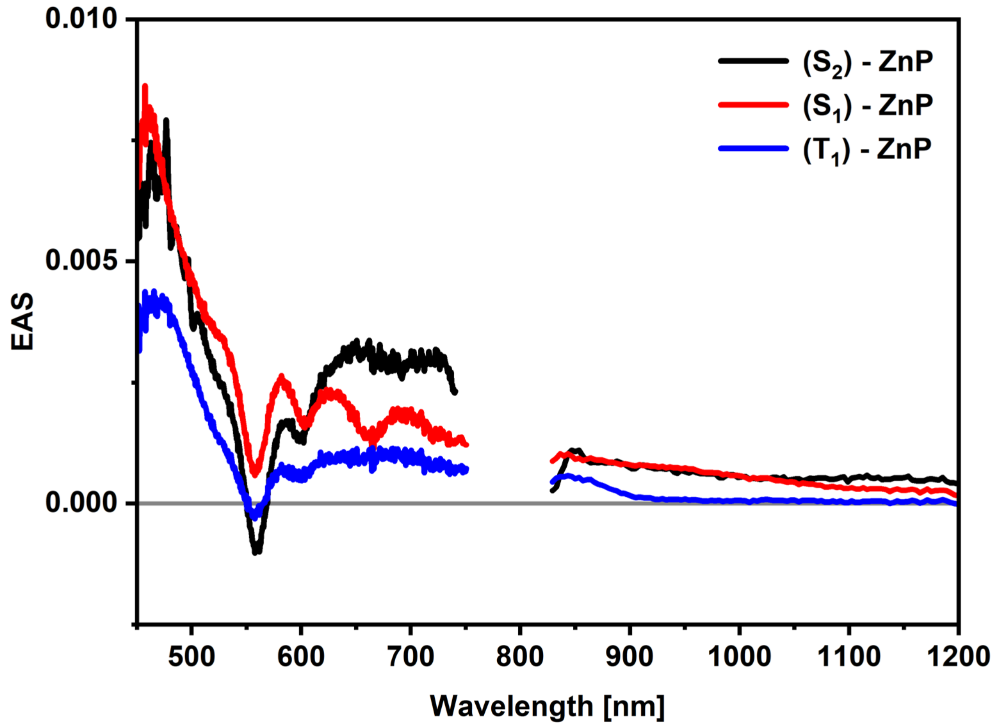

Figure S41: Deconvoluted evolution-associated spectra (EAS) obtained via global analysis of femtosecond differential absorption spectra of aza[10]CPP-rotaxane **2** (2.5 x 10^{-6} M) in argon purged PhCN at time delays between 1 and 7525 ps after 430 nm laser excitation. Following a sequential deactivation model (black-red-blue). Measured at 80 °C without stirring.

To simulate the ratio of free and [10]CPP-complexed *trans-3* diethyl malonate C_{60} bisadduct under the conditions used for the TAS measurements the tool "*bindsim*" from *supramol.org* was used. Using a conservative assumption for the binding constant of K_A = 1.7 10^4 M^{-1} (determined in toluene)[14], the concentration of 2.5 10^{-6} M and the 1:1 stoichiometry of the TAS experiments, the simulation determines a ratio of approximately 4:96 between the complexed and uncomplexed fullerene.

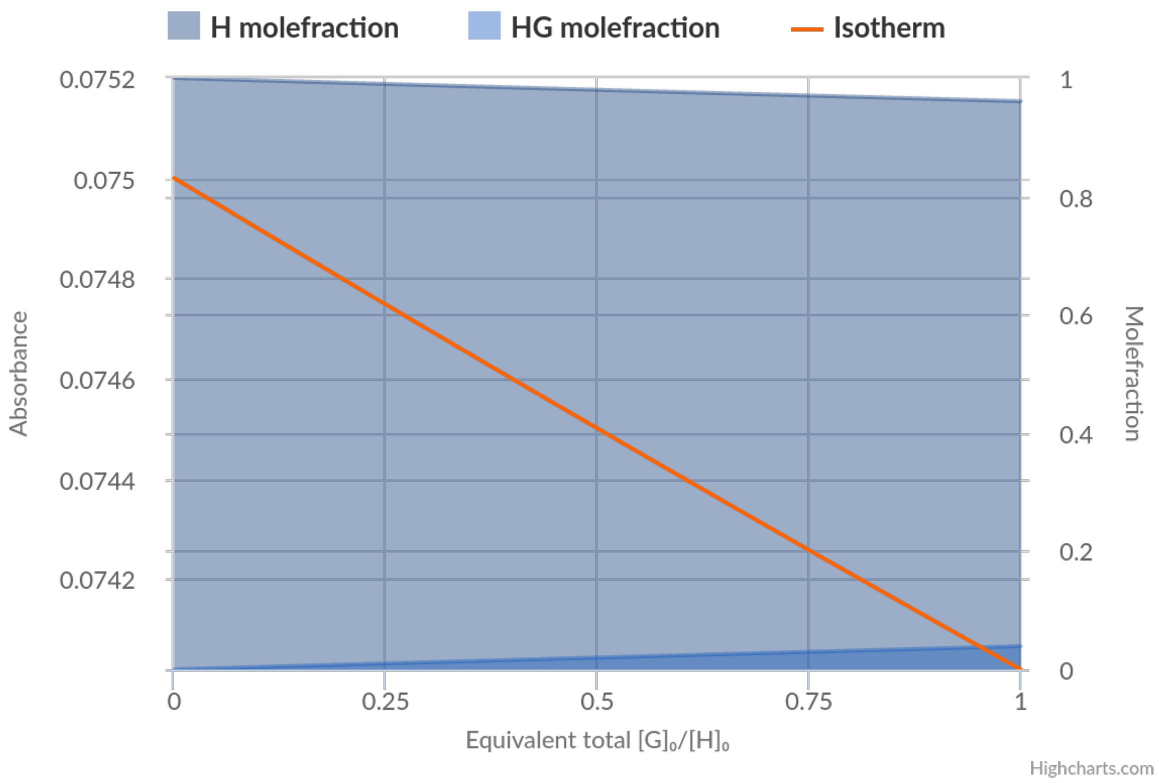

Figure S42: *Bindsim* simulation[15, 16] for the the ratio of free and [10]CPP complexed *trans-3* diethyl malonate C_{60} bisadduct.

8. Spectra

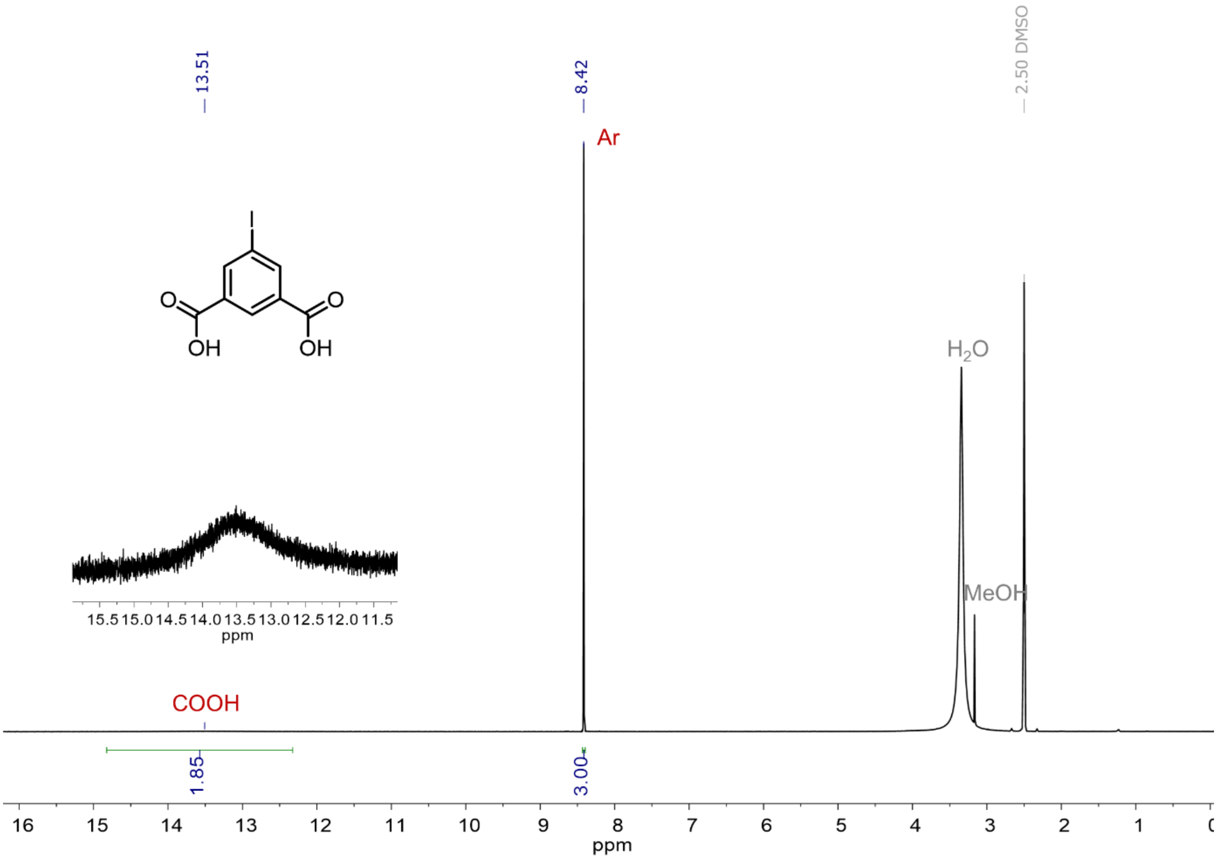

Figure S43: ^{1}H NMR (400 MHz, $CDCl_3$) of compound **9.**

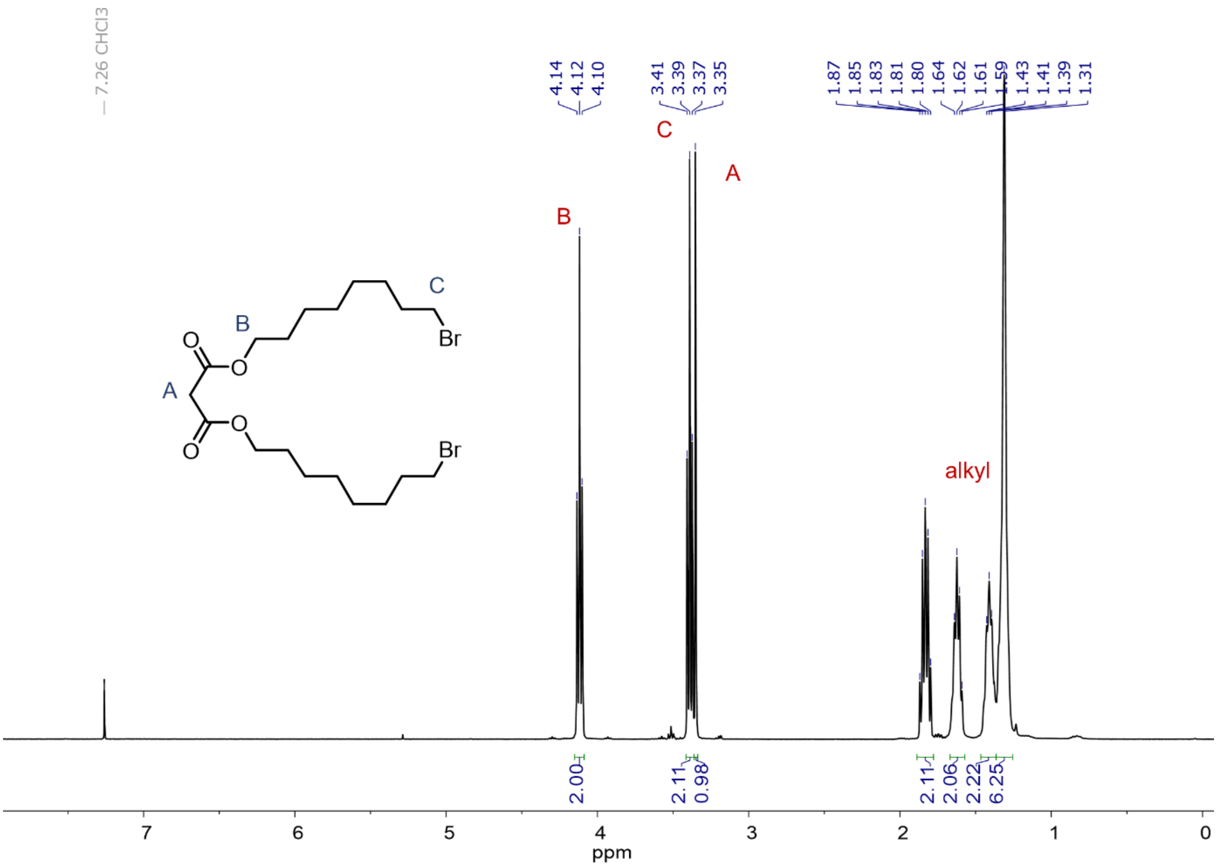

Figure S44: ^{1}H NMR (400 MHz, CDCl3) of compound **8.**

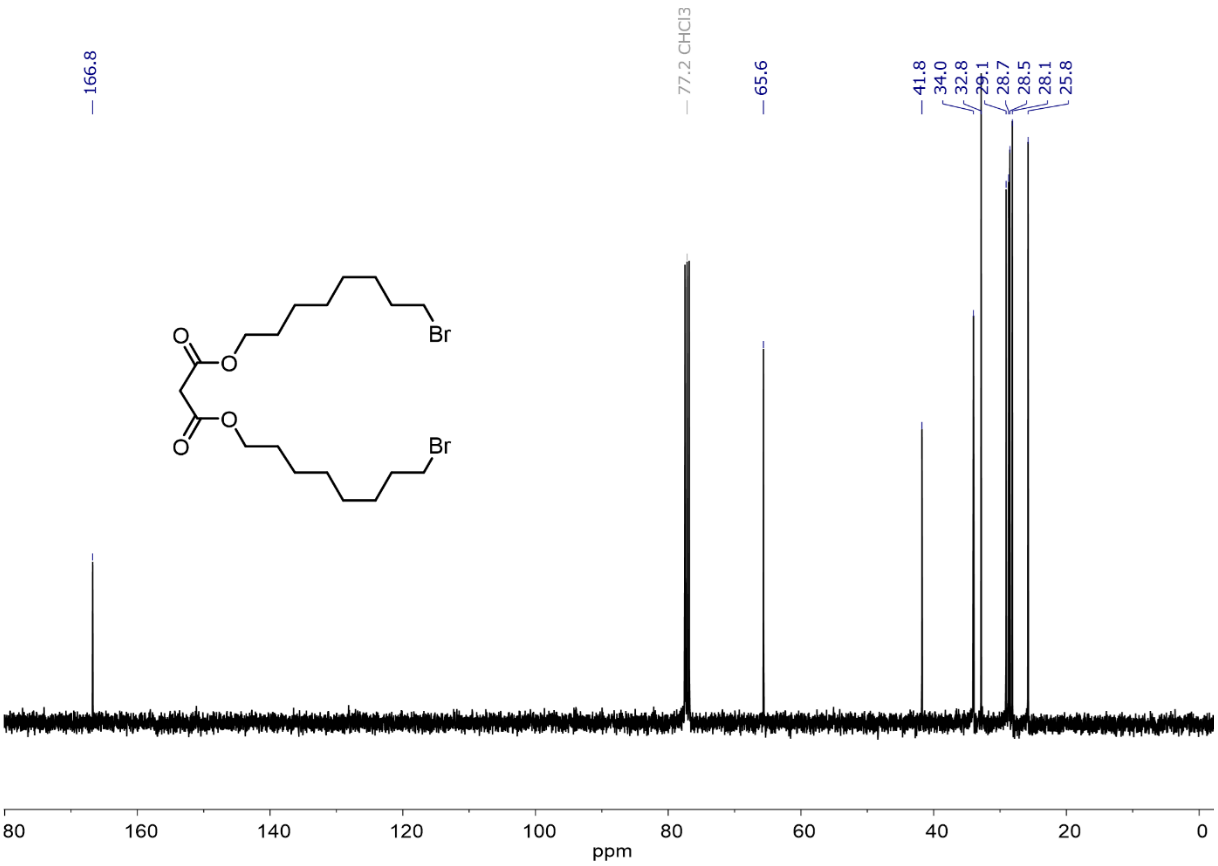

Figure S45: ^{13}C NMR (101 MHz, $CDCl_3$) of compound **8**.

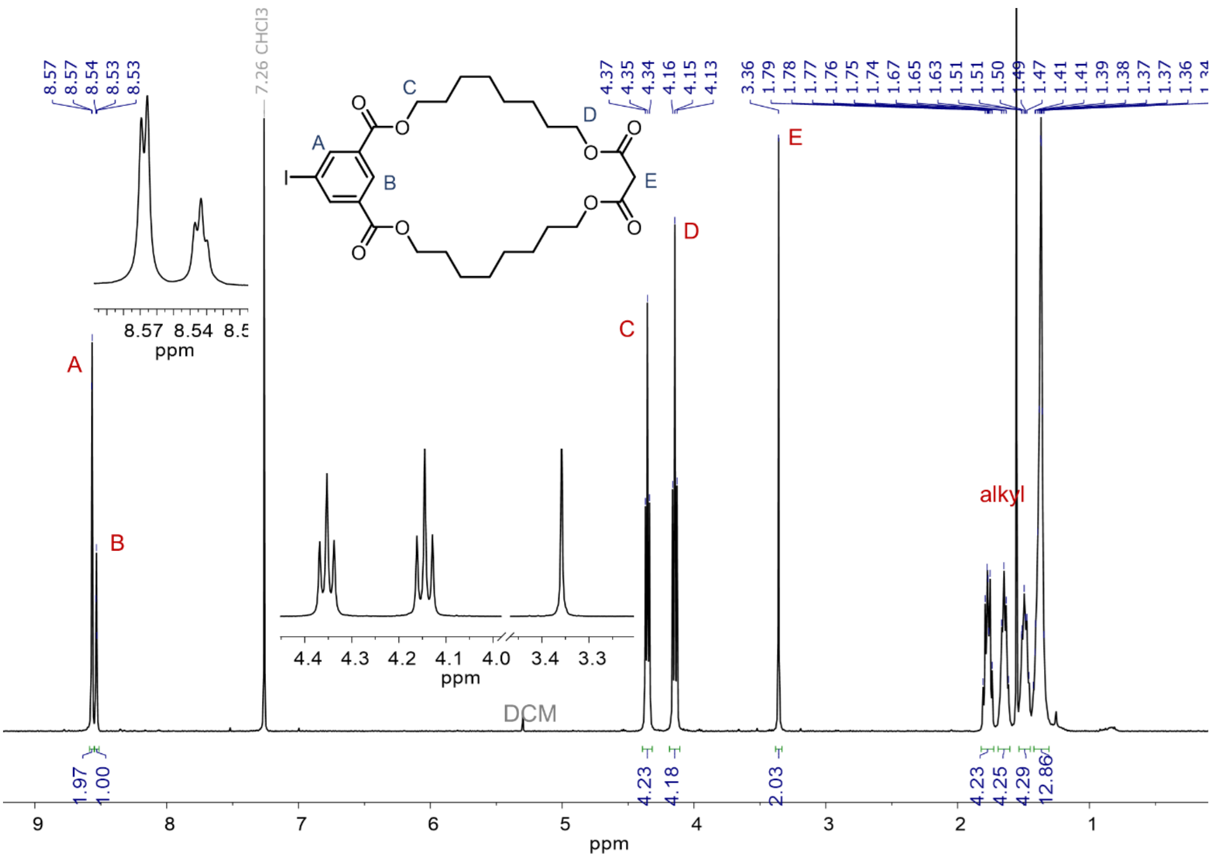

Figure S46: ^{1}H NMR (400 MHz, $CDCl_3$) of compound **10.**

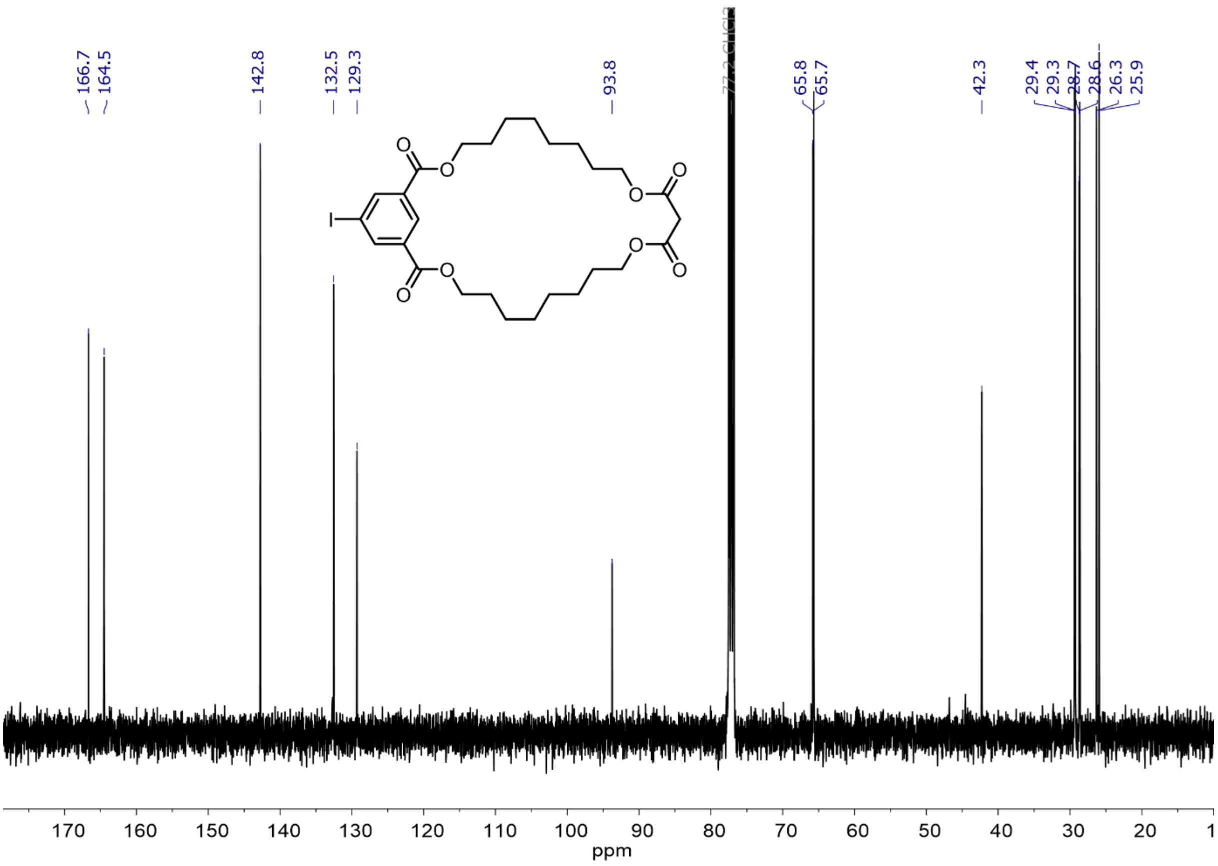

Figure S47: ^{13}C NMR (101 MHz, $CDCl_3$) of compound **10**.

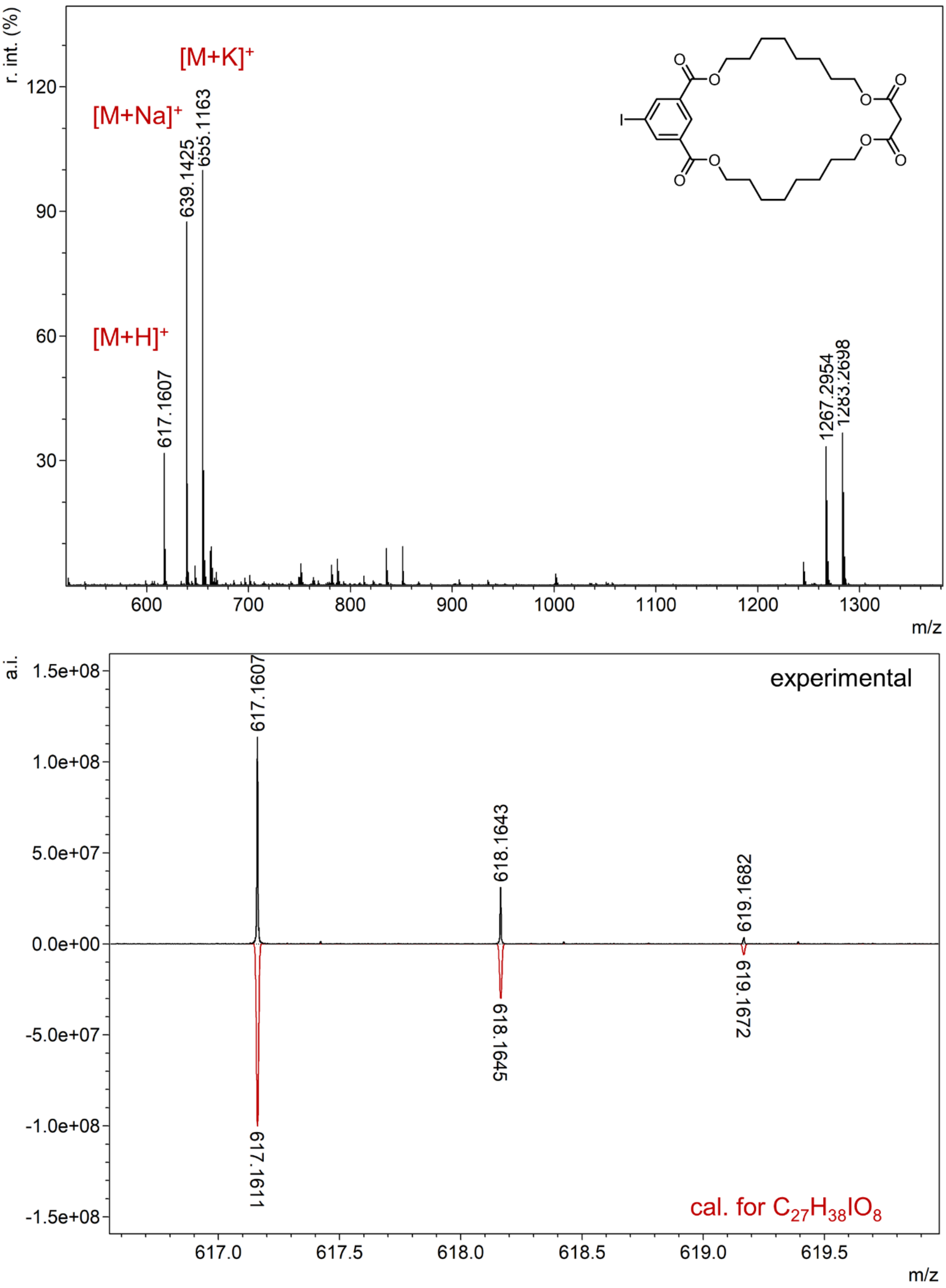

Figure S48: HRMS (MALDI, matrix: DCTB) of compound **10**.

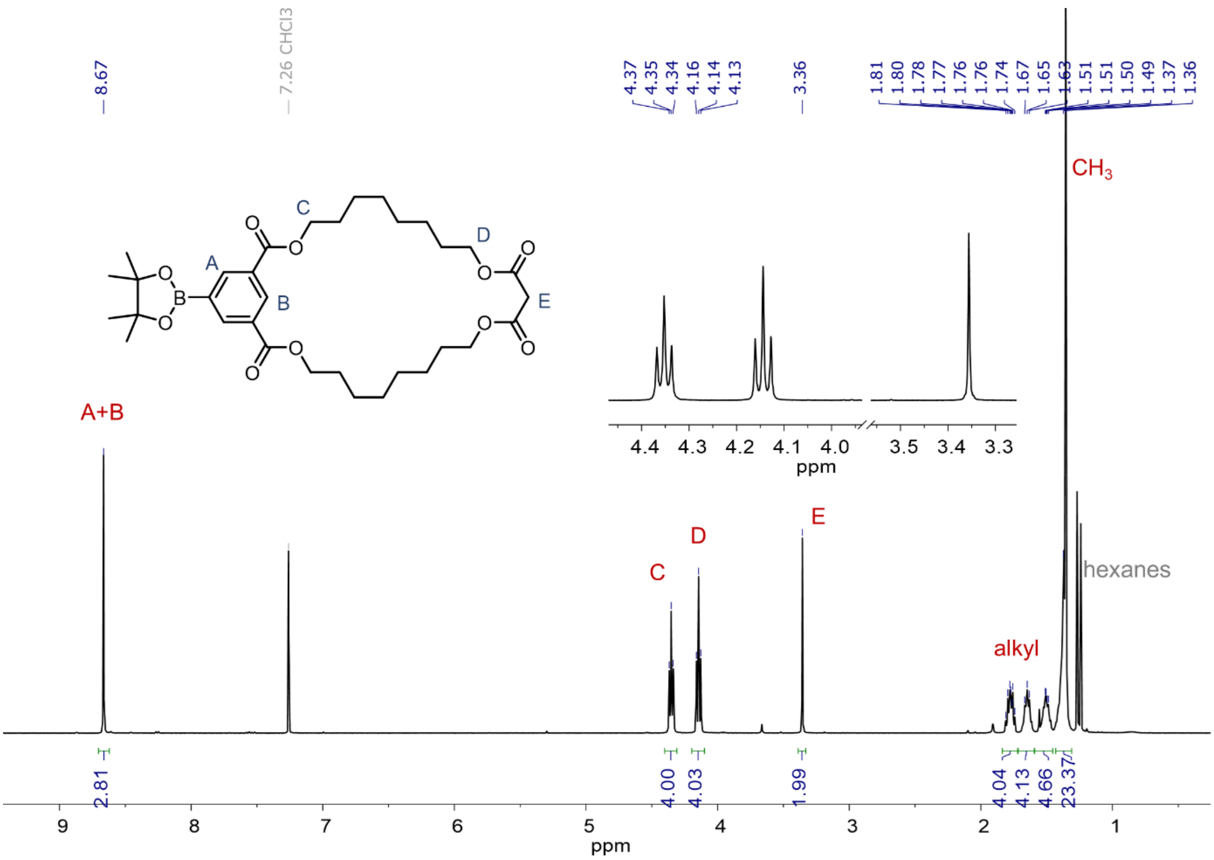

Figure S49: ^{1}H NMR (400 MHz, $CDCl_3$) of compound **11**

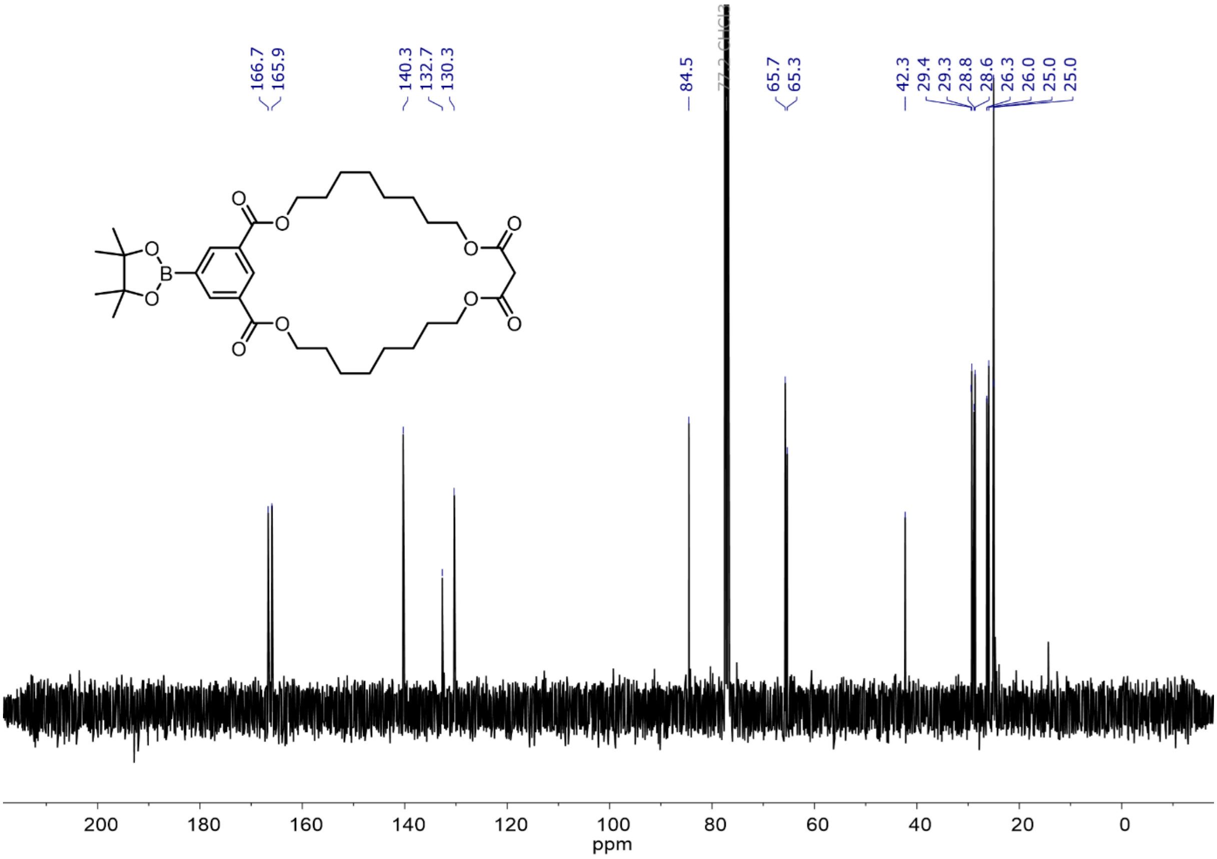

Figure S50: ^{13}C NMR (101 MHz, $CDCl_3$) of compound **11**.

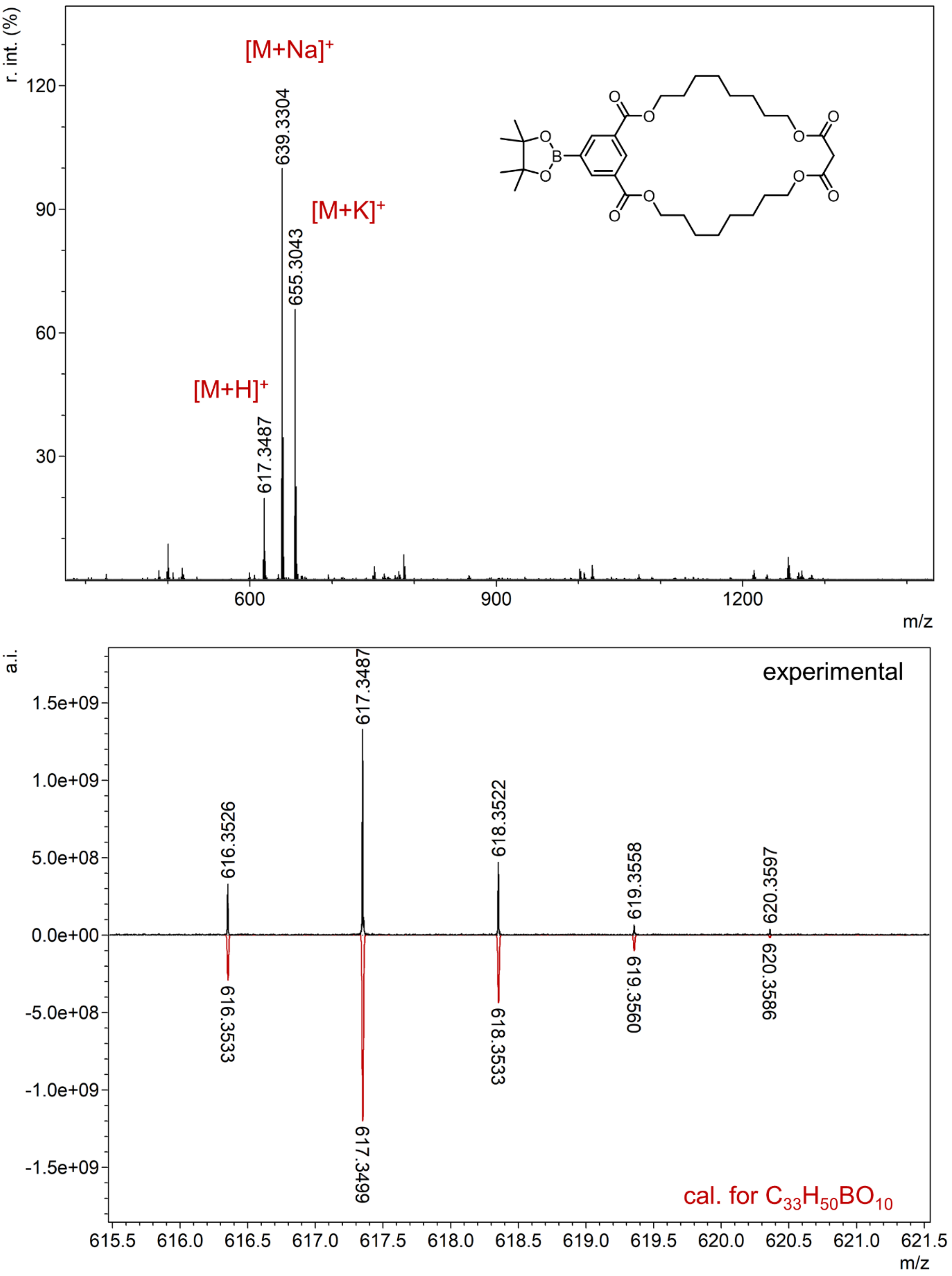

Figure S51: HRMS (MALDI, matrix: DCTB) of compound **11**.

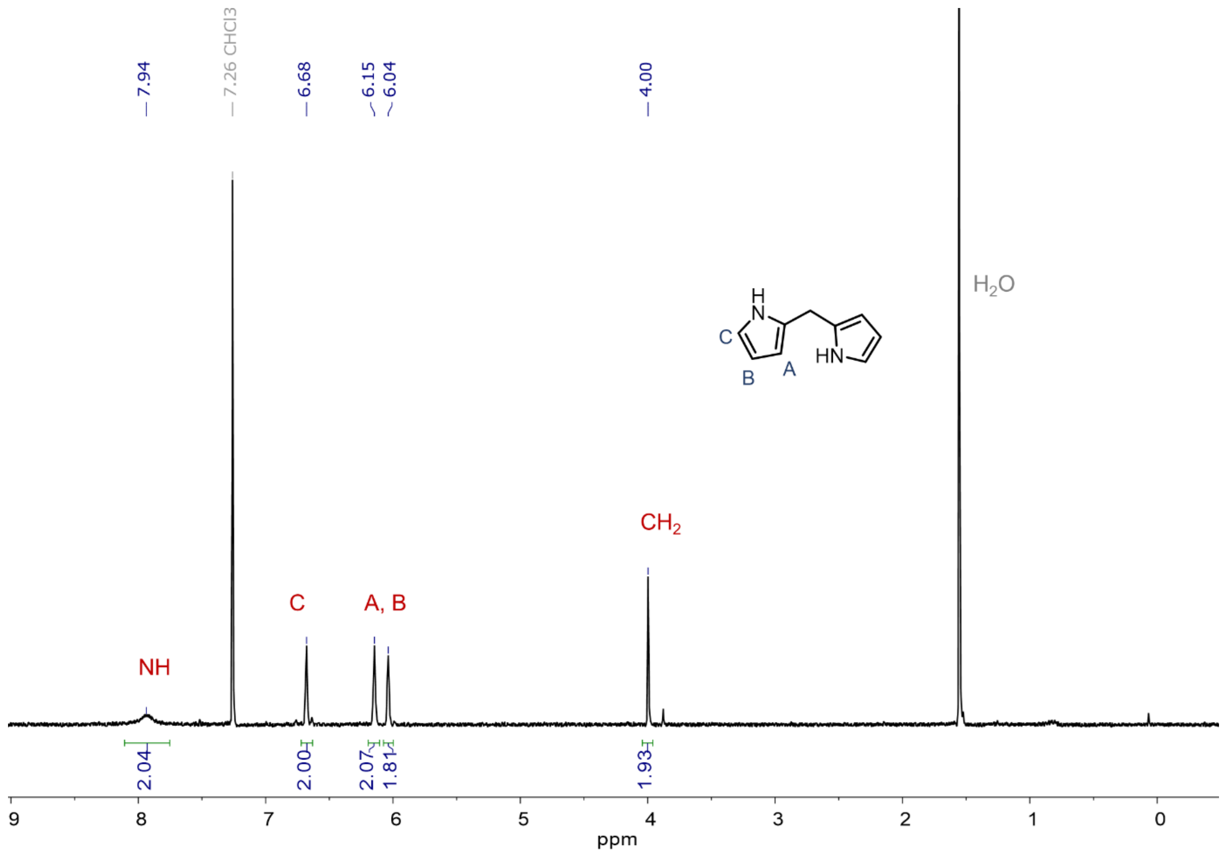

Figure S52: ^{1}H NMR (400 MHz, CDCl3) of di(1H-pyrrol-2-yl)methane.

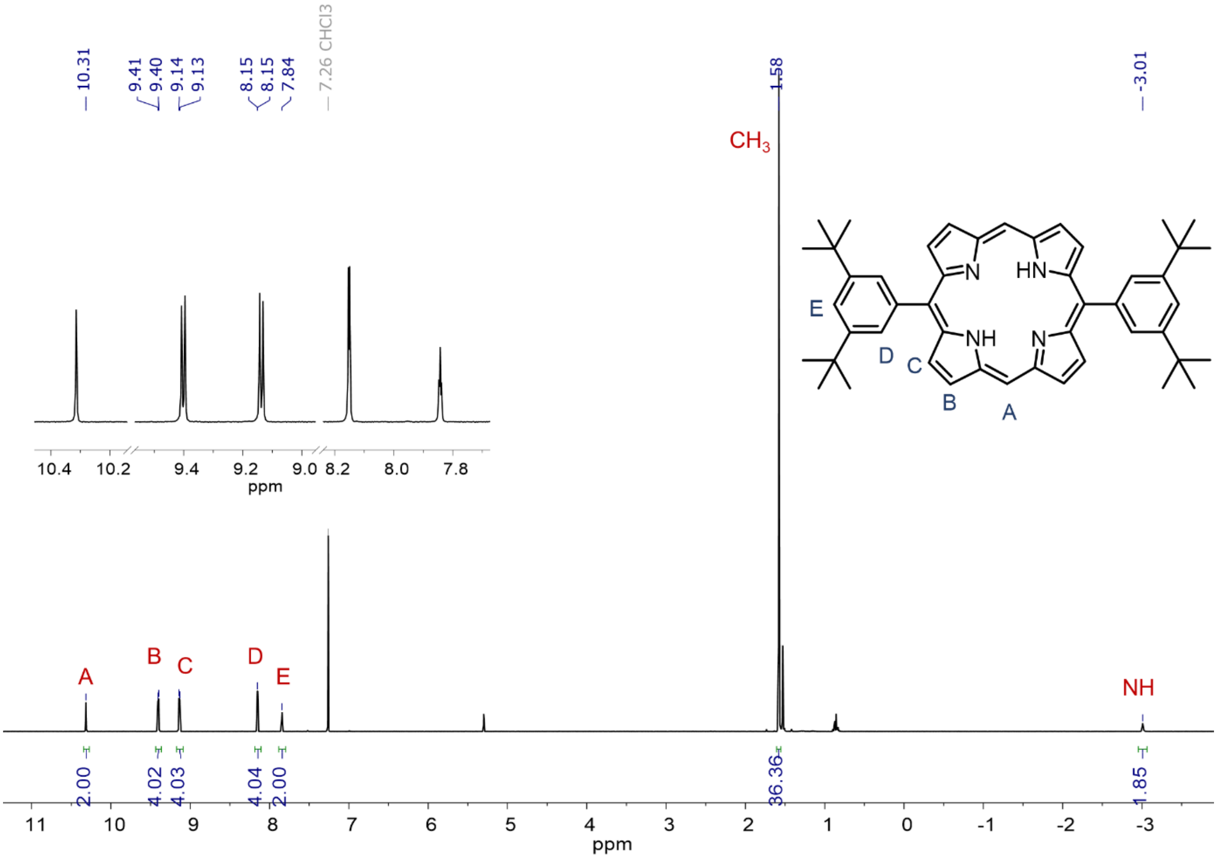

Figure S53: ^{1}H NMR (400 MHz, $CDCl_3$) of compound **4**.

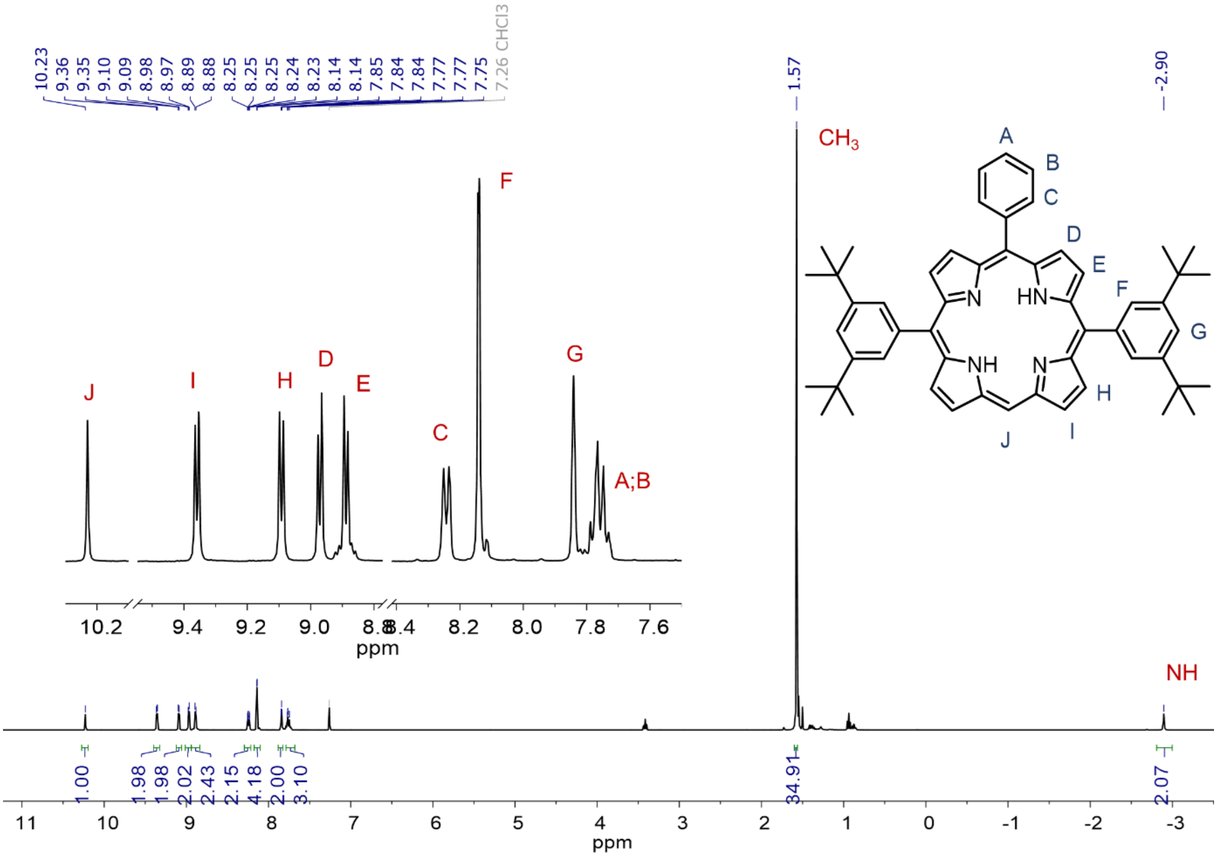

Figure S54: ^{1}H NMR (400 MHz, $CDCl_3$) of compound **5**.

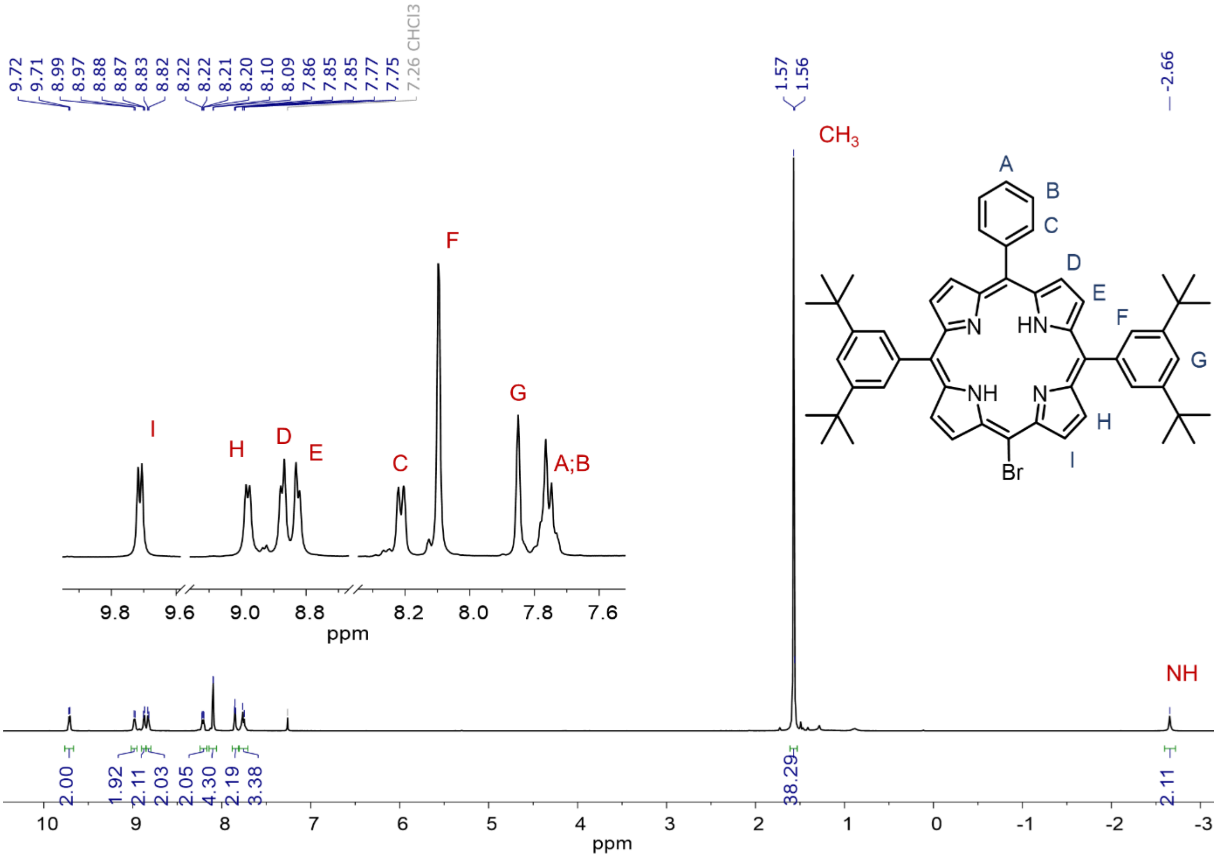

Figure S55: ^{1}H NMR (400 MHz, $CDCl_3$) of compound **6**.

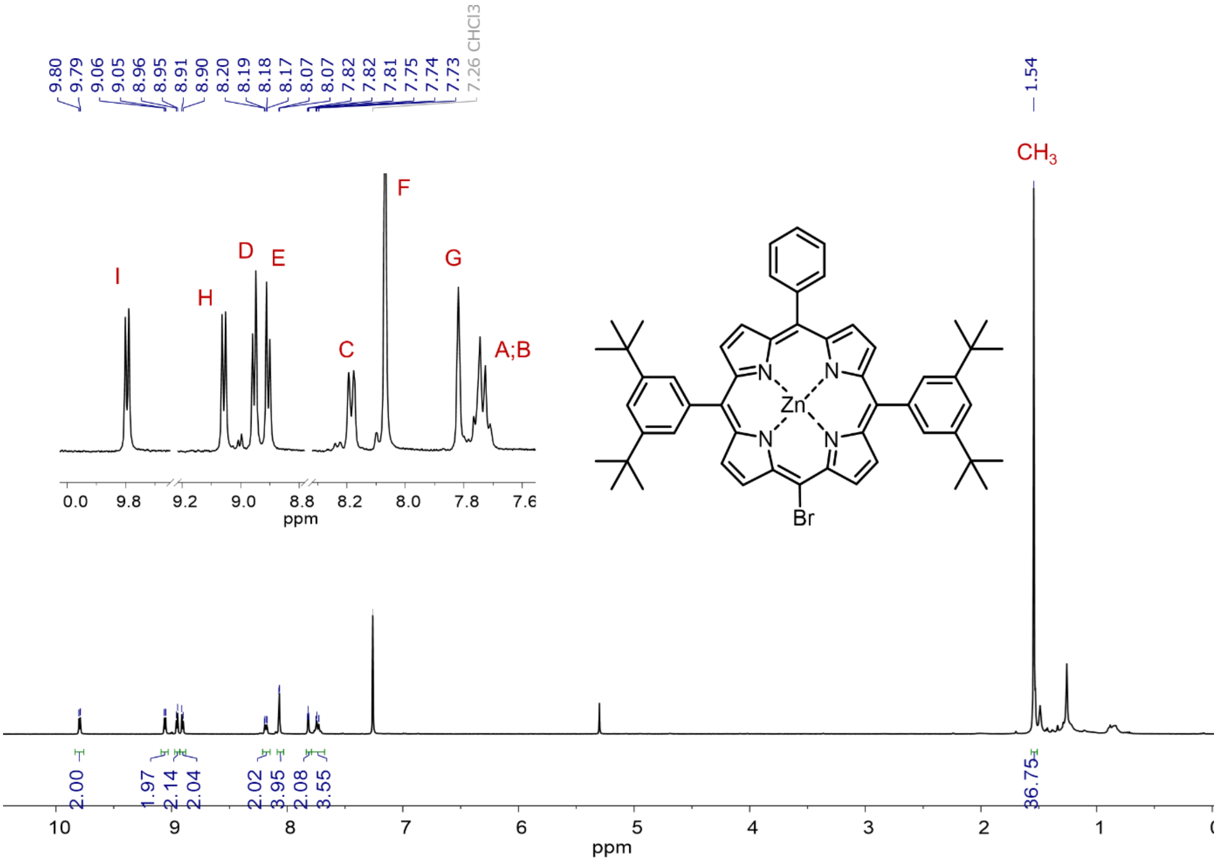

Figure S56: ^{1}H NMR (400 MHz, $CDCl_3$) of compound **7**.

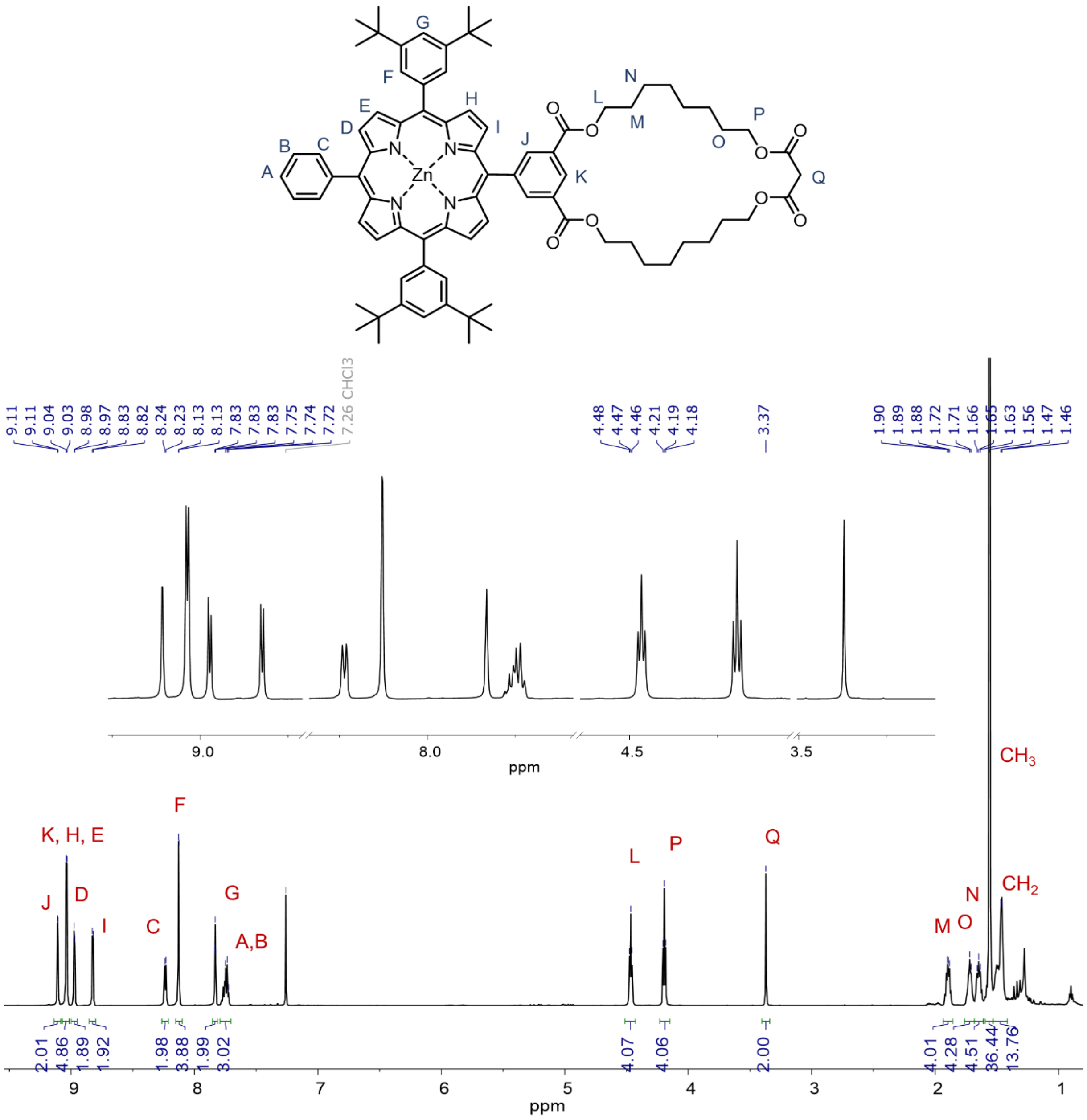

Figure S57: ^{1}H NMR (600 MHz, $CDCl_3$) of compound **12**.

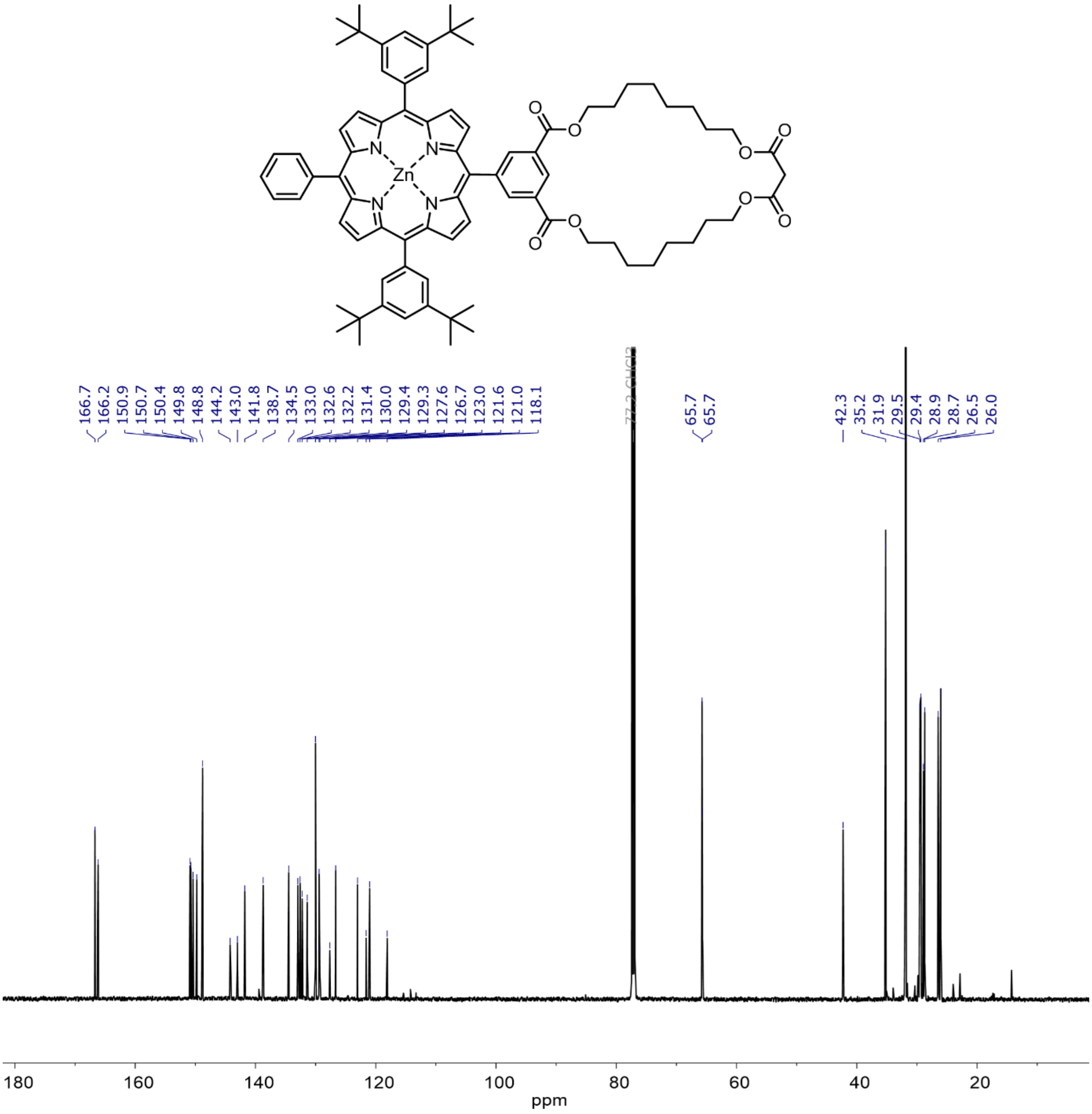

Figure S58: ^{13}C NMR (151 MHz, $CDCl_3$) of compound **12**.

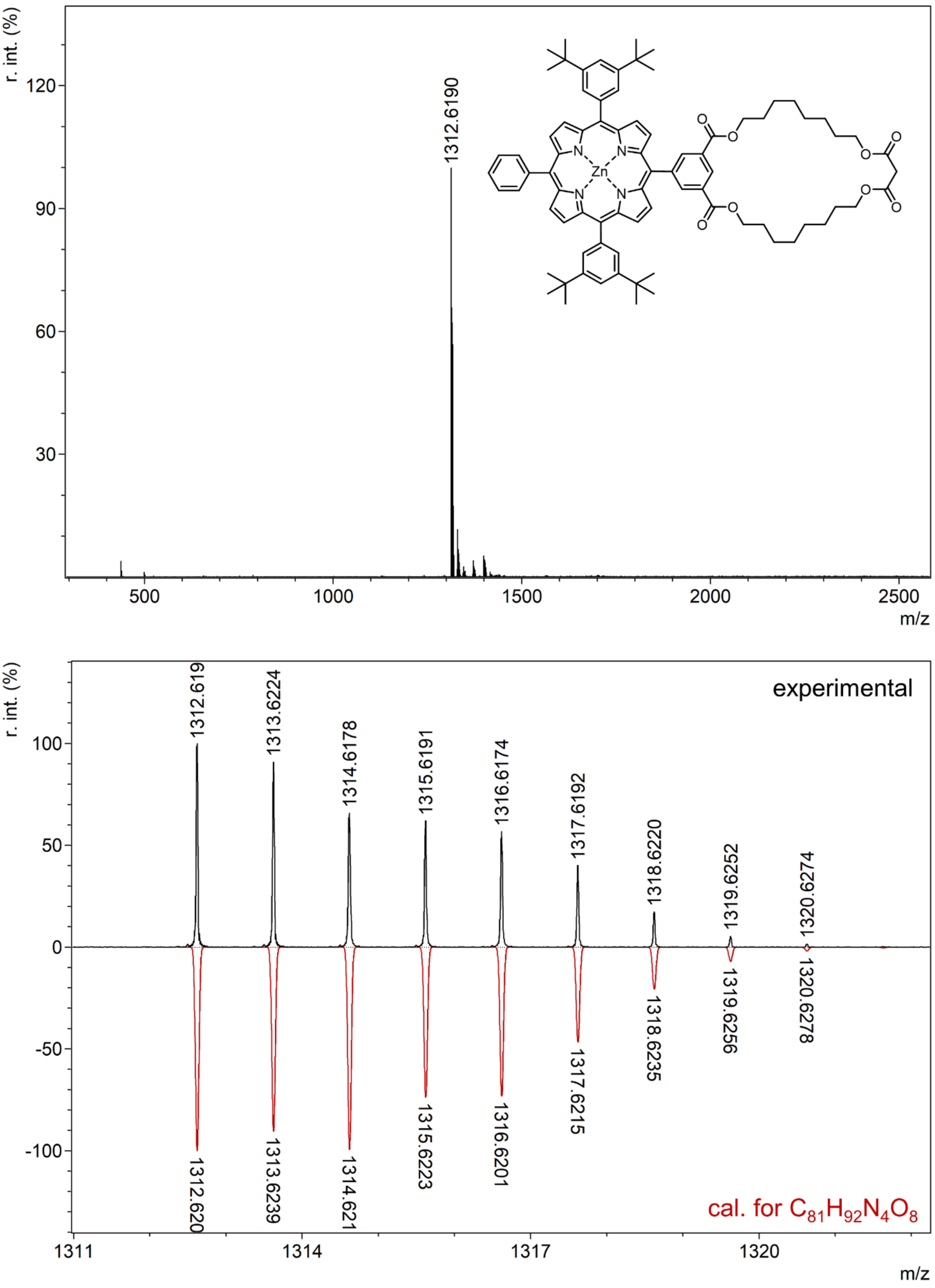

Figure S59: HRMS (MALDI, matrix: DCTB) of compound **12**.

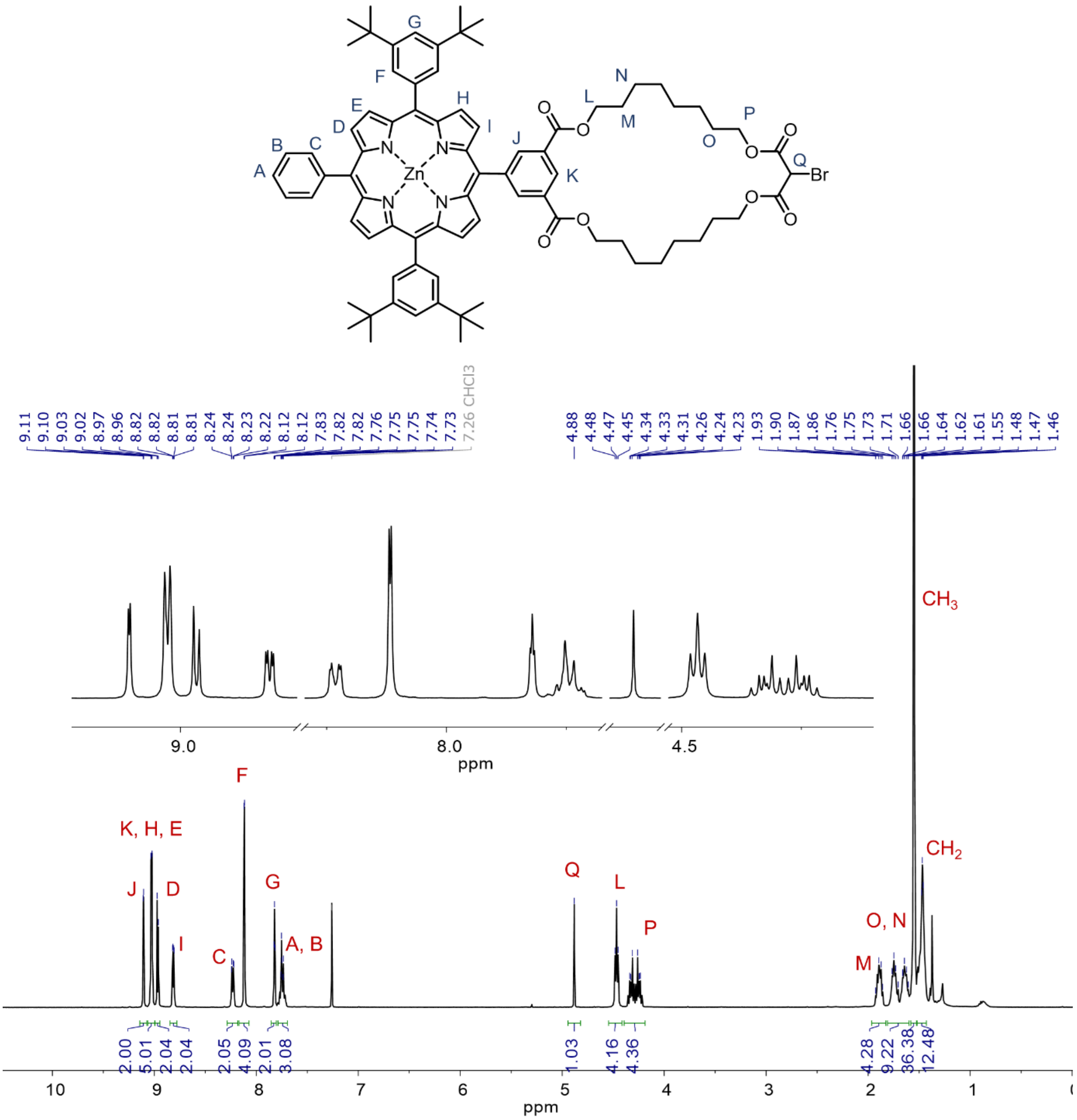

Figure S60: ^1H NMR (400 MHz, $CDCl_3$) of compound **13**.

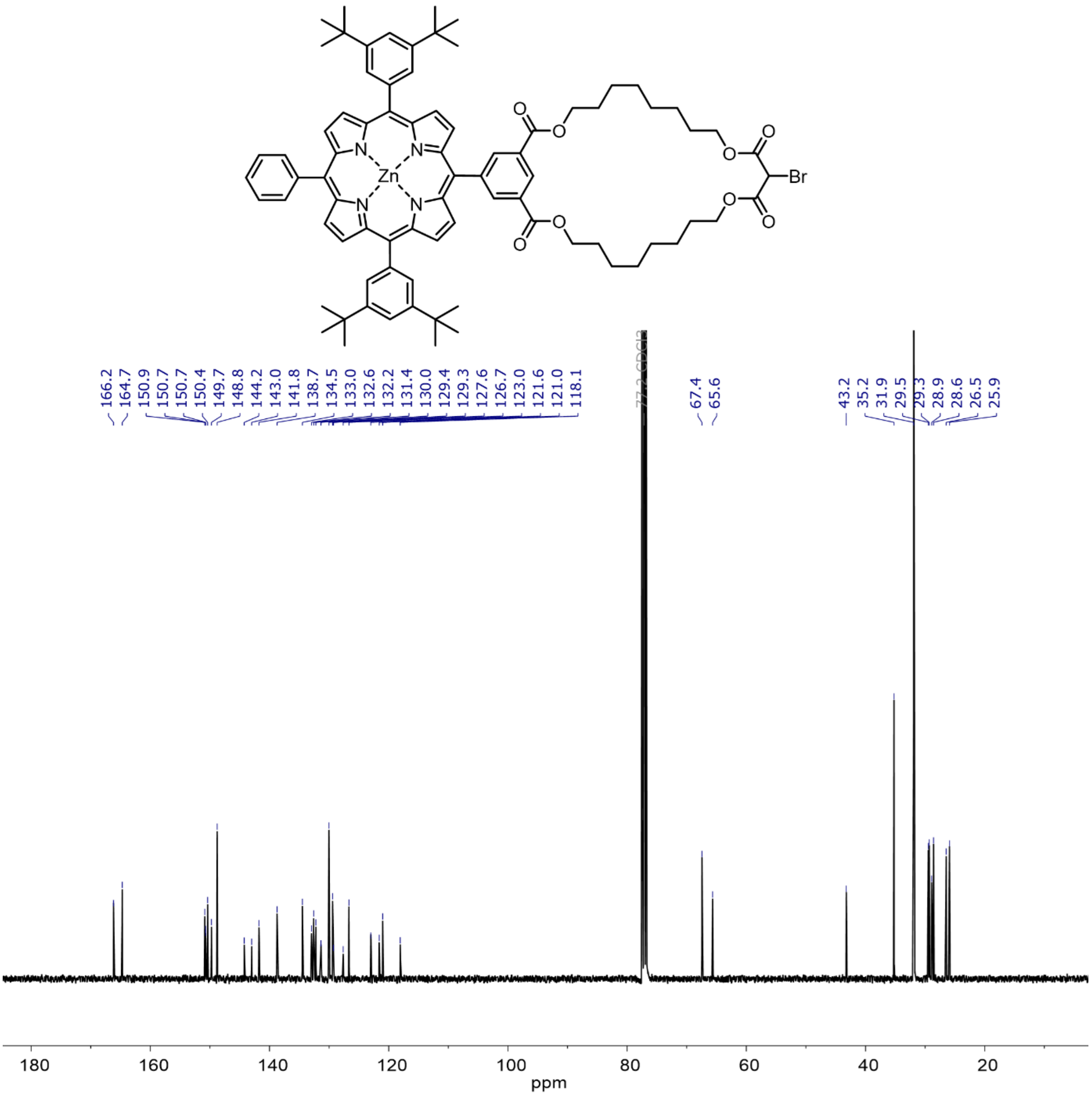

Figure S61: ^{13}C NMR (151 MHz, $CDCl_3$) of compound **13**.

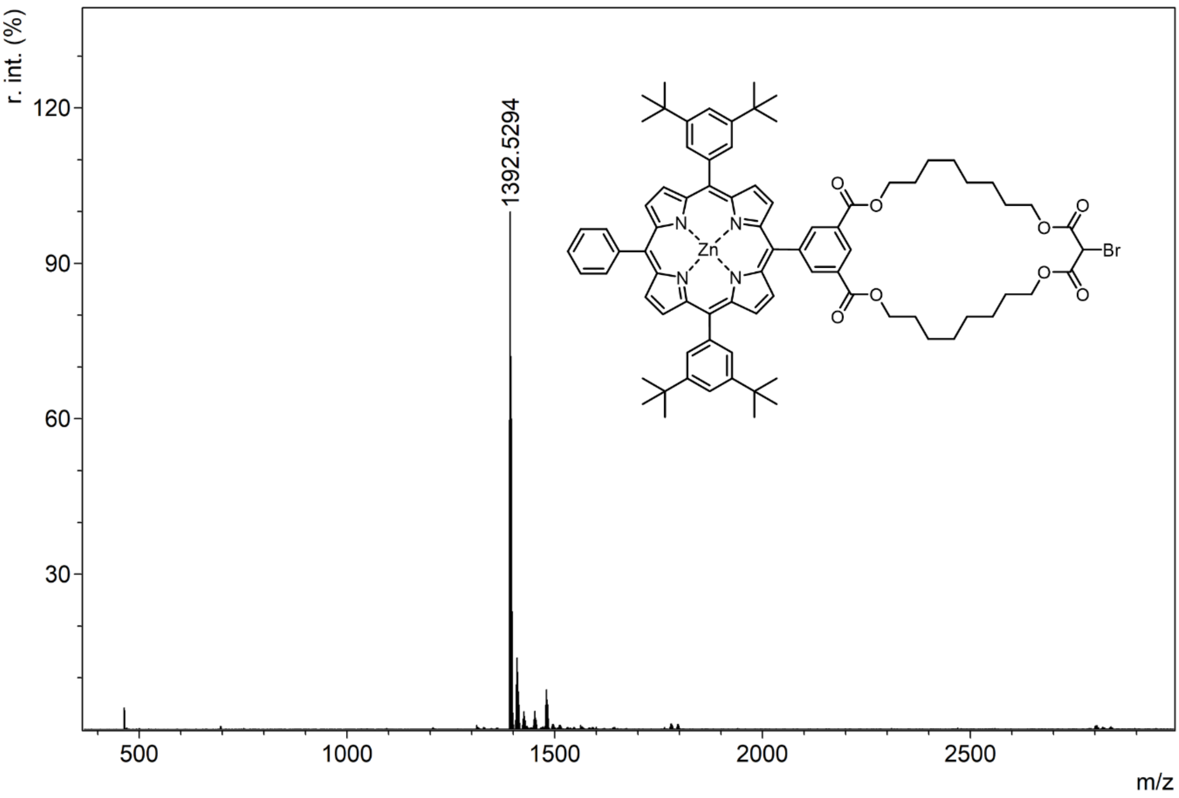

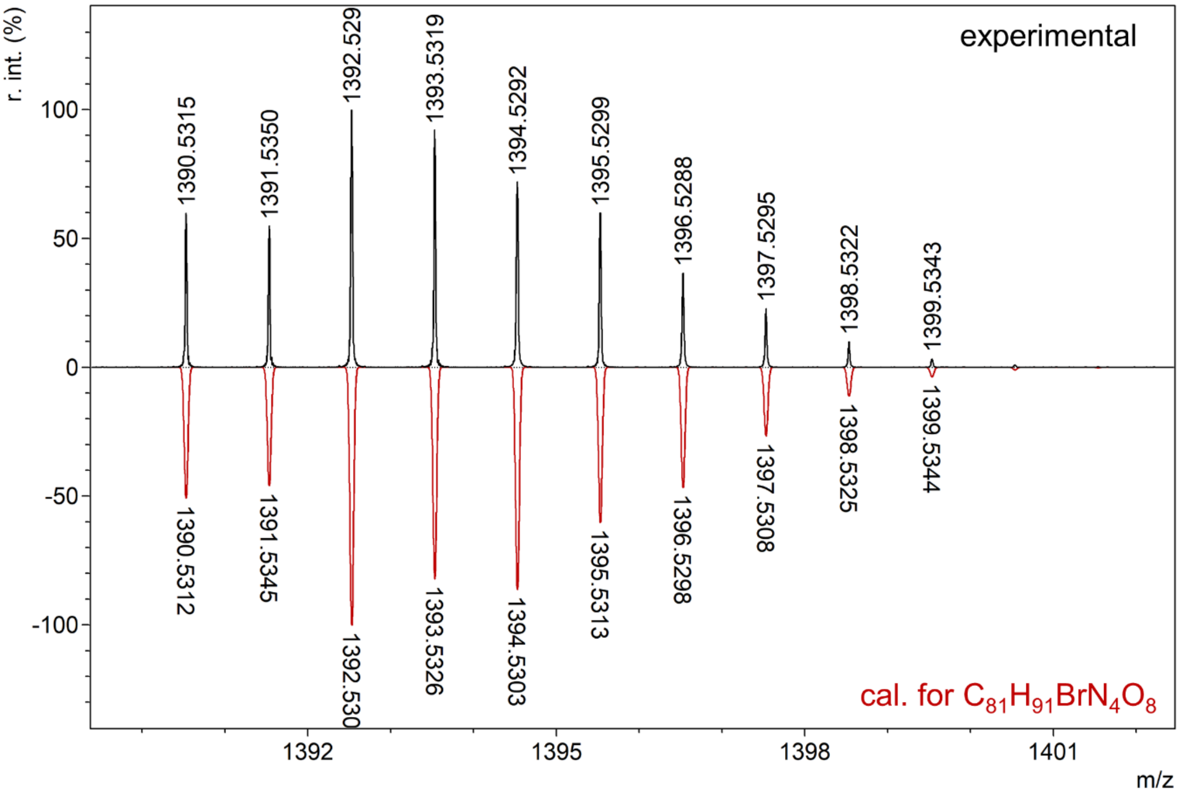

Figure S62: HRMS (MALDI, matrix: DCTB) of compound **13**.

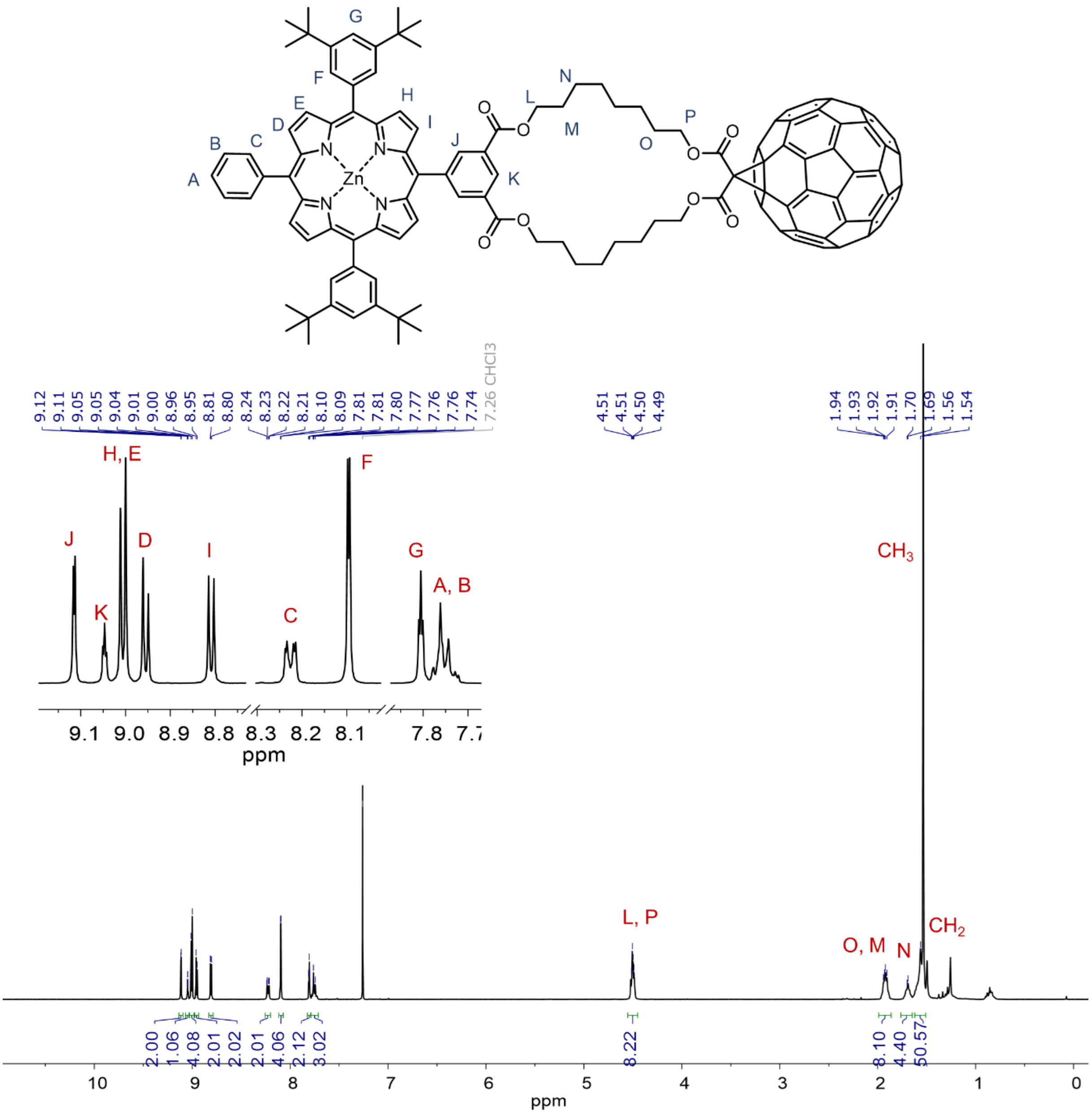

Figure S63: ^{1}H NMR (400 MHz, $CDCl_3$) of compound **14**.

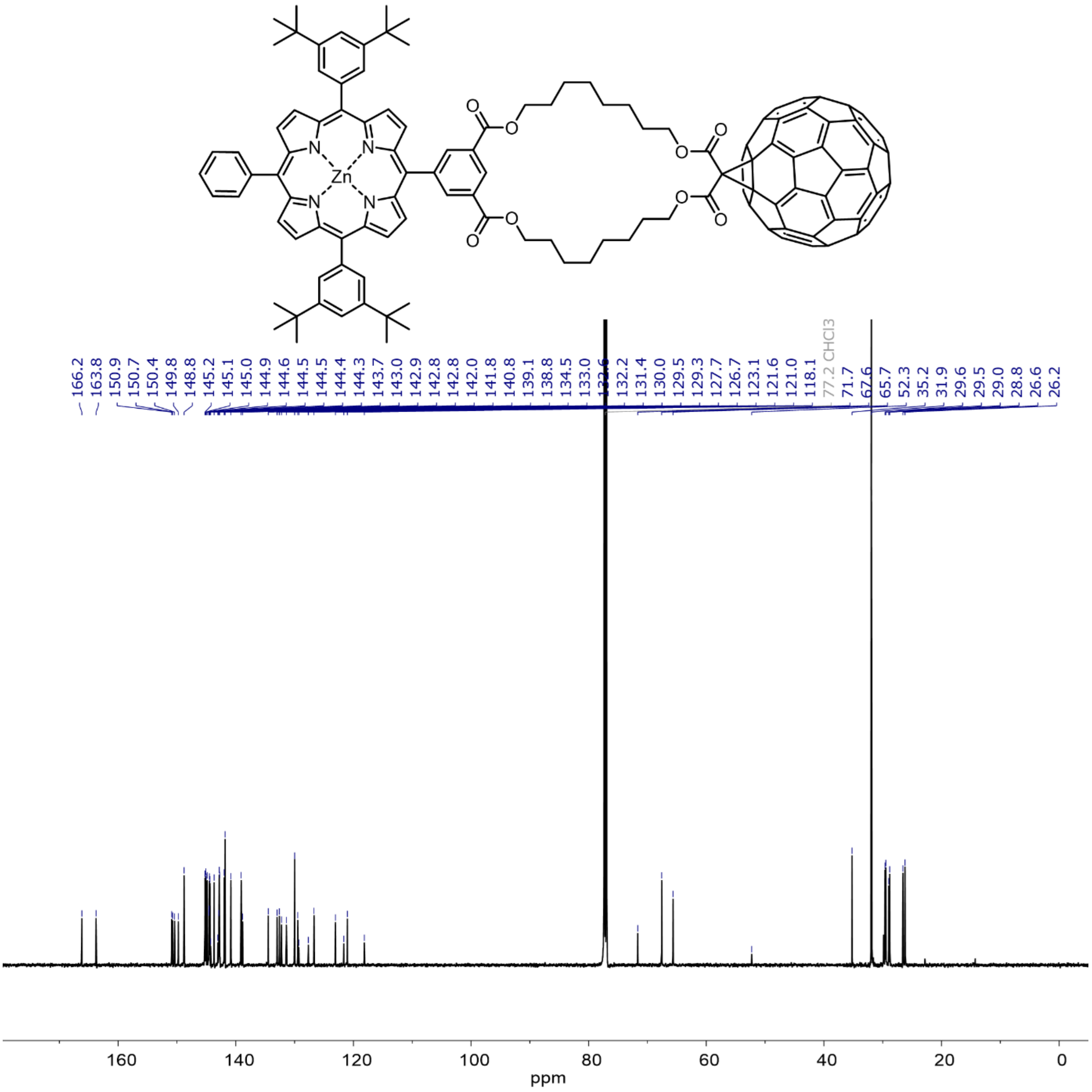

Figure S64: ^{13}C NMR (151 MHz, $CDCl_3$) of compound **14**.

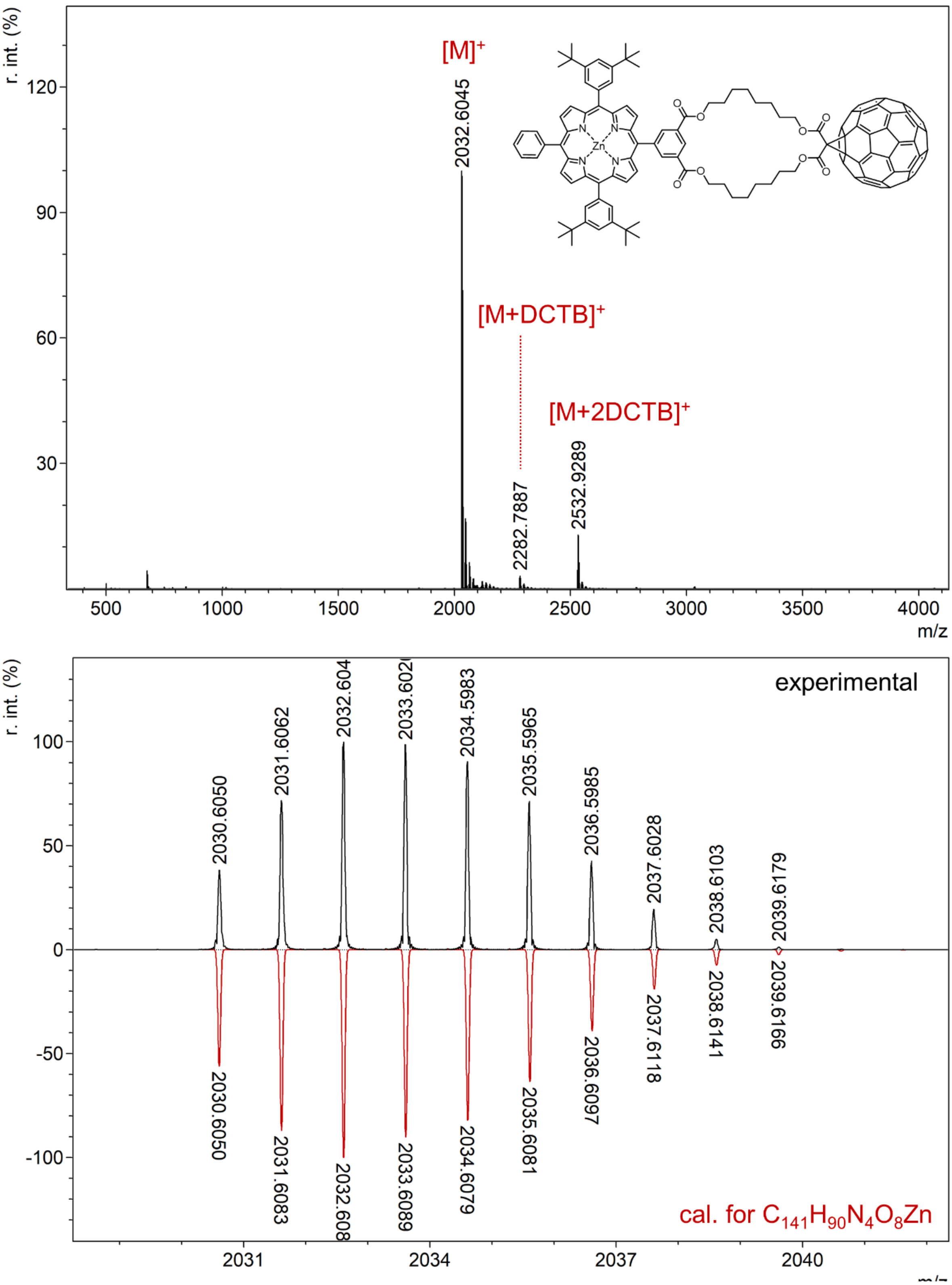

Figure S65: HRMS (MALDI, matrix: DCTB) of compound **14**.

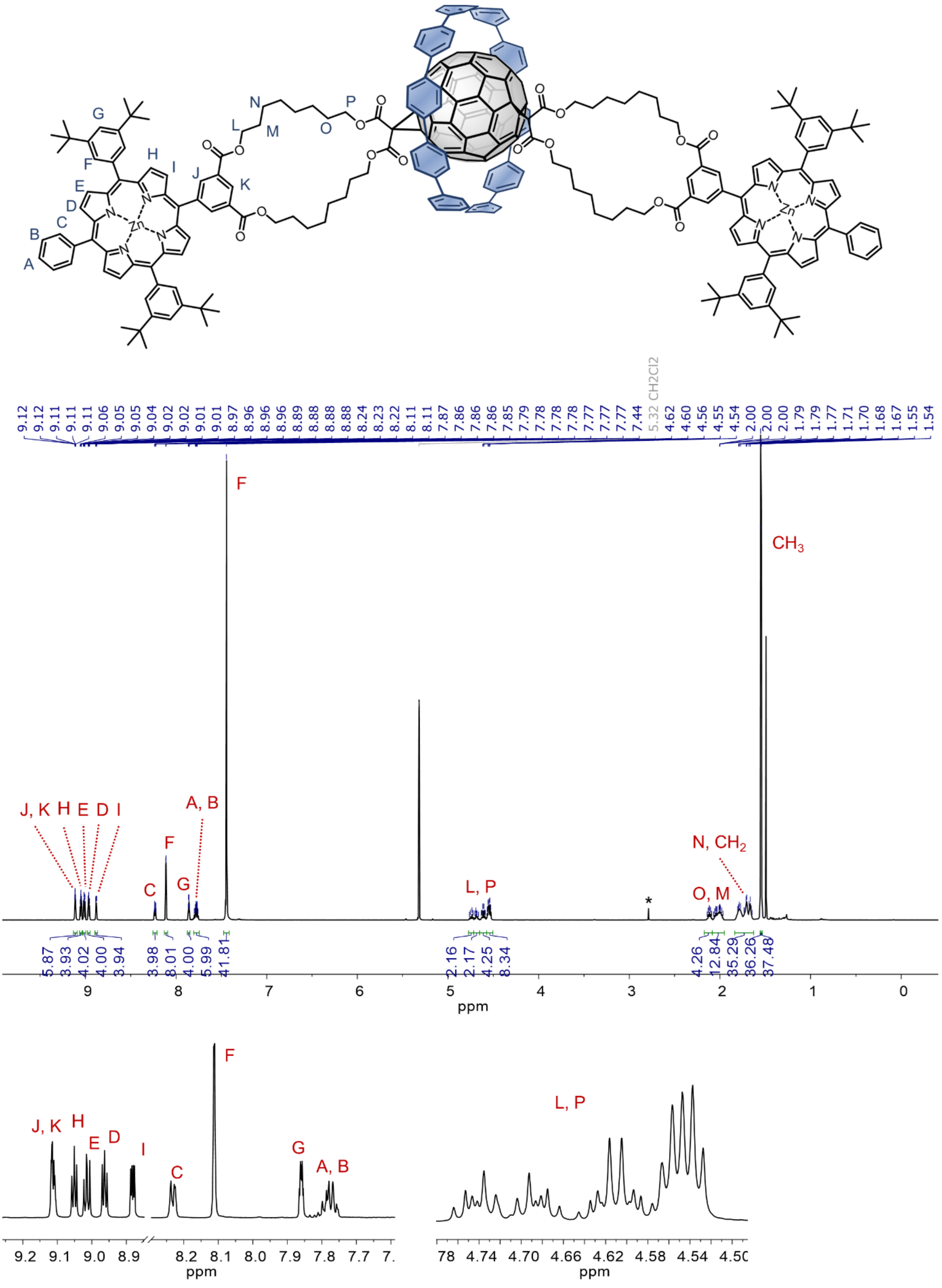

Figure S66: ^{1}H NMR (600 MHz, CD_2Cl_2) of compound ***trans*-2-1**.

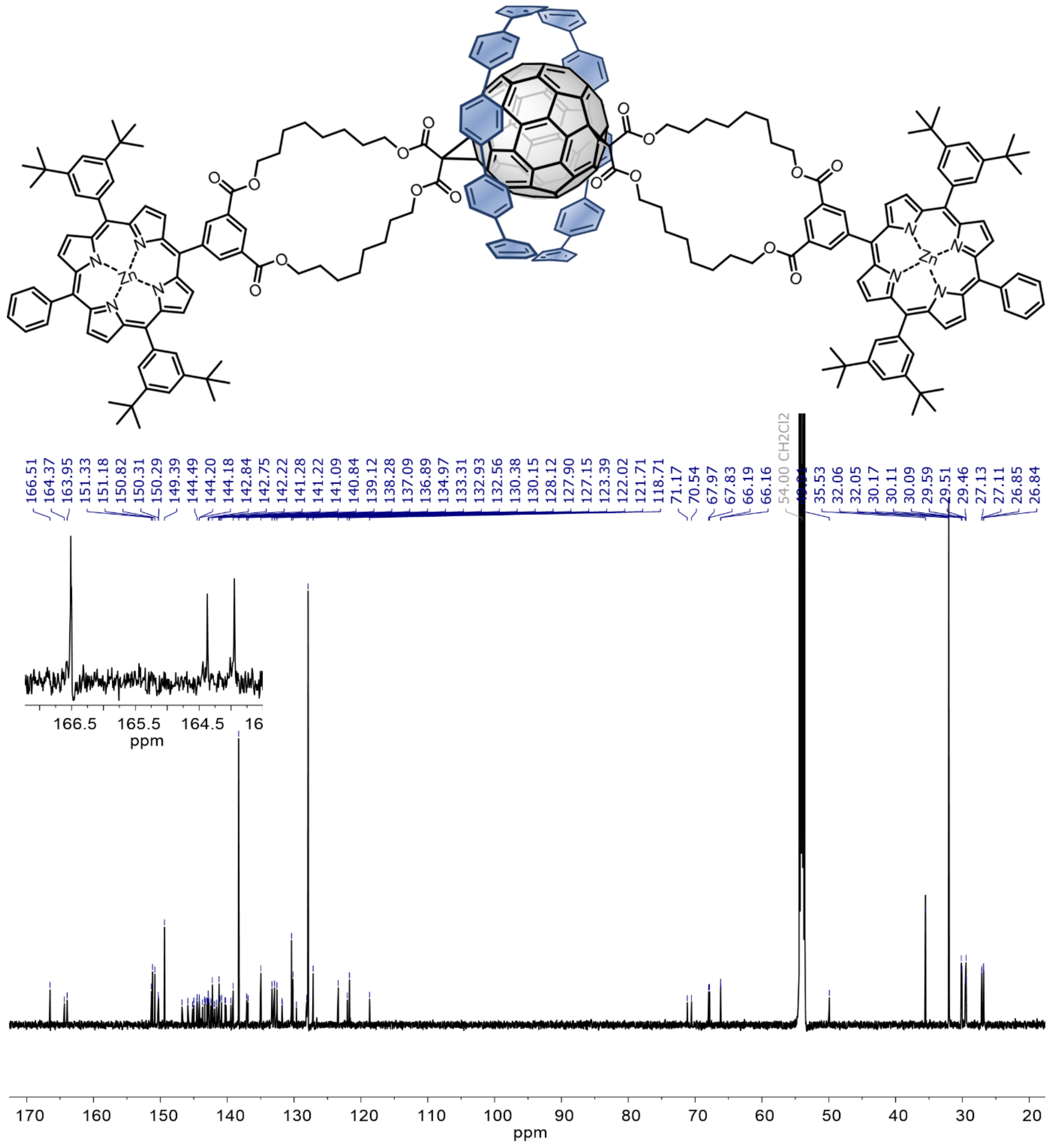

Figure S67: ^{13}C NMR (151 MHz, CD_2Cl_2) of compound ***trans*-2-1**.

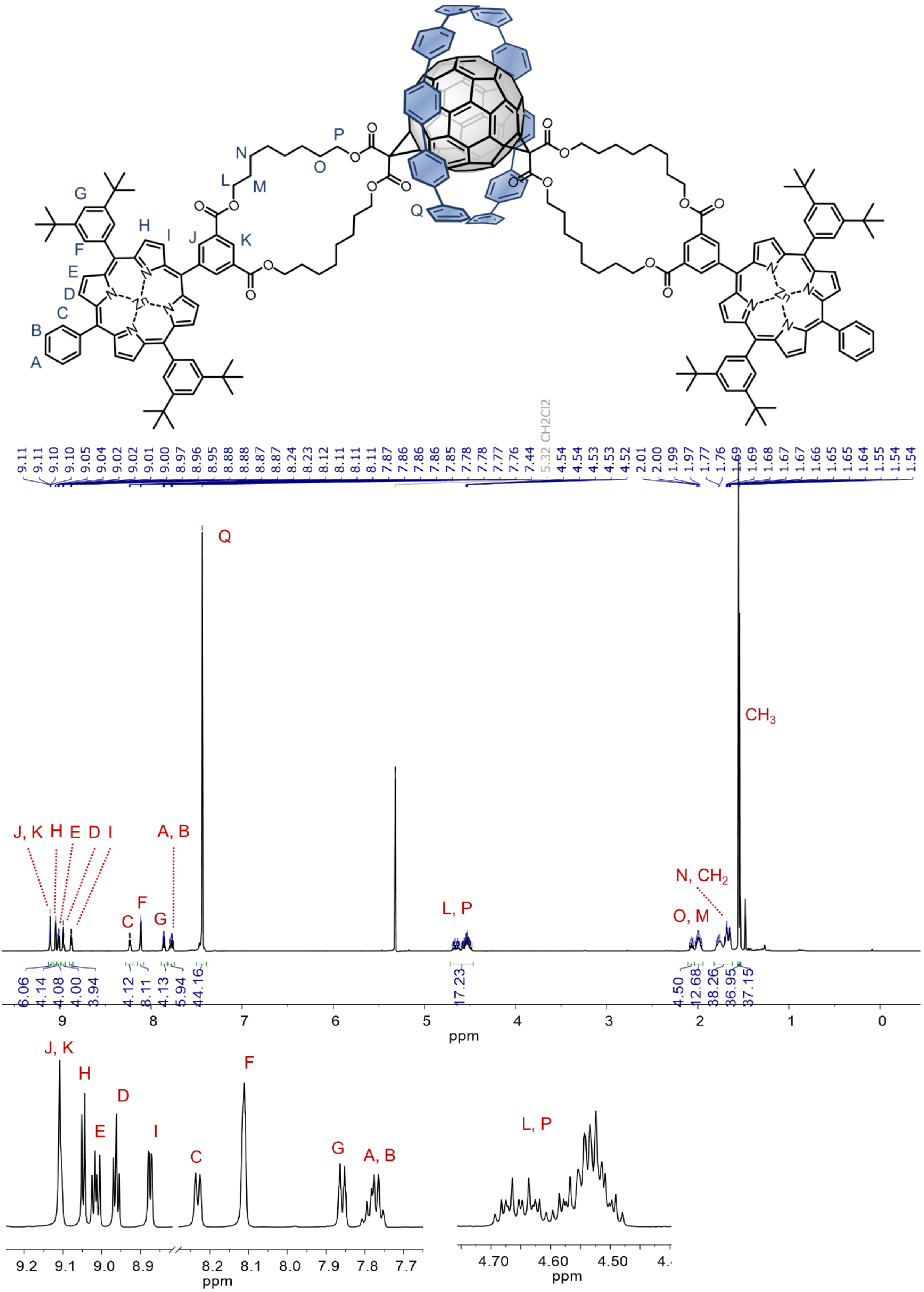

Figure S68: ^{1}H NMR (600 MHz, CD_2Cl_2) of compound ***trans*-3-1**.

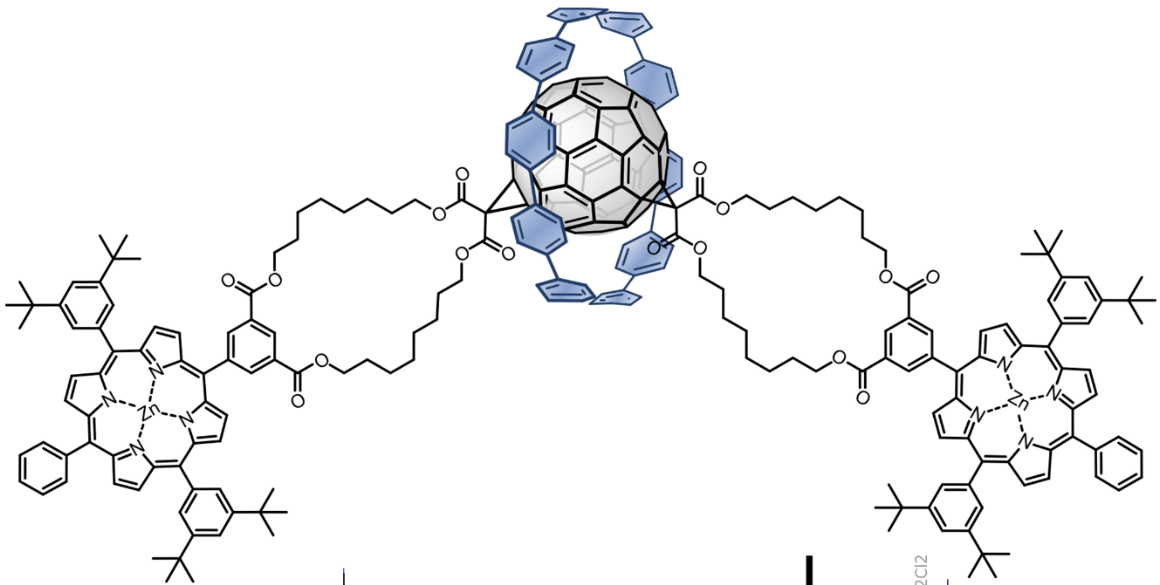

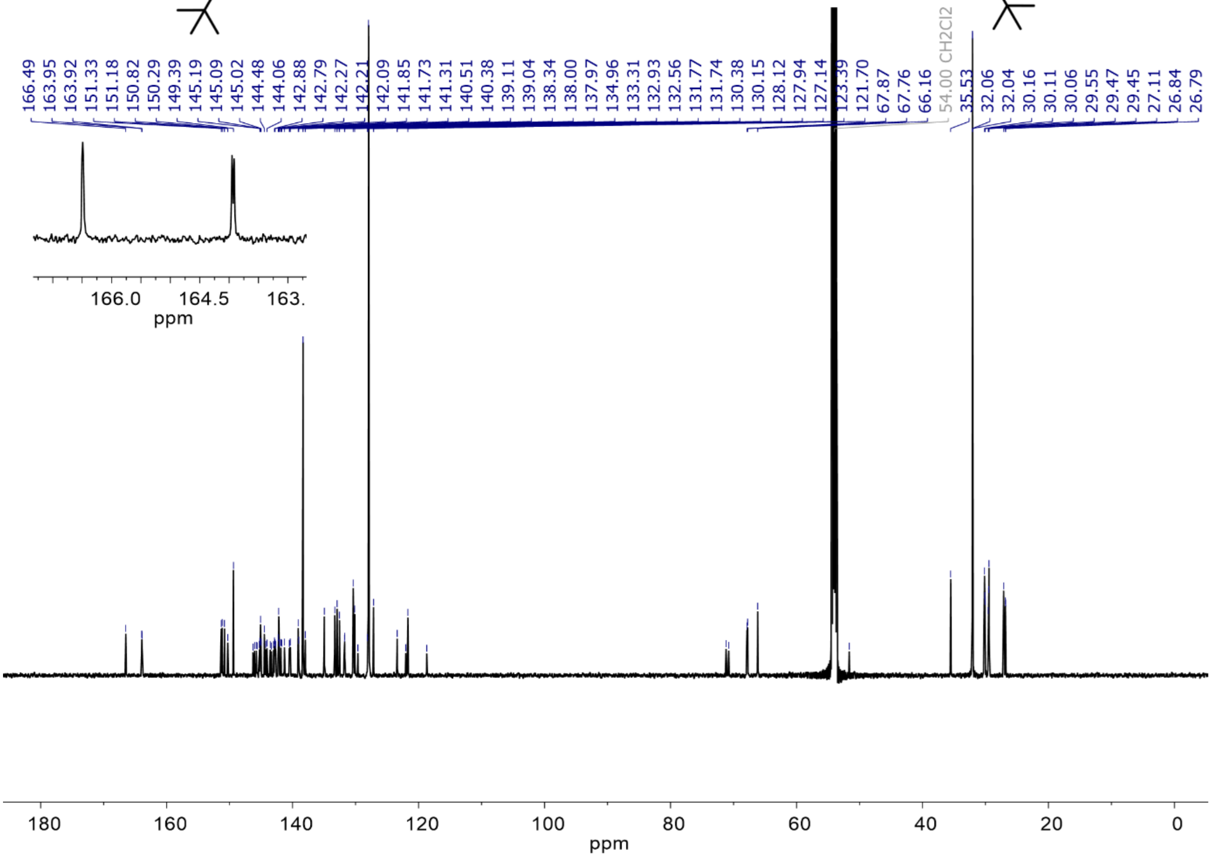

Figure S69: ^{13}C NMR (151 MHz, CD_2Cl_2) of compound ***trans*-3-1**.

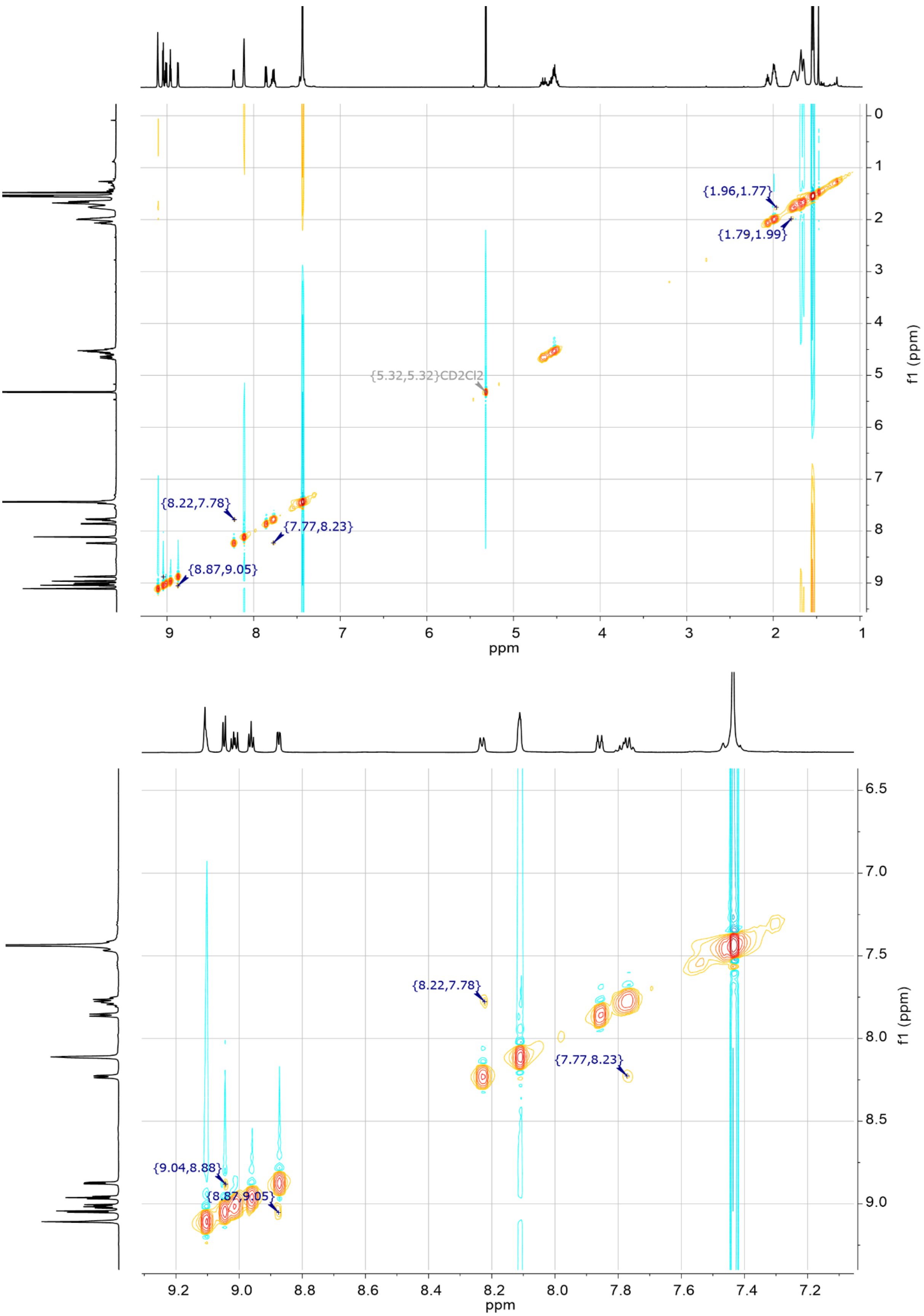

Figure S70: 2D-^{1}H,^{1}H-NOESY NMR (600 MHz, CD_2Cl_2) of compound ***trans*-3-1**.

The only clear cross-peaks observed are between the neighbouring protons H (9.04 ppm) and I (8.88 ppm), as well as C (8.22) and B (7.78) and between protons in the alkyl chains.

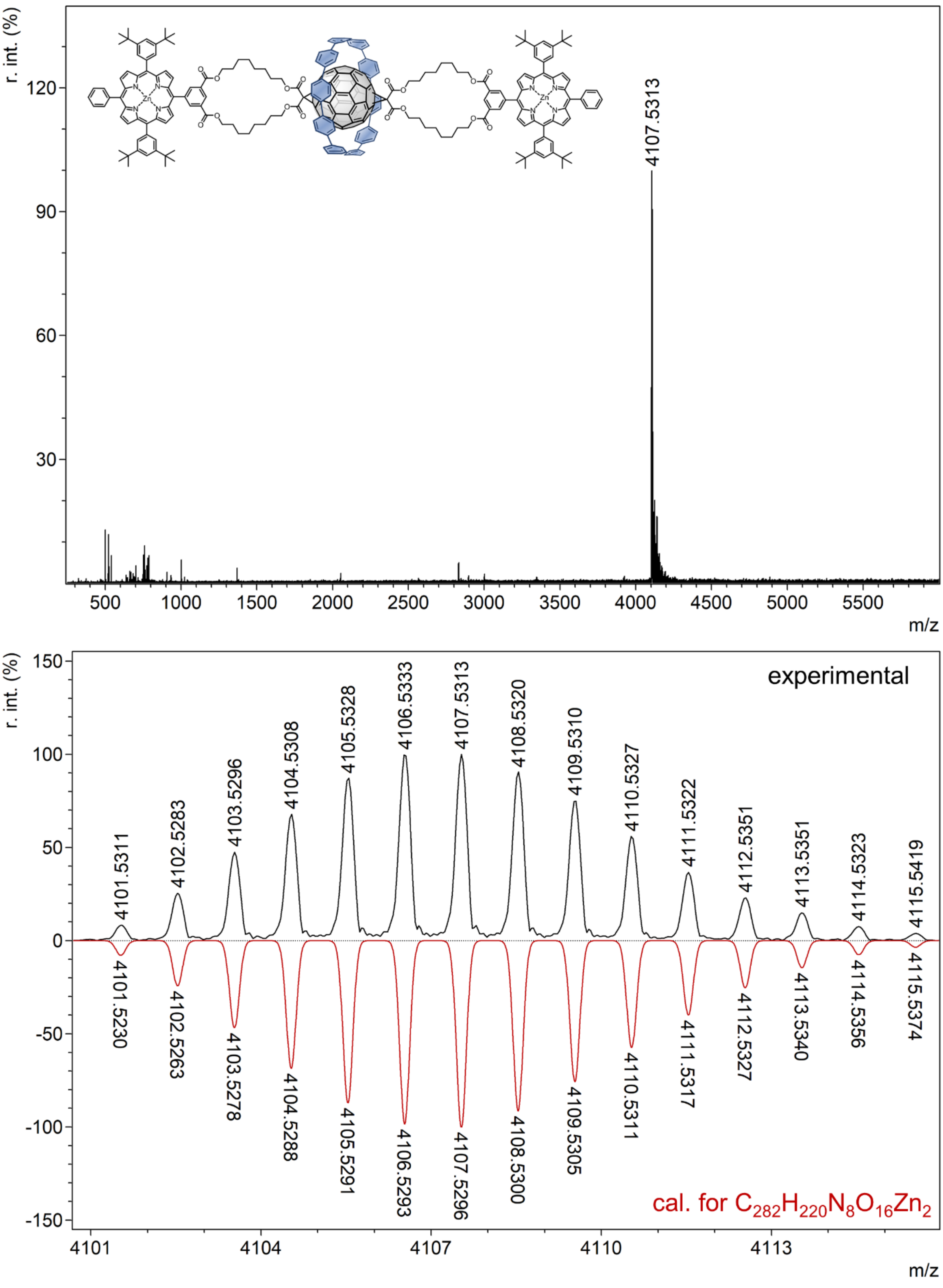

Figure S71: HRMS (MALDI, matrix: DCTB) of compound **1**.

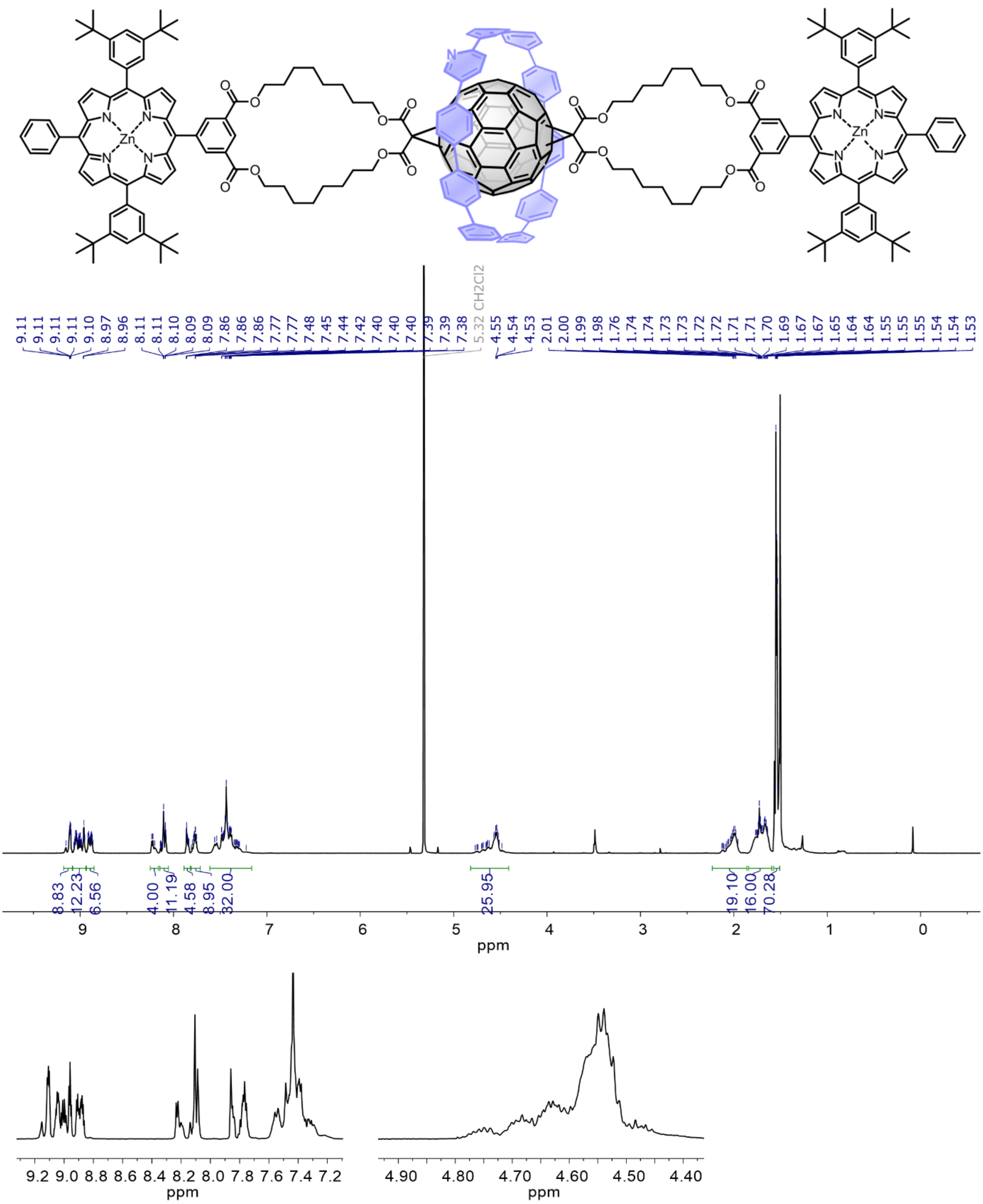

Figure S72: ^{1}H NMR (600 MHz, CD_2Cl_2) of compound **2**

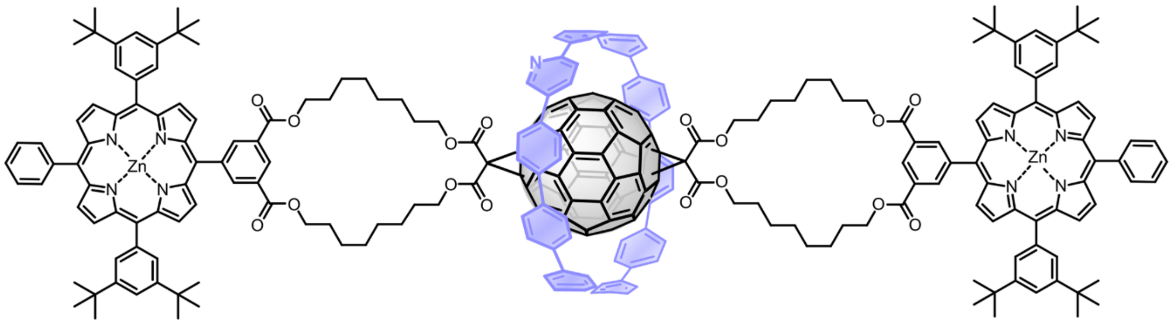

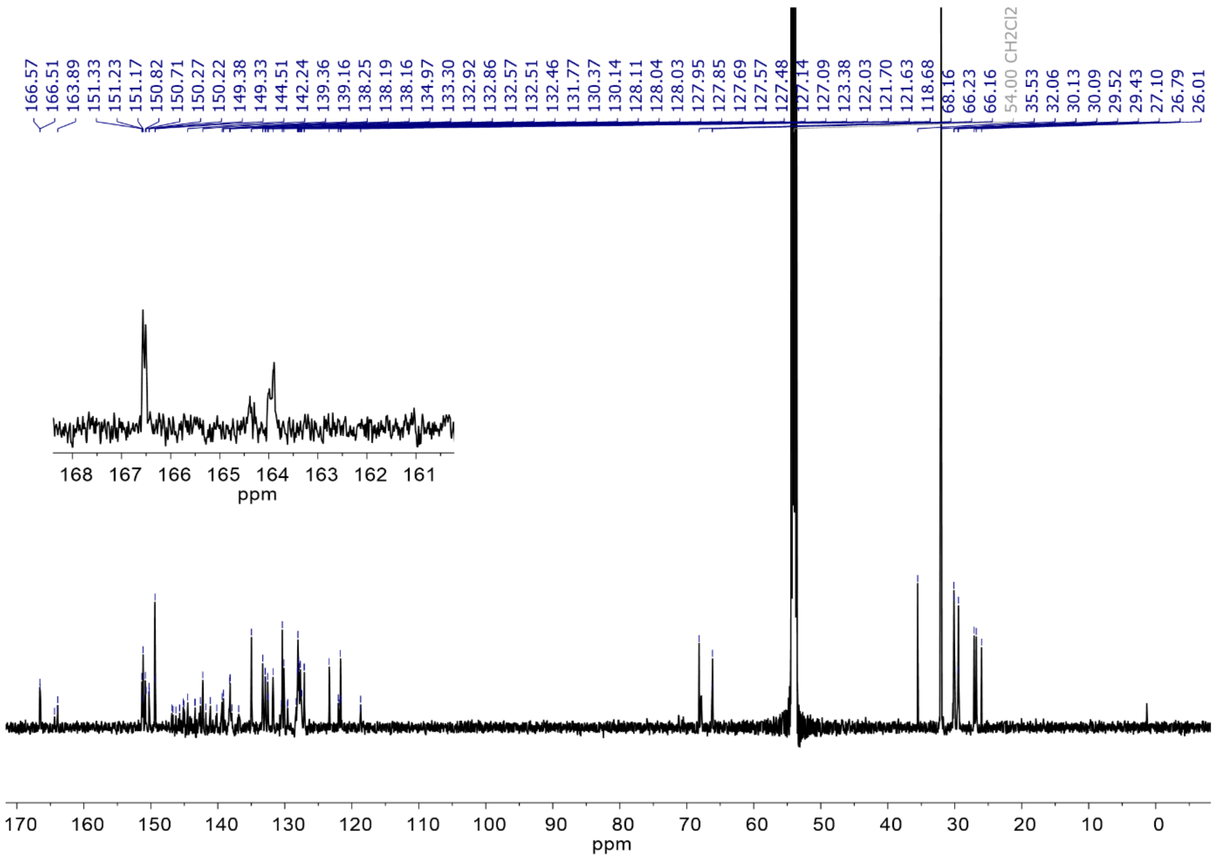

Figure S73: ^{13}C NMR (151 MHz, CD_2Cl_2) of compound **2**.

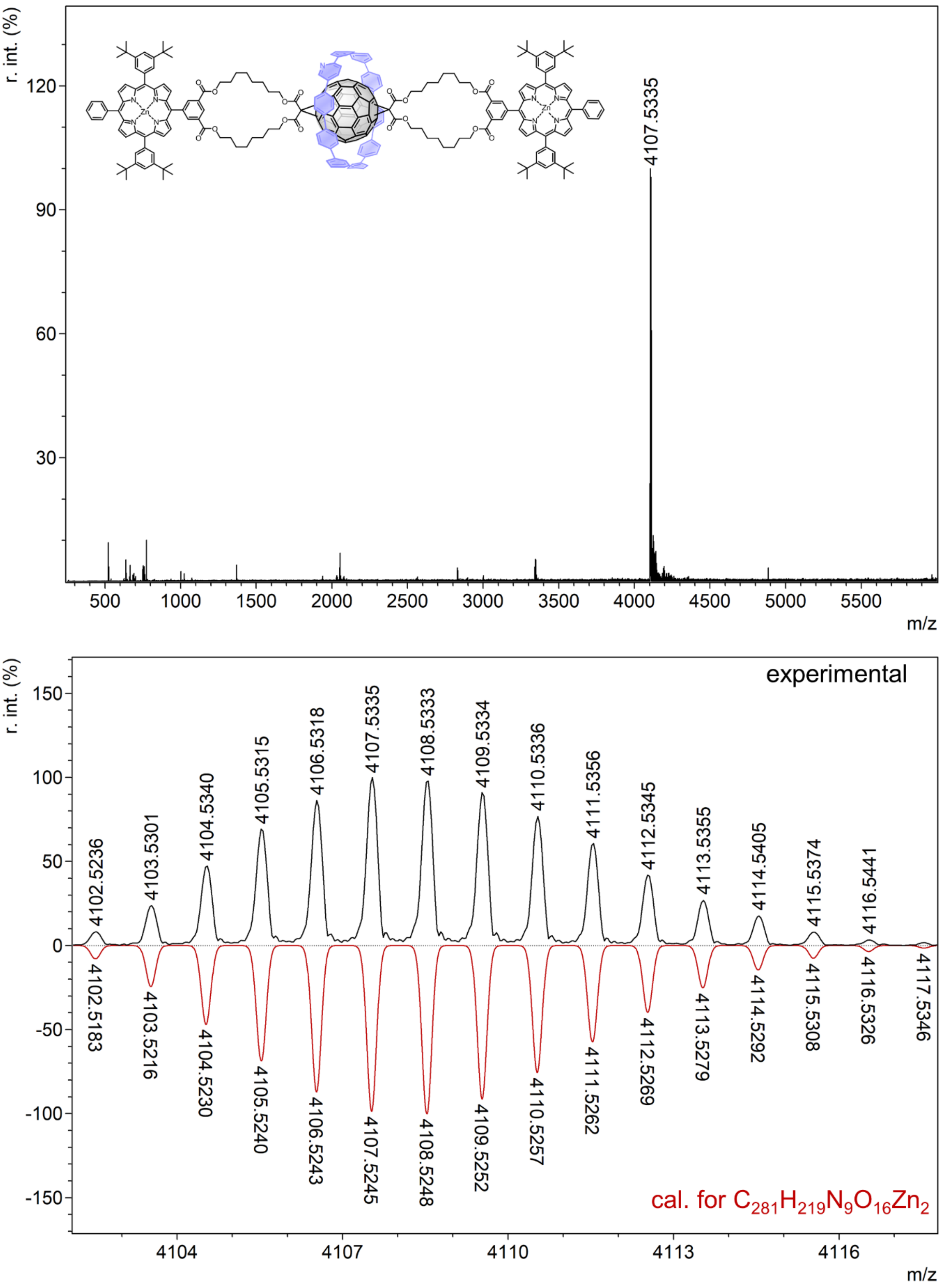

Figure S74: HRMS (MALDI, matrix: DCTB) of compound **2**.

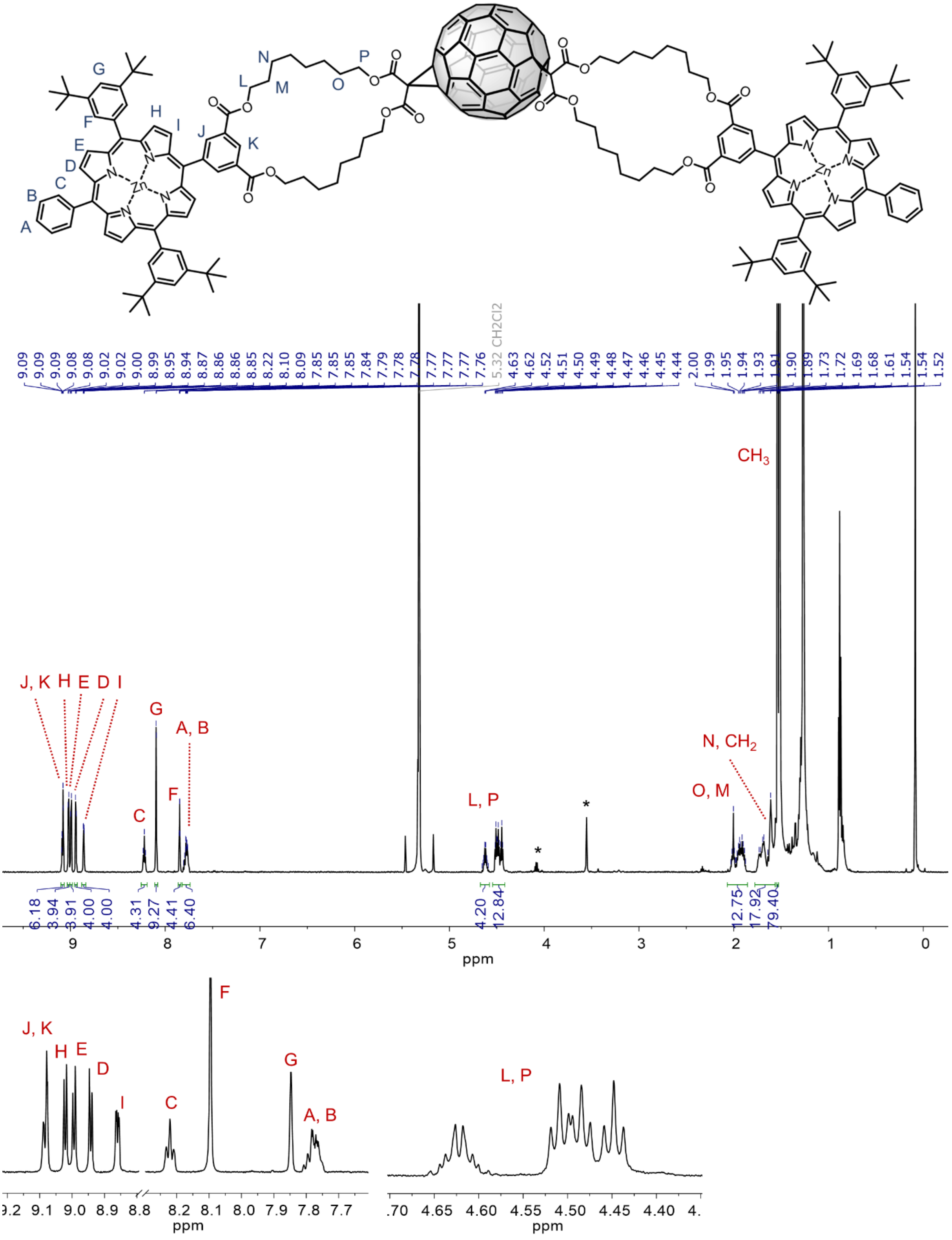

Figure S75 ^{1}H NMR (600 MHz, CD_2Cl_2) of compound ***trans-2*-3.** * indicates unknown minor impurities.

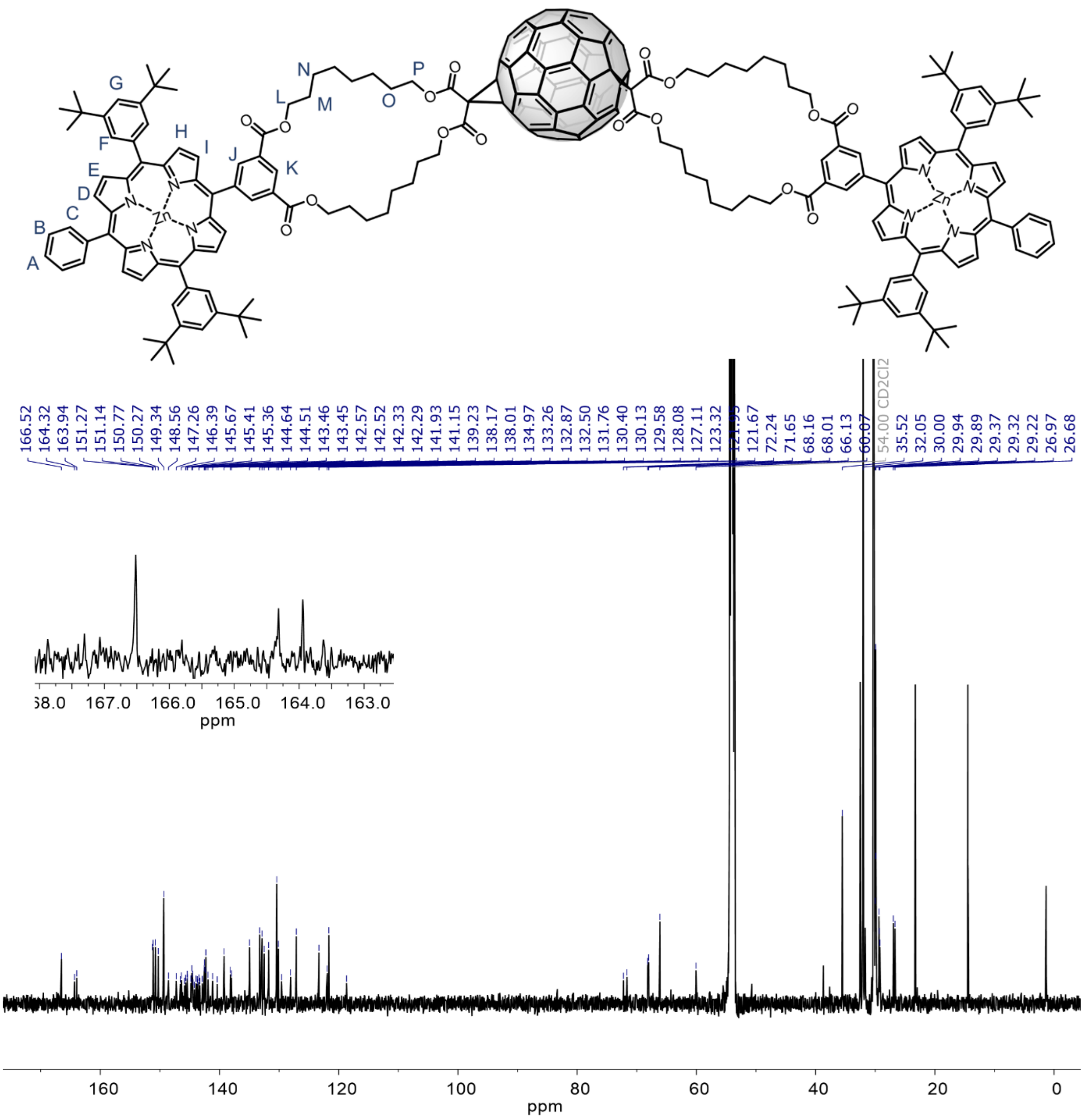

Figure S76: ^{13}C NMR (151 MHz, CD_2Cl_2) of compound ***trans*-2-3**.

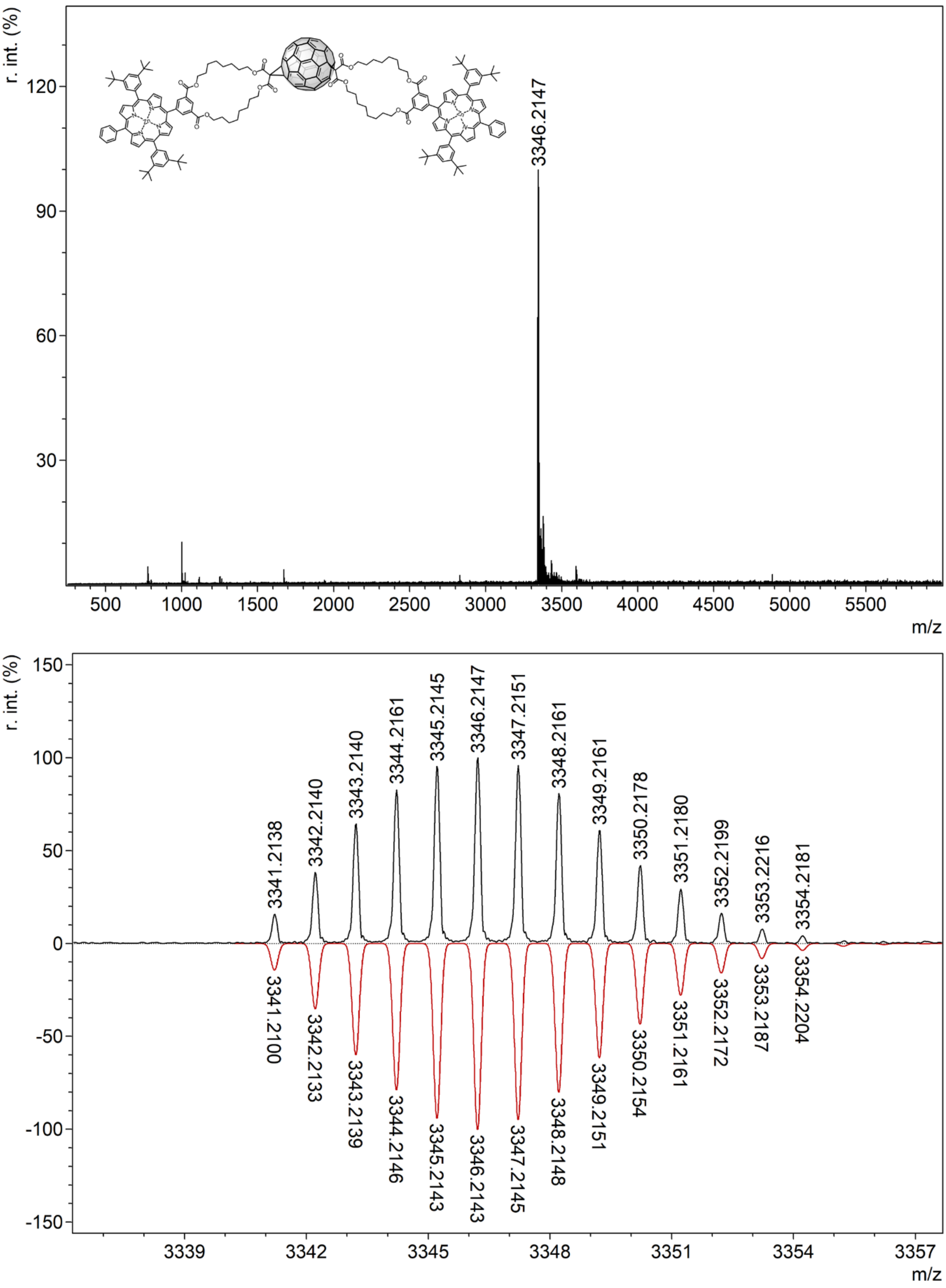

Figure S77: HRMS (MALDI, matrix: DCTB) of compound ***trans-2*-3**.

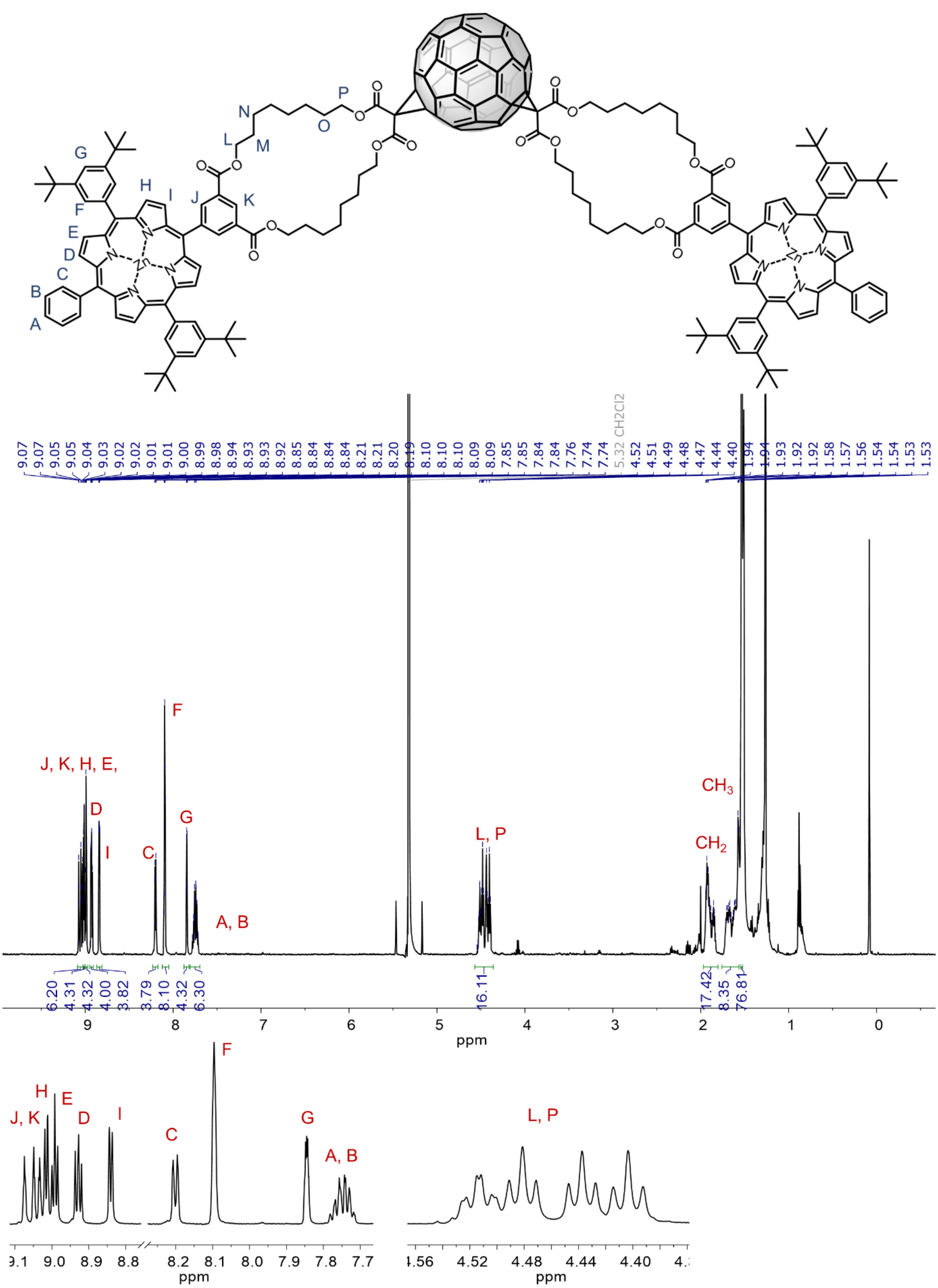

Figure S78: ^{1}H NMR (600 MHz, CD_2Cl_2) of compound ***trans-3*-3.**

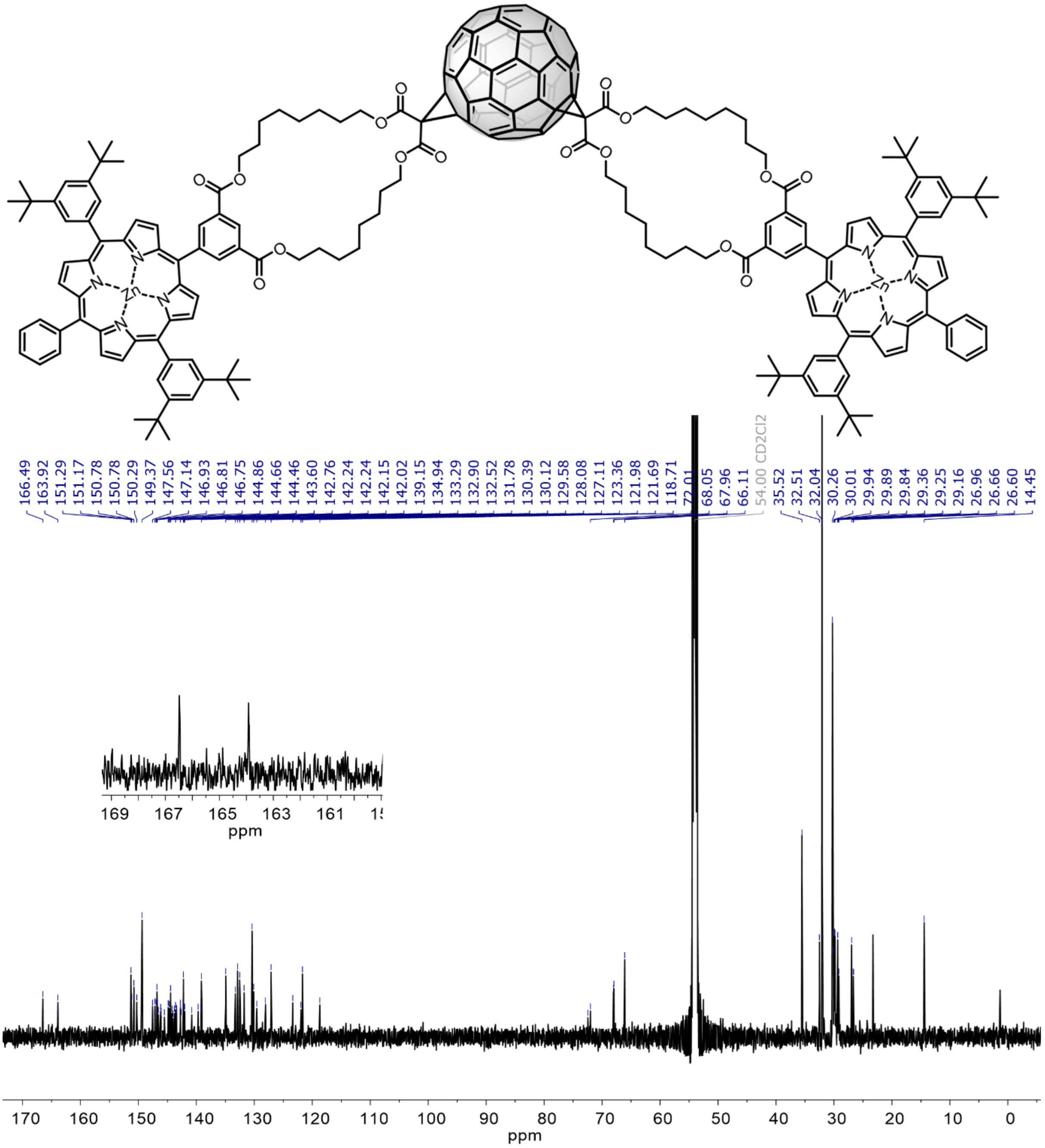

Figure S79: ^{13}C NMR (151 MHz, CD_2Cl_2) of compound ***trans-3*-3**.

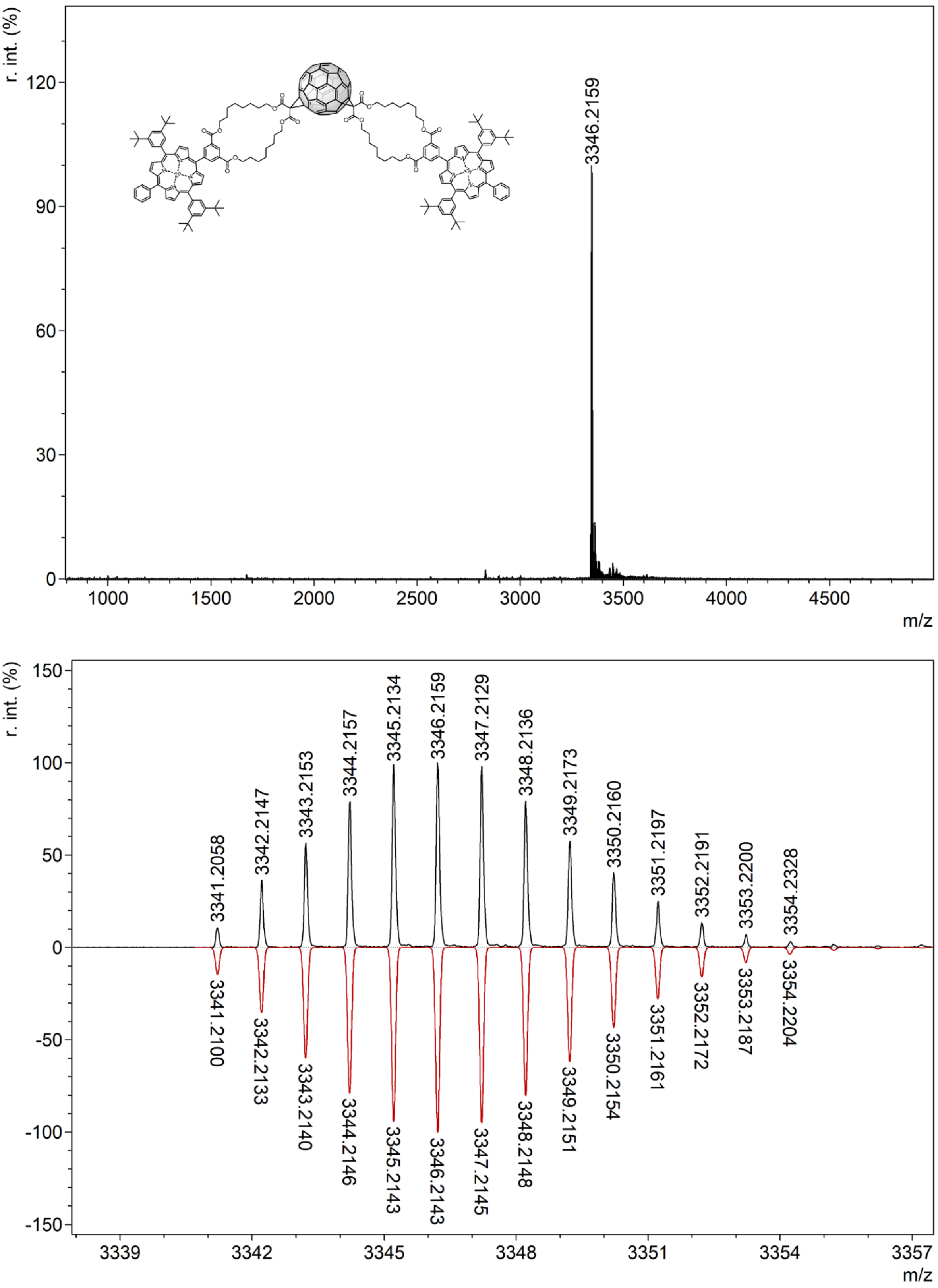

Figure S80: HRMS (MALDI, matrix: DCTB) of compound *trans-3*-**3**.

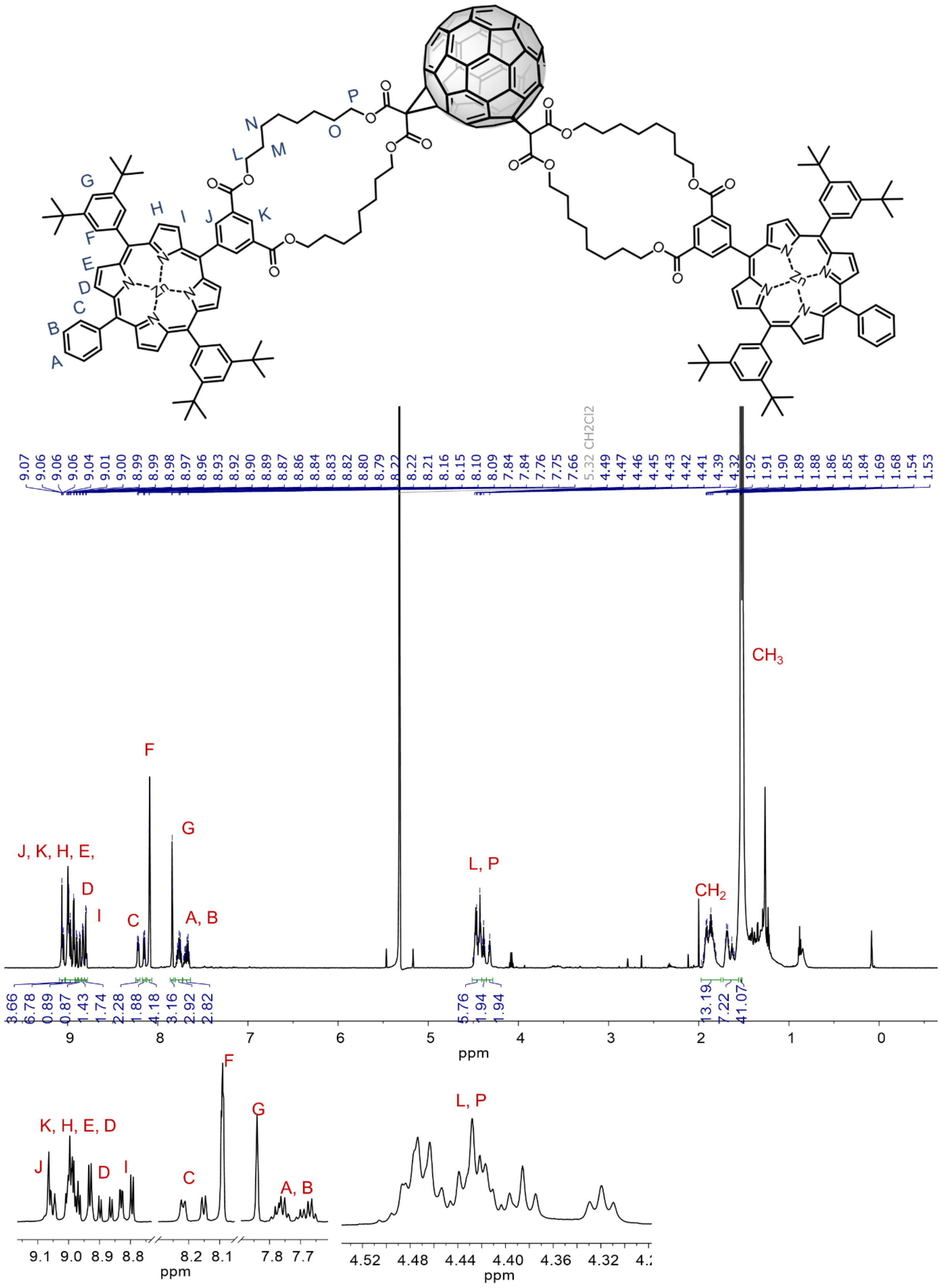

Figure S81: ^{1}H NMR (600 MHz, CD_2Cl_2) of compound ***e*-3.**

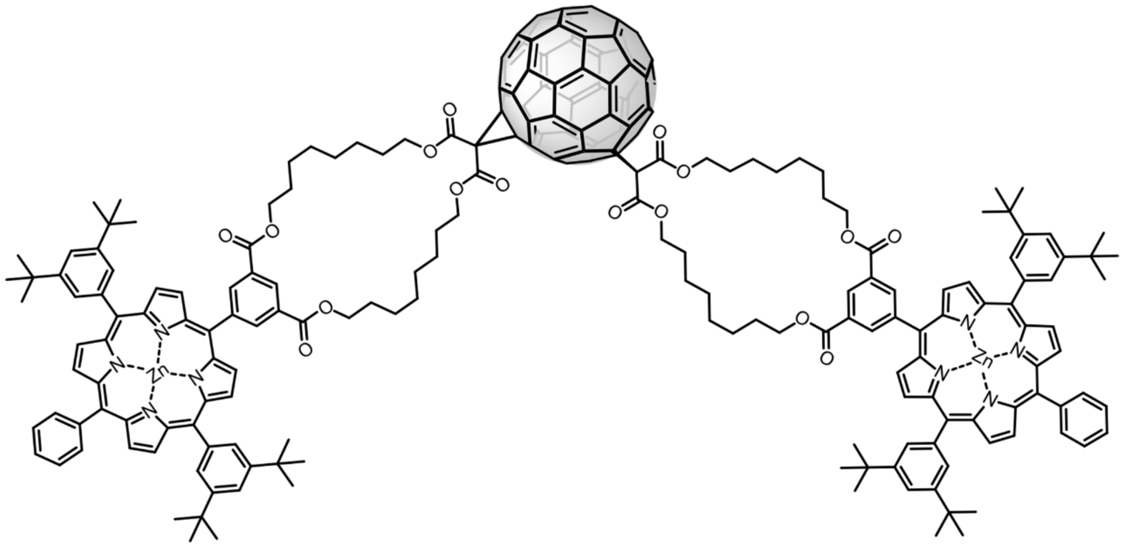

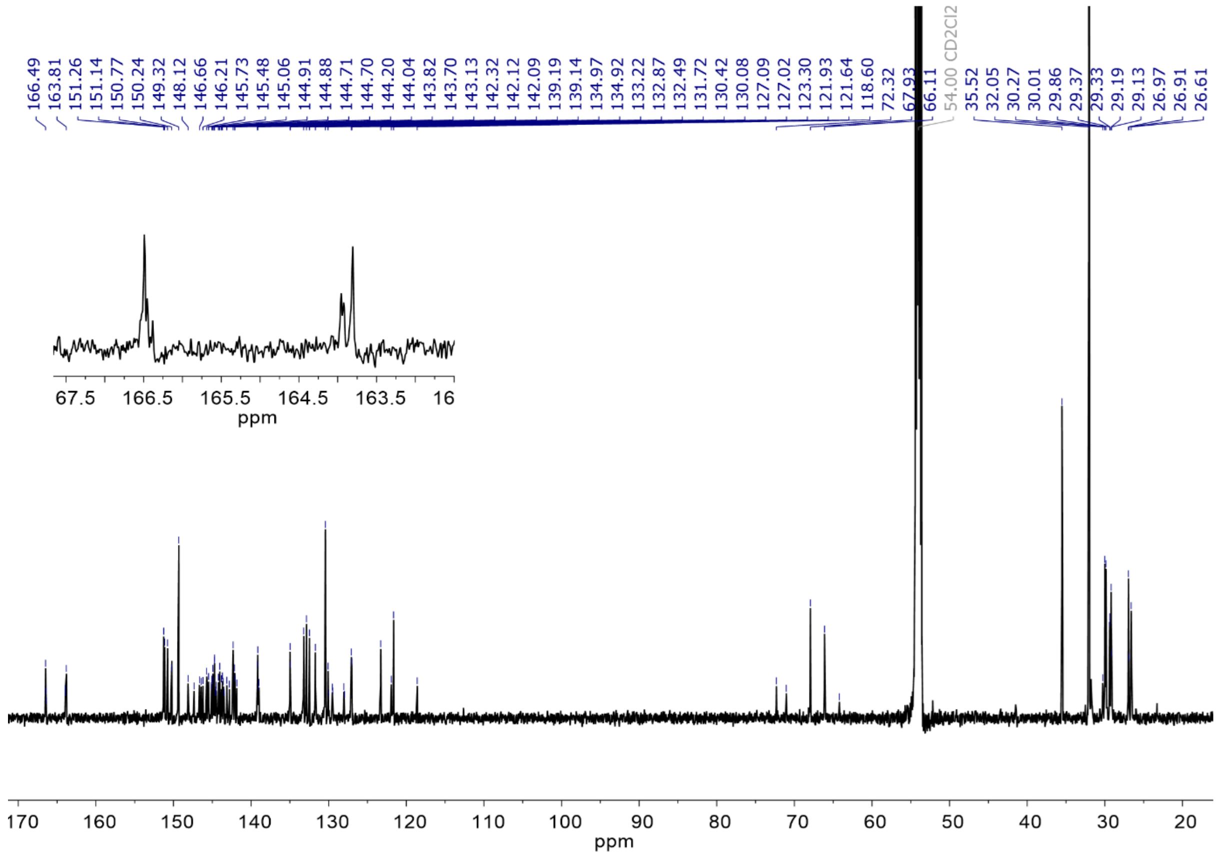

Figure S82: ^{13}C NMR (151 MHz, CD_2Cl_2) of compound ***e*-3**.

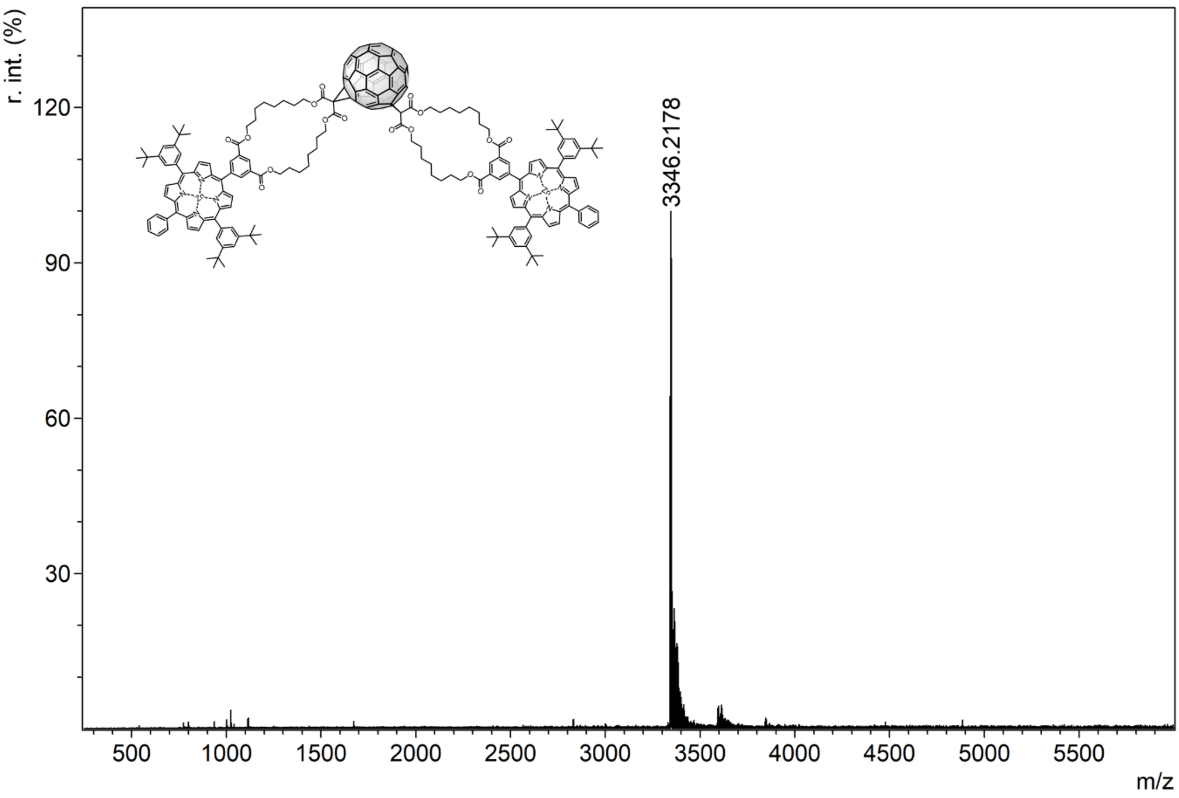

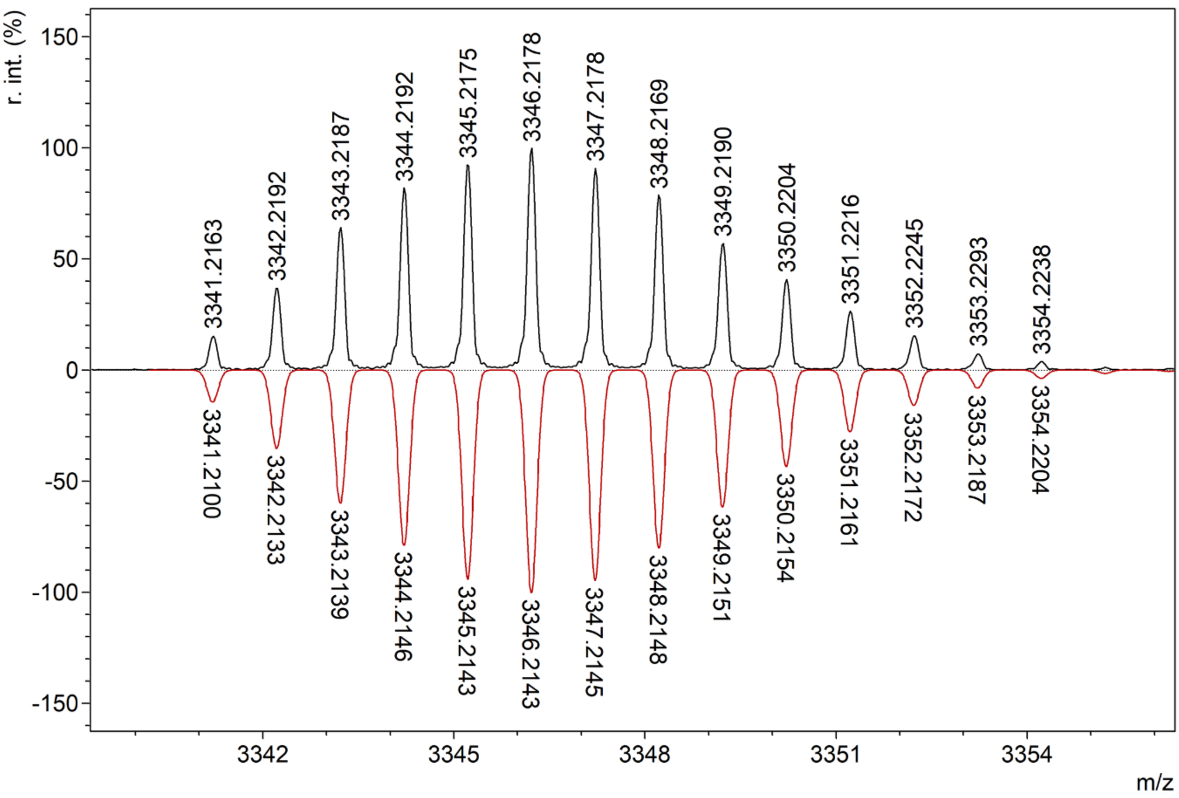

Figure S83: HRMS (MALDI, matrix: DCTB) of compound ***e*-3**.

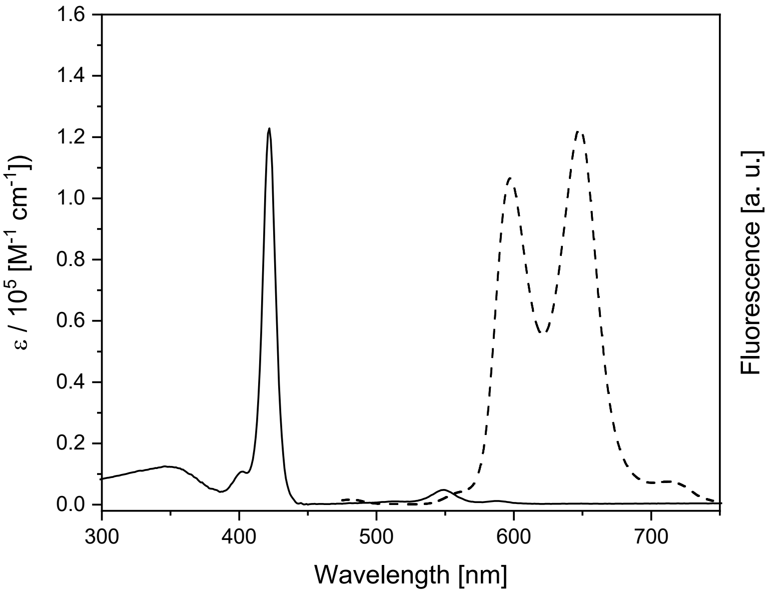

Figure S84: UV/vis absorption (solid line) and fluorescence spectrum (dashed line, excitation at 422 nm) of compound trans-3-**1** in DCM.

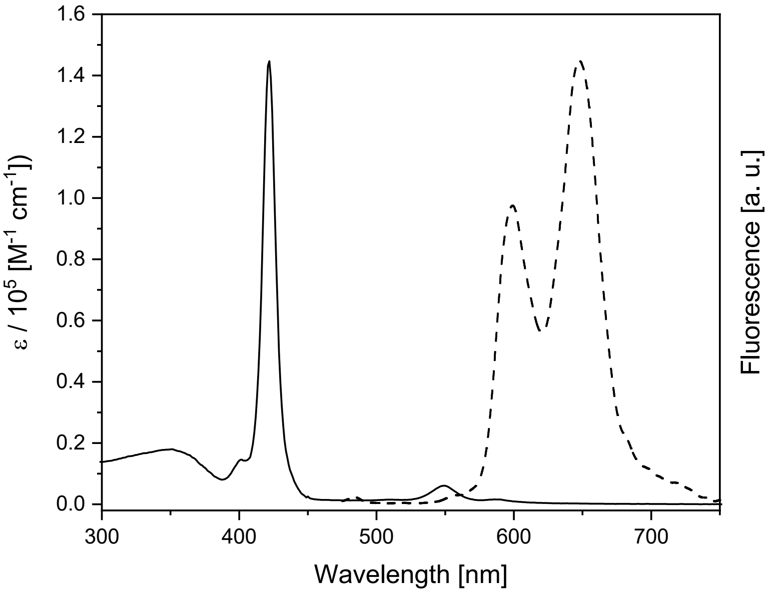

Figure S85: UV/vis absorption (solid line) and fluorescence spectrum (dashed line, excitation at 422 nm) of compound **2** (mixture of isomers) in DCM.

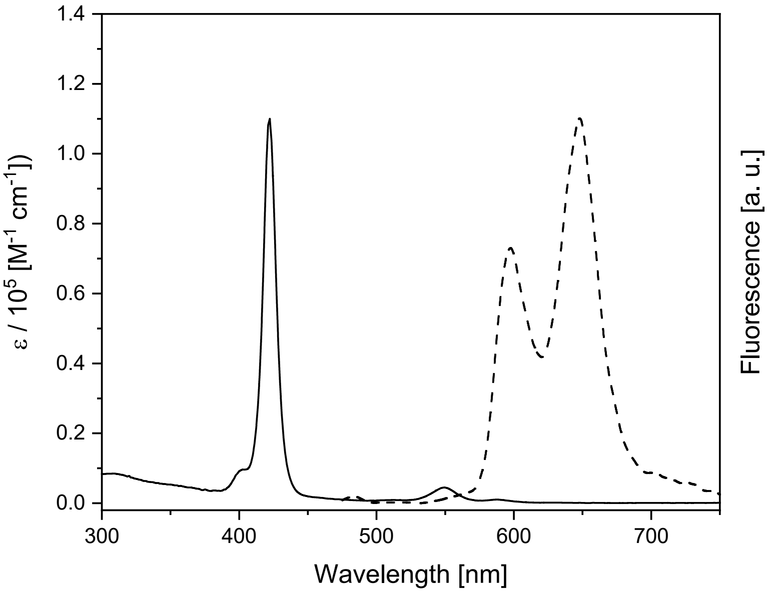

Figure S86: UV/vis absorption (solid line) and fluorescence spectrum (dashed line, excitation at 422 nm) of compound **3** (mixture of isomers) in DCM.

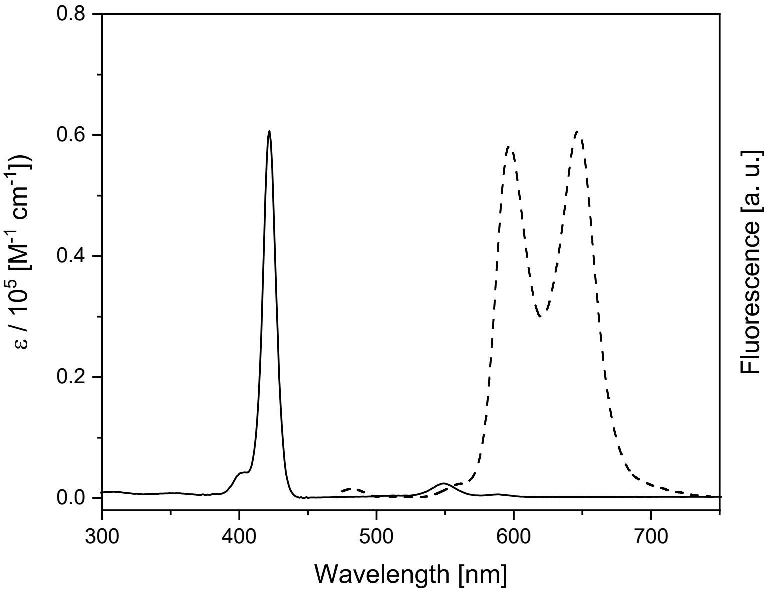

Figure S87: UV/vis absorption (solid line) and fluorescence spectrum (dashed line, excitation at 422 nm) of compound **12** in DCM.

9. References

[1] F. Schwer, S. Zank, M. Freiberger, R. Kaur, S. Frühwald, C. C. Robertson, A. Görling, T. Drewello, D. M. Guldi, M. von Delius, *Org. Mater.* **2022**, *4*, 7–17.

[2] B. T. Ruotolo, J. L. P. Benesch, A. M. Sandercock, S. J. Hyung, C. V. Robinson, *Nat. Protoc.* **2008**, *3*, 1139–1152.

[3] S. M. Stow, T. J. Causon, X. Zheng, R. T. Kurulugama, T. Mairinger, J. C. May, E. E. Rennie, E. S. Baker, R. D. Smith, J. A. McLean, S. Hann, J. C. Fjeldsted, *Anal. Chem.* **2017**, *89*, 9048–9055.

[4] J. J. Snellenburg, S. Laptenok, R. Seger, K. M. Mullen, I. H. M. van Stokkum, *J. Stat. Softw.* **2012**, *49*, 1–22.

[5] A. J. F. N. Sobral, N. G. C. L. Rebanda, M. Da Silva, S. H. Lampreia, M. Ramos Silva, A. Matos Beja, J. A. Paixão, A. M. D. A. Rocha Gonsalves, *Tetrahedron Lett.* **2003**, *44*, 3971–3973.

[6] J. S. Manka, D. S. Lawrence, *Tetrahedronrahedron* **1989**, *30*, 6989–6992.

[7] M. J. Plater, S. Aiken, G. Bourhill, *Tetrahedron* **2002**, *58*, 2405–2413.

[8] M. Séverac, L. Le Pleux, A. Scarpaci, E. Blart, F. Odobel, *Tetrahedron Lett.* **2007**, *48*, 6518–6522.

[9] M. Berthelot, G. Hoffmann, A. Bousfiha, J. Echaubard, J. Roger, H. Cattey, A. Romieu, D. Lucas, P. Fleurat-Lessard, C. H. Devillers, *Chem. Commun.* **2018**, *54*, 5414–5417.

[10] K. J. Thorley, J. M. Hales, H. L. Anderson, J. W. Perry, *Angew. Chem. Int. Ed.* **2008**, *47*, 7095–7098.

[11] Q. Chen, Y. Z. Zhu, Q. J. Fan, S. C. Zhang, J. Y. Zheng, *Org. Lett.* **2014**, *16*, 1590–1593.

[12] P. Pierrat, S. Vanderheiden, T. Muller, S. Bräse, *Chem. Commun.* **2009**, 1748–1750.

[13] F. M. Steudel, E. Ubasart, L. Leanza, M. Pujals, T. Parella, G. M. Pavan, X. Ribas, M. von Delius, *Angew. Chem. Int. Ed.* **2023**, *62*, e202309393.

[14] Y. Xu, R. Kaur, B. Wang, M. B. Minameyer, S. Gsänger, B. Meyer, T. Drewello, D. M. Guldi, M. Von Delius, *J. Am. Chem. Soc.* **2018**, *140*, 13413–13420.

[15] http://supramolecular.org

[16] D. Brynn Hibbert and Pall Thordarson, *Chem. Commun.* **2016**, *52*, 12792–12805.
